# Effect of initiation of antiretroviral drugs for HIV prevention or treatment on the vaginal microbiome of pregnant women in Malawi

**DOI:** 10.1038/s41522-025-00697-8

**Published:** 2025-04-26

**Authors:** Friday Saidi, Lauren A. Graybill, Jennifer H. Tang, Twambilile Phanga, Beteniko Milala, Gabriel Banda, Manley Kamija, Margaret Kasaro, Wilbroad Mutale, Joan Price, Lameck Chinula, Jeffrey Stringer, Mina C. Hosseinipour, Benjamin H. Chi, Jacques Ravel, Johanna B. Holm

**Affiliations:** 1 UNC Project Malawi, Lilongwe, Malawi; 2 Department of Obstetrics and Gynecology, School of Medicine, University of North Carolina at Chapel Hill, Chapel Hill, NC USA; 3 Department of Obstetrics and Gynecology, Kamuzu University of Health Sciences, Blantyre, Malawi; 4 Institute for Global Health and Infectious Disease, University of North Carolina at Chapel Hill, Chapel Hill, NC USA; 5 Department of Health Policy and Systems, University of Zambia School of Medicine, Lusaka, Zambia; 6 Department of Medicine, School of Medicine, University of North Carolina, Chapel Hill, NC USA; 7 Institute for Genome Sciences, University of Maryland School of Medicine, Baltimore, MD USA; 8 Department of Microbiology and Immunology, University of Maryland School of Medicine, Baltimore, MD USA

**Keywords:** Biological techniques, Microbiology

## Abstract

Lack of *Lactobacillus* and/or dysbiosis is linked to spontaneous preterm birth (sPTB). The impact of antiretrovirals (ARVs) for HIV treatment or prevention on the vaginal microbiome during pregnancy remains unclear. We examined vaginal microbiome changes in pregnant women in Lilongwe, Malawi. Women living with HIV (WLHIV) initiated antiretroviral therapy (ART), while HIV-negative women began oral pre-exposure prophylaxis (PrEP). Of 255 participants (191 HIV-negative, 64 WLHIV) who provided baseline vaginal swabs, 181 provided follow-up swabs one month after ARV initiation. At enrollment, WLHIV had higher Shannon diversity and were more likely to have CST IV-B than CST I or III. After ARV initiation, α-diversity decreased in WLHIV but increased in HIV-negative women. Women initiating PrEP had a lower risk of sPTB compared to WLHIV initiating ART, but transitioning to CST IV during pregnancy increased the odds of sPTB. Larger studies are needed to explore ARV impact on pregnancy outcomes.

## Introduction

Deficiency in *Lactobacillus* species in the vaginal microbiota increases the risk of sexually transmitted infections, including HIV, and adverse birth outcomes, such as preterm birth (PTB)^1^. The burden of preterm birth is disproportionately borne by low-income countries like Malawi, where the estimated risk of PTB in the general population is around 19%^2^. Major risk factors for spontaneous PTB (spontaneous labor prior to 37 weeks gestation presenting with or without intact membranes; sPTB) include maternal malnutrition and maternal infection with nearly 10% of the risk attributable to the low-*Lactobacillus* and high diversity state of bacterial vaginosis (BV)^3^. Untreated, advanced HIV disease is associated with adverse birth outcomes^4^. Antiretroviral therapy (ART) adds further complexity to the relationship between HIV infection and birth outcomes as current literature is conflicting on whether ART is associated with sPTB^5^. Whilst the benefit of ARV drugs to both mother and infant is indisputable^6^, evidence on how ART or pre-exposure prophylaxis (PrEP) use during pregnancy affects the vaginal microbiota remains limited, particularly for PrEP use.

To better understand the interconnection between ARV drugs, the vaginal microbiome, and adverse birth outcomes, we characterized the vaginal microbiome of pregnant women living with HIV (WLHIV) and pregnant HIV-negative women. We compared the vaginal microbiome by HIV serostatus prior to initiation of ARVs for either HIV treatment (ART: tenofovir disoproxil fumarate, lamivudine, and dolutegravir) or prevention (PrEP: tenofovir disoproxil fumarate and emtricitabine), and evaluated the effects ART or PrEP initiation. Further, we explored the relationships between changes in the vaginal microbiome after ART or PrEP initiation and sPTB.

## Results

**Fig. 1:**
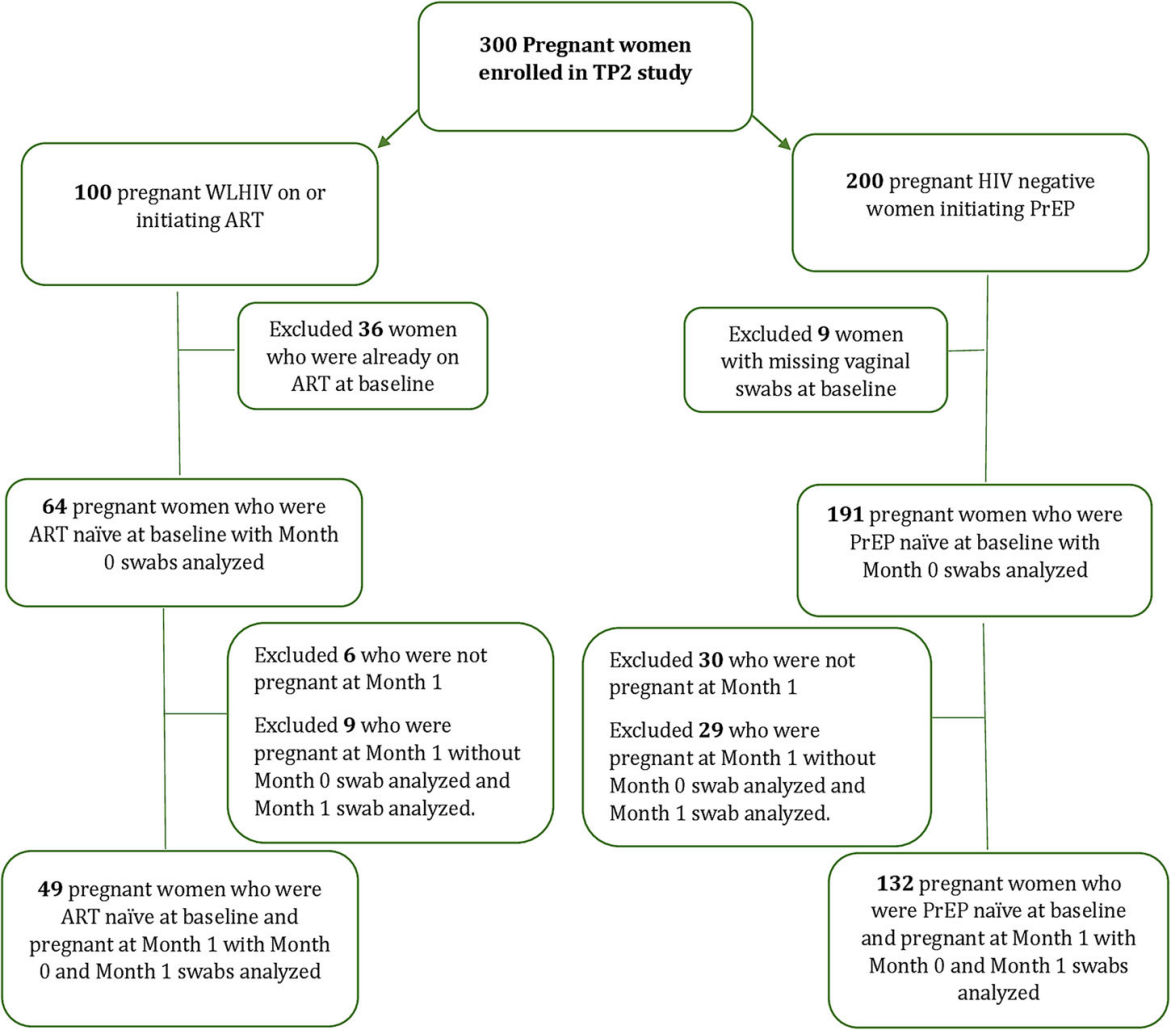
CONSORT flow diagram.

From March 2, 2020 to August 5, 2021, 300 eligible pregnant participants (100 WLHIV and 200 HIV-negative women) were enrolled in the TP2 study (Fig. 1). Among 255 (85%) women with vaginal swabs, 64 were WLHIV and ART naïve, while 191 were HIV-negative and PrEP naïve. Of these, 181 women contributed swabs 4-6 weeks after initiating ARV (132 initiated PrEP, and 49 initiated ART). No seroconversions occurred between M0 and M1. Most participants included in this secondary analysis had gestational age estimated based on last menstrual period (84%) and 16% had dating determined by obstetric ultrasound. At enrollment, HIV-negative women were younger (mean 25 vs. 27 years) than WLHIV (Table 1). No differences were observed in the number of partners in the past three months, consistent condom use with primary partner (Table 1). Data for STI testing were not available. A greater proportion of HIV-negative women received a syphilis diagnosis during the index pregnancy (41% vs. 8%), reported abnormal vaginal discharge in the past three months (42% vs. 3%), and reported genital sores or ulcers in the past three months (19% vs. 5%) than WLHIV.

**Table 1:** Baseline characteristics of pregnant HIV-negative and WLHIV participants from the Tonse Pamodzi 2 cohort included in each analysis

	M0 Analysis			Before and After ARV initiation analysis		
	WLHIV (*N* = 64)	HIV-Negative (*N* = 191)	*P*-value	WLHIV (ART-exposed) (*N* = 49)	HIV-Negative (PrEP-exposed) (*N* = 132)	*P*-value
**Age at M0 (years)**						
Mean (SD)	27.4 (6.02)	25.4 (5.45)	0.02	27.4 (6.19)	25.4 (5.40)	0.05
Median [Min, Max]	26.0 [18.0, 42.0]	24.0 [18.0, 40.0]		26.0 [18.0, 42.0]	25.0 [18.0, 40.0]	
**Gestational age at M0 (weeks)**						
Mean (SD)	23.6 (6.40)	25.6 (8.84)	0.06	22.3 (5.59)	22.8 (7.48)	0.61
Median [Min, Max]	24.0 [10.0, 37.0]	26.0 [6.00, 41.0]		22.0 [10.0, 36.0]	23.0 [9.00, 41.0]	
**Gravidity**						
No prior pregnancies	13 (20.3%)	42 (22.0%)	0.92	11 (22.4%)	31 (23.5%)	1
At least one prior pregnancy	51 (79.7%)	149 (78.0%)		38 (77.6%)	101 (76.5%)	
Primary partner HIV serostatus*						
HIV-negative	13 (20.3%)	139 (72.8%)	< 0.01	9 (18.4%)	96 (72.7%)	< 0.01
WLHIV	11 (17.2%)	9 (4.7%)		8 (16.3%)	7 (5.3%)	
Don’t know	37 (57.8%)	39 (20.4%)		29 (59.2%)	27 (20.5%)	
Missing	3 (4.7%)	4 (2.1%)		3 (6.1%)	2 (1.5%)	
**Number of partners in past three months**						
No partners	3 (4.7%)	4 (2.1%)	0.24	3 (6.1%)	2 (1.5%)	0.14
One partner	61 (95.3%)	182 (95.3%)		46 (93.9%)	127 (96.2%)	
Multiple partners	0	5 (2.6%)		0	3 (2.3%)	
**Consistent condom use with primary partner in past 30 days ^**						
Abstained	11 (17.2%)	26 (13.6%)	0.3	9 (18.4%)	17 (12.9%)	0.39
Never or Sometimes	53 (82.8%)	159 (83.2%)		40 (81.6%)	112 (84.8%)	
Consistent	0	6 (3.1%)		0	3 (2.3%)	
**Diagnosed with syphilis in past 3 months**						
No	59 (92.2%)	109 (57.1%)	< 0.01	47 (95.9%)	73 (55.3%)	< 0.01
Yes	5 (7.8%)	79 (41.4%)		2 (4.1%)	57 (43.2%)	
Missing	0	3 (1.6%)		0	2 (1.5%)	
**Abnormal vaginal discharge observed in past 3 months (self-report)**						
No	61 (95.3%)	111 (58.1%)	< 0.01	47 (95.9%)	78 (59.1%)	< 0.01
Yes	3 (4.7%)	80 (41.9%)		2 (4.1%)	54 (40.9%)	
**Genital ulcers observed in past 3 months (self-report)**						
No	61 (95.3%)	154 (80.6%)	0.01	47 (95.9%)	111 (84.1%)	0.06
Yes	3 (4.7%)	37 (19.4%)		2 (4.1%)	21 (15.9%)	
**Running water in home**						
No	49 (76.6%)	137 (71.7%)	0.55	36 (73.5%)	91 (68.9%)	0.68
Yes	15 (23.4%)	54 (28.3%)		13 (26.5%)	41 (31.1%)	
**Pregnancy Outcome**						
Term Delivery	48 (75.0%)	159 (83.2%)	0.17	44 (89.8%)	121 (91.7%)	0.73
Preterm Delivery	7 (10.9%)	10 (5.2%)		4 (8.2%)	7 (5.3%)	
Missing	9 (14.1%)	22 (11.5%)		1 (2.0%)	4 (3.0%)	
**Days between M0 and M1**						
Mean (SD)				28.2 (2.85)	29.6 (5.36)	0.02
Median [Min, Max]				28.0 [19.0, 40.0]	29.0 [18.0, 73.0]	

*Restricted to participants who reported at least one male partner in past three months (*N* = 61 WLHIV; *N* = 187 Women who tested HIV-negative during ANC)

^ Restricted to participants who reported vaginal or anal sex in the last 30 days (*N* = 50 WLHIV; *N* = 165 Women who tested HIV-negative during ANC)

**Fig. 2:**
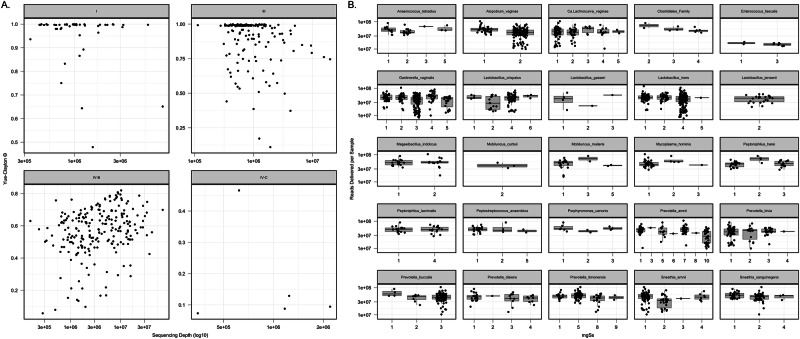
Community State Types (CST) and Metagenomic Assignments. CST (**A**) and mgSs (**B**) assignments were not related to sequencing depth.

**Fig. 3:**
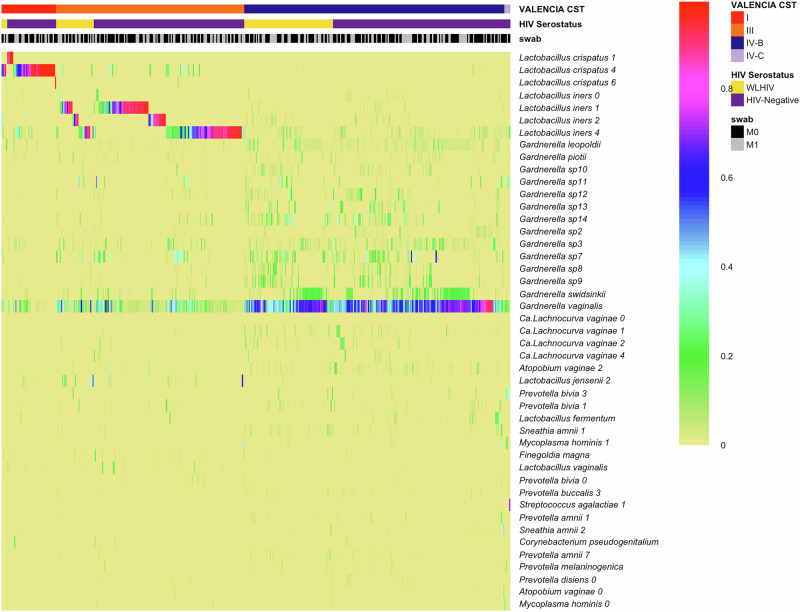
The proportion of the metagenomic subspecies (mgSs) in each sample. Samples (columns) are assigned to a community state types (CST) using VALENCIA^25^ based on species compositions inferred from mgSs of vaginal microbiomes, and were colored by M0 HIV serostatus (WLHIV: Women Living With HIV). The 45 most abundant mgSs are shown.

**Fig. 4:**
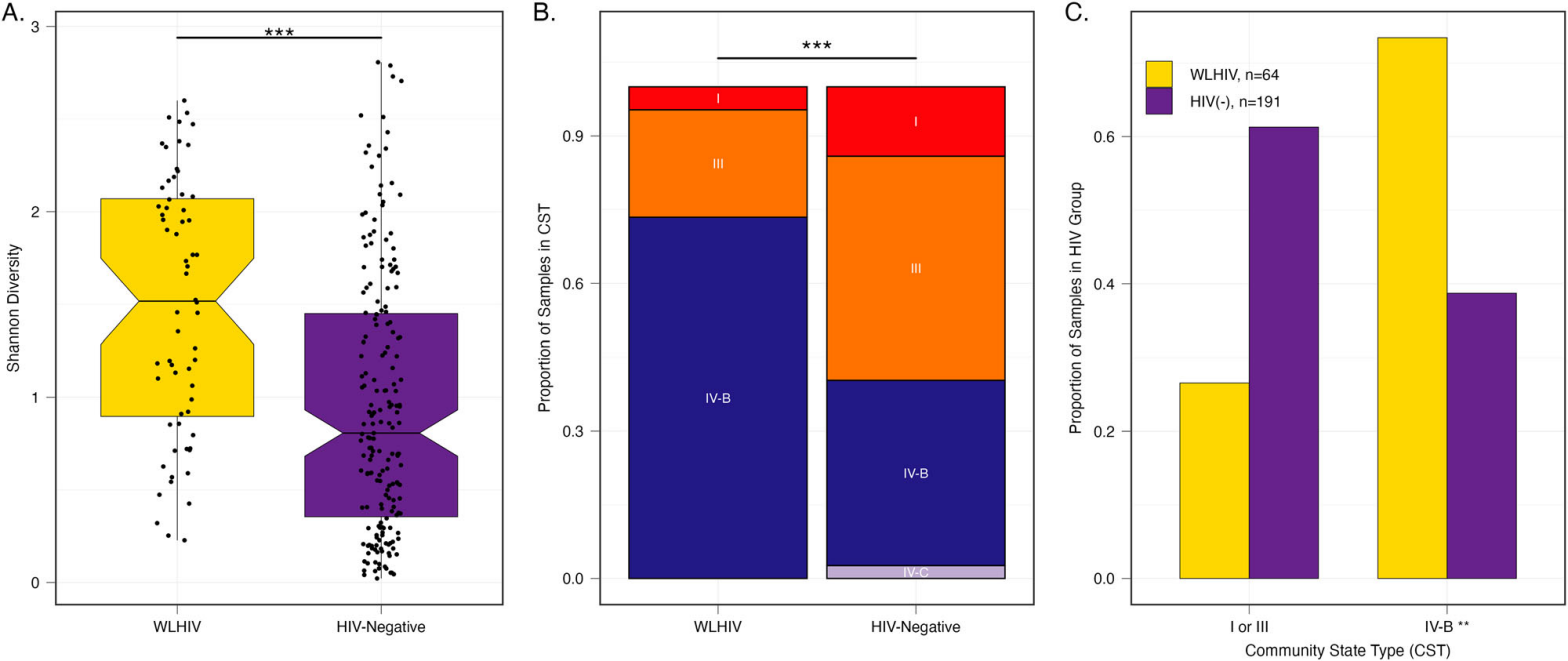
Baseline Differences in Alpha Diversity and CST Distribution Between WLHIV and HIV-negative women. Alpha-diversity is higher at M0 in WLHIV compared to HIV-negative women as measured by Shannon diversity indices (**A**). **B** CST IV-B and III were the most prevalent CSTs in WLHIV and HIV-negative women at M0, respectively. **C** There were more HIV-negative participants in CST I and III and more WLHIV participants in CST IV-B. (*p < 0.05, **p < 0.01, ***p < 0.001).

**Fig. 5:**
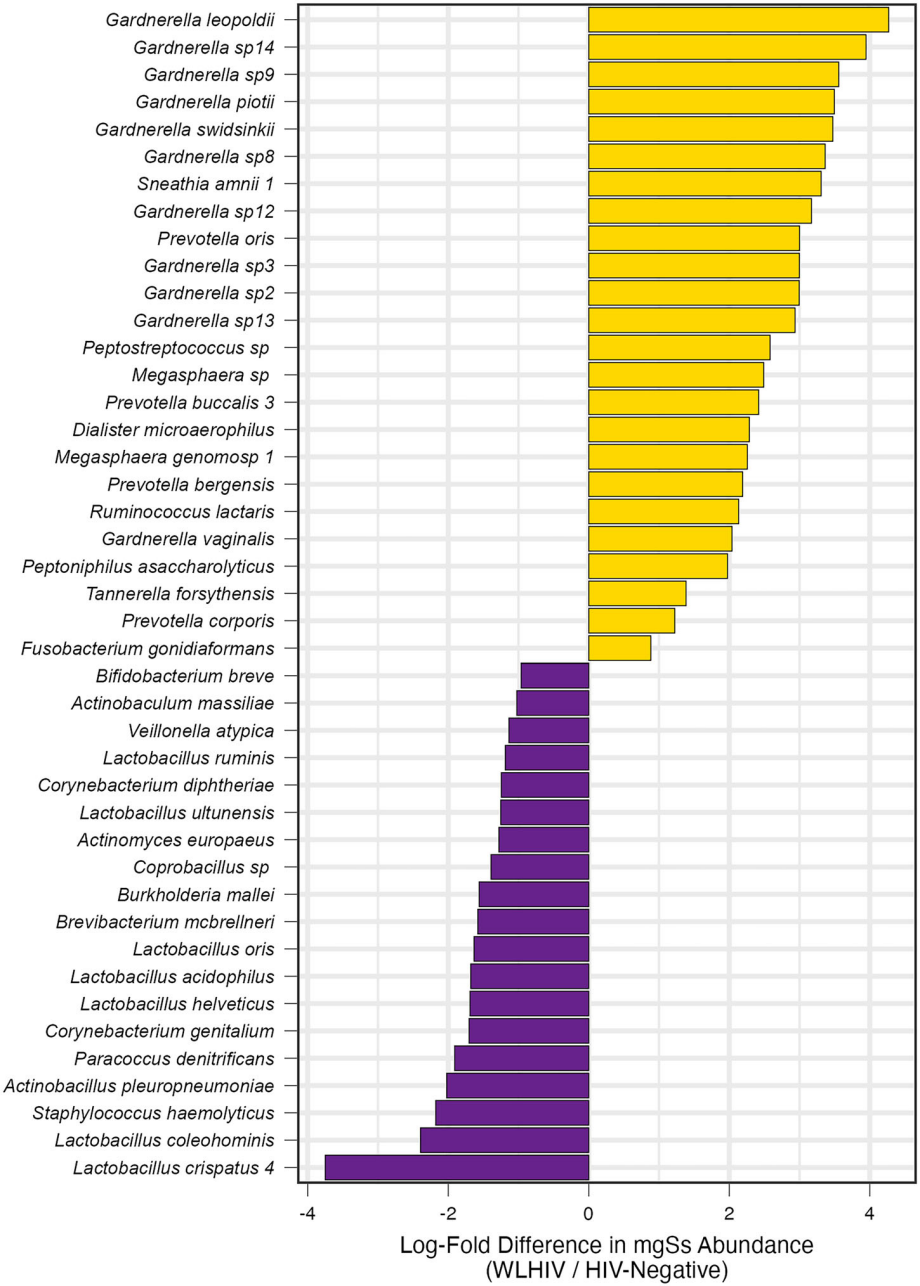
Baseline Differences in Vaginal Metagenomic Subspecies Abundances by HIV serostatus. Metagenomic subspecies (mgSs) are associated with M0 HIV serostatus. The abundances of mgSs were significantly greater (yellow) or lower (purple) in WLHIV compared to HIV-negative women at M0. Only those with adjusted *p*-values < 0.01 are shown. < 0.05, **p < 0.01, ***p < 0.001).

In total, 436 samples yielded high-quality metagenomic sequence reads with a mean 4·1 million microbial sequence reads per sample; mgSs and CST assignments were not dependent on sequencing depth (Fig. 2). Analyses of the vaginal microbiota composition and abundance in non-pregnant and pregnant women have defined five major types, termed community state types (CSTs)^7–10^. CST II, predominated by *L. gasseri*, was not observed in this study (Fig. 3). At M0, the median Shannon diversity of all vaginal microbiomes was 0·8. Shannon diversity was higher among WLHIV compared to HIV-negative women (1·5 vs. 0·7, *p* < 0.001; Fig. 4A), and HIV serostatus was associated with CST (*p* < 0·001; Fig. 4B). The odds of having CST IV-B were 4-times greater than CST I or III among WLHIV compared to HIV-negative women (aOR: 4·0, 95% CI: 1·7, 9·8; Fig. 4C). HIV serostatus at M0 was also associated with the abundances of certain metagenomic subspecies (*p* < 0·01; Fig. 5). WLHIV were more likely to harbor a variety of *Gardnerella* species including *G. leopoldii*, *G*. sp 14, and *G. swidsinksii* as well as *Sneathia amnii* mgSs 1 and *Prevotella oris* compared to HIV-negative women. *L. crispatus* mgSs 4 was more abundant in the vaginal microbiota of HIV-negative women.

**Fig. 6:**
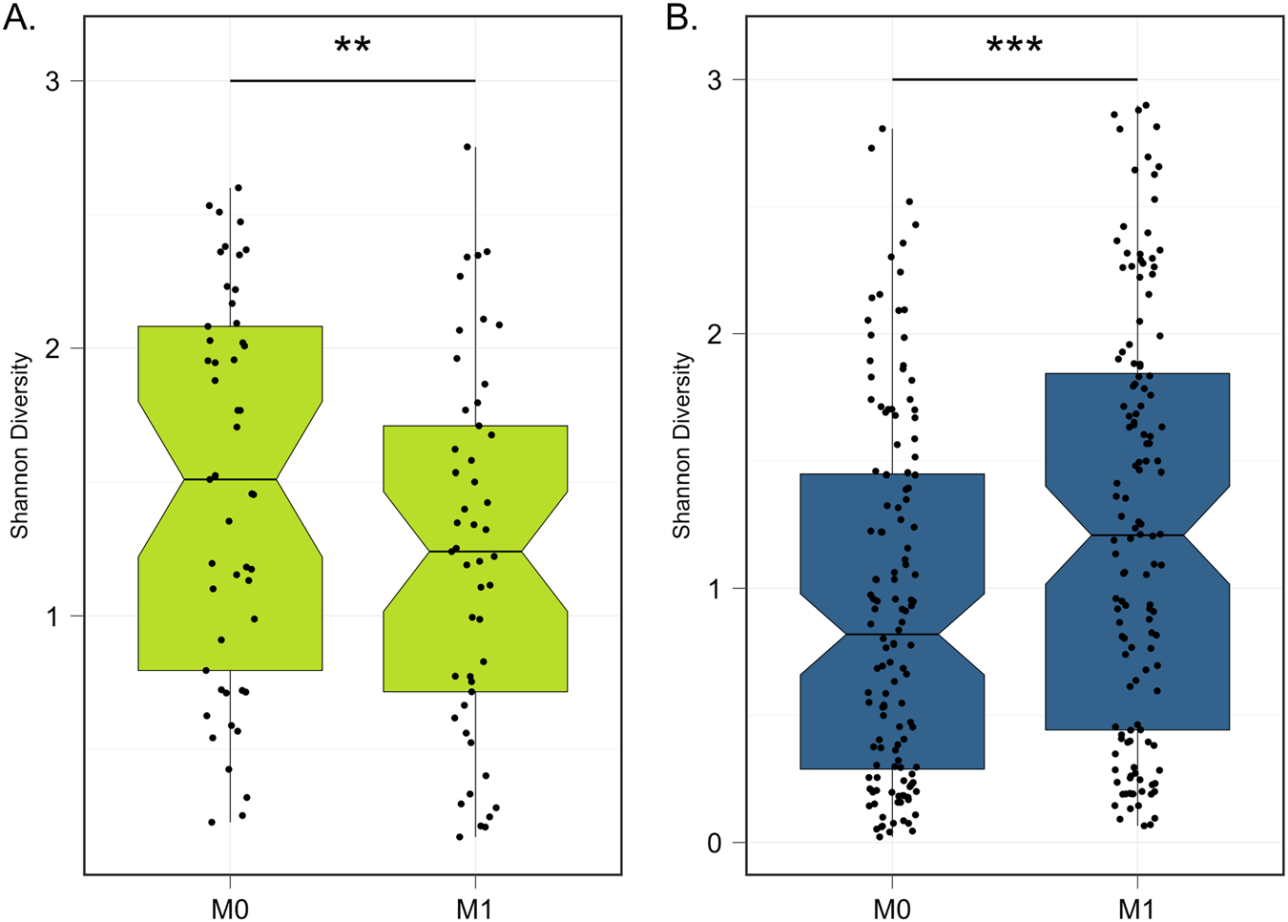
Changes in Alpha Diversity at Month 1 Following ART or PrEP Initiation among WLHIV and HIV-negative Women. **A** At M1 following initiation of ART, alpha diversity was 24% lower among WLHIV (*p* = 0.006), while (**B**) alpha diversity was 27% higher among HIV-negative participants at M1 after initiating PrEP (*p* < 0.001).

**Fig. 7:**
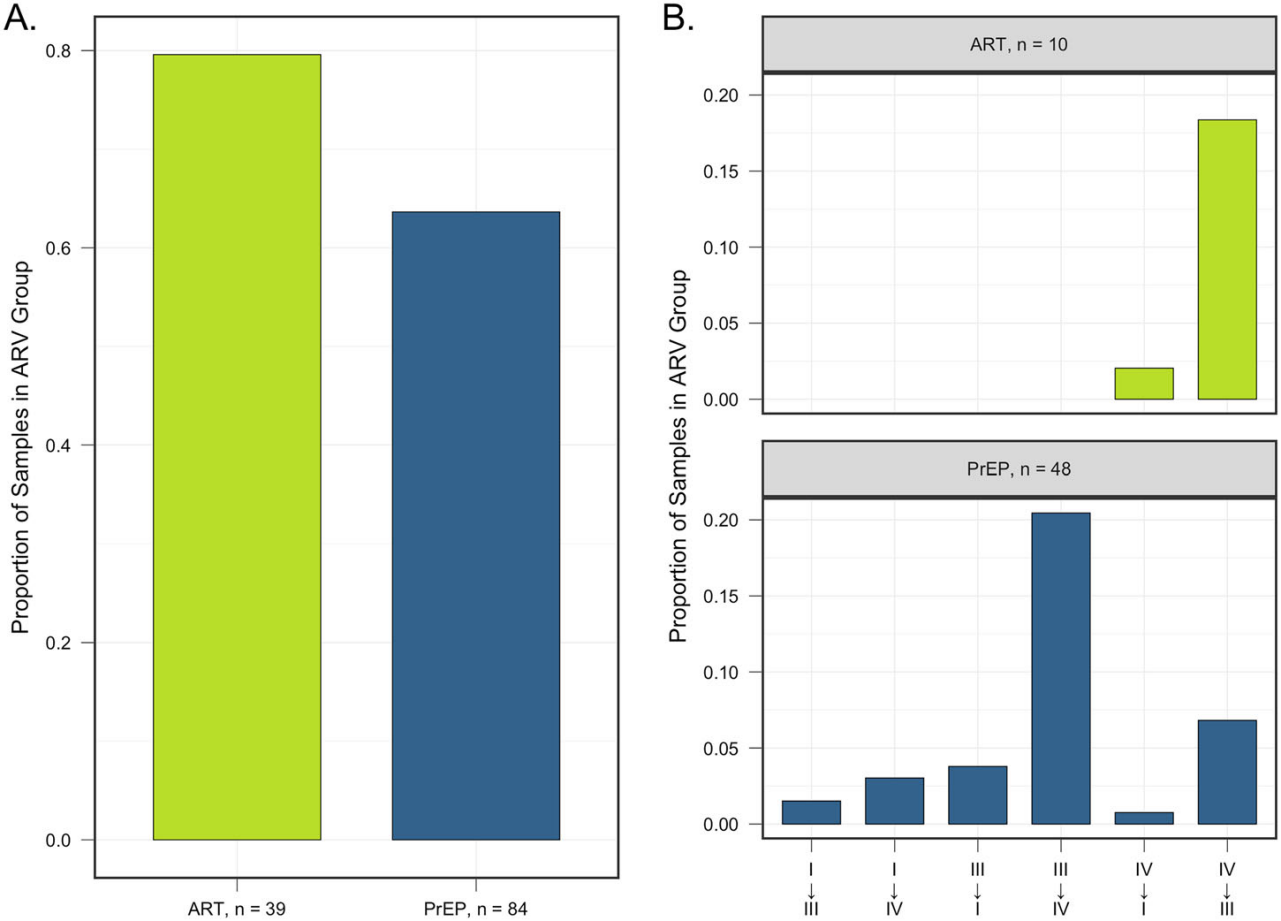
CST Stability and Direction of CST Shifts at Month 1 following ARV Initiation Among ART and PrEP Users. **A** The proportion of participants that did not change CST after ARV initiation at M1 was similar among ART and PrEP users. **B** ART users that changed CSTs most frequently between M0 and M1 switched from CST IV-B to CST III. Conversely, PrEP users that changed CSTs were most likely to switch from CST III to CST IV-B.

**Fig. 8:**
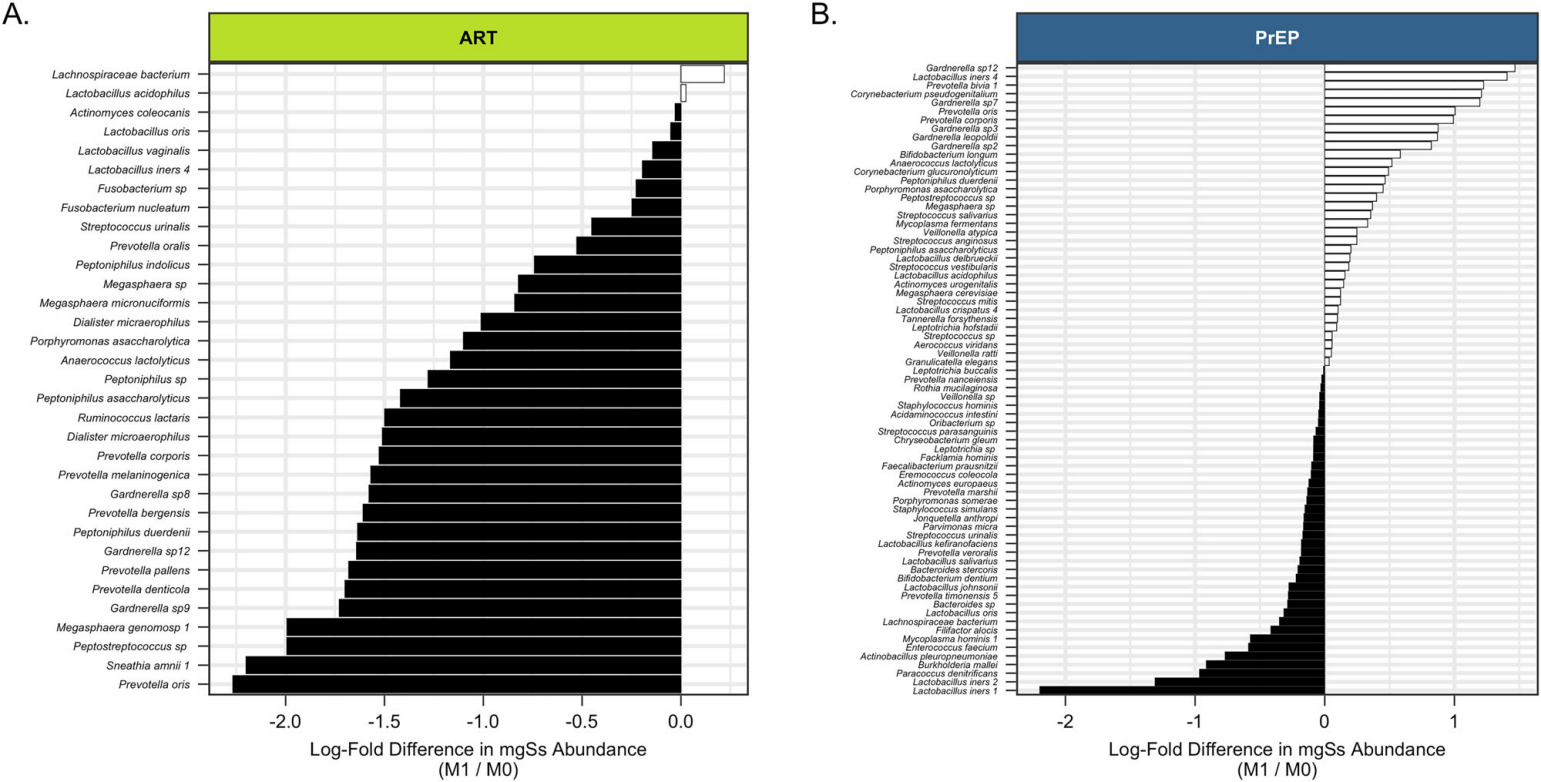
Shifts in Vaginal Microbial Strain Abundance at Month 1 following ART or PrEP Initiation in Pregnant Women. **A** Among pregnant WLHIV, the abundances of a four mgSs decreased at M1 after ART initiation. **B** Among HIV-negative pregnant women, the abundances of several mgSs increased or decreased at M1 after PrEP initiation. All taxa plotted were significant (*p*_adj_ < 0.05).

A subset of participants (*n* = 181) contributed a second swab following ART (*N* = 49) or PrEP (*N* = 132) initiation. Of these, the median time since initiation was 28 days (19–40) and 29 days (18-73), respectively. On average, Shannon diversity at M1 decreased from M0 by 24% in WLHIV (Fig. 6A, *p* < 0·01), and increased by a mean of 27% among HIV-negative (*p* < 0·001; Fig. 6B). At M1, WLHIV were less likely to have transitioned to another CST (10/49, 20%) than HIV-negative women who initiated PrEP (48/132, 37%) (aOR: 0·3, 95% CI: 0·1, 0·9; Fig. 6A). When transitions occurred, the majority of WLHIV experienced transitions from CST IV to CST III after ART initiation (9 out of 11, or 81%; Fig. 7B). This shift was associated with notable reductions in the abundances of several mgSs, including *Prevotella*, *Gardnerella*, and *Peptostreptococcus* species (Fig. 8A). Following initiation of PrEP, most HIV-negative women went from CST III to IV (27/48, 55%) (Fig. 7B) characterized by increases in abundances of *Gardnerella* sp 12, *L. iners* mgSs 4, *Prevotella bivia* mgSs 1 and *P. oris*, while *L. iners* mgSs 1 and mgSs 2 decreased (*p* < 0·05; Fig. 8B).

To estimate the odds of transitioning to CST IV at M1 following PrEP exposure, a sub-cohort of 196 HIV-negative, pregnant women unexposed to PrEP and with less than 41 days between visits from the ZAPPS study was used^11^. The ZAPPS sub-cohort resembled the TP2 cohort in participant age and syphilis test results at enrollment but were further along in their pregnancies (28 vs 23 weeks), all had prior pregnancies, and were more likely to have bottled or running water at home (Table 2). The proportion of participants that maintained the same CST from M0 to M1 was also similar in both cohorts (TP2: 64%, ZAPPS: 73%; Table 2). Pregnant individuals exposed to PrEP in the TP2 study had significantly higher odds of experiencing a transition to CST IV (31/47, 66%) than those not exposed to PrEP in the ZAPPS sub-cohort (10/55, 18%) (aOR: 6·1, 95% CI: 1·3−30).

**Table 2:** Baseline demographic information for sub-analysis comparing the ARV-exposed Tonse Pamodzi 2 cohort to the ARV-unexposed ZAPPS cohort. Included participants were HIV-negative, pregnant, and had less than 44 days between M0 and M1

	Tonse Pamodzi 2 (PrEP-exposed)(*N* = 132)	ZAPPS (PrEP-unexposed) (*N* = 237)	*P*-value
**Age at M0 (years)**			
Mean (SD)	25.5 (5.40)	26.1 (5.21)	0.27
Median [Min, Max]	25.0 [18.0, 40.0]	26.0 [15.0, 40.0]	
**Gestational age at M0 (weeks)**			
Mean (SD)	22.8 (7.47)	24.5 (4.66)	0.03
Median [Min, Max]	23.0 [9.00, 41.0]	27.5 [16.4, 30.0]	
**Gravidity**			
No prior pregnancies	29 (22.3%)	0 (0.0%)	< 0.01
At least one prior pregnancy	101 (77.7%)	196 (100.0%)	
**Diagnosed with syphilis in past 3 months**			
No	73 (56.2%)	127 (64.8%)	0.15
Yes	57 (43.8%)	69 (35.2%)	
**Running water in home**			
No	89 (68.5%)	2 (1.0%)	< 0.01
Yes	41 (31.5%)	194 (99.0%)	
**Days between M0 and M1**			
Mean (SD)	29.6 (5.40)	30.2 (5.26)	0.34
Median [Min, Max]	29.0 [18.0, 73.0]	29.0 [19.0, 40.0]	
**CST between M0 and M1**			
Changed	46 (35.4%)	53 (27.0%)	0.14
No Change	84 (64.6%)	143 (73.0%)	

Among all TP2 participants in this analysis, pregnancy outcomes were available for 224. Among 55 WLHIV with available pregnancy outcomes, 7 delivered pre-term (12%) and among 169 HIV-negative women, 10 delivered preterm (6%). Thus, the odds of sPTB were three times greater among WLHIV initiating ART compared to HIV-negative women initiating PrEP (aOR: 3·0, 95% CI: 0·6, 17). Any woman with CST IV detected at either M0 or M1 had similar odds of sPTB as women who had no CST IV during pregnancy (9/138 vs. 8/85; aOR: 0·9, 95% CI: 0·3, 2·9). Among women with available pregnancy outcome data who contributed swabs at both M0 and M1 (*n* = 176), those who changed CSTs during the study period had similar odds of sPTB as those who did not (4/58 vs. 7/118; aOR: 1·2, 95% CI: 0·2, 5·1). However, when a woman transitioned from non-CST IV at M0 to CST IV at M1 (which occurred only among HIV-negative women initiating PrEP), the odds of sPTB trended towards 4-fold greater (3/31, 10%) than term delivery, relative to those that experienced any other or no CST transition during pregnancy (4/97, 4%) (aOR: 4·0, 95% CI: 0·5, 34). Notably, these estimates are not precise.

## Discussion

In this analysis we characterized the vaginal microbiome of pregnant WLHIV and HIV-negative pregnant women who initiated ARV drugs during pregnancy. To the best of our knowledge, this is the first study to longitudinally characterize the vaginal microbiome during pregnancy in the context of antiretroviral drug initiation for both HIV treatment and prevention among pregnant women. Pregnant individuals exposed to PrEP in the TP2 study had significantly higher odds of experiencing a transition to CST IV than those not exposed to PrEP in the ZAPPS sub-cohort. The odds of sPTB were three times greater among WLHIV initiating ART compared to HIV-negative women initiating PrEP.

Following ARV initiation, most participants did not experience shifts in their vaginal microbiome after starting ARV drugs regardless of HIV serostatus. These findings align with previous studies in pregnant women that indicated that standard HIV antiretroviral regimens (tenofovir disoproxil fumarate + lamivudine + dolutegravir) may not directly disrupt vaginal microbiome composition^12^. For example, initiation of TDF-based ART showed no significant changes in vaginal bacterial community structure in African cohorts ^12^. Similarly, oral PrEP with daily TDF–emtricitabine did not cause adverse shifts in vaginal microbiota in women, with longitudinal analyses showing Lactobacillus-dominated communities remaining stable under PrEP^13^. However, among WLHIV in our cohort the most common transition of the vaginal microbiome after initiating antiretroviral therapy (ART) was from *Gardnerella* spp. (CST IV-B) to *L. iners* (CST III) predominance. This transition was the result of reductions in the relative abundances of multiple metagenomic subspecies (mgSs) of *Prevotella* and *Gardnerella* spp., producing a proportional increase in the relative abundance of *L. iners*, with the exception of *L. iners* mgSs 4, a mgSs shown to be associated with bacterial vaginosis^14^. A transition to a *Lactobacillus*-dominant vaginal microbiota among WLHIV following ART initiation indicates the optimization of the vaginal microbiome during pregnancy. Even if the transition leads to a *L. iners*-dominated microbiome, which is generally considered more optimal than anaerobe-predominant microbiomes, it is a beneficial transition. Interestingly, this effect was not observed in individuals exposed to PrEP. Instead, when HIV-negative pregnant women experienced transitions in the structure of their vaginal microbiome following PrEP initiation, it was most frequently from *L. iners* to non-optimal *Gardnerella* spp. (CST IV-B) predominance, and specifically a reduction in *L. iners* mgSs 1 and 2, and an increase in *Gardnerella* sp. 12. These kinds of transitions are considered non-optimal.

Comparisons to a control sub-cohort unexposed to PrEP^11,15^ provided evidence that such transitions to non-optimal microbiome may be the result of PrEP exposure. However, these transitions may be also explained by the geographic, genetic and social differences between cohorts. Further, our analyses suggest that this may be meaningful to birth outcomes. In our small study, we observed a trend towards greater odds of sPTB when the vaginal microbiome shifted towards *Gardnerella* spp. predominance during gestation. These results emphasize the need for further investigations to understand the mechanistic impacts of PrEP exposure on the vaginal microbiome and the host during pregnancy.

At M0, prior to ARV exposure, the vaginal microbiomes of pregnant WLHIV contained higher bacterial diversity relative to HIV-negative pregnant women at M0. Moreover, we found that HIV-negative women were more likely to harbor *L. iners*-dominant CST III as well as CST I comprising *L. crispatus* mgSs 4, while CST IV-B was more frequently observed among WLHIV, confirming previously reported associations between the vaginal microbiome composition and HIV infection^16–18^. Studies in non-pregnant women in sub-Saharan Africa have broadly linked diverse anaerobe-predominant vaginal microbiomes to increased HIV viral shedding among WLHIV and a higher risk of HIV acquisition in uninfected individuals^18–21^. Conversely, *L. crispatus* has been associated with a protective effect against HIV acquisition^22^.

Currently, there is a significant lack of data concerning the impact of antiretroviral drugs, whether used for HIV treatment or prevention, on the vaginal microbiome of healthy women in sub-Saharan Africa^13^. This study contributes to the limited body of literature available that describes the vaginal microbiome among African women during pregnancy^16,18,23,24^. In addition to the longitudinal characterization of the vaginal microbiome during pregnancy in the context of antiretroviral drug initiation for both HIV treatment and prevention among pregnant women, a major strength of this study lies in the use of higher-resolution characterization of the vaginal microbiome through metagenomic analyses that distinguish mgSs of *L. iners* and *Gardnerella* genomospecies^14,15,25^. This approach provided important information on how these medications impact the vaginal microenvironment during pregnancy in sub-Saharan Africa and, ultimately, how this may influence pregnancy outcomes.

The findings from our study should be interpreted with caution, as the study design did not assess causality. Limitations of the study included a relatively small sample size, infrequent sampling, missing outcome data, and differences in gestational age at M0 between the cohorts. Furthermore, significant baseline differences were observed between women with HIV and women without HIV, which are known to influence the vaginal microbiome and the risk of preterm birth. Notably, women without HIV had significantly higher rates of syphilis, vaginal discharge, and genital ulcers at baseline compared to women with HIV.

We also lacked data on other sexually transmitted infections such as gonorrhea, chlamydia, herpes simplex virus (HSV), and trichomonas, as well as information on sexual and behavioral practices, including douching. These factors could have influenced both baseline and longitudinal measures of the microbiome and the risk of preterm birth. Another limitation of the study is the geographic, genetic, and social variation between the TP2 and ZAPPS cohorts. Additionally, the TP2 cohort did not include HIV-control participants who were not on PrEP and though there are significant limitations in this analysis, we did not find the same microbiome changes in the Zambian cohort that was not on PrEP. It is also important to note that spontaneous preterm birth is relatively rare, meaning that many women with “non-optimal” vaginal microbiomes still deliver healthy, term babies, as demonstrated in this study. Our study provides important insights into the vaginal microbiome before and after exposure to antiretroviral drugs during pregnancy. Distinct CST transitions were observed between HIV-negative pregnant women who initiated HIV prophylaxis and pregnant WLHIV who initiated treatment. The study highlights the importance of the vaginal microbiome in HIV infection and pregnancy in sub-Saharan Africa. Future research with larger and frequently sampled cohorts, and long-term follow-up will be crucial for understanding antiretroviral drugs’ impact on the vaginal microbiome, its function, and maternal and infant health outcomes.

## Methods

### Setting and population

The *Tonse Pamodzi 2* (TP2) study was a pilot randomized controlled trial that evaluated a combination intervention to support antiretroviral drug regimen adherence among pregnant WLHIV initiating antiretroviral therapy (ART) and among HIV-negative pregnant women initiating daily oral pre-exposure prophylaxis (PrEP). A description of the study protocol has been published previously^26^. Briefly, the TP2 study was a randomized trial of adherence support intervention for ART or PrEP among pregnant women. The study enrolled 100 pregnant WLHIV initiating once-daily ART (tenofovir disoproxil fumarate, lamivudine, and dolutegravir) and 200 pregnant HIV-negative women initiating daily oral PrEP, comprised of co-formulated tenofovir disoproxil fumarate and emtricitabine. The TP2 study was conducted at Bwaila District Hospital, a high volume public hospital located in Lilongwe, Malawi, that offers maternity care services to approximately 18,000 women each year. Study eligibility details were previously published by Saidi et al.^26^. Participants were eligible if they were 18+, had a confirmed pregnancy by test or ultrasound, no history of intimate partner violence, and had initiated first-line ART or PrEP. Delivery gestational age was based on LMP or ultrasound^27^.

### Specimen collection

Trained research nurses collected samples from all participants at enrollment (Month 0, M0) and four to six weeks after enrollment (Month 1, M1) using COPAN Eswabs. Collection involved inserting a swab into the vagina and gently rotating it three times along the vaginal wall, without a speculum. The swabs were placed into individual cryovials with 1 mL nucleic acid stabilizing solution (ZYMO DNA/RNA Shield) and frozen to −80 °C within four hours. All samples were shipped from the UNC Project-Malawi Laboratory to the Institute for Genome Sciences at the University of Maryland for DNA extraction, metagenomic sequencing, and data analysis.

### DNA isolation and metagenomic sequencing

DNA was extracted from 200 µl swab eluates using the MagAttract Power Microbiome DNA/RNA Kit (Qiagen) and bead-beating on a TissueLyser II according to the manufacturer’s instructions and automated on a Hamilton STAR robotic platform. Metagenomic sequencing libraries were constructed using the Illumina Nextera XT DNA Library Preparation kit and unique dual indices according to the manufacturer’s recommendations. The libraries were sequenced on an Illumina NovaSeq 6000 S4 Flowcell. Positive (ZymoBIOMICS Microbial Community Standard, Zymo Research, Irvine, CA) and negative controls (water) were run alongside samples. An average of 41 million [7–120 million] 150 bp sequence reads per sample were obtained before processing.

### Metagenomic sequence reads data processing and bioinformatics

Host reads were removed from all metagenomic sequence data using BMTagger and the GRCh38 reference genome^28^. Ribosomal RNA was removed using SortMeRNA (v.2.1b) with the silva-bac-16s-id90 reference database and the paired_in option^29^. Low quality reads were removed using fastqp (v0.21.0), cut window size 4 bp, cut mean quality Q20, minimum read length: 50 bp^30^. A mean 4·1 million [0.11–69 million] sequence reads per sample survived filtering. Remaining metagenomic sequence reads were mapped to the VIRGO gene catalogue^31^ using bowtie (v1; parameters: -p 16 -l 25 --fullref --chunkmbs 512 --best --strata -m 20 --suppress 2,4,5,6,7,8), producing a taxonomic and gene annotation for each read. The number of reads mapped to a gene was multiplied by the read length (150 bp) and divided by the gene length to produce a coverage value for each gene as described by Ma et al.^31^. CST assignments were performed by processing the taxonomic composition tables generated using VIRGO through the vaginal CST classifier VALENCIA^25^. Gene abundance and taxonomic composition tables were processed through the metagenomic CST (mgCST) classifier^14^, producing metagenomic subspecies (mgSs) proportions for every sample. MgSs found with more than 300 sequences in negative controls were removed from downstream analyses and included *Escherichia coli, Prevotella timonensis 0, Propionibacterium sp., Salmonella enterica, Sneathia amnii 0*, and *Staphylococcus epidermidis*. The proportion of *Gardnerella* genomospecies in each sample was estimated by first mapping VIRGO genes to existing genomospecies genomes^32^, and calculating the proportion of all *Gardnerella* reads attributed to each genomospecies. When a genomospecies could not be determined, it was called *G. vaginalis*.

### Statistical analysis

All statistical analyses were performed and figures generated using R (v 4.2.2)^33^, and ggplot2 (v3.4.2)^34^.

#### Covariates

All covariates other than gestational age (GA) and syphilis test results were self-reported on the behavioral survey administered at study enrollment. Unless otherwise stated, adjusted analyses accounted for participant age, estimated GA, results from the most recent syphilis test conducted during the index pregnancy, gravidity, abnormal vaginal discharge in the past three months, genital ulcers observed in the past three months, primary partner HIV serostatus, and if the participant’s home had running water. All adjusted analyses were restricted to participants with no missing data.

#### Alpha diversity, HIV serostatus, and ART or PrEP initiation

The Shannon diversity index was determined for each swab using the estimate_richness function of the phyloseq package (v 1.42.0)^35^. We compared Shannon diversity between HIV serostatus at M0 (WLHIV or HIV-negative) using Wilcoxon rank sum tests (unadjusted). Additionally, we compared Shannon diversity between M0 and M1 within each HIV serostatus using the mean within-woman difference via ANOVA.

#### CSTs, HIV serostatus, and ART or PrEP initiation

Generalized logistic regression was used to estimate the odds of CST assignment at M0 by HIV serostatus and adjusted for covariates: the predictor was HIV serostatus and the outcome was CST I/III or CST IV-B (CST IV-C was excluded). For participants that contributed both M0 and M1 swabs, generalized logistic regression was used to estimate the odds of CST transition (binary) given HIV serostatus/ARV type. Here, the model accounted for days between M0 and M1 in addition to other covariates. Among those that did change CST, transition types were described (for example, transition from CST III at M0 to CST IV at M1 was defined as “III → IV”).

#### MgSs, HIV serostatus, and ART or PrEP initiation

To identify associations between mgSs and HIV serostatus at M0, the abundance data of mgSs present in at least 10% of samples were normalized for differences in coverage using the “poscounts” estimator in the R package DESeq2 which also addresses many zeroes^36^. Mixed effect linear regression (limma version 3.58.1^37^) was used to model the relationship between mgSs and HIV serostatus at M0 and associations were tested for significance using the eBayes test^38^. Models assessing associations with HIV serostatus at M0 accounted for all covariates. To evaluate associations with ARV initiation, data were first stratified by HIV serostatus, and then models were fit to evaluate the relationships of mgSs at M1 compared to M0. In addition to other covariates, models accounted for the days between M0 and M1 and participant ID. All p-values were adjusted for multiple testing with Bonferroni-Holm correction with a false discovery rate threshold of 5%, and log_2_ differences in mgSs relative abundances are reported.

#### Transition to CST IV and PrEP exposure

In this study, microbiome transitions to CST IV were only observed among HIV-negative women initiating PrEP and no HIV-negative participants were unexposed to PrEP. Hence, in order to gauge the correlation between PrEP exposure and the progression to CST IV during pregnancy, we utilized an autonomous dataset sourced from Lusaka, Zambia, referred to as ZAPPS (Zambian Preterm Birth Prevention Study, *N* = 1450 participants), wherein vaginal microbiome data had already been compiled and published^11,15,16^. From these data, we selected microbiomes from pregnant, HIV-negative participants unexposed to PrEP with less than 41 days between clinical visits as a comparator sub-cohort (*N* = 196). Employing both the PrEP-unexposed ZAPPS sub-cohort and the PrEP-exposed TP2 sub-cohort, we conducted generalized logistic regression analysis to assess the relationship between transitioning from non-CST IV at M0 to CST IV at M1 and PrEP exposure and accounted for participant age, gestational age, gravidity, access to drinking water, and syphilis test result at M0.

#### HIV serostatus, the vaginal microbiome, and sPTB

We defined PTB as delivery before the 37th week of pregnancy^19,20^. The PTB classification was further refined into two categories: sPTB (*i.e*., parturition was initiated without any intervention from a healthcare provider) and provider-initiated (*i.e*., parturition was initiated due to an intervention by a healthcare provider). Among all TP2 participants in this secondary analysis, the association between HIV serostatus and pregnancy outcome (term or sPTB) was evaluated using generalized logistic regression. We also assessed the association between having CST IV at any point during pregnancy (M0 or M1) and pregnancy outcome. For participants that contributed swabs at both M0 and M1, we evaluated separately if an association existed with any change in CST (versus no change in CST) and a change to CST IV at M1 from a non-CST IV at M0 (versus no change or any other transition). The latter was only evaluated among HIV-negative women initiating PrEP because transitions to CST IV were only observed in these participants. Each model accounted for all covariates as stated in *Covariates* with the exception of primary partner HIV serostatus.

### Ethics

Ethical approval for Tonse Pamodzi 2 study was obtained for the Malawi National Health Sciences Research Committee (19/05/2334) and the University of North Carolina at Chapel Hill Institutional Review Board (19-1060). Written informed consent was obtained from each participants prior to Tonse Pamodzi 2 study enrollment and data collection. The study was registered in clinicaltrials.gov (NCT04330989) before enrollment commenced. The ZAPPS study received approval from the University of Zambia School of Medicine (016-04-14) and the University of North Carolina School of Medicine (14-2113).

## Supplementary information

Supplementary Material

Supplementary Code 1

## Data Availability

Sequence data that support the findings of this study have been deposited in the NIH SRA and the BioProject ID is PRJNA1179925.

## Code availability

All analyses are available in Supplementary Code 1.
