# Supplementary material for: Effect of initiation of antiretroviral drugs for HIV prevention or treatment on the vaginal microbiome of pregnant women in Malawi
Source: NPJ Biofilms Microbiomes. 2025 Apr 26;11:67. doi: 10.1038/s41522-025-00697-8 (PMC12033299; doi:10.1038/s41522-025-00697-8)
Supplement: Supplementary file 2 — Supplementary Code 1 [file 41522_2025_697_MOESM2_ESM.zip › Supplementary 1.html]

MLAWI 2023


Code 

- Show All Code
- Hide All Code
- Download Rmd

# MLAWI 2023

This notebook captures the analysis of the MLAWI project as outlined
in Feb\_08\_email/Saidi\_Microbiome Draft.docx by Friday Saidi.

24Mar2023: - Switch around the exposure and the outcome (the exposure
is HIV status and the outcome is CST). - Re-explore the covariates
included to only look at those associated with CST (outcome), not HIV. -
Update the models to reflect the correct adjustments.


```
setwd("~/Dropbox (IGS)/MSL/MLAWI/")
```


# Date and colors

## Colors


```
CST.col<-as.data.frame(rbind(c("I", "#FE0308"),c("I-A", "#FE0308"), c("I-B", "#F6D3DA"), c("II", "#86C61A"),c("III", "#FF7200"),c("III-A", "#FF7200"),c("III-B", "#F8A40E"), c("IV", "#221886"),c("IV-A", "#448A73"), c("IV-B", "#221886"), c("IV-C", "#C0ACD3"),c("IV-C0", "#989898"),c("IV-C1", "#EF53A7"),c("IV-C2", "#A7DDDC"),c("IV-C3", "#98C999"),c("IV-C4", "#7F0B7C"), c("V", "#FAE50D"), c("", "white"), c("NA", "white")))
names(CST.col)<-c("CST", "color")

mgCST.col<-as.data.frame(rbind(c("1", "#FE0308"),c("2", "#F54C5E"), c("3", "#F07084"), c("4", "#EC94A5"),c("5", "#F0BCCC"),c("6", "#F6D3DA"),c("7", "#86C61A"), c("8", "#B4DB29"),c("9", "#DBEA77"), c("10", "#FF7200"), c("11", "#F68A11"),c("12", "#F8A40E"),c("13", "#F3BC11"),c("14", "#f7d15a"), c("15", "#FAE50D"),c("16", "#F3F46E"),c("17", "#448A73"),c("18", "#89BEAB"), c("19", "#BCD6CD"),c("20", "#221886"),c("21", "#3E3792"),c("22", "#5D579E"),c("23", "#7C76AC"),c("24", "#9A98BF"),c("25", "#C9C8D8"),c("26", "#98C999"), c("27", "#989898"), c("", "white"), c("NA", "white")))
names(mgCST.col)<-c("mgCST", "color")
```


```
mgss.colors<-as.data.frame(rbind(c("Lactobacillus_crispatus_1", "#FE0308"),c("Lactobacillus_crispatus_2", "#F54C5E"), c("Lactobacillus_crispatus_3", "#F07084"), c("Lactobacillus_crispatus_4", "#EC94A5"),c("Lactobacillus_crispatus_5", "#F0BCCC"),c("Lactobacillus_crispatus_6", "#F6D3DA"),c("Lactobacillus_gasseri_1", "#86C61A"), c("Lactobacillus_gasseri_2", "#B4DB29"),c("Lactobacillus_gasseri_3", "#DBEA77"), c("Lactobacillus_iners_1", "#FF7200"), c("Lactobacillus_iners_2", "#F68A11"),c("Lactobacillus_iners_3", "#F8A40E"),c("Lactobacillus_iners_5", "#F3BC11"),c("Lactobacillus_iners_6", "#f7d15a"),c("Lactobacillus_iners_4", "#f7d16a"), c("Lactobacillus_jensenii_1", "#FAE50D"),c("Lactobacillus_jensenii_2", "#F3F46E"),c("Ca.Lachnocurva_vaginae_1", "#448A73"),c("Ca.Lachnocurva_vaginae_2", "#89BEAB"), c("Ca.Lachnocurva_vaginae_3", "#BCD6CD"),c("Ca.Lachnocurva_vaginae_4", "#BCD6CF"), c("Ca.Lachnocurva_vaginae_5", "#BCD6DC"), c("Gardnerella_vaginalis_0", "#221990"), c("Gardnerella_vaginalis_1", "#221886"),c("Gardnerella_vaginalis_2", "#3E3792"),c("Gardnerella_vaginalis_3", "#5D579E"),c("Gardnerella_vaginalis_4", "#7C76AC"),c("Gardnerella_vaginalis_5", "#9A98BF"),c("Gardnerella_vaginalis_6", "#C9C8D8"),c("Bifidobacterium*", "#98C999"), c("Staphylococcus*", "#989898"), c("", "white"), c("NA", "white"), c("Enterococcus_faecalis","#bbffff"),c("Raoultella_planticola","#ffc2ff"),c("g_Peptoniphilus","#CCCC00"),c("Sneathia_sanguinegens","#c7aa8f"),c("Atopobium_vaginae_1","#0000cd"),c("Atopobium_vaginae_2","#0000cd"),c("g_Atopobium","#0000cd"),c("Lacotbacillus_helveticus","#00ccff"),c("Mageeibacillus_indolicus","#3cb371"),c("g_Anaerococcus","#87cefa"),c("g_Gardnerella","#20b2aa"),c("Megasphaera_sp_type_1","#0000ff"),c("Streptococcus_agalactiae","#ff738a"),c("g_Megasphaera","#008B45"),c("Megasphaera_genomosp.","#008B45"),c("Streptococcus_oralis","#ff66ff"),c("Prevotella_bivia_1","#bfbfbf"),c("Prevotella_bivia_1","#bfbfbf"),c("Prevotella_bivia_3","#bfbfbf"), c("Aerococcus_christensenii","#bebebe"),c("Anaerococcus_tetradius","#87cefa"),c("Gemella","#daa520"),c("Prevotella_genogroup_1","#b0b0b0"),c("Prevotella_buccalis","#b0b0b0"),c("Lactobacillus_vaginalis","#ffffff"),c("Other","black"),c("Bifidobacterium_longum","#c1ffc1"),c("Bifidobacterium_breve","#c1ffc1"),c("Eggerthella","#DE7710"),c("Mycoplasma_hominis","#10DE4E"),c("Porphyromonas_bennonis","#DE4310"),c("Eubacterium_saphenum","#8F10DE"),c("Fusobacterium_nucleatum","#CD853F"),c("Fusobacterium_gonidiaformans","#CD853F"),c("Streptococcus_anginosus","#ffc0cb"),c("Peptostreptococcus_anaerobius","#DEDB10"),c("Arcanobacterium_phocae","#8c10de"),c("Bacteroides_uniformis","#de1058"),c("Ureaplasma_parvum","#9999ff"),c("Peptoniphilus_harei","#CCCC00"),c("Mobiluncus_mulieris","#f08080"),c("Megasphaera_sp._type_2","#008B45"),c("Mageeibacillus_indolicus","#3bb16f"),c("g_Escherichia.Shigella","#12456b"),c("Peptoniphilus_lacrimalis","#d2b48c"),c("Veillonella_montpellierensis","#ff8c69"),c("Prevotella_genogroup_3","#b36200"),c("Prevotella_disiens","#b36200"),c("Parvimonas_micra","#cdcd00"),c("Corynebacterium_accolens","#ffff00"),c("Finegoldia_magna","#800080"),c("Prevotella_genogroup_2","#b0b0b0"),c("Staphylococcus_epidermidis","#ffffff"),c("Prevotella_timonensis","#f8fca9"),c("g_Streptococcus","#ffc0cb"),c("g_Bifidobacterium","#c1ffc1"),c("g_Enterococcus","#bbffff"),c("g_Staphylococcus","#800080"),c("g_Finegoldia","#800080"),c("g_Prevotella","#bfbfbf"),c("g_Sneathia","#d1e8eb"),c("g_Aerococcus","#e8e1ba"),c("g_Leptotrichia","#7ca386"),c("g_Veillonella","#499996"),c("g_Dialister","#997dbd"),c("g_Corynebacterium_1","#ffff00"),c("g_Varibaculum","#094717"),c("g_Delftia","#090c47"),c("g_Corynebacterium_1","#ffff00"),c("Prevotella_amnii_1","#ac8fc7"),c("Prevotella_amnii_6","#ac8fc7"),c("Sneathia_amnii","#6963ff"), c("Propionibacterium_sp.","#bfbfbd")))
names(mgss.colors)<-c("taxa", "color")

taxa.colors<-as.data.frame(rbind(c("Lactobacillus_crispatus","#ff0000"),c("Gardnerella_vaginalis","#20b2aa"),c("g_Lactobacillus","#eef06c"),c("Lactobacillus_iners","#ff8c00"),c("Lactobacillus_gasseri","#7fff00"),c("Lactobacillus_jensenii","#333333"),c("Enterococcus_faecalis","#bbffff"),c("Raoultella_planticola","#ffc2ff"),c("g_Peptoniphilus","#CCCC00"),c("Sneathia_sanguinegens","#c7aa8f"),c("Atopobium_vaginae","#0000cd"),c("g_Atopobium","#0000cd"),c("Lacotbacillus_helveticus","#00ccff"),c("Mageeibacillus_indolicus","#3cb371"),c("g_Anaerococcus","#87cefa"),c("g_Gardnerella","#20b2aa"),c("Megasphaera_sp_type_1","#0000ff"),c("Streptococcus_agalactiae","#ff738a"),c("g_Megasphaera","#008B45"),c("Megasphaera_genomosp.","#008B45"),c("Streptococcus_oralis","#ff66ff"),c("Prevotella_bivia","#bfbfbf"),c("Aerococcus_christensenii","#bebebe"),c("Anaerococcus_tetradius","#87cefa"),c("Gemella","#daa520"),c("Prevotella_genogroup_1","#b0b0b0"),c("Prevotella_buccalis","#b0b0b0"),c("Lactobacillus_vaginalis","#ffffff"),c("other","#808080"),c("Bifidobacterium_longum","#c1ffc1"),c("Bifidobacterium_breve","#c1ffc1"),c("Eggerthella","#DE7710"),c("Mycoplasma_hominis","#10DE4E"),c("Porphyromonas_bennonis","#DE4310"),c("Eubacterium_saphenum","#8F10DE"),c("Fusobacterium_nucleatum","#CD853F"),c("Fusobacterium_gonidiaformans","#CD853F"),c("Streptococcus_anginosus","#ffc0cb"),c("Peptostreptococcus_anaerobius","#DEDB10"),c("Arcanobacterium_phocae","#8c10de"),c("Bacteroides_uniformis","#de1058"),c("Ureaplasma_parvum","#9999ff"),c("Peptoniphilus_harei","#CCCC00"),c("Mobiluncus_mulieris","#f08080"),c("Megasphaera_sp._type_2","#008B45"),c("Ca_LachncurvaVaginae","#b31900"),c("g_Escherichia.Shigella","#12456b"),c("Peptoniphilus_lacrimalis","#d2b48c"),c("Veillonella_montpellierensis","#ff8c69"),c("Prevotella_genogroup_3","#b36200"),c("Prevotella_disiens","#b36200"),c("Parvimonas_micra","#cdcd00"),c("Corynebacterium_accolens","#ffff00"),c("Finegoldia_magna","#800080"),c("Prevotella_genogroup_2","#b0b0b0"),c("Staphylococcus_epidermidis","#ffffff"),c("Prevotella_timonensis","#f8fca9"),c("g_Streptococcus","#ffc0cb"),c("g_Bifidobacterium","#c1ffc1"),c("g_Enterococcus","#bbffff"),c("g_Staphylococcus","#800080"),c("g_Finegoldia","#800080"),c("g_Prevotella","#bfbfbf"),c("g_Sneathia","#d1e8eb"),c("g_Aerococcus","#e8e1ba"),c("g_Leptotrichia","#7ca386"),c("g_Veillonella","#499996"),c("g_Dialister","#997dbd"),c("g_Corynebacterium_1","#ffff00"),c("g_Varibaculum","#094717"),c("g_Delftia","#090c47"),c("Prevotella_amnii","#ac8fc7"),c("Sneathia_amnii","#6963ff")))
names(taxa.colors)<-c("taxa", "color")


hiv.cols<-c("gold", "darkorchid4")
treat.cols<-c("#B8DE29FF", "#33638DFF")
```

## Date


```
today <- strsplit(date(), " ")
month <- today[[1]][2]
if(today[[1]][3] %in% ""){
    day <- today[[1]][4]
    year <- today[[1]][6]
    }else{
    day <- today[[1]][3]
    year <- today[[1]][5]
    }
today2 <- paste(day,month,year, sep="")
```

## Packages


```
require(broom)
```


```
Loading required package: broom
```


```
require(ggplot2)
```


```
Loading required package: ggplot2
```


```
require(dplyr)
```


```
Loading required package: dplyr

Attaching package: ‘dplyr’

The following objects are masked from ‘package:stats’:

    filter, lag

The following objects are masked from ‘package:base’:

    intersect, setdiff, setequal, union
```


```
require(reshape2)
```


```
Loading required package: reshape2
```


```
require(EnvStats)
```


```
Loading required package: EnvStats

Attaching package: ‘EnvStats’

The following objects are masked from ‘package:stats’:

    predict, predict.lm
```


```
require(readxl)
```


```
Loading required package: readxl
```


```
require(vegan)
```


```
Loading required package: vegan
Loading required package: permute
Loading required package: lattice
This is vegan 2.6-6.1

Attaching package: ‘vegan’

The following object is masked from ‘package:EnvStats’:

    calibrate
```


```
require(stringr)
```


```
Loading required package: stringr
```


```
require(ggpubr)
```


```
Loading required package: ggpubr
```


```
require(ggstatsplot)
```


```
Loading required package: ggstatsplot
You can cite this package as:
     Patil, I. (2021). Visualizations with statistical details: The 'ggstatsplot' approach.
     Journal of Open Source Software, 6(61), 3167, doi:10.21105/joss.03167
```


```
require(DESeq2)
```


```
Loading required package: DESeq2
Loading required package: S4Vectors
Loading required package: stats4
Loading required package: BiocGenerics

Attaching package: ‘BiocGenerics’

The following objects are masked from ‘package:dplyr’:

    combine, intersect, setdiff, union

The following objects are masked from ‘package:stats’:

    IQR, mad, sd, var, xtabs

The following objects are masked from ‘package:base’:

    anyDuplicated, aperm, append, as.data.frame, basename, cbind, colnames,
    dirname, do.call, duplicated, eval, evalq, Filter, Find, get, grep, grepl,
    intersect, is.unsorted, lapply, Map, mapply, match, mget, order, paste,
    pmax, pmax.int, pmin, pmin.int, Position, rank, rbind, Reduce, rownames,
    sapply, setdiff, table, tapply, union, unique, unsplit, which.max,
    which.min


Attaching package: ‘S4Vectors’

The following objects are masked from ‘package:dplyr’:

    first, rename

The following object is masked from ‘package:utils’:

    findMatches

The following objects are masked from ‘package:base’:

    expand.grid, I, unname

Loading required package: IRanges

Attaching package: ‘IRanges’

The following objects are masked from ‘package:dplyr’:

    collapse, desc, slice

Loading required package: GenomicRanges
Loading required package: GenomeInfoDb
Loading required package: SummarizedExperiment
Loading required package: MatrixGenerics
Loading required package: matrixStats

Attaching package: ‘matrixStats’

The following object is masked from ‘package:EnvStats’:

    iqr

The following object is masked from ‘package:dplyr’:

    count


Attaching package: ‘MatrixGenerics’

The following objects are masked from ‘package:matrixStats’:

    colAlls, colAnyNAs, colAnys, colAvgsPerRowSet, colCollapse, colCounts,
    colCummaxs, colCummins, colCumprods, colCumsums, colDiffs, colIQRDiffs,
    colIQRs, colLogSumExps, colMadDiffs, colMads, colMaxs, colMeans2,
    colMedians, colMins, colOrderStats, colProds, colQuantiles, colRanges,
    colRanks, colSdDiffs, colSds, colSums2, colTabulates, colVarDiffs,
    colVars, colWeightedMads, colWeightedMeans, colWeightedMedians,
    colWeightedSds, colWeightedVars, rowAlls, rowAnyNAs, rowAnys,
    rowAvgsPerColSet, rowCollapse, rowCounts, rowCummaxs, rowCummins,
    rowCumprods, rowCumsums, rowDiffs, rowIQRDiffs, rowIQRs, rowLogSumExps,
    rowMadDiffs, rowMads, rowMaxs, rowMeans2, rowMedians, rowMins,
    rowOrderStats, rowProds, rowQuantiles, rowRanges, rowRanks, rowSdDiffs,
    rowSds, rowSums2, rowTabulates, rowVarDiffs, rowVars, rowWeightedMads,
    rowWeightedMeans, rowWeightedMedians, rowWeightedSds, rowWeightedVars

Loading required package: Biobase
Welcome to Bioconductor

    Vignettes contain introductory material; view with 'browseVignettes()'. To
    cite Bioconductor, see 'citation("Biobase")', and for packages
    'citation("pkgname")'.


Attaching package: ‘Biobase’

The following object is masked from ‘package:MatrixGenerics’:

    rowMedians

The following objects are masked from ‘package:matrixStats’:

    anyMissing, rowMedians
```


```
require(ggrepel)
```


```
Loading required package: ggrepel
```


```
require(lmerTest)
```


```
Loading required package: lmerTest
Loading required package: lme4
Loading required package: Matrix

Attaching package: ‘Matrix’

The following object is masked from ‘package:S4Vectors’:

    expand


Attaching package: ‘lmerTest’

The following object is masked from ‘package:lme4’:

    lmer

The following object is masked from ‘package:stats’:

    step
```


```
require(lme4)
require(tidyverse)
```


```
Loading required package: tidyverse
```


```
── Attaching core tidyverse packages ──────────────────────────────── tidyverse 2.0.0 ──
✔ forcats   1.0.0     ✔ readr     2.1.5
✔ lubridate 1.9.3     ✔ tibble    3.2.1
✔ purrr     1.0.2     ✔ tidyr     1.3.1
── Conflicts ────────────────────────────────────────────────── tidyverse_conflicts() ──
✖ lubridate::%within%()    masks IRanges::%within%()
✖ IRanges::collapse()      masks dplyr::collapse()
✖ Biobase::combine()       masks BiocGenerics::combine(), dplyr::combine()
✖ matrixStats::count()     masks dplyr::count()
✖ IRanges::desc()          masks dplyr::desc()
✖ tidyr::expand()          masks Matrix::expand(), S4Vectors::expand()
✖ dplyr::filter()          masks stats::filter()
✖ S4Vectors::first()       masks dplyr::first()
✖ dplyr::lag()             masks stats::lag()
✖ tidyr::pack()            masks Matrix::pack()
✖ BiocGenerics::Position() masks ggplot2::Position(), base::Position()
✖ purrr::reduce()          masks GenomicRanges::reduce(), IRanges::reduce()
✖ S4Vectors::rename()      masks dplyr::rename()
✖ lubridate::second()      masks S4Vectors::second()
✖ lubridate::second<-()    masks S4Vectors::second<-()
✖ IRanges::slice()         masks dplyr::slice()
✖ tidyr::unpack()          masks Matrix::unpack()
ℹ Use the ]8;;http://conflicted.r-lib.org/conflicted package]8;; to force all conflicts to become errors
```


```
require(phyloseq)
```


```
Loading required package: phyloseq
Registered S3 method overwritten by 'data.table':
  method           from
  print.data.table     

Attaching package: ‘phyloseq’

The following object is masked from ‘package:SummarizedExperiment’:

    distance

The following object is masked from ‘package:Biobase’:

    sampleNames

The following object is masked from ‘package:GenomicRanges’:

    distance

The following object is masked from ‘package:IRanges’:

    distance
```


```
library(edgeR)
```


```
Loading required package: limma

Attaching package: ‘limma’

The following object is masked from ‘package:DESeq2’:

    plotMA

The following object is masked from ‘package:BiocGenerics’:

    plotMA
```


```
require(table1)
```


```
Loading required package: table1

Attaching package: ‘table1’

The following objects are masked from ‘package:base’:

    units, units<-
```


```
require(nlme)
```


```
Loading required package: nlme

Attaching package: ‘nlme’

The following object is masked from ‘package:lme4’:

    lmList

The following object is masked from ‘package:IRanges’:

    collapse

The following object is masked from ‘package:dplyr’:

    collapse
```


```
require(epitools)
```


```
Loading required package: epitools
```


```
require(ggpmisc)
```


```
Loading required package: ggpmisc
Loading required package: ggpp
Registered S3 methods overwritten by 'ggpp':
  method                  from   
  heightDetails.titleGrob ggplot2
  widthDetails.titleGrob  ggplot2

Attaching package: ‘ggpp’

The following objects are masked from ‘package:ggpubr’:

    as_npc, as_npcx, as_npcy

The following object is masked from ‘package:ggplot2’:

    annotate
```


```
require(pheatmap)
```


```
Loading required package: pheatmap
```


```
pvalue <- function(x, ...) {
  # Construct vectors of data y, and groups (strata) g
  y <- unlist(x)
  g <- factor(rep(1:length(x), times=sapply(x, length)))
  if (is.numeric(y)) {
    # For numeric variables, perform a standard 2-sample t-test
    p <- round(t.test(y ~ g)$p.value, 2)
  } else {
    # For categorical variables, perform a chi-squared test of independence
    p <- round(chisq.test(table(y, g))$p.value, 2)
  }
  # Format the p-value, using an HTML entity for the less-than sign.
  # The initial empty string places the output on the line below the variable label.
  c("", sub("<", "&lt;", format.pval(p, digits=2, eps=0.01)))
}
```

# Load Data


```
#load("MLAWI.RData")
```

# Metadata

## Aim 1

Aim 1: To compare the vaginal microbiota composition and structure,
between HIV+ and HIV- pregnant women, prior to initiation of either ART
(for HIV+ pregnant women) or PrEP (for HIV- pregnant women). OR
pre-treatment (ART or PrEP) microbiome HIV +/-

AIM 1 Analysis plan: We will quantify key structure and functional
characteristics of the vaginal microbiota at baseline (prior to exposure
to ARVs) using 1. mean relative abundance of prevalent bacterial taxa
(e.g., L. crispatus, L. iners, G. vaginalis), and 2. metagenomic
clusters; 3. species diversity (Shannon diversity index); and 4. species
richness (Chao richness estimator).

The taxonomic composition of the vaginal microbiota will be
established by mapping the metagenomic reads to VIRGO, a comprehensive
gene catalogue of the vaginal microbiome. The patterns of gene content
in each sample will be subjected to hierarchical clustering using
Bray-Curtis dissimilarity and Ward linkage to establish metagenomic
clusters. Because of VIRGO extensive functional and taxonomic
annotations, these clusters are defined by both composition and
function.

Associations between vaginal microbiota composition (taxonomy) and
structure (metagenomic clusters) and HIV serostatus (HIV+ vs HIV-) and
microbiome characteristics will be tested using a rank-sum test and
linear regression will be used in adjusted analyses.

Figure 2: Comparison of the mean relative abundance by HIV status
Figure 3a: Shannon diversity indices by community state type and Figure
3b Shannon diversity indices by HIV serostatus

QUESTION: WHERE IS METADATA FOR ADJUSTMENTS, SUCH AS TIME IN
PREGNANCY, SYPHILIS INFECTION, antibiotics? – Syphilis bc high risk
group – it was part of enrollment criteria for HIV (-) to have had STI
or vaginal in prior 3 months

maternal age, parity, education and socioeconomic status, nutrition,
gestational age at the time of sample collection, HIV viral load (For
HIV+ women)?


```
a1.t1<-read_excel("Feb_08_Email/Saidi_Aim1_Trial1_AnalyticSample.xlsx")
dim(a1.t1) ## 64 x 5 --- These are HIV+
```


```
[1] 64  5
```


```
a1.t1$hiv<-1
a1.t2<-read_excel("Feb_08_Email/Saidi_Aim1_Trial2_AnalyticSample.xlsx")
dim(a1.t2) ## 191 x 3 --- HIV (-)
```


```
[1] 191   3
```


```
a1.t2$hiv<-0

a1<-merge(a1.t1, a1.t2, all=TRUE)
#255 x 6
a1$hiv<-factor(a1$hiv)
a1$hiv<-relevel(a1$hiv, ref="1")
a1$aim<-"Aim 1"
```

## Aim 2

Aim 2: To compare longitudinal changes in vaginal microbiota
composition and structure, within HIV+ women on ART (DTG/3TC/TDF) and
within HIV- women on PrEP, before and after initiation of ARV drugs.

Analysis plan: To evaluate the longitudinal changes in the microbiota
composition and structure, we will assess the relative stability of the
vaginal microbiome before and after exposure to ARVs using various
measures: 1) change in Shannon Diversity Index and an indicator variable
for increasing diversity, 2) change in species richness and an indicator
for increasing richness, 3) Jensen-Shannon divergence (JSD), and 4) an
indicator of change from Lactobacillus-dominant to
Lactobacillus-deficient metagenomic clusters. All these measures in
vaginal microbiota composition and structure will be compared within
HIV+ women on ART (DTG/3TC/TDF) and within HIV- women on PrEP before and
after exposure to ARVs using logistic regression.

Figure 4: Changes in Shannon diversity indices, species richness and
Jensen-Shannon divergence by HIV serostatus/ARV type (ART and PrEP)

Sensitivity analysis to account for cases where they did not adhere
to PREp or HIV

QUESTION: FOR HIV- WHERE IS PREP INITIATION DATA?


```
a2.t1<-read_excel("Feb_08_Email/Saidi_Aim2&3_Trial1_AnalyticSample.xlsx")
dim(a2.t1) # 49 X 9
```


```
[1] 49  9
```


```
a2.t1$hiv<-1
a2.t1a<-a2.t1[,c(1:3,5:10)]
names(a2.t1a)[3]<-"maryland_ID"
a2.t1a$swab<-1
a2.t1b<-a2.t1[,c(1:2,4:10)]
names(a2.t1b)[3]<-"maryland_ID"
a2.t1b$swab<-2
a2.t1<-rbind(a2.t1a, a2.t1b)

a2.t2<-read_excel("Feb_08_Email/Saidi_Aim2&3_Trial2_AnalyticSample.xlsx")
dim(a2.t2) # 132 x 7
```


```
[1] 132   7
```


```
a2.t2$hiv<-0
a2.t2a<-a2.t2[,c(1:3,5:8)]
names(a2.t2a)[3]<-"maryland_ID"
a2.t2a$swab<-1
a2.t2b<-a2.t2[,c(1:2,4:8)]
names(a2.t2b)[3]<-"maryland_ID"
a2.t2b$swab<-2
a2.t2<-rbind(a2.t2a, a2.t2b)

a2<-merge(a2.t1, a2.t2, all=T)

a2$hiv<-factor(a2$hiv)
a2$hiv<-relevel(a2$hiv, ref="1")

a2$aim<-"Aim 2"

all.samples<-merge(a1, a2, all=TRUE)
sample.list<-unique(all.samples$maryland_ID)
```

## Covariates


```
file.names<-list.files("Feb_10_email/", pattern="*.xlsx", full.names = TRUE)
list_all <- lapply(file.names, function(x) read_excel(x))
dictionary<-list_all[5]
list_all[5]<-NULL
file.names<-list.files("Feb_10_email/", pattern="*.xlsx", full.names = FALSE)[1:4]

a1.covariate<-merge(list_all[1],list_all[2], all=TRUE)
a1<-merge(a1, a1.covariate, all=TRUE)
a2.covariate<-merge(list_all[3],list_all[4], all=TRUE)
a2<-merge(a2, a2.covariate, all=TRUE)
```

# Metagenome Data

### Sequencing QA


```
## Check reads delivered vs. quality filtered reads per sample
read.stats<-read.csv("MG_processing_stats.csv")
read.stats$sample[read.stats$sample %in% "401825_NEG"]<-"401825_ZYMO_POS"
sample_exclude<-read_excel("QC_Results.xlsx")
read.stats$exclude<-ifelse(grepl("Control", read.stats$type), as.character(read.stats$type), ifelse(read.stats$sample %in% sample_exclude$maryland_ID, as.character(sample_exclude$reason[match(read.stats$sample, sample_exclude$maryland_ID)]), NA))
## 132 samples (swabs) were excluded for reasons indicated in "exclude" column

read.stats.m<-reshape2::melt(read.stats[is.na(read.stats$exclude), as.vector(names(read.stats)[1:13])], id.vars=c("sample", "type"), variable.name = "stat_type", value.name = "reads") ## melt all columns except "exclude" (column 14)
read.stats.m$group<-ifelse(grepl("Control", read.stats.m$type), as.character(read.stats.m$type), ifelse(read.stats.m$sample %in% all.samples[all.samples$hiv == 1, "maryland_ID"], "WLHIV", ifelse(read.stats.m$sample %in% all.samples[all.samples$hiv == 0, "maryland_ID"], "HIV-Negative", NA)))
read.stats.m$pre_post<-ifelse(grepl("Control", read.stats.m$type), as.character(read.stats.m$type), ifelse(read.stats.m$sample %in% a2$maryland_ID, as.character(a2$swab[match(read.stats.m$sample, a2$maryland_ID)]), "1"))

to.plot<-read.stats.m
to.plot$group<-factor(to.plot$group, levels=c("WLHIV", "HIV-Negative"))
ggplot(to.plot[to.plot$stat_type %in% c("Reads_delivered", "host_removed", "rRNA_removed", "quality_filtered"), ], aes(x=group, y=as.numeric(reads), fill=group))+geom_boxplot(lwd=0.1, notch = T, outlier.shape = NA)+geom_jitter(width=0.1, size=0.2)+scale_y_log10()+theme_bw()+theme(text=element_text(size=8), legend.position = "none")+ylab("Total Reads")+xlab("HIV Status")+facet_wrap(~stat_type)+scale_fill_manual(values=hiv.cols)+stat_kruskal_test(size = 2, vjust = 0.8)
ggsave(paste("Analysis_Figures/Sample_coverage_by_HIV_", today2, ".tiff", sep=""), height=5, width=8)
```


```
ggplot(to.plot[to.plot$stat_type %in% c("Reads_delivered", "host_removed", "rRNA_removed", "quality_filtered"), ], aes(x=pre_post, y=as.numeric(reads), fill=pre_post))+geom_boxplot(lwd=0.1, notch = T, outlier.shape = NA)+geom_jitter(width=0.1, size=0.2)+scale_y_log10()+theme_bw()+theme(text=element_text(size=8), legend.position = "none")+ylab("Total Reads")+xlab("Swab")+facet_wrap(~stat_type)+scale_fill_manual(values=c("blue", "darkblue"))+stat_kruskal_test(size = 2, vjust = 0.8)
ggsave(paste("Analysis_Figures/Sample_coverage_by_swab_", today2, ".tiff", sep=""), height=5, width=8)
```


```
ggplot(to.plot[to.plot$stat_type %in% c("Reads_delivered", "host_removed", "rRNA_removed", "quality_filtered"), ], aes(x=stat_type, y=as.numeric(reads)))+geom_boxplot(lwd=0.1, notch = T, outlier.shape = NA)+geom_jitter(width=0.1, size=0.2)+theme_bw()+theme(text=element_text(size=8), legend.position = "none")+ylab("Total Reads")+xlab("")+stat_mean_sd_text(size = 2, vjust = 0.8)
ggsave(paste("Analysis_Figures/Sample_coverage_", today2, ".tiff", sep=""), height=5, width=8)
```


Note: There are more host-removed reads in the HIV-Neg samples. This
is likely because more of these samples were lactobacillus-predominated
communities in HIV-Neg samples which have been shown to contain
relatively more human reads than CST IV samples (Cite Ma et
al. (VIRGO))

### Controls


```
mg<-read.csv("20Mar2023/norm_counts_mgSs_mgCST_20Mar2023.csv", header=T, stringsAsFactors = F)
names(mg)[1]<-"sampleID"
n<-which(names(mg) %in% "Streptococcus_agalactiae_1")
counts<-mg[,c(2:n)]
counts$maryland_ID<-mg$sampleID
#401825_NEG was determined to actually be a positive control.
counts$maryland_ID[counts$maryland_ID %in% "401825_NEG"]<-"401825_ZYMO_POS"
rownames(counts)<-counts$maryland_ID

counts.m<-reshape2::melt(counts, id.vars="maryland_ID", variable.name = "taxa", value.name = "counts")
total.counts<-as.data.frame(counts.m %>% group_by(maryland_ID) %>% dplyr::summarise(total_counts=sum(counts)))
total.species<-as.data.frame(cbind(total_species=apply(counts, 1, function(x) sum(x >= 0.5)), maryland_ID=rownames(counts)))
counts$maryland_ID<-NULL

## POSITIVE CONTROLS (vagina + ZYMO)
pos.ctrls<-counts.m[grepl("pos", counts.m$maryland_ID, ignore.case = T), ]
top.taxa<-as.data.frame(pos.ctrls[pos.ctrls$counts > 0, ] %>% group_by(taxa) %>% dplyr::summarise(mean=mean(counts)))

top.taxa<-as.data.frame(pos.ctrls[pos.ctrls$counts > 0, ] %>% dplyr::group_by(taxa) %>% dplyr::summarise(mean=mean(counts)))
ctrl.taxa.cols<-as.data.frame(cbind(taxa=as.vector(top.taxa[top.taxa$mean > 1e4, "taxa"]), color=c(RColorBrewer::brewer.pal(12,"Set3"), RColorBrewer::brewer.pal(12,"Paired"), RColorBrewer::brewer.pal(3,"Set1"))))
```


```
Warning in cbind(...) :
  number of rows of result is not a multiple of vector length (arg 1)
```


```
top.taxa<-as.vector(top.taxa[top.taxa$mean > 1e4, "taxa"])
pos.ctrls<-pos.ctrls[pos.ctrls$taxa %in% top.taxa, ]
cols<-ctrl.taxa.cols[match(top.taxa, ctrl.taxa.cols$taxa), "color"]
ggplot(pos.ctrls, aes(x=maryland_ID, y=counts, fill=taxa))+geom_bar(stat="identity")+theme_bw()+theme(legend.position = "bottom", legend.text = element_text(size=6), legend.title = element_blank(), axis.text.x = element_text(size=5))+xlab("")+ylab("Total Corrected Reads")+scale_fill_manual(values=cols)+ggtitle("Taxa observed in positive controls with >= 1e4 reads")
ggsave(paste("Analysis_Figures/Pos_ctrls_", today2, ".tiff", sep=""), height = 5, width=7)
```


```
## compositionally similar, but the total number of reads per plate differ (coverage by plate)
as.data.frame(pos.ctrls %>% group_by(maryland_ID) %>% dplyr::summarise(sum(counts)))
```


```
## NEGATIVE CONTROLS
neg.ctrls<-counts.m[grepl("neg", counts.m$maryland_ID, ignore.case = T), ]
top.taxa<-as.data.frame(neg.ctrls[neg.ctrls$counts > 0, ] %>% group_by(taxa) %>% dplyr::summarise(mean=mean(counts), n=length(unique(maryland_ID))))
to.rm<-as.vector(top.taxa[top.taxa$n == 3 & top.taxa$mean > 300, "taxa"])

ggplot(neg.ctrls, aes(x=maryland_ID, y=counts, fill=taxa))+geom_bar(stat="identity")+theme_bw()+theme(legend.position = "none", legend.text = element_text(size=6), legend.key.size = unit(0.5,"line"), legend.title = element_blank(), axis.text.x = element_text(size=5))+xlab("")+ylab("Total Corrected Reads")+ggtitle("Taxa observed in negative controls with >= 100 reads")#+guides(fill=guide_legend(nrow=3,byrow=TRUE))
ggsave(paste("Analysis_Figures/Neg_ctrls_", today2, ".tiff", sep=""), height = 5, width=7)
```


```
as.data.frame(neg.ctrls %>% group_by(maryland_ID) %>% dplyr::summarise(sum(counts)))
```


```
## get vector of all control names to remove later.
ctrls<-as.vector(unique(c(pos.ctrls$maryland_ID, neg.ctrls$maryland_ID)))
```


Negative controls have 12-52k reads and contaminating DNA from:
“Escherichia\_coli”  
“Prevotella\_timonensis\_0”  
“Propionibacterium\_sp.”  
“Salmonella\_enterica”  
“Sneathia\_amnii\_0”  
“Staphylococcus\_epidermidis”

## Count Tables


```
## norm spp. counts w/mgSs
counts<-counts[, !names(counts) %in% to.rm] ## remove contaminants

## relabund
relabund<-counts/rowSums(counts)
relabund$maryland_ID<-rownames(counts)
relabund.m<-reshape2::melt(relabund, id.vars="maryland_ID", variable.name = "taxa", value.name = "relabund")

counts$maryland_ID<-rownames(counts)
counts.m<-reshape2::melt(counts, id.vars="maryland_ID", variable.name = "taxa", value.name = "counts")

## merge
counts.m<-merge(counts.m, relabund.m, all=TRUE)
counts.m$taxa<-gsub("\\._", "\\.", counts.m$taxa)
counts.m<-counts.m[counts.m$maryland_ID %in% sample.list, ]
counts.m<-counts.m[complete.cases(counts.m), ]
counts.m$coverage<-read.stats[match(counts.m$maryland_ID, read.stats$sample), "Reads_delivered"]

## write out for VALENCIA
to.write<-read.delim("MG processing/summary.Abundance.txt", header=T, stringsAsFactors = F, check.names = F)
names(to.write)[1]<-"sampleID"
to.write$sampleID[to.write$sampleID %in% "401825_NEG"]<-"401825_ZYMO_POS"
to.write<-to.write[!to.write$sampleID %in% unique(ctrls),!names(to.write) %in% to.rm] ## rm taxa from negative controls
to.write[,2:ncol(to.write)]<-round(to.write[,2:ncol(to.write)])
l<-as.data.frame(cbind(sampleID=to.write$sampleID, read_count=round(rowSums(to.write[,2:ncol(to.write)]))))
to.write<-merge(l, to.write, all = T)
write.csv(to.write, "20Mar2023/counts_for_VALENCIA.csv", row.names = F, quote=F)
```


### Read in Gene counts (normalized by gene length)


```
## norm gene counts
genes<-read.delim("20Mar2023/summary.NR.abundance.txt", check.names = F)
names(genes)[names(genes) %in% "401825_NEG"]<-"401825_ZYMO_POS"
rownames(genes)<-genes$sampleID
genes$sampleID<-NULL
```

### Summary Stats


```
total.samp.nGenes<-as.data.frame(cbind(total_genes=apply(genes, MARGIN = 2, function(x) sum(x>=0.5)), maryland_ID=names(genes)))
total.samp.counts<-as.data.frame(counts.m %>% group_by(maryland_ID) %>% dplyr::summarise(total_counts=sum(counts)))
total.samp.species<-as.data.frame(cbind(total_species=apply(counts, 1, function(x) sum(x >= 0.5)), maryland_ID=rownames(counts)))

sample.summary<-merge(total.samp.nGenes, total.samp.counts, all=TRUE)
sample.summary<-merge(sample.summary, total.samp.species, all=TRUE)
sample.summary<-merge(sample.summary, read.stats, all.x=TRUE, by.y="sample", by.x="maryland_ID")
```


###### Plots


```
#Does total species correspond to sequencing depth? 
ggplot(sample.summary, aes(x=log10(as.numeric(total_species)), y=log10(Reads_delivered)))+geom_point()+geom_smooth(method="lm")+stat_poly_eq() # No
```


```
`geom_smooth()` using formula = 'y ~ x'
```


```
#Does total genes correspond to sequencing depth?
ggplot(sample.summary, aes(x=log10(as.numeric(total_genes)), y=log10(Reads_delivered)))+geom_point()+geom_smooth(method="lm")+stat_poly_eq() # Slight positive, but R2 < 0.1
```


```
`geom_smooth()` using formula = 'y ~ x'
```


cd 20Mar2023

python3 ~/bin/VALENCIA/Valencia.py -ref
~/bin/speciateIT/VALENCIA2\_CST\_centroids\_20Mar2024.csv -i
counts\_for\_VALENCIA.csv -o MLAWI\_CST

Rscript ~/bin/mgCST-classifier/classify\_mgCST\_centroid.R
/Users/johannaholm/IGS Dropbox/Johanna Holm/MSL/MLAWI/20Mar2023/summary.Abundance.txt
/Users/johannaholm/IGS Dropbox/Johanna Holm/MSL/MLAWI/20Mar2023/summary.NR.abundance.txt
~/bin/VIRGO-master/ ~/bin/mgCST-classifier/

## Load and prepare sequencing, mgCST, and CST data for analyses


```
## mgCSTs
mgCSTs<-read.csv("20Mar2023/mgCSTs_26Jun2024.csv")
names(mgCSTs)[1]<-"maryland_ID"
#mgCSTs<-mgCSTs[mgCSTs$maryland_ID %in% unique(all.samples$maryland_ID), ]
mgCSTs$mgCST<-gsub("mgCST ", "", mgCSTs$mgCST)
mgCSTs$mgCST<-factor(mgCSTs$mgCST, levels=unique(sort(as.numeric(mgCSTs$mgCST))), ordered = F)

## VALENCIA csts
CSTs<-read.csv("20Mar2023/MLAWI_CST.csv")
names(CSTs)[1]<-"maryland_ID"
CSTs<-CSTs[,c("maryland_ID", "CST", "score")]
CSTs<-CSTs[CSTs$maryland_ID %in% unique(all.samples$maryland_ID), ]
CSTs$CST<-factor(CSTs$CST, levels=sort(unique(CSTs$CST)), ordered = F)
CSTs$finalReads_pe_se<-sample.summary[match(CSTs$maryland_ID, sample.summary$maryland_ID), "finalReads_pe_se"]
```


###### Update mgSs


```
## Get rid of mgSs for below: 
## few samples: 
counts.m$taxa2<-ifelse(grepl("Clostridiales_Family", counts.m$taxa), "Clostridiales_Family", as.character(counts.m$taxa))
counts.m$taxa2<-ifelse(grepl("Enterococcus_faecalis", counts.m$taxa), "Enterococcus_faecalis", as.character(counts.m$taxa2))
counts.m$taxa2<-ifelse(grepl("Lactobacillus_gasseri", counts.m$taxa), "Lactobacillus_gasseri", as.character(counts.m$taxa2))
counts.m$taxa2<-ifelse(grepl("Lactobacillus_jensenii", counts.m$taxa), "Lactobacillus_jensenii", as.character(counts.m$taxa2))
counts.m$taxa2<-ifelse(grepl("Mobiluncus_mulieris", counts.m$taxa), "Mobiluncus_mulieris", as.character(counts.m$taxa2))
counts.m$taxa2<-ifelse(grepl("Mobiluncus_curtisii", counts.m$taxa), "Mobiluncus_curtisii", as.character(counts.m$taxa2))
counts.m$taxa2<-ifelse(grepl("Mycoplasma_hominis", counts.m$taxa), "Mycoplasma_hominis", as.character(counts.m$taxa2))
counts.m$taxa2<-ifelse(grepl("Peptostreptococcus_anaerobius", counts.m$taxa), "Peptostreptococcus_anaerobius", as.character(counts.m$taxa2))
counts.m$taxa2<-ifelse(grepl("Porphyromonas_uenonis", counts.m$taxa), "Porphyromonas_uenonis", as.character(counts.m$taxa2))
counts.m$taxa2<-ifelse(grepl("Streptococcus_agalactiae", counts.m$taxa), "Streptococcus_agalactiae", as.character(counts.m$taxa2))
counts.m<-counts.m[!counts.m$taxa2 %in% to.rm, ]

counts.m$spp<-paste(str_split_fixed(counts.m$taxa2, pattern = "_", n = 3)[,1], str_split_fixed(counts.m$taxa2, pattern = "_", n = 3)[,2], sep="_")
counts.m$mgss<-str_split_fixed(counts.m$taxa2, pattern = "_", n = 3)[,3]
counts.m$mgss<-factor(counts.m$mgss, levels=c(0:10), ordered=F)

mgss<-unique(counts.m$taxa2[grepl("_[0-9]$", counts.m$taxa)])
spp<-unique(paste(str_split_fixed(mgss, pattern = "_", n = 3)[,1], str_split_fixed(mgss, pattern = "_", n = 3)[,2], sep="_"))
```

###### Update & Merge mgCSTs


```
mgCST.col<-as.data.frame(rbind(c("1", "#FE0308"),c("2", "#F54C5E"), c("3", "#F07084"), c("4", "#EC94A5"),c("5", "#F0BCCC"),c("6", "#F6D3DA"),c("7", "#86C61A"), c("8", "#B4DB29"),c("9", "#DBEA77"), c("10", "#FF7200"), c("11", "#F68A11"),c("12", "#F8A40E"),c("13", "#F3BC11"),c("14", "#f7d15a"), c("15", "#FAE50D"),c("16", "#F3F46E"),c("17", "#448A73"),c("18", "#89BEAB"), c("19", "#BCD6CD"),c("20", "#221886"),c("21", "#3E3792"),c("22", "#5D579E"),c("23", "#7C76AC"),c("24", "#9A98BF"),c("25", "#C9C8D8"),c("26", "#98C999"), c("27", "#989898"), c("", "white"), c("NA", "white")))
names(mgCST.col)<-c("mgCST", "color")

table(mgCSTs$mgCST)
```


```
  1   2   4   6  10  11  13  16  17  18  19  20  21  22  23  24  25  27 
  5   3  50   4  66  25   1   4   8   7   8  45   1  32 141  44  35  89
```


```
mgCSTs$mgCST2<-ifelse(mgCSTs$mgCST %in% 1:6, 4, as.character(mgCSTs$mgCST))
mgCSTs$mgCST2<-ifelse(mgCSTs$mgCST2 %in% 13, 27, as.character(mgCSTs$mgCST2))
mgCSTs$mgCST2<-ifelse(mgCSTs$mgCST2 %in% 21, 20, as.character(mgCSTs$mgCST2))
mgCSTs$mgCST2<-ifelse(mgCSTs$mgCST2 %in% 17:19, 17, as.character(mgCSTs$mgCST2))
l<-as.data.frame(cbind(top_taxa=colnames(counts)[apply(counts,1,which.max)], maryland_ID=rownames(counts)))
l$top_taxa<-gsub("//._", "//.", l$top_taxa)
mgCSTs<-merge(mgCSTs, l, all.x=TRUE)
mgCSTs$mgCST2<-ifelse(mgCSTs$mgCST2 %in% 27 & mgCSTs$top_taxa %in% "Lactobacillus_iners_4", 11, as.character(mgCSTs$mgCST2))
table(mgCSTs$mgCST2)
```


```
 10  11  16  17  20  22  23  24  25  27   4 
 66  80   4  23  46  32 141  44  35  35  62
```


```
## add mgCST2 to counts.m
counts.m$mgCST<-mgCSTs[match(counts.m$maryland_ID, mgCSTs$maryland_ID), "mgCST2"]
counts.m$mgCST<-factor(counts.m$mgCST, levels=unique(sort(as.numeric(counts.m$mgCST))), ordered = F)
```

##### Species


```
counts.m$spp<-paste(str_split_fixed(counts.m$taxa, pattern = "_", n = 3)[,1], str_split_fixed(counts.m$taxa, pattern = "_", n = 3)[,2], sep="_")
counts.m$mgss<-str_split_fixed(counts.m$taxa, pattern = "_", n = 3)[,3]
mgss<-unique(counts.m$taxa[grepl("_[0-9]$", counts.m$taxa)])## which species have mgSs
spp<-unique(paste(str_split_fixed(mgss, pattern = "_", n = 3)[,1], str_split_fixed(mgss, pattern = "_", n = 3)[,2], sep="_"))

spp.summary<-as.data.frame(counts.m %>% group_by(spp) %>% dplyr::summarise(meanRelabund=mean(relabund), meanCounts=mean(counts), nMgss=length(unique(mgss))))
mgss.summary<-as.data.frame(counts.m %>% group_by(spp, taxa) %>% dplyr::summarise(meanRelabund=mean(relabund), meanCounts=mean(counts)))
```


```
`summarise()` has grouped output by 'spp'. You can override using the `.groups`
argument.
```


###### FIG S1: Coverage by CST And mgSS


```
#Does the nGenes per species per sample differ by sequencing depth or relative abundance?
#If the nGenes per species differs by sequencing depth, then mgSs likely won't work, bc mgSs will be dependent on coverage. If it differs by relabund, the mgSs will work
## Determine species for which mgSs will work.
p1<-ggplot(CSTs, aes(x=finalReads_pe_se, y=score))+
  geom_point(size=0.1)+
  facet_wrap(~CST, scales="free")+
  scale_x_log10()+
  theme_bw()+
  theme(legend.position = "none", 
      text=element_text(size=1, color="black"), 
      legend.key.size = unit(0.3, "cm"), 
      line = element_line(linewidth = 0.1), 
      axis.title = element_text(size=4),
      strip.text = element_text(size=3),
      axis.text = element_text(size=4, color="black"))+
  ylab("Yue-Clayton ϴ")+
  xlab("Sequencing Depth (log10)")


p2<-ggplot(counts.m[counts.m$spp %in% spp[1:25] & counts.m$mgss %in% c(1:10) & counts.m$counts > 0, ], aes(x=reorder(x = factor(mgss), as.numeric(mgss)), y=coverage))+
  geom_boxplot(lwd=0.1, fill="gray", size=0.1, outlier.shape = NA)+
  geom_jitter(size=0.1, width=0.2)+
  facet_wrap(~spp, scales="free_x")+
  theme_bw()+
  theme(legend.position = "none", 
      text=element_text(size=1, color="black"), 
      legend.key.size = unit(0.3, "cm"), 
      line = element_line(linewidth = 0.1), 
      axis.title = element_text(size=4), 
      strip.text = element_text(size=3),
      axis.text = element_text(size=4, color="black"))+
  xlab("mgSs")+
  ylab("Reads Delivered per Sample")+
  theme(text=element_text(size=6), legend.position = "none")+
  scale_y_log10()

top_row = ggarrange(p1, p2, ncol = 2, labels = c("A.", "B."), font.label = list(face="plain", size=8), widths = c(0.75, 1))
tiff(paste("Manuscript_FIGURES/Figure_S1_", today2, ".tiff", sep=""), height = 2400, width=5600, res=600)
top_row
dev.off()
```


```
null device 
          1
```

#### Coverage & mgCSTs


```
mgcst.cols<-mgCST.col[match(sort(unique(counts.m$mgCST)), mgCST.col$mgCST), "color"]

ggplot(unique(counts.m[counts.m$counts > 0, c("maryland_ID", "coverage", "mgCST")]), aes(x=mgCST, y=coverage, fill=mgCST))+geom_violin(lwd=0.1)+geom_jitter(size=0.1, width=0.2)+theme_bw()+xlab("mgCST")+ylab("Reads Delivered per Sample")+theme(text=element_text(size=8), legend.position = "none")+scale_y_log10()+scale_fill_manual(values=mgcst.cols)
```


```
##ggsave(paste("Analysis_Figures/Sample_coverage_by_mgCST_", today2, ".tiff", sep=""), height=5, width=8)
```

# Merge MG and METADATA


```
a1.mg<-merge(a1, mgCSTs, all.x=TRUE)
a1.mg<-merge(a1.mg, CSTs, all.x=TRUE)
# OUTCOME
a1.mg$hiv<-factor(a1.mg$hiv, levels=c(1, 0), labels=c("WLHIV", "HIV-Negative"))

a2.t1<-read_excel("Feb_10_Email/Saidi_Aim2&3_Trial1_AnalyticSample.xlsx") ## ART
dim(a2.t1) # 49 X 38
```


```
[1] 49 38
```


```
a2.t1$hiv<-1
a2.t1a<-a2.t1[,c(1:3,5:6, 8:39)]
names(a2.t1a)[3:4]<-c("maryland_ID", "Spec_Date")
a2.t1a$swab<-1
a2.t1b<-a2.t1[,c(1:2,4,6:7,8:39)]
names(a2.t1b)[c(3,5)]<-c("maryland_ID", "Spec_Date")
a2.t1b$swab<-2
a2.t1<-rbind(a2.t1a, a2.t1b)
a2.t1$ART_PREP_date<-a2.t1$ART_date

a2.t2<-read_excel("Feb_10_Email/Saidi_Aim2&3_Trial2_AnalyticSample.xlsx") ## PREP
dim(a2.t2) # 132 x 37
```


```
[1] 132  37
```


```
a2.t2$hiv<-0
a2.t2a<-a2.t2[,c(1:3,5:6,8:38)]
names(a2.t2a)[3:4]<-c("maryland_ID", "Spec_Date")
a2.t2a$swab<-1
a2.t2b<-a2.t2[,c(1:2,4,6:38)]
names(a2.t2b)[c(3, 5)]<-c("maryland_ID", "Spec_Date")
a2.t2b$swab<-2
a2.t2<-rbind(a2.t2a, a2.t2b)
a2.t2$ART_PREP_date<-a2.t2$PrEP_Date

a2<-merge(a2.t1, a2.t2, all=T)
a2$ART_date<-NULL
a2$PrEP_Date<-NULL

a2.mg<-merge(a2, mgCSTs[,c("maryland_ID", "mgCST2")], all.x=TRUE)
a2.mg<-merge(a2.mg, CSTs[,c("maryland_ID", "CST")], all.x=TRUE)
ordering<-unique(a2.mg[order(a2.mg$CST), "maryland_ID"])
a2.mg$maryland_ID<-factor(a2.mg$maryland_ID, levels=ordering)
a2.mg$PID<-factor(a2.mg$PID)
a2.mg$swab<-factor(a2.mg$swab)

treat.cols<-c("#B8DE29FF", "#33638DFF")
a2.mg$treatment<-factor(ifelse(a2.mg$hiv == 1, "ART", "PrEP"))
a2.mg$treatment<-factor(a2.mg$treatment, levels=c("ART", "PrEP"))

# OUTCOME
a2.mg$hiv<-factor(a2.mg$hiv, levels=c(1, 0), labels=c("WLHIV", "HIV-Negative"))
```


## HEATMAP Plot CST + Taxa


```
colfunc <- colorRampPalette(c("khaki", "limegreen", "darkslategray1", "mediumblue", "magenta", "red"))

#relabund.mgCST<-dcast(relabund.m[relabund$maryland_ID %in% sample.list, ], formula = maryland_ID~species, value.var = "relabund", fun.aggregate = sum)
relabund.mgCST<-dcast(counts.m[counts.m$maryland_ID %in% sample.list, ], formula = maryland_ID~taxa, value.var = "relabund", fun.aggregate = sum)
rownames(relabund.mgCST)<-relabund.mgCST$maryland_ID
relabund.mgCST$maryland_ID<-NULL
relabund.mgCST<-relabund.mgCST[,order(colSums(relabund.mgCST), decreasing = TRUE)]
relabund.mgCST$CST<-CSTs[match(rownames(relabund.mgCST), CSTs$maryland_ID), "CST"]

top.taxa.names<-names(relabund.mgCST)[1:50]
taxa.order<-c(sort(top.taxa.names[grep("Lactobacillus_crispatus", top.taxa.names)]),
sort(top.taxa.names[grep("Lactobacillus_iners", top.taxa.names)]),
sort(top.taxa.names[grep("Gardnerella_vaginalis", top.taxa.names)]),
sort(top.taxa.names[grep("Ca.Lachnocurva_vaginae", top.taxa.names)]))
taxa.order<-append(taxa.order, c(top.taxa.names[!top.taxa.names %in% taxa.order], "Prevotella_oris"))
               
the.dist<-vegdist(as.matrix(relabund.mgCST[,1:100]), na.rm = T)
the.clsting<-hclust(the.dist, method="ward.D")
top<-relabund.mgCST[,1:100]

to.write<-as.data.frame(cbind(sampleID=the.clsting$labels, order=the.clsting$order, domTaxa=colnames(top)[max.col(top, ties.method="first")], abund=apply(top, 1, max)))
to.write$CST<-relabund.mgCST[match(to.write$sampleID, rownames(relabund.mgCST)), "CST"]
to.write$cutree<-cutree(the.clsting, k=8)

to.write<-to.write[order(to.write$CST, to.write$domTaxa, to.write$abund), ]
sample.order<-to.write$sampleID

all.samples.hiv<-unique(rbind(a1.mg[,c("maryland_ID", "hiv")], a2.mg[,c("maryland_ID", "hiv")]))
all.samples.hiv$swab<-ifelse(to.write$sampleID %in% a2.mg$maryland_ID, as.character(a2.mg[match(to.write$sampleID, a2.mg$maryland_ID), "swab"]), "1")
all.samples.hiv$swab<-gsub("1", "M0", all.samples.hiv$swab)
all.samples.hiv$swab<-gsub("2", "M1", all.samples.hiv$swab)
all.samples.hiv$swab<-factor(all.samples.hiv$swab)
  
  a2.mg[match(all.samples.hiv$maryland_ID, a2.mg$maryland_ID), "swab"]
```


```
  [1] 1    <NA> <NA> 1    1    1    1    1    1    1    1    1    1    <NA> 1    1   
 [17] 1    <NA> <NA> 1    1    1    1    <NA> 1    1    1    1    1    1    1    <NA>
 [33] 1    <NA> <NA> 1    1    1    1    1    1    1    <NA> 1    <NA> 1    1    1   
 [49] 1    <NA> 1    1    1    1    1    <NA> 1    1    <NA> <NA> 1    <NA> 1    <NA>
 [65] 1    <NA> <NA> 1    1    <NA> <NA> 1    1    1    1    1    <NA> <NA> 1    1   
 [81] 1    1    1    <NA> 1    <NA> 1    <NA> <NA> 1    <NA> 1    <NA> 1    1    <NA>
 [97] 1    1    1    1    1    1    <NA> 1    1    <NA> <NA> <NA> 1    1    1    <NA>
[113] 1    1    <NA> 1    <NA> 1    1    <NA> 1    <NA> 1    <NA> 1    <NA> 1    <NA>
[129] 1    <NA> <NA> 1    <NA> 1    1    1    1    1    1    <NA> 1    <NA> 1    1   
[145] 1    1    1    1    1    1    <NA> 1    1    1    <NA> 1    <NA> 1    <NA> 1   
[161] <NA> 1    1    1    1    1    <NA> 1    1    <NA> 1    1    <NA> 1    1    1   
[177] 1    <NA> 1    1    1    <NA> 1    1    1    1    1    1    1    1    <NA> <NA>
[193] 1    1    <NA> 1    1    1    <NA> 1    1    1    1    <NA> 1    1    1    1   
[209] 1    <NA> 1    1    1    1    1    <NA> 1    1    1    1    1    1    1    1   
[225] 1    <NA> 1    1    1    1    <NA> 1    <NA> 1    1    <NA> 1    <NA> 1    1   
[241] 1    1    1    <NA> 1    1    1    1    <NA> 1    <NA> 1    <NA> <NA> 1    2   
[257] 2    2    2    2    2    2    2    2    2    2    2    2    2    2    2    2   
[273] 2    2    2    2    2    2    2    2    2    2    2    2    2    2    2    2   
[289] 2    2    2    2    2    2    2    2    2    2    2    2    2    2    2    2   
[305] 2    2    2    2    2    2    2    2    2    2    2    2    2    2    2    2   
[321] 2    2    2    2    2    2    2    2    2    2    2    2    2    2    2    2   
[337] 2    2    2    2    2    2    2    2    2    2    2    2    2    2    2    2   
[353] 2    2    2    2    2    2    2    2    2    2    2    2    2    2    2    2   
[369] 2    2    2    2    2    2    2    2    2    2    2    2    2    2    2    2   
[385] 2    2    2    2    2    2    2    2    2    2    2    2    2    2    2    2   
[401] 2    2    2    2    2    2    2    2    2    2    2    2    2    2    2    2   
[417] 2    2    2    2    2    2    2    2    2    2    2    2    2    2    2    2   
[433] 2    2    2    2   
Levels: 1 2
```


```
all.samples.hiv$swab[is.na(all.samples.hiv$swab)]<-"M0"

the.clusts<-as.data.frame(cbind(`VALENCIA CST`=as.character(to.write$CST), 
                                `HIV Serostatus`=as.character(all.samples.hiv[match(to.write$sampleID, all.samples.hiv$maryland_ID), "hiv"]), 
                                swab=as.character(all.samples.hiv[match(to.write$sampleID, all.samples.hiv$maryland_ID), "swab"])))

row.names(the.clusts)<-to.write$sampleID
the.clusts$`VALENCIA CST`<-as.factor(the.clusts$`VALENCIA CST`)
CST<-as.data.frame(rbind(c("I", "#FE0308"),c("I-A", "#FE0308"), c("I-B", "#F6D3DA"), c("II", "#86C61A"),c("III", "#FF7200"),c("III-A", "#FF7200"),c("III-B", "#F8A40E"), c("IV", "#221886"),c("IV-A", "#448A73"), c("IV-B", "#221886"), c("IV-C", "#C0ACD3"),c("IV-C0", "#989898"),c("IV-C1", "#EF53A7"),c("IV-C2", "#A7DDDC"),c("IV-C3", "#98C999"),c("IV-C4", "#7F0B7C"), c("V", "#FAE50D"), c("", "white"), c("NA", "white")))
cols<-CST[match(sort(unique(the.clusts$`VALENCIA CST`)), CST$V1), "V2"]
names(cols)<-unique(the.clusts$`VALENCIA CST`)

#to.plot<-relabund.mgCST[sample.order, 1:50]
to.plot<-relabund.mgCST[sample.order, taxa.order]
names(to.plot)<-gsub("_", " ", names(to.plot))
names(to.plot)<-gsub("_", " ", names(to.plot))
italic_row_labels <- lapply(names(to.plot), function(label) {
  return(bquote(italic(.(label))))
})

names(hiv.cols)<-c("WLHIV", "HIV-Negative")
swab.cols<-c("black", "gray")
names(swab.cols)<-c("M0", "M1")
colfunc <- colorRampPalette(c("khaki", "limegreen", "darkslategray1", "mediumblue", "magenta", "red"))

png(paste("Analysis_Figures/heatmap_dend_", today2, ".png", sep=""), width=7, height=5.5, units="in", res=600)
pheatmap(t(as.matrix(to.plot)), 
         color = alpha(colfunc(100), 1), 
         cluster_rows = FALSE, 
         cluster_cols = FALSE,
         annotation_row = NULL,
         legend = TRUE, 
         fontsize = 7, 
         labels_col = "", 
         annotation_col = the.clusts, 
         annotation_colors = list(`VALENCIA CST`=cols, `HIV Serostatus`=hiv.cols, swab=swab.cols),
         labels_row = as.expression(italic_row_labels)
         )
dev.off()
```


```
null device 
          1
```


```
tiff(paste("Analysis_Figures/heatmap_dend_", today2, ".tiff", sep=""), width=7, height=5.5)
```


```
pheatmap(t(as.matrix(to.plot)), 
         color = alpha(colfunc(100), 1), 
         cluster_rows = FALSE, 
         cluster_cols = FALSE,
         annotation_row = NULL,
         legend = TRUE, 
         fontsize = 7, 
         labels_col = "", 
         annotation_col = the.clusts, 
         annotation_colors = list(`VALENCIA CST`=cols, `HIV Serostatus`=hiv.cols, swab=swab.cols),
         labels_row = as.expression(italic_row_labels)
         )
dev.off()
```


```
null device 
          1
```

## Gardnerella speciation

### mgCSTs & Gardnerella


```
## norm spp. counts
temp<-reshape2::melt(counts, id.vars="maryland_ID", variable.name = "taxa", value.name = "counts")
temp<-merge(temp, relabund.m, all=TRUE)
temp$taxa<-gsub("\\._", "\\.", temp$taxa)
temp$coverage<-read.stats[match(temp$maryland_ID, read.stats$sample), "Reads_delivered"]
temp$spp<-paste(str_split_fixed(temp$taxa, pattern = "_", n = 3)[,1], str_split_fixed(temp$taxa, pattern = "_", n = 3)[,2], sep="_")
temp<-temp[!temp$maryland_ID %in% ctrls, ]

g<-read.delim("~/Dropbox (IGS)/mgss_mcst_devel/gardenerella_genomosp.txt", header=T)
g.counts<-genes[rownames(genes) %in% g$VIRGO_ID, ]
g.counts<-g.counts[,!names(g.counts) %in% ctrls] 
g.counts$Ggenomospecies<-g[match(rownames(g.counts), g$VIRGO_ID), "Species"] ## get genomosp
g.counts$VIRGO_ID<-rownames(g.counts)
g.counts.m<-reshape2::melt(g.counts, id.vars=c("VIRGO_ID", "Ggenomospecies"), variable.name = "maryland_ID", value.name = "normcounts") ## total counts
g.counts.m$mgCST<-mgCSTs[match(g.counts.m$maryland_ID, mgCSTs$maryland_ID), "mgCST2"] ## add mgCST

## get relabundance of G in a sample
total.Gcounts<-as.data.frame(temp[temp$spp %in% "Gardnerella_vaginalis", ] %>% dplyr::group_by(maryland_ID) %>% dplyr::summarise(totalCounts=sum(counts), relAbund=sum(relabund)))

g.counts.m.sum<-g.counts.m[g.counts.m$normcounts > 0, ] %>% dplyr::group_by(maryland_ID, Ggenomospecies, mgCST) %>% dplyr::summarise(nNRgenes=length(unique(VIRGO_ID)), gene_abundance=sum(normcounts))
```


```
`summarise()` has grouped output by 'maryland_ID', 'Ggenomospecies'. You can override
using the `.groups` argument.
```


```
g.counts.m.sum$coverage<-sample.summary[match(g.counts.m.sum$maryland_ID, sample.summary$maryland_ID), "finalReads_pe_se"]
g.counts.m.sum<-merge(g.counts.m.sum, total.Gcounts, all.x=TRUE)
g.counts.m.sum$propGgs<-g.counts.m.sum$gene_abundance/g.counts.m.sum$totalCounts
g.counts.m.sum$prop_of_G_gs<-g.counts.m.sum$relAbund*g.counts.m.sum$propGgs
names(g.counts.m.sum)[c(2, 5, 10)]<-c("taxa", "counts", "relabund")
  
counts.m.temp<-counts.m[!counts.m$spp %in% "Gardnerella_vaginalis", ]
counts.m.temp<-rbind(counts.m.temp[,c("maryland_ID", "taxa", "counts", "relabund")], g.counts.m.sum[,c("maryland_ID", "taxa", "counts", "relabund")])
counts.m.temp$taxa<-gsub("not assigned", "Gardnerella_vaginalis", counts.m.temp$taxa)
```

### FIG 2: HEATMAP Plot CST + Taxa w/Gard


```
colfunc <- colorRampPalette(c("khaki", "limegreen", "darkslategray1", "mediumblue", "magenta", "red"))

#relabund.mgCST<-dcast(relabund.m[relabund$maryland_ID %in% sample.list, ], formula = maryland_ID~species, value.var = "relabund", fun.aggregate = sum)
relabund.gard<-dcast(counts.m.temp[counts.m.temp$maryland_ID %in% sample.list, ], formula = maryland_ID~taxa, value.var = "counts", fun.aggregate = sum)
rownames(relabund.gard)<-relabund.gard$maryland_ID
relabund.gard$maryland_ID<-NULL
relabund.gard<-relabund.gard/rowSums(relabund.gard)
relabund.gard<-relabund.gard[,order(colSums(relabund.gard), decreasing = TRUE)]

top.taxa.names<-names(relabund.gard)[1:45]
taxa.order<-c(sort(top.taxa.names[grep("Lactobacillus_crispatus", top.taxa.names)]),
sort(top.taxa.names[grep("Lactobacillus_iners", top.taxa.names)]),
sort(top.taxa.names[grep("Gardnerella", top.taxa.names)]),
sort(top.taxa.names[grep("Ca.Lachnocurva_vaginae", top.taxa.names)]))
taxa.order<-append(taxa.order, c(top.taxa.names[!top.taxa.names %in% taxa.order]))

all.samples.hiv<-unique(rbind(a1.mg[,c("maryland_ID", "hiv")], a2.mg[,c("maryland_ID", "hiv")]))
all.samples.hiv$swab<-ifelse(to.write$sampleID %in% a2.mg$maryland_ID, as.character(a2.mg[match(to.write$sampleID, a2.mg$maryland_ID), "swab"]), "1")
all.samples.hiv$swab<-gsub("1", "M0", all.samples.hiv$swab)
all.samples.hiv$swab<-gsub("2", "M1", all.samples.hiv$swab)
all.samples.hiv$swab<-factor(all.samples.hiv$swab)
  
  a2.mg[match(all.samples.hiv$maryland_ID, a2.mg$maryland_ID), "swab"]
```


```
  [1] 1    <NA> <NA> 1    1    1    1    1    1    1    1    1    1    <NA> 1    1   
 [17] 1    <NA> <NA> 1    1    1    1    <NA> 1    1    1    1    1    1    1    <NA>
 [33] 1    <NA> <NA> 1    1    1    1    1    1    1    <NA> 1    <NA> 1    1    1   
 [49] 1    <NA> 1    1    1    1    1    <NA> 1    1    <NA> <NA> 1    <NA> 1    <NA>
 [65] 1    <NA> <NA> 1    1    <NA> <NA> 1    1    1    1    1    <NA> <NA> 1    1   
 [81] 1    1    1    <NA> 1    <NA> 1    <NA> <NA> 1    <NA> 1    <NA> 1    1    <NA>
 [97] 1    1    1    1    1    1    <NA> 1    1    <NA> <NA> <NA> 1    1    1    <NA>
[113] 1    1    <NA> 1    <NA> 1    1    <NA> 1    <NA> 1    <NA> 1    <NA> 1    <NA>
[129] 1    <NA> <NA> 1    <NA> 1    1    1    1    1    1    <NA> 1    <NA> 1    1   
[145] 1    1    1    1    1    1    <NA> 1    1    1    <NA> 1    <NA> 1    <NA> 1   
[161] <NA> 1    1    1    1    1    <NA> 1    1    <NA> 1    1    <NA> 1    1    1   
[177] 1    <NA> 1    1    1    <NA> 1    1    1    1    1    1    1    1    <NA> <NA>
[193] 1    1    <NA> 1    1    1    <NA> 1    1    1    1    <NA> 1    1    1    1   
[209] 1    <NA> 1    1    1    1    1    <NA> 1    1    1    1    1    1    1    1   
[225] 1    <NA> 1    1    1    1    <NA> 1    <NA> 1    1    <NA> 1    <NA> 1    1   
[241] 1    1    1    <NA> 1    1    1    1    <NA> 1    <NA> 1    <NA> <NA> 1    2   
[257] 2    2    2    2    2    2    2    2    2    2    2    2    2    2    2    2   
[273] 2    2    2    2    2    2    2    2    2    2    2    2    2    2    2    2   
[289] 2    2    2    2    2    2    2    2    2    2    2    2    2    2    2    2   
[305] 2    2    2    2    2    2    2    2    2    2    2    2    2    2    2    2   
[321] 2    2    2    2    2    2    2    2    2    2    2    2    2    2    2    2   
[337] 2    2    2    2    2    2    2    2    2    2    2    2    2    2    2    2   
[353] 2    2    2    2    2    2    2    2    2    2    2    2    2    2    2    2   
[369] 2    2    2    2    2    2    2    2    2    2    2    2    2    2    2    2   
[385] 2    2    2    2    2    2    2    2    2    2    2    2    2    2    2    2   
[401] 2    2    2    2    2    2    2    2    2    2    2    2    2    2    2    2   
[417] 2    2    2    2    2    2    2    2    2    2    2    2    2    2    2    2   
[433] 2    2    2    2   
Levels: 1 2
```


```
all.samples.hiv$swab[is.na(all.samples.hiv$swab)]<-"M0"

the.clusts<-as.data.frame(cbind( swab=as.character(all.samples.hiv[match(to.write$sampleID, all.samples.hiv$maryland_ID), "swab"]),
                                `HIV Serostatus`=as.character(all.samples.hiv[match(to.write$sampleID, all.samples.hiv$maryland_ID), "hiv"]), 
                                 `VALENCIA CST`=as.character(to.write$CST)))

row.names(the.clusts)<-to.write$sampleID
the.clusts$`VALENCIA CST`<-as.factor(the.clusts$`VALENCIA CST`)
the.clusts$`HIV Serostatus`<-factor(the.clusts$`HIV Serostatus`, levels=c("WLHIV", "HIV-Negative"))
#sample.order<-rownames(the.clusts)[order(the.clusts$swab, the.clusts$`HIV Serostatus`, the.clusts$`VALENCIA CST`)]
sample.order<-rownames(the.clusts)[order(the.clusts$`VALENCIA CST`, the.clusts$`HIV Serostatus`)]


CST<-as.data.frame(rbind(c("I", "#FE0308"),c("I-A", "#FE0308"), c("I-B", "#F6D3DA"), c("II", "#86C61A"),c("III", "#FF7200"),c("III-A", "#FF7200"),c("III-B", "#F8A40E"), c("IV", "#221886"),c("IV-A", "#448A73"), c("IV-B", "#221886"), c("IV-C", "#C0ACD3"),c("IV-C0", "#989898"),c("IV-C1", "#EF53A7"),c("IV-C2", "#A7DDDC"),c("IV-C3", "#98C999"),c("IV-C4", "#7F0B7C"), c("V", "#FAE50D"), c("", "white"), c("NA", "white")))
cols<-CST[match(sort(unique(the.clusts$`VALENCIA CST`)), CST$V1), "V2"]
names(cols)<-unique(the.clusts$`VALENCIA CST`)

#to.plot<-relabund.mgCST[sample.order, 1:50]
to.plot<-relabund.gard[sample.order, taxa.order[1:45]]
the.dist<-vegdist(as.matrix(to.plot), na.rm = T)
the.clsting<-hclust(the.dist, method="ward.D")

names(to.plot)<-gsub("_", " ", names(to.plot))
names(to.plot)<-gsub("_", " ", names(to.plot))
italic_row_labels <- lapply(names(to.plot), function(label) {
  return(bquote(italic(.(label))))
})

names(hiv.cols)<-c("WLHIV", "HIV-Negative")
swab.cols<-c("black", "gray")
names(swab.cols)<-c("M0", "M1")
colfunc <- colorRampPalette(c("khaki", "limegreen", "darkslategray1", "mediumblue", "magenta", "red"))
#tiff(paste("Manuscript_FIGURES/Figure_2_", today2, ".tiff", sep=""), width=4, height=3)
#width_in_pixels <- width_in_inches * high_res
#height_in_pixels <- height_in_inches * high_res

tiff(filename = paste("Manuscript_FIGURES/Figure_2_", today2, ".tiff", sep=""), width = 2400, height = 1800, res = 600)
pheatmap(t(as.matrix(to.plot)), 
         color = alpha(colfunc(100), 1), 
         cluster_rows = FALSE, 
         cluster_cols = FALSE,
         annotation_row = NULL,
         annotation_legend = TRUE,
         legend = TRUE, 
         fontsize = 3, 
         show_colnames = F,
         annotation_col = the.clusts, 
         annotation_colors = list(`VALENCIA CST`=cols, `HIV Serostatus`=hiv.cols, swab=swab.cols),
         labels_row = as.expression(italic_row_labels),
         width = 3,
         height = 4
         )
dev.off()
```


```
null device 
          1
```

# Aim 1, HIV (+) to (-) x.s.

## Covariates


```
# FIXED EFFECTS - to reduce the chances that an HIV/microbiome association is confounded by something else.
label(a1.mg$sd_age)<-"Age at enrollment (years)"
covar<-c("sd_age")

## ph_ega: estimated gestational age at enrollment: Continuous 
label(a1.mg$ph_ega)<-"Gestational age at enrollment (weeks)"
covar<-append(covar, "ph_ega")

# Gravidity: "0=No prior pregnancies, 1=At least one prior pregnancy"
a1.mg$gravidity<-factor(a1.mg$gravidity, levels=c(0, 1), labels=c("No prior pregnancies", "At least one prior pregnancy"))
label(a1.mg$gravidity)<-"Gravidity"
covar<-append(covar, "gravidity")

# Primary partner HIV status: 0=HIV-negative, 1=HIV-positive, 2=Indeterminate, 3=Partner never tested for HIV, 998=I don't know, 999=No response
a1.mg$p1_status[a1.mg$p1_status %in% c(3, 998)]<-3
a1.mg$p1_status<-factor(a1.mg$p1_status, levels=c(0, 1, 3), labels=c("HIV-negative", "HIV-positive", "Don't know"))
label(a1.mg$p1_status)<-"Primary partner HIV serostatus *"
covar<-append(covar, "p1_status")

# # Number of sexual intercourse acts in the past 30 days: "0=No partners, 1=One partner, 2=Multiple partners"
# a1.mg$sex_freq_cat<-ifelse(a1.mg$sex_freq %in% 0, 0, ifelse(a1.mg$sex_freq %in% 1-5, 1, ifelse(is.na(a1.mg$sex_freq), 3, 2)))
# a1.mg$sex_freq_cat<-factor(a1.mg$sex_freq, levels=c(0, 1, 2, 3), labels=c("0", "1-5", ">5", "Abstained"))
# label(a1.mg$sex_freq_cat)<-"Number of sexual intercourse acts in the past 30 days"
# covar<-append(covar, "sex_freq_cat")

# Number of partners in past three months: "0=No partners, 1=One partner, 2=Multiple partners"
a1.mg$curr_ptnrs<-factor(a1.mg$curr_ptnrs, levels=c(0, 1, 2), labels=c("0", "1", "Multiple")) 
label(a1.mg$curr_ptnrs)<-"Number of partners in past three months"
covar<-append(covar, "curr_ptnrs")

#FIX a1.mg$condom_freq_cat ... try grouping sometimes with never - how does this change?
#Consistent condom use with primary partner in past 30 days: "0=Never, 1=Sometimes, 2=Consistent"
a1.mg$condom_freq_cat_f<-a1.mg$condom_freq_cat
a1.mg$condom_freq_cat_f[a1.mg$condom_freq_cat_f %in% c(0, 1)]<-0
a1.mg$condom_freq_cat_f[a1.mg$condom_freq_cat_f %in% 2]<-2
a1.mg$condom_freq_cat_f[is.na(a1.mg$condom_freq_cat)]<-3
a1.mg$condom_freq_cat_f<-factor(a1.mg$condom_freq_cat_f, levels=c(0, 2, 3), labels=c("Never or Sometimes", "Consistent", "Abstained"))
a1.mg$condom_freq_cat_f<-relevel(a1.mg$condom_freq_cat_f, ref="Abstained")
label(a1.mg$condom_freq_cat_f)<-"Consistent condom use with primary partner in past 30 days ^"
# covar<-append(covar, "condom_freq_cat_f")

## ph_syphilis_told: self-report syphilis past 3 months. CAT
# Results of syphilis test during index pregnancy (antenatal record): "0=Nonreactive, 1=Reactive, 999=No test results recorded"
a1.mg$bltest_sypresults<-factor(a1.mg$bltest_sypresults, levels=c(0, 1), labels = c("No", "Yes"))
label(a1.mg$bltest_sypresults)<-"Diagnosed with syphilis in past 3 months"
covar<-append(covar, "bltest_sypresults")

# Abnormal vaginal discharge observed in past 3 months (self-report): "0=No, 1=Yes, 998=I don't know, 999=No Response"
a1.mg$ph_discharge<-factor(a1.mg$ph_discharge, levels=c(0, 1), labels=c("No", "Yes"))
label(a1.mg$ph_discharge)<-"Abnormal vaginal discharge observed in past 3 months (self-report)"
covar<-append(covar, "ph_discharge")

# Genital ulcers observed in past 3 months (self-report): "0=No, 1=Yes, 998=I don't know, 999=No Response"
a1.mg$ph_sores_ulcers<-factor(a1.mg$ph_sores_ulcers, levels=c(0, 1), labels=c("No", "Yes"))
label(a1.mg$ph_sores_ulcers)<-"Genital ulcers observed in past 3 months (self-report)"
covar<-append(covar, "ph_sores_ulcers")

# Running water in home: "0=No, 1=Yes"
a1.mg$water<-factor(a1.mg$water, levels=c(0, 1), labels=c("No", "Yes"))
label(a1.mg$water)<-"Running water in home"
covar<-append(covar, "water")

# Pregnancy Outcome
a1.mg$preg_outcome<-a2$preg_outcome[match(a1.mg$maryland_ID, a2$maryland_ID)]
meta<-read.csv("Feb_10_email/TP2_pregoutcome_needed.csv")
a1.mg$preg_outcome<-ifelse(is.na(a1.mg$preg_outcome), meta$preg_outcome[match(a1.mg$maryland_ID, meta$maryland_ID)], as.character(a1.mg$preg_outcome))
a1.mg$preg_outcome_clean<-factor(ifelse(grepl("Preterm Delivery", a1.mg$preg_outcome), "Preterm Delivery", ifelse(grepl("Term Delivery", a1.mg$preg_outcome), "Term Delivery", NA)), levels=c("Term Delivery", "Preterm Delivery"))
a1.mg$preg_outcome_clean<-relevel(a1.mg$preg_outcome_clean, ref="Term Delivery")
label(a1.mg$preg_outcome_clean)<-"Pregnancy Outcome"

covars<-paste(covar, collapse=" + ")
table1(~ sd_age + ph_ega + gravidity + p1_status + curr_ptnrs + condom_freq_cat_f + bltest_sypresults + ph_discharge + ph_sores_ulcers + water + preg_outcome_clean  | hiv, data=a1.mg, overall=F, extra.col=list(`P-value`=pvalue))
```


```
Warning in chisq.test(table(y, g)) :
  Chi-squared approximation may be incorrect
Warning in chisq.test(table(y, g)) :
  Chi-squared approximation may be incorrect
Warning in chisq.test(table(y, g)) :
  Chi-squared approximation may be incorrect
Warning in chisq.test(table(y, g)) :
  Chi-squared approximation may be incorrect
```


|  | WLHIV (N=64) | HIV-Negative (N=191) | P-value |
| --- | --- | --- | --- |
| Age at enrollment (years) |  |  |  |
| Mean (SD) | 27.4 (6.02) | 25.4 (5.45) | 0.02 |
| Median [Min, Max] | 26.0 [18.0, 42.0] | 24.0 [18.0, 40.0] |  |
| Gestational age at enrollment (weeks) |  |  |  |
| Mean (SD) | 23.6 (6.40) | 25.6 (8.84) | 0.06 |
| Median [Min, Max] | 24.0 [10.0, 37.0] | 26.0 [6.00, 41.0] |  |
| Gravidity |  |  |  |
| No prior pregnancies | 13 (20.3%) | 42 (22.0%) | 0.92 |
| At least one prior pregnancy | 51 (79.7%) | 149 (78.0%) |  |
| Primary partner HIV serostatus \* |  |  |  |
| HIV-negative | 13 (20.3%) | 139 (72.8%) | <0.01 |
| HIV-positive | 11 (17.2%) | 9 (4.7%) |  |
| Don't know | 37 (57.8%) | 39 (20.4%) |  |
| Missing | 3 (4.7%) | 4 (2.1%) |  |
| Number of partners in past three months |  |  |  |
| 0 | 3 (4.7%) | 4 (2.1%) | 0.24 |
| 1 | 61 (95.3%) | 182 (95.3%) |  |
| Multiple | 0 (0%) | 5 (2.6%) |  |
| Consistent condom use with primary partner in past 30 days ^ |  |  |  |
| Abstained | 11 (17.2%) | 26 (13.6%) | 0.3 |
| Never or Sometimes | 53 (82.8%) | 159 (83.2%) |  |
| Consistent | 0 (0%) | 6 (3.1%) |  |
| Diagnosed with syphilis in past 3 months |  |  |  |
| No | 59 (92.2%) | 109 (57.1%) | <0.01 |
| Yes | 5 (7.8%) | 79 (41.4%) |  |
| Missing | 0 (0%) | 3 (1.6%) |  |
| Abnormal vaginal discharge observed in past 3 months (self-report) |  |  |  |
| No | 61 (95.3%) | 111 (58.1%) | <0.01 |
| Yes | 3 (4.7%) | 80 (41.9%) |  |
| Genital ulcers observed in past 3 months (self-report) |  |  |  |
| No | 61 (95.3%) | 154 (80.6%) | 0.01 |
| Yes | 3 (4.7%) | 37 (19.4%) |  |
| Running water in home |  |  |  |
| No | 49 (76.6%) | 137 (71.7%) | 0.55 |
| Yes | 15 (23.4%) | 54 (28.3%) |  |
| Pregnancy Outcome |  |  |  |
| Term Delivery | 48 (75.0%) | 159 (83.2%) | 0.17 |
| Preterm Delivery | 7 (10.9%) | 10 (5.2%) |  |
| Missing | 9 (14.1%) | 22 (11.5%) |  |


### Species richness & diversity (Chao richness & Shannon)

24Feb - the results look as-expected for prior HIV studies according
to Joni. – Update analyses to account for covariate data. #####-
Diversity by raw and rarefied data


```
counts<-as.data.frame(reshape2::dcast(counts.m, maryland_ID~taxa2, value.var="counts", fun.aggregate = sum))
rownames(counts)<-counts$maryland_ID
counts$maryland_ID<-NULL
exp<-phyloseq(otu_table(round(counts[rownames(counts) %in% a1.mg$maryland_ID, ]), taxa_are_rows = F))
counts$maryland_ID<-rownames(counts)
rownames(a1.mg)<-a1.mg$maryland_ID
exp<-merge_phyloseq(exp, sample_data(a1.mg))
rich<-estimate_richness( exp, measures = c("Chao1", "Shannon"))
rich$maryland_ID<-rownames(rich)
a1.mg<-merge(a1.mg, rich, all.x=TRUE)
```


#####- Alpha Diversity & HIV


```
a1.mg %>%
  summarise(
    count = n(),
    median = median(Shannon, na.rm = TRUE),
    IQR = IQR(Shannon, na.rm = TRUE)
  )
```


```
group_by(a1.mg, hiv) %>%
  summarise(
    count = n(),
    median = median(Shannon, na.rm = TRUE),
    IQR = IQR(Shannon, na.rm = TRUE)
  )
```


```
wilcox.test(Shannon ~ hiv, data = a1.mg,
                   exact = FALSE)
```


```
    Wilcoxon rank sum test with continuity correction

data:  Shannon by hiv
W = 8756, p-value = 2.26e-07
alternative hypothesis: true location shift is not equal to 0
```


```
wilcox_test<-wilcox.test(Shannon ~ hiv, data = a1.mg,
                   exact = FALSE)
p_value <- wilcox_test$p.value
significance <- ifelse(p_value < 0.001, "***", ifelse(p_value < 0.01, "**", ifelse(p_value < 0.05, "*", "ns")))
#At enrollment (prior to ARV initation), median Shannon diversity of all vaginal microbiomes was 0.8, with a significantly higher value observed among WLHIV compared to HIV-negative women (1.45 vs. 0.7, p<0.001, Figure 3A).
```


######FIG 3A: Shannon & HIV


```
cmp<-list(c("WLHIV", "HIV-Negative"))
p1<-ggplot(a1.mg, aes(x=hiv, y=Shannon, fill=hiv))+
  geom_boxplot(color="black", lwd=0.1, outlier.shape = NA, notch = T)+
  scale_fill_manual(values=hiv.cols, label=c("HIV(-)", "WLHIV"), name="")+
  geom_point(position=position_jitterdodge(), size=0.01)+
  theme_bw()+
  theme(legend.position = "none",
        text=element_text(size=6, color="black"),
        line = element_line(linewidth = 0.1), 
        axis.title = element_text(size=5), 
        axis.text = element_text(color="black"))+
  ylab("Shannon Diversity")+
  xlab("")+
  geom_signif(comparisons = cmp, 
              annotations = significance, 
              y_position = 2.8, 
              tip_length = 0, 
              map_signif_level = c("***"=0.001, "**"=0.01, "*"=0.05), 
              vjust = 0.8, 
              size = 0.3, 
              textsize = 3)
#ggsave(paste("Manuscript_FIGURES/Figure_3a", today2, ".tiff", sep=""), height = 3, width=2, dpi = 600)
```


#####- Alpha Diversity & HIV


```
### ChiSq
chisq_test<-chisq.test(x = a1.mg$CST, y=a1.mg$hiv)
```


```
Warning in chisq.test(x = a1.mg$CST, y = a1.mg$hiv) :
  Chi-squared approximation may be incorrect
```


```
p_value <- chisq_test$p.value
significance <- ifelse(p_value < 0.001, "***", ifelse(p_value < 0.01, "**", ifelse(p_value < 0.05, "*", "ns")))

## X-squared = 25.219, df = 3, p-value = 1.39e-05
#Baseline HIV serostatus was associated with CST (p<0.001, Figure 3B).
```


######FIG 3B: Shannon & HIV Test proportion of CST by HIV


```
chi.test <- function(a, b) {
  return(chisq.test(cbind(a, b)))
}
## make df: CST hiv n
to.plot<-as.data.frame(a1.mg[,c("hiv", "CST", "maryland_ID")] %>% 
                         group_by(hiv, CST) %>% 
                         summarise(n=length(unique(maryland_ID)))) %>% 
                         group_by(hiv) %>%
                         mutate(freq = n / sum(n))
```


```
`summarise()` has grouped output by 'hiv'. You can override using the `.groups`
argument.
```


```
cst.cols<-CST.col[match(sort(unique(to.plot$CST)), CST.col$CST), "color"]

to.plot <- to.plot %>%
  group_by(hiv) %>%
  mutate(y_pos = 1-(cumsum(freq) - 0.5 * freq))

p2<-ggplot(to.plot, aes(x=hiv, y=freq, fill=CST, label=CST))+
  geom_bar(stat="identity", position = "fill", color="black", lwd=0.2)+
  geom_text(aes(y=y_pos), size=1.5, color="white")+
  scale_fill_manual(values=cst.cols)+
  theme_bw()+
  theme(legend.position="none", 
        text=element_text(size=6, color="black"), 
        line = element_line(linewidth = 0.1), 
        axis.title = element_text(size=5), 
        axis.text = element_text(color="black"))+
  xlab("")+
  ylab("Proportion of Samples in CST")+
  ylim(c(0,1.1))+
  geom_signif(comparisons = list(c("WLHIV","HIV-Negative")), 
              annotations=significance,
              y_position = 1.01, 
              tip_length = 0, 
              map_signif_level = c("***"=0.001, "**"=0.01, "*"=0.05), 
              vjust = 0.4, 
              size = 0.3, 
              textsize = 3)
#ggsave(paste("Manuscript_FIGURES/Figure_3b", today2, ".tiff", sep=""), height = 3, width=2, dpi = 600)
```


#####- ODDS of CST GIVEN HIV STATUS


```
#stratifying the data by CST + covariates is very tough. Which covariates are absolutely needed? 
## NOTE: CST V == 2 samples --> combine with CST I or remove? 
## NOTE: CST IV-C == 2 samples --> remove?
## Remove covariates due to sparseness: condom_freq_cat_f, curr_ptnrs
### USE THIS ### 
## Does not converge when using condom_freq_cat_f for CST IV-B
require(nnet)
```


```
Loading required package: nnet
```


```
v<-a1.mg
v$hiv<-factor(v$hiv, levels=c("WLHIV", "HIV-Negative"))
v$hiv<-relevel(v$hiv, ref="HIV-Negative")
v$CST<-ifelse(v$CST %in% c("I", "III"), 0, 1)
v$CST<-factor(v$CST, levels=c(0,1), labels=c("I or III", "IV"))
the.model<-glm(CST~hiv + sd_age + ph_ega + gravidity + p1_status + curr_ptnrs + bltest_sypresults + ph_discharge + ph_sores_ulcers + water, data = v, family = "binomial")
c<-as.data.frame(broom::tidy(the.model, conf.int = T, exponentiate = T))
c[grepl("WLHIV", c$term), ]
```


```
# Compared to WLHIV, HIV-negative women had three times the odds of being assigned to CST I or III at enrollment (CST I: aOR: 5.5, 95% CI: 1.1, 55, padj=0.07; CST III: aOR: 2.9, 95% CI: 1.2, 7.8, padj=0.09, Figure 3C).
```


######FIG 3C: Proportion of HIV in CST


```
to.plot<-a1.mg[a1.mg$CST %in% c("I", "III", "IV-B"), ]
to.plot$CST<-ifelse(to.plot$CST %in% c("I", "III"), "I or III", as.character(to.plot$CST))
to.plot$CST<-gsub("IV-B", "IV-B **", to.plot$CST)
p3<-ggplot(to.plot, aes(x=CST, y=..prop.., fill=hiv, group=hiv))+
  geom_bar(stat="count", position="dodge", width=0.7, color="black", lwd=0.1)+
  scale_fill_manual(values=hiv.cols, label=c("WLHIV, n=64", "HIV(-), n=191"), name="")+
  theme_bw()+
  theme(legend.position = c(0.2,0.9), 
        text=element_text(size=6, color="black"), 
        axis.title = element_text(size=5), 
        axis.text = element_text(color="black"),
        legend.key.size = unit(0.3, "cm"), 
        legend.background = element_blank(),
        line = element_line(linewidth = 0.1))+
  ylab("Proportion of Samples in HIV Group")+
  xlab("Community State Type (CST)")#+
```


```
Warning: A numeric `legend.position` argument in `theme()` was deprecated in ggplot2 3.5.0.
ℹ Please use the `legend.position.inside` argument of `theme()` instead.
This warning is displayed once every 8 hours.
Call `lifecycle::last_lifecycle_warnings()` to see where this warning was generated.
```


```
  #annotate(geom = "text", x = 3, y = 0.75, label = "**", size=3)
  #geom_signif(comparisons = list(c("WLHIV","HIV-Negative")), test = "chi.test", y_position = -3.15, tip_length = 0, map_signif_level = c("****"=0.001, "***"=0.01, "*"=0.05), vjust = 0.8, size = 0.3, textsize = 3)

#ggsave(paste("Manuscript_FIGURES/Figure_3c", today2, ".tiff", sep=""), height = 3, width=3, dpi = 600)
```


##### FIG 3: PRINT


```
top_row = ggarrange(p1, p2, p3, ncol = 3, labels = c("A.", "B.", "C."), font.label = list(face="plain", size=8))
```


```
Warning: The dot-dot notation (`..prop..`) was deprecated in ggplot2 3.4.0.
ℹ Please use `after_stat(prop)` instead.
This warning is displayed once every 8 hours.
Call `lifecycle::last_lifecycle_warnings()` to see where this warning was generated.
```


```
top_row
ggsave(paste("Manuscript_FIGURES/Figure_3_", today2, ".tiff", sep=""), height = 3, width=7, dpi = 600)
```

### Taxa Specific Analysis

— This representation needs to be improved – colors and also sorting.
— use deseq based code here – see zappgapps scripts for looping through
CSTs.


```
#counts<-read.csv("20Mar2023/norm_counts_mgSs_mgCST_20Mar2023.csv", header=T, stringsAsFactors = F, check.names = F)
#names(counts)[1]<-"maryland_ID"
#counts<-dcast(counts.m[counts.m$maryland_ID %in% sample.list, ], formula = maryland_ID~taxa, value.var = "counts", fun.aggregate = sum)
counts<-dcast(counts.m.temp[counts.m.temp$maryland_ID %in% sample.list, ], formula = maryland_ID~taxa, value.var = "counts", fun.aggregate = sum)

## Remove all taxa found in zymo positive controls
mgss0<-names(counts)[grepl("0$", names(counts))]
counts<-counts[,!names(counts) %in% c("Enterococcus_faecalis", "Escherichia_coli", "Lactobacillus_fermentum", "Salmonella_enterica", "Staphylococcus_aureus", "Candidatus_Pelagibacter", mgss0, to.rm)]

rownames(counts)<-counts$maryland_ID
counts$maryland_ID<-NULL
counts.all<-counts

samples<-a1.mg$maryland_ID
counts<-counts.all[rownames(counts.all) %in% samples, ]
names(counts)<-gsub("_\\.", " ", names(counts))
names(counts)<-gsub("_", " ", names(counts))
names(counts)<-gsub(pattern = "\\.\\.", replacement = " ", names(counts))
names(counts)<-gsub(pattern = "\\.", replacement = " ", names(counts))
counts<-counts[,!is.na(names(counts))]
counts$maryland_ID<-rownames(counts)

d.m<-reshape2::melt(counts, id.vars = "maryland_ID", variable.name = "Taxon", value.name = "count", na.rm = T)
d.m<-merge(d.m, a1.mg[,c("maryland_ID", "hiv")], all.x=TRUE)

samples.x.taxon<-d.m[d.m$count > 0, ] %>% dplyr::group_by(Taxon, hiv) %>% dplyr::summarise(nSamples=length(unique(maryland_ID)))
```


```
`summarise()` has grouped output by 'Taxon'. You can override using the `.groups`
argument.
```


```
prop.table(table(a1.mg$hiv))
```


```
       WLHIV HIV-Negative 
   0.2509804    0.7490196
```


```
samples.x.taxon.df<-reshape2::dcast(samples.x.taxon, Taxon~hiv, value.var = "nSamples")
samples.x.taxon.df$`NA`<-NULL
samples.x.taxon.df[is.na(samples.x.taxon.df)]<-0
samples.x.taxon.df$Total<-samples.x.taxon.df$`WLHIV`+samples.x.taxon.df$`HIV-Negative`

## Test only those taxa which are present in at least 5% of samples =  min. 8 samples = 107 taxa
keep<-as.vector(samples.x.taxon.df[samples.x.taxon.df$Total >= 0.1*nrow(counts), "Taxon"])
c<-reshape2::dcast(d.m[d.m$Taxon %in% keep, ], Taxon~maryland_ID, value.var = "count", fun.aggregate = sum)
rownames(c)<-c$Taxon
c<-c[-1]
c<-c[, colSums(c) > 0]
c<-c[rowSums(c) > 0,  ]

meta.sym<-a1.mg[,c("maryland_ID", covar, "hiv")]
meta.sym<-meta.sym[meta.sym$maryland_ID %in% names(c), ]
meta.sym<-meta.sym[complete.cases(meta.sym), ]
c<-c[,colnames(c) %in% meta.sym$maryland_ID]

obj.sym<-DESeqDataSetFromMatrix(countData = round(c), colData = meta.sym, design=~ sd_age + ph_ega + gravidity + p1_status + curr_ptnrs + bltest_sypresults + ph_discharge + ph_sores_ulcers + water + hiv)
```


```
converting counts to integer mode
  the design formula contains one or more numeric variables with integer values,
  specifying a model with increasing fold change for higher values.
  did you mean for this to be a factor? if so, first convert
  this variable to a factor using the factor() function
  the design formula contains one or more numeric variables that have mean or
  standard deviation larger than 5 (an arbitrary threshold to trigger this message).
  Including numeric variables with large mean can induce collinearity with the intercept.
  Users should center and scale numeric variables in the design to improve GLM convergence.
factor levels were dropped which had no samples
  Note: levels of factors in the design contain characters other than
  letters, numbers, '_' and '.'. It is recommended (but not required) to use
  only letters, numbers, and delimiters '_' or '.', as these are safe characters
  for column names in R. [This is a message, not a warning or an error]
```


```
#obj.sym$hiv<-relevel(obj.sym$hiv, ref="0")
obj.sym = estimateSizeFactors( obj.sym, type="poscounts" )
```


```
  Note: levels of factors in the design contain characters other than
  letters, numbers, '_' and '.'. It is recommended (but not required) to use
  only letters, numbers, and delimiters '_' or '.', as these are safe characters
  for column names in R. [This is a message, not a warning or an error]
```


```
obj.sym = estimateDispersions( obj.sym, fitType="local" )
```


```
gene-wise dispersion estimates
mean-dispersion relationship
  Note: levels of factors in the design contain characters other than
  letters, numbers, '_' and '.'. It is recommended (but not required) to use
  only letters, numbers, and delimiters '_' or '.', as these are safe characters
  for column names in R. [This is a message, not a warning or an error]
final dispersion estimates
```


```
## limma + voom
hiv<-make.names(meta.sym$hiv)
sd_age<-meta.sym$sd_age
ph_ega<-meta.sym$ph_ega
gravidity<-make.names(meta.sym$gravidity)
p1_status<-make.names(meta.sym$p1_status)
curr_ptnrs<-make.names(meta.sym$curr_ptnrs)
bltest_sypresults<-make.names(meta.sym$bltest_sypresults)
ph_discharge<-make.names(meta.sym$ph_discharge)
ph_sores_ulcers<-make.names(meta.sym$ph_sores_ulcers)
water<-make.names(meta.sym$water)

#design<-model.matrix(~ sd_age + ph_ega + gravidity + p1_status + curr_ptnrs + bltest_sypresults + ph_discharge + ph_sores_ulcers + water + hiv)
## Model does not converge when including curr_ptnrs
design<-model.matrix(~sd_age + ph_ega + gravidity + p1_status + bltest_sypresults + ph_discharge + ph_sores_ulcers + water + hiv)

nc <- counts(obj.sym, normalized=TRUE)
d0 <- DGEList(nc)
d0 <- calcNormFactors(d0)
v<-voom(d0, design, plot=T)
```


```
fit<-lmFit(v, design)
tmp <- eBayes(fit)

top.table <- topTable(tmp, adjust.method = "BH", sort.by = "F", n = Inf)
```


```
Removing intercept from test coefficients
```


```
top.table[top.table$adj.P.Val < 0.01, ]
```


```
top.table$taxa<-rownames(top.table)
taxa.all.results.pre<-top.table
taxa.all.results.pre<-unique(taxa.all.results.pre)
taxa.all.results.pre$association<-ifelse(taxa.all.results.pre$adj.P.Val < 0.011, ifelse(taxa.all.results.pre$hivWLHIV < 0, "Position Association with HIV(-) status", "Position Association with WLHIV"), "Not Significant")
taxa.all.results.pre$taxa<-factor(taxa.all.results.pre$taxa)
```


#### FIG 4: Taxa by HIV serostatus


```
## The reference here is HIV- "0".
ggplot(taxa.all.results.pre[taxa.all.results.pre$adj.P.Val < 0.011 & !is.na(taxa.all.results.pre$adj.P.Val) & !taxa.all.results.pre$taxa %in% "NA", ], aes(x=hivWLHIV, y=reorder(taxa,hivWLHIV), fill=association))+
  geom_bar(stat="identity", color="black", lwd=0.1)+
  xlab("Log-Fold Difference in mgSs Abundance\n(WLHIV / HIV-Negative)")+
  scale_fill_manual(values=rev(as.vector(hiv.cols)), name="Associated with:", labels=c("HIV(-)", "WLHIV"))+
  theme_bw()+
  theme(text=element_text(size=5, color="black"), 
        axis.text = element_text(color="black"),
        axis.text.y = element_text(face="italic", "black", family="Arial"), 
        legend.position = "none", 
        legend.background = element_rect(fill="transparent"),
        axis.ticks = element_line(linewidth = 0.1), 
        legend.key.size = unit(0.4, "cm"), 
        line = element_line(linewidth = 0.5))+
  ylab("")
ggsave(paste("Manuscript_FIGURES/Figure_4_",today2, ".tiff", sep=""), height=4, width=3, dpi = 600)
```


```
write.csv(taxa.all.results.pre, paste("Analysis_Figures/Table_mgSs_Aim1_",today2,".csv", sep=""), quote=F)
```

# Aim 2, PrEP ART Long

## Data & Covariates (at enrollment)


```
## COVARIATES
# FIXED EFFECTS - to reduce the chances that an HIV/microbiome association is confounded by something else.
label(a2.mg$sd_age)<-"Age at enrollment (years)"
covar<-c("sd_age")

## ph_ega: estimated gestational age at enrollment: Continuous 
label(a2.mg$ph_ega)<-"Gestational age at enrollment (weeks)"
covar<-append(covar, "ph_ega")

# Gravidity: "0=No prior pregnancies, 1=At least one prior pregnancy"
a2.mg$gravidity<-factor(a2.mg$gravidity, levels=c(0, 1), labels=c("No prior pregnancies", "At least one prior pregnancy"))
label(a2.mg$gravidity)<-"Gravidity"
covar<-append(covar, "gravidity")

# Primary partner HIV status: 0=HIV-negative, 1=HIV-positive, 2=Indeterminate, 3=Partner never tested for HIV, 998=I don't know, 999=No response
a2.mg$p1_status[a2.mg$p1_status %in% c(3, 998)]<-3
a2.mg$p1_status<-factor(a2.mg$p1_status, levels=c(0, 1, 3), labels=c("HIV-negative", "HIV-positive", "Don't know"))
label(a2.mg$p1_status)<-"Primary partner HIV serostatus *"
covar<-append(covar, "p1_status")

# Number of partners in past three months: "0=No partners, 1=One partner, 2=Multiple partners"
a2.mg$curr_ptnrs<-factor(a2.mg$curr_ptnrs, levels=c(0, 1, 2), labels=c("0", "1", "Multiple")) 
label(a2.mg$curr_ptnrs)<-"Number of partners in past three months"
covar<-append(covar, "curr_ptnrs")

#FIX a2.mg$condom_freq_cat ... try grouping sometimes with never - how does this change?
#Consistent condom use with primary partner in past 30 days: "0=Never, 1=Sometimes, 2=Consistent"
a2.mg$condom_freq_cat_f<-a2.mg$condom_freq_cat
a2.mg$condom_freq_cat_f[a2.mg$condom_freq_cat_f %in% c(0, 1)]<-0
a2.mg$condom_freq_cat_f[a2.mg$condom_freq_cat_f %in% 2]<-2
a2.mg$condom_freq_cat_f[is.na(a2.mg$condom_freq_cat)]<-3
a2.mg$condom_freq_cat_f<-factor(a2.mg$condom_freq_cat_f, levels=c(0, 2, 3), labels=c("Never or Sometimes", "Consistent", "Abstained"))
a2.mg$condom_freq_cat_f<-relevel(a2.mg$condom_freq_cat_f, ref="Abstained")
label(a2.mg$condom_freq_cat_f)<-"Consistent condom use with primary partner in past 30 days ^"
# covar<-append(covar, "condom_freq_cat_f")

## ph_syphilis_told: self-report syphilis past 3 months. CAT
# Results of syphilis test during index pregnancy (antenatal record): "0=Nonreactive, 1=Reactive, 999=No test results recorded"
a2.mg$bltest_sypresults<-factor(a2.mg$bltest_sypresults, levels=c(0, 1), labels = c("No", "Yes"))
label(a2.mg$bltest_sypresults)<-"Diagnosed with syphilis in past 3 months"
covar<-append(covar, "bltest_sypresults")

# Abnormal vaginal discharge observed in past 3 months (self-report): "0=No, 1=Yes, 998=I don't know, 999=No Response"
a2.mg$ph_discharge<-factor(a2.mg$ph_discharge, levels=c(0, 1), labels=c("No", "Yes"))
label(a2.mg$ph_discharge)<-"Abnormal vaginal discharge observed in past 3 months (self-report)"
covar<-append(covar, "ph_discharge")

# Genital ulcers observed in past 3 months (self-report): "0=No, 1=Yes, 998=I don't know, 999=No Response"
a2.mg$ph_sores_ulcers<-factor(a2.mg$ph_sores_ulcers, levels=c(0, 1), labels=c("No", "Yes"))
label(a2.mg$ph_sores_ulcers)<-"Genital ulcers observed in past 3 months (self-report)"
covar<-append(covar, "ph_sores_ulcers")

# Running water in home: "0=No, 1=Yes"
a2.mg$water<-factor(a2.mg$water, levels=c(0, 1), labels=c("No", "Yes"))
label(a2.mg$water)<-"Running water in home"
covar<-append(covar, "water")

# Pregnancy Outcome
a2.mg$preg_outcome_clean<-factor(ifelse(grepl("Preterm Delivery", a2.mg$preg_outcome), "Preterm Delivery", ifelse(grepl("Term Delivery", a2.mg$preg_outcome), "Term Delivery", NA)), levels=c("Term Delivery", "Preterm Delivery"))
a2.mg$preg_outcome_clean<-relevel(a2.mg$preg_outcome_clean, ref="Term Delivery")
label(a2.mg$preg_outcome_clean)<-"Pregnancy Outcome"

label(a2.mg$date_diff)<-"Days between M0 and M1"

covars<-paste(covar, collapse=" + ")
```

## Table1 (at enrollment)


```
a2.enroll<-unique(a2.mg[a2.mg$swab %in% "1", !names(a2.mg) %in% c("Spec_Date", "maryland_ID", "mgCST2", "ART_PREP_date", "CST", "swab")])

# FIXED EFFECTS - to reduce the chances that an HIV/microbiome association is confounded by something else.
label(a2.enroll$sd_age)<-"Age at enrollment (years)"

## ph_ega: estimated gestational age at enrollment: Continuous 
label(a2.enroll$ph_ega)<-"Gestational age at enrollment (weeks)"

# Gravidity: "0=No prior pregnancies, 1=At least one prior pregnancy"
label(a2.enroll$gravidity)<-"Gravidity"

# Primary partner HIV status: 0=HIV-negative, 1=HIV-positive, 2=Indeterminate, 3=Partner never tested for HIV, 998=I don't know, 999=No response
label(a2.enroll$p1_status)<-"Primary partner HIV serostatus *"


# Number of partners in past three months: "0=No partners, 1=One partner, 2=Multiple partners"
label(a2.enroll$curr_ptnrs)<-"Number of partners in past three months"

#FIX a2.enroll$condom_freq_cat ... try grouping sometimes with never - how does this change?
#Consistent condom use with primary partner in past 30 days: "0=Never, 1=Sometimes, 2=Consistent"
label(a2.enroll$condom_freq_cat_f)<-"Consistent condom use with primary partner in past 30 days ^"

## ph_syphilis_told: self-report syphilis past 3 months. CAT
# Results of syphilis test during index pregnancy (antenatal record): "0=Nonreactive, 1=Reactive, 999=No test results recorded"
label(a2.enroll$bltest_sypresults)<-"Diagnosed with syphilis in past 3 months"

# Abnormal vaginal discharge observed in past 3 months (self-report): "0=No, 1=Yes, 998=I don't know, 999=No Response"
label(a2.enroll$ph_discharge)<-"Abnormal vaginal discharge observed in past 3 months (self-report)"

# Genital ulcers observed in past 3 months (self-report): "0=No, 1=Yes, 998=I don't know, 999=No Response"
label(a2.enroll$ph_sores_ulcers)<-"Genital ulcers observed in past 3 months (self-report)"

# Running water in home: "0=No, 1=Yes"
label(a2.enroll$water)<-"Running water in home"

label(a2.enroll$preg_outcome_clean)<-"Pregnancy Outcome"

label(a2.enroll$date_diff)<-"Days between M0 and M1"

table1(~ sd_age + ph_ega + gravidity + p1_status + curr_ptnrs + condom_freq_cat_f + bltest_sypresults + ph_discharge + ph_sores_ulcers + water + preg_outcome_clean + date_diff | hiv, data=a2.enroll, overall=F, extra.col=list(`P-value`=pvalue))
```


```
Warning in chisq.test(table(y, g)) :
  Chi-squared approximation may be incorrect
Warning in chisq.test(table(y, g)) :
  Chi-squared approximation may be incorrect
Warning in chisq.test(table(y, g)) :
  Chi-squared approximation may be incorrect
Warning in chisq.test(table(y, g)) :
  Chi-squared approximation may be incorrect
```


|  | WLHIV (N=49) | HIV-Negative (N=132) | P-value |
| --- | --- | --- | --- |
| Age at enrollment (years) |  |  |  |
| Mean (SD) | 27.4 (6.19) | 25.4 (5.40) | 0.05 |
| Median [Min, Max] | 26.0 [18.0, 42.0] | 25.0 [18.0, 40.0] |  |
| Gestational age at enrollment (weeks) |  |  |  |
| Mean (SD) | 22.3 (5.59) | 22.8 (7.48) | 0.61 |
| Median [Min, Max] | 22.0 [10.0, 36.0] | 23.0 [9.00, 41.0] |  |
| Gravidity |  |  |  |
| No prior pregnancies | 11 (22.4%) | 31 (23.5%) | 1 |
| At least one prior pregnancy | 38 (77.6%) | 101 (76.5%) |  |
| Primary partner HIV serostatus \* |  |  |  |
| HIV-negative | 9 (18.4%) | 96 (72.7%) | <0.01 |
| HIV-positive | 8 (16.3%) | 7 (5.3%) |  |
| Don't know | 29 (59.2%) | 27 (20.5%) |  |
| Missing | 3 (6.1%) | 2 (1.5%) |  |
| Number of partners in past three months |  |  |  |
| 0 | 3 (6.1%) | 2 (1.5%) | 0.14 |
| 1 | 46 (93.9%) | 127 (96.2%) |  |
| Multiple | 0 (0%) | 3 (2.3%) |  |
| Consistent condom use with primary partner in past 30 days ^ |  |  |  |
| Abstained | 9 (18.4%) | 17 (12.9%) | 0.39 |
| Never or Sometimes | 40 (81.6%) | 112 (84.8%) |  |
| Consistent | 0 (0%) | 3 (2.3%) |  |
| Diagnosed with syphilis in past 3 months |  |  |  |
| No | 47 (95.9%) | 73 (55.3%) | <0.01 |
| Yes | 2 (4.1%) | 57 (43.2%) |  |
| Missing | 0 (0%) | 2 (1.5%) |  |
| Abnormal vaginal discharge observed in past 3 months (self-report) |  |  |  |
| No | 47 (95.9%) | 78 (59.1%) | <0.01 |
| Yes | 2 (4.1%) | 54 (40.9%) |  |
| Genital ulcers observed in past 3 months (self-report) |  |  |  |
| No | 47 (95.9%) | 111 (84.1%) | 0.06 |
| Yes | 2 (4.1%) | 21 (15.9%) |  |
| Running water in home |  |  |  |
| No | 36 (73.5%) | 91 (68.9%) | 0.68 |
| Yes | 13 (26.5%) | 41 (31.1%) |  |
| Pregnancy Outcome |  |  |  |
| Term Delivery | 44 (89.8%) | 121 (91.7%) | 0.73 |
| Preterm Delivery | 4 (8.2%) | 7 (5.3%) |  |
| Missing | 1 (2.0%) | 4 (3.0%) |  |
| Days between M0 and M1 |  |  |  |
| Mean (SD) | 28.2 (2.85) | 29.6 (5.36) | 0.02 |
| Median [Min, Max] | 28.0 [19.0, 40.0] | 29.0 [18.0, 73.0] |  |

## Alpha Diversity & ART vs PrEP


```
counts<-as.data.frame(reshape2::dcast(counts.m, maryland_ID~taxa2, value.var="counts", fun.aggregate = sum))
rownames(counts)<-counts$maryland_ID
counts$maryland_ID<-NULL
exp<-phyloseq(otu_table(round(counts[rownames(counts) %in% a2.mg$maryland_ID, ]), taxa_are_rows = F))
counts$maryland_ID<-rownames(counts)
rownames(a2.mg)<-a2.mg$maryland_ID
exp<-merge_phyloseq(exp, sample_data(a2.mg))
rich<-estimate_richness( exp, measures = c("Chao1", "Shannon"))
rich$maryland_ID<-rownames(rich)

a2.mg.rich<-merge(a2.mg, rich, all=TRUE)

a2.mg<-a2.mg.rich[,c("PID", "maryland_ID", covar, "Shannon", "swab", "hiv", "treatment", "CST", "date_diff")]
#a2.mg<-a2.mg[complete.cases(a2.mg), ] ## lost 14 samples do to incomplete cases.
```


### - Diversity by swab and treatment


```
# In ART: only curr_ptnrs=1
the.model1<-lmer(formula = Shannon ~ swab + sd_age + ph_ega + gravidity + p1_status + bltest_sypresults + ph_discharge + ph_sores_ulcers + water + date_diff + (1|PID), data = a2.mg[a2.mg$treatment %in% "ART", ])
anova(the.model1)
```


```
Type III Analysis of Variance Table with Satterthwaite's method
                   Sum Sq Mean Sq NumDF DenDF F value   Pr(>F)   
swab              1.55542 1.55542     1    45  8.2775 0.006117 **
sd_age            0.30520 0.30520     1    35  1.6242 0.210905   
ph_ega            0.16339 0.16339     1    35  0.8695 0.357474   
gravidity         0.14530 0.14530     1    35  0.7732 0.385220   
p1_status         0.04481 0.02240     2    35  0.1192 0.887961   
bltest_sypresults 0.85587 0.85587     1    35  4.5547 0.039912 * 
ph_discharge      1.21714 1.21714     1    35  6.4772 0.015491 * 
ph_sores_ulcers   0.88250 0.88250     1    35  4.6964 0.037117 * 
water             0.24178 0.24178     1    35  1.2867 0.264368   
date_diff         0.90590 0.90590     1    35  4.8209 0.034836 * 
---
Signif. codes:  0 ‘***’ 0.001 ‘**’ 0.01 ‘*’ 0.05 ‘.’ 0.1 ‘ ’ 1
```


```
pvalue1<-summary(the.model1)$coefficients[, "Pr(>|t|)"]["swab2"]

the.model2<-lmer(formula = Shannon ~ swab + sd_age + ph_ega + gravidity + p1_status + bltest_sypresults + ph_discharge + ph_sores_ulcers + water + date_diff + (1|PID), data = a2.mg[a2.mg$treatment %in% "PrEP", ])
anova(the.model2)
```


```
Type III Analysis of Variance Table with Satterthwaite's method
                  Sum Sq Mean Sq NumDF DenDF F value    Pr(>F)    
swab              6.5785  6.5785     1   127 17.2140 6.071e-05 ***
sd_age            0.0753  0.0753     1   117  0.1972   0.65785    
ph_ega            0.4094  0.4094     1   117  1.0713   0.30280    
gravidity         1.9411  1.9411     1   117  5.0793   0.02608 *  
p1_status         0.3270  0.1635     2   117  0.4279   0.65289    
bltest_sypresults 2.2938  2.2938     1   117  6.0022   0.01577 *  
ph_discharge      0.1886  0.1886     1   117  0.4936   0.48370    
ph_sores_ulcers   0.9603  0.9603     1   117  2.5128   0.11562    
water             0.0005  0.0005     1   117  0.0014   0.97004    
date_diff         0.0543  0.0543     1   117  0.1422   0.70682    
---
Signif. codes:  0 ‘***’ 0.001 ‘**’ 0.01 ‘*’ 0.05 ‘.’ 0.1 ‘ ’ 1
```


```
pvalue2<-summary(the.model2)$coefficients[, "Pr(>|t|)"]["swab2"]

pvals_df <- data.frame(
  treatment = c("ART", "PrEP"),
  p_value = c("**", "***")
)
```

### FIG 5: Shannon by swab


```
## Individuals which initiate ART are less likely to experience an increase in alpha-diversity relative to those that initial PrEP.
cmp<-list(c("M0", "M1"))
to.plot<-a2.mg
to.plot$swab<-factor(ifelse(to.plot$swab %in% "1", "M0", "M1"))

segments_df <- data.frame(
  treatment_group = c("ART", "PrEP"),
  x = c(1, 1),
  xend = c(2, 2),
  y = 3,
  yend = 3
)

p1<-ggplot(to.plot[to.plot$treatment %in% "ART", ], aes(x=swab, y=Shannon))+
  geom_boxplot(color="black", lwd=0.1, outlier.shape = NA, notch = T, aes(fill=treatment))+
  geom_point(position=position_jitterdodge(), size=0.05, aes(fill=treatment))+
  scale_fill_manual(values=c(treat.cols[1]), label=c("ART", "PrEP"), name="")+
  theme_bw()+
  theme(legend.position = "none", 
        text=element_text(size=5, color="black"), 
        legend.key.size = unit(0.3, "cm"), 
        line = element_line(linewidth = 0.1), 
        axis.title = element_text(size=5), 
        axis.text = element_text(size=6, color="black"))+
  ylab("Shannon Diversity")+
  xlab("")+
  geom_text(data = pvals_df[1,], aes(x = 1.5, y = 3.1, label = p_value, hjust = 0.5))+
  geom_segment(data = segments_df[1,], aes(x = x, xend = xend, y = y, yend = yend), linewidth = 0.25)

p2<-ggplot(to.plot[to.plot$treatment %in% "PrEP", ], aes(x=swab, y=Shannon))+
  geom_boxplot(color="black", lwd=0.1, outlier.shape = NA, notch = T, aes(fill=treatment))+
  geom_point(position=position_jitterdodge(), size=0.05, aes(fill=treatment))+
  scale_fill_manual(values=c(treat.cols[2]), label=c("ART", "PrEP"), name="")+
  theme_bw()+
  theme(legend.position = "none", 
        text=element_text(size=5, color="black"), 
        legend.key.size = unit(0.3, "cm"), 
        line = element_line(linewidth = 0.1), 
        axis.title = element_text(size=5), 
        axis.text = element_text(size=6, color="black"))+
  ylab("Shannon Diversity")+
  xlab("")+
  geom_text(data = pvals_df[2,], aes(x = 1.5, y = 3.1, label = p_value, hjust = 0.5))+
  geom_segment(data = segments_df[2,], aes(x = x, xend = xend, y = y, yend = yend), linewidth = 0.25)

top_row = ggarrange(p1, p2, ncol = 2, labels = c("A.", "B."), font.label = list(face="plain", size=8), widths = c(0.5, 0.5))
tiff(paste("Manuscript_FIGURES/Figure_5_", today2, ".tiff", sep=""), height = 1800, width=2400, res=600)
top_row
dev.off()
```


```
null device 
          1
```

### - CST Transition and ARV


```
a2.mg.df<-reshape2::dcast(a2.mg, PID+treatment+date_diff~swab, value.var = "CST")
a2.mg.df$`1`<-gsub("IV-A", "IV", a2.mg.df$`1`)
a2.mg.df$`1`<-gsub("IV-B", "IV", a2.mg.df$`1`)
a2.mg.df$`1`<-gsub("IV-C", "IV", a2.mg.df$`1`)
a2.mg.df$`2`<-gsub("IV-A", "IV", a2.mg.df$`2`)
a2.mg.df$`2`<-gsub("IV-B", "IV", a2.mg.df$`2`)
a2.mg.df$`2`<-gsub("IV-C", "IV", a2.mg.df$`2`)
a2.mg.df$transition<-factor(ifelse(a2.mg.df$`1` == a2.mg.df$`2`, 0, 1), levels=c(0, 1))
a2.mg.df$transition_det<-factor(ifelse(a2.mg.df$transition == 0, "No Change", paste(a2.mg.df$`1`, "↓", a2.mg.df$`2`, sep="\n")), levels=c("No Change", "I\n↓\nIII", "I\n↓\nIV", "III\n↓\nI", "III\n↓\nIV", "III\n↓\nV", "IV\n↓\nI", "IV\n↓\nIII", "V\n↓\nIV"))

## Mean diff in shannon after ARV
shannon<-reshape2::dcast(a2.mg, PID~swab, value.var = "Shannon")
shannon$shannon_diff<-shannon$`2`-shannon$`1`
names(shannon)[2:3]<-c("Baseline_shannon", "AfterARV_shannon")
a2.mg.df$Shannon<-NULL
a2.mg.df<-merge(a2.mg.df, shannon, all=TRUE)
a2.mg.df %>% group_by(treatment) %>% summarise(meanShannondiff=mean(shannon_diff))
```


```
## Add in covariates at enrollment
a2.mg.df<-merge(a2.mg.df, a2.mg[a2.mg$swab %in% "1", ], all.x=TRUE)

the.model<-glm(transition~treatment + sd_age + ph_ega + gravidity + p1_status + bltest_sypresults + ph_discharge + ph_sores_ulcers + water + date_diff, data = a2.mg.df, family = "binomial")
c<-as.data.frame(broom::tidy(the.model, conf.int = T, exponentiate = T))
# people that change csts are less likely to have syph at enrollment and less likely to have water
```

### FIG 6: PRINT


```
to.plot<-a2.mg.df %>% group_by(treatment, transition_det) %>% summarise(n=length(unique(PID)))
```


```
`summarise()` has grouped output by 'treatment'. You can override using the `.groups`
argument.
```


```
to.plot[to.plot$treatment %in% "ART", "prop"]<-to.plot[to.plot$treatment %in% "ART", "n"]/49
to.plot[to.plot$treatment %in% "PrEP", "prop"]<-to.plot[to.plot$treatment %in% "PrEP", "n"]/132

levels(to.plot$treatment) <- c("ART, n = 39", "PrEP, n = 84")
p1<-ggplot(to.plot[to.plot$transition_det %in% "No Change", ], aes(x=treatment, y=prop, fill=treatment))+
  geom_bar(stat="identity", position="dodge", width=0.7, color="black", lwd=0.1)+
  scale_fill_manual(values=treat.cols, name="")+
  theme_bw()+
  theme(legend.position = "none", 
        text=element_text(size=5, color="black"), 
        legend.key.size = unit(0.3, "cm"), 
        line = element_line(linewidth = 0.1), 
        axis.title = element_text(size=5), 
        axis.text = element_text(color="black"))+
  ylab("Proportion of Samples in ARV Group")+
  xlab("")

levels(to.plot$treatment) <- c("ART, n = 10", "PrEP, n = 48")
p2<-ggplot(to.plot[!to.plot$transition_det %in% "No Change", ], aes(x=transition_det, y=prop, fill=treatment, group=treatment))+
  geom_bar(stat="identity", position="dodge", width=0.7, color="black", lwd=0.1)+
  scale_fill_manual(values=treat.cols, name="")+
  theme_bw()+
  theme(legend.position = "none", 
        text=element_text(size=5, color="black"), 
        legend.key.size = unit(0.3, "cm"), 
        line = element_line(linewidth = 0.1), 
        axis.title = element_text(size=5), 
        axis.text = element_text(color="black"))+
  ylab("Proportion of Samples in ARV Group")+
  xlab("")+
  facet_wrap(~treatment, nrow = 2)
#ggsave(paste("Analysis_Figures/Aim2_treatment_prevalence_by_cst_transition_type_", today2, ".png", sep=""), height = 3, width=3, dpi = 1200)

##### FIG 6: PRINT
top_row = ggarrange(p1, p2, ncol = 2, labels = c("A.", "B."), font.label = list(face="plain", size=8))
tiff(paste("Manuscript_FIGURES/Figure_6_", today2, ".tiff", sep=""), height = 1800, width=2400, res=600)
top_row
dev.off()
```


```
null device 
          1
```

## Taxa specific analysis, before vs. after by treatment


```
counts<-dcast(counts.m.temp[counts.m.temp$maryland_ID %in% sample.list, ], formula = maryland_ID~taxa, value.var = "counts", fun.aggregate = sum)

## Remove all taxa found in zymo positive controls
mgss0<-names(counts)[grepl("0$", names(counts))]
counts<-counts[,!names(counts) %in% c("Enterococcus_faecalis", "Escherichia_coli", "Lactobacillus_fermentum", "Salmonella_enterica", "Staphylococcus_aureus", "Candidatus_Pelagibacter", mgss0, to.rm)]

rownames(counts)<-counts$maryland_ID
counts$maryland_ID<-NULL
counts.all<-counts

taxa.all.results.long<-data.frame()
for(i in c("ART", "PrEP")){
  pid<-a2.mg.df[a2.mg.df$treatment %in% i , "PID"]
  samples<-a2.mg[a2.mg$PID %in% pid, "maryland_ID"]
  counts<-counts.all[rownames(counts.all) %in% samples, ]
  names(counts)<-gsub("_\\.", " ", names(counts))
  names(counts)<-gsub("_", " ", names(counts))
  names(counts)<-gsub(pattern = "\\.\\.", replacement = " ", names(counts))
  names(counts)<-gsub(pattern = "\\.", replacement = " ", names(counts))
  counts$maryland_ID<-rownames(counts)

  d.m<-reshape2::melt(counts, id.vars = "maryland_ID", variable.name = "Taxon", value.name = "count", na.rm = T)
  d.m<-merge(d.m, a2.mg[,c("maryland_ID", "swab")], all.x=TRUE)
  d.m$swab<-factor(ifelse(d.m$swab %in% "1", "M0", "M1"))
  samples.x.taxon<-d.m[d.m$count > 0, ] %>% dplyr::group_by(Taxon, swab) %>% dplyr::summarise(nSamples=length(unique(maryland_ID)))

  samples.x.taxon.df<-reshape2::dcast(samples.x.taxon, Taxon~swab, value.var = "nSamples")
  samples.x.taxon.df$`NA`<-NULL
  samples.x.taxon.df[is.na(samples.x.taxon.df)]<-0
  samples.x.taxon.df$Total<-samples.x.taxon.df$`M0`+samples.x.taxon.df$`M1`

  ## Test only those taxa which are present in at least 5% of samples =  min. 8 samples = 107 taxa
  keep<-as.vector(samples.x.taxon.df[samples.x.taxon.df$Total >= 0.05*nrow(counts), "Taxon"])
  c<-reshape2::dcast(d.m[d.m$Taxon %in% keep, ], Taxon~maryland_ID, value.var = "count", fun.aggregate = sum)
  rownames(c)<-c$Taxon
  c<-c[-1]
  c<-c[, colSums(c) > 0]
  c<-c[rowSums(c) > 0,  ]

  meta.sym<-a2.mg[,c("maryland_ID", "swab", covar, "date_diff")]
  meta.sym$swab<-factor(ifelse(meta.sym$swab %in% "1", "M0", "M1"))
  meta.sym$curr_ptnrs<-NULL
  meta.sym$swab<-factor(meta.sym$swab)
  meta.sym$swab<-relevel(meta.sym$swab, ref="M0")
  meta.sym<-meta.sym[meta.sym$maryland_ID %in% names(c), ]
  meta.sym<-meta.sym[complete.cases(meta.sym), ]
  c<-c[,colnames(c) %in% meta.sym$maryland_ID]
  
  obj.sym<-DESeqDataSetFromMatrix(countData = round(c), colData = meta.sym, design=~sd_age + ph_ega + gravidity + p1_status + bltest_sypresults + ph_discharge + ph_sores_ulcers + water + date_diff + swab)

  obj.sym = estimateSizeFactors( obj.sym, type="poscounts" )
  obj.sym = estimateDispersions( obj.sym, fitType="local" )

  ## Try limma + voom
  swab<-make.names(meta.sym$swab)
  sd_age<-meta.sym$sd_age
  ph_ega<-meta.sym$ph_ega
  date_diff<-meta.sym$date_diff
  gravidity<-make.names(meta.sym$gravidity)
  p1_status<-make.names(meta.sym$p1_status)
  bltest_sypresults<-make.names(meta.sym$bltest_sypresults)
  ph_discharge<-make.names(meta.sym$ph_discharge)
  ph_sores_ulcers<-make.names(meta.sym$ph_sores_ulcers)
  water<-make.names(meta.sym$water)
  
  design<-model.matrix(~sd_age + ph_ega + gravidity + p1_status + bltest_sypresults + ph_discharge + ph_sores_ulcers + water + date_diff + swab)
  
  nc <- counts(obj.sym, normalized=TRUE)
  PID<-a2.mg[a2.mg$maryland_ID %in% colnames(nc), c("PID")]
  d0 <- DGEList(nc)
  d0 <- calcNormFactors(d0)
  v<-voom(d0, design, block=PID, plot=T)
  corfit<-duplicateCorrelation(v, design, block=PID)
  fit<-lmFit(v, design, block=PID, correlation = corfit$consensus.correlation)
  tmp <- eBayes(fit)
  top.table <- topTable(tmp, sort.by = "F", n = Inf)
  top.table$taxa<-rownames(top.table)
  top.table$treatment<-i
  taxa.all.results.long<-rbind(taxa.all.results.long, top.table)
}
```


```
`summarise()` has grouped output by 'Taxon'. You can override using the `.groups`
argument.
```


```
converting counts to integer mode
  the design formula contains one or more numeric variables with integer values,
  specifying a model with increasing fold change for higher values.
  did you mean for this to be a factor? if so, first convert
  this variable to a factor using the factor() function
  the design formula contains one or more numeric variables that have mean or
  standard deviation larger than 5 (an arbitrary threshold to trigger this message).
  Including numeric variables with large mean can induce collinearity with the intercept.
  Users should center and scale numeric variables in the design to improve GLM convergence.
  Note: levels of factors in the design contain characters other than
  letters, numbers, '_' and '.'. It is recommended (but not required) to use
  only letters, numbers, and delimiters '_' or '.', as these are safe characters
  for column names in R. [This is a message, not a warning or an error]
  Note: levels of factors in the design contain characters other than
  letters, numbers, '_' and '.'. It is recommended (but not required) to use
  only letters, numbers, and delimiters '_' or '.', as these are safe characters
  for column names in R. [This is a message, not a warning or an error]
gene-wise dispersion estimates
mean-dispersion relationship
  Note: levels of factors in the design contain characters other than
  letters, numbers, '_' and '.'. It is recommended (but not required) to use
  only letters, numbers, and delimiters '_' or '.', as these are safe characters
  for column names in R. [This is a message, not a warning or an error]
final dispersion estimates
Removing intercept from test coefficients
```


```
`summarise()` has grouped output by 'Taxon'. You can override using the `.groups`
argument.
```


```
converting counts to integer mode
  the design formula contains one or more numeric variables with integer values,
  specifying a model with increasing fold change for higher values.
  did you mean for this to be a factor? if so, first convert
  this variable to a factor using the factor() function
  the design formula contains one or more numeric variables that have mean or
  standard deviation larger than 5 (an arbitrary threshold to trigger this message).
  Including numeric variables with large mean can induce collinearity with the intercept.
  Users should center and scale numeric variables in the design to improve GLM convergence.
  Note: levels of factors in the design contain characters other than
  letters, numbers, '_' and '.'. It is recommended (but not required) to use
  only letters, numbers, and delimiters '_' or '.', as these are safe characters
  for column names in R. [This is a message, not a warning or an error]
  Note: levels of factors in the design contain characters other than
  letters, numbers, '_' and '.'. It is recommended (but not required) to use
  only letters, numbers, and delimiters '_' or '.', as these are safe characters
  for column names in R. [This is a message, not a warning or an error]
gene-wise dispersion estimates
mean-dispersion relationship
  Note: levels of factors in the design contain characters other than
  letters, numbers, '_' and '.'. It is recommended (but not required) to use
  only letters, numbers, and delimiters '_' or '.', as these are safe characters
  for column names in R. [This is a message, not a warning or an error]
final dispersion estimates
```


```
Removing intercept from test coefficients
```


```
taxa.all.results.long<-unique(taxa.all.results.long)
taxa.all.results.long$association<-ifelse(taxa.all.results.long$swabM1 < 0, "Negative", "Positive")
order.tax<-taxa.all.results.long[order(taxa.all.results.long$swabM1, decreasing = T), "taxa"]
taxa.all.results.long$taxa<-factor(taxa.all.results.long$taxa)

taxa.all.results.long$treatment<-factor(taxa.all.results.long$treatment, levels=c("ART", "PrEP"))
```


### FIG 7: PrEP / ART Taxa


```
## The reference was before, so white is increased after and black is decrease after ARV initiation. 
p2<-ggplot(taxa.all.results.long[taxa.all.results.long$adj.P.Val < 0.05 & !is.na(taxa.all.results.long$adj.P.Val) & taxa.all.results.long$treatment %in% "PrEP", ], aes(x=swabM1, y=reorder(taxa, swabM1), fill=association))+
  geom_bar(stat="identity", color="black", lwd=0.1)+
  xlab("Log-Fold Difference in mgSs Abundance\n(M1 / M0)")+
  scale_fill_manual(values=c("black", "white"), name="Taxa Associated with Treatment Initiation")+
  theme_bw()+
  theme(axis.title.x = element_text(size=5, color="black"), 
        axis.text.y = element_text(size=2.5, face="italic", color="black"), 
        axis.text.x=element_text(size=5, color="black"), 
        axis.ticks = element_line(linewidth = 0.2),
        strip.background = element_rect(fill=treat.cols[2]), 
        strip.text = element_text(color="white", face="bold", size=5), 
        legend.position = "none")+
  ylab("")+
  facet_wrap(~treatment) 
#ggsave(paste("Analysis_Figures/MLAWI_mgSs_treatment_change_PrEP_",today2, ".tiff", sep=""), height=4, width=4, dpi = 600)

p1<-ggplot(taxa.all.results.long[taxa.all.results.long$adj.P.Val < 0.05 & !is.na(taxa.all.results.long$adj.P.Val) & taxa.all.results.long$treatment %in% "ART", ], aes(x=swabM1, y=reorder(taxa, swabM1), fill=association))+
  geom_bar(stat="identity", color="black", lwd=0.1)+
  xlab("Log-Fold Difference in mgSs Abundance\n(M1 / M0)")+
  scale_fill_manual(values=c("black", "white"), name="Taxa Associated with Treatment Initiation")+
  theme_bw()+
  theme(axis.title.x = element_text(size=5, color="black"), 
        axis.text.y = element_text(size=3, face="italic", color="black"), 
        axis.text.x=element_text(size=5, color="black"),
        axis.ticks = element_line(linewidth = 0.2),
        strip.background = element_rect(fill=treat.cols[1]), 
        strip.text = element_text(color="black", face="bold", size=5), 
        legend.position = "none")+
  ylab("")+
  facet_wrap(~treatment)
#ggsave(paste("Analysis_Figures/MLAWI_mgSs_treatment_change_ART_",today2, ".tiff", sep=""), height=4, width=4, dpi = 600)
top_row = ggarrange(p1, p2, ncol = 2, labels = c("A.", "B."), font.label = list(face="plain", size=8), widths = c(0.5,0.5))
tiff(paste("Manuscript_FIGURES/Figure_7_", today2, ".tiff", sep=""), height = 1800, width=3600, res=600)
top_row
dev.off()
```


```
null device 
          1
```

## Comparison cohort (ZAPPS)

Subset the ZAPPs data to include the below information (separately
for HIV- and WLHIV) Gestational Age at Enrollment (weeks) WLHIV HIV(-)
6-20 19 (38.8%) 53 (40.2%) 21-30 27 (55.1%) 62 (47.0%) 31-41 3 (6.1%) 17
(12.9%)

Gestational Age at Enrollment (weeks) WLHIV HIV(-) Mean (SD) 22.3
(5.59) 22.8 (7.48) 0.606 Median [Min, Max] 22.0 [10.0, 36.0] 23.0 [9.00,
41.0]

Days between ARV initiation and 2nd sample WLHIV HIV(-) Mean (SD)
28.2 (2.85) 29.6 (5.36) Median [Min, Max] 28.0 [19.0, 40.0] 29.0 [18.0,
73.0]

Age at Enrollment (years) WLHIV HIV(-) 18-24 17 (34.7%) 65
(49.2%)

##### ZAPPS: Pick samples


```
zapps<-read_excel("ZAPPS/Combined_ZAPPS_PreSSMat_Metadata_FINAL_11162021_clean.xlsx", sheet = "ZAPPS_PreSSMat")
zapps<-unique(zapps)
csts<-read.csv("ZAPPS/2022_GATES_all_runs_dada2_abundance_table_StR_CST.csv")
csts$sampleID2<-gsub("\\.", "-", csts$sampleID)
zapps$subCST<-csts[match(zapps$specimenid, csts$sampleID2), "sim_subCST"]
zapps.cast.1<-reshape2::dcast(unique(zapps[!is.na(zapps$subCST), c("participant_id", "Visit", "subCST")]), participant_id~Visit, value.var = "subCST")
names(zapps.cast.1)[2:4]<-paste("CST_",names(zapps.cast.1)[2:4], sep="")
zapps.cast.2<-reshape2::dcast(unique(zapps[!is.na(zapps$subCST), c("participant_id", "Visit", "subCST", "ega_days")]), participant_id~Visit, value.var = "ega_days")
names(zapps.cast.2)[2:4]<-paste("ega_days_",names(zapps.cast.2)[2:4], sep="")
zapps.cast.3<-reshape2::dcast(unique(zapps[!is.na(zapps$subCST), c("participant_id", "Visit", "subCST", "ega_weeks")]), participant_id~Visit, value.var = "ega_weeks")
names(zapps.cast.3)[2:4]<-paste("ega_weeks_",names(zapps.cast.3)[2:4], sep="")
zapps.cast.4<-reshape2::dcast(unique(zapps[!is.na(zapps$subCST), c("participant_id", "Visit", "subCST", "ega_weeks", "case")]), participant_id~Visit, value.var = "case")
names(zapps.cast.4)[2:4]<-paste("case_",names(zapps.cast.4)[2:4], sep="")
zapps.cast<-merge(zapps.cast.1, zapps.cast.2, all=TRUE)
zapps.cast<-merge(zapps.cast, zapps.cast.3, all=TRUE)
zapps.cast<-merge(zapps.cast, zapps.cast.4, all=TRUE)
zapps.cast$ega_day_EV1_diff<-zapps.cast$ega_days_Visit_1-zapps.cast$ega_days_Enrollment
zapps.cast$ega_day_V1V2_diff<-zapps.cast$ega_days_Visit_2-zapps.cast$ega_days_Visit_1
zapps.cast$case_pattern<-paste(zapps.cast$case_Enrollment, zapps.cast$case_Visit_1, zapps.cast$case_Visit_2, sep="")

zapps.meta<-unique(read.csv("ZAPPS/processed_clinical_data_GAPPS_subset_12dec2023.csv")) ## extra metadata for proper modeling with similar covariates to MLAWI:
zapps.meta$visnum<-str_split_fixed(make.unique(zapps.meta$ORIG_ID, sep = "_"), "_", 2)[,2]
zapps.meta$visnum[zapps.meta$visnum %in% ""]<-"Enrollment"
# sd_age.cat -- PW_AGE
# + preg_outcome -- GAGEBRTH (weeks) or ERLY_PTB
# + ph_ega.cat -- EGA_
# + bltest_sypresults RPR
# + sex_freq.cat 
# + condom_freq_cat_f 
# + p1_status_f 
# + curr_ptnrs 
# + gravidity -- GRAVIDITY
# + ph_discharge 
# + water -- DRINKING SOURCE == 1 or 2
# + ph_sores_ulcers -- 
# + electric -- HH_ELECTRICITY
# + educat -- PW_EDUCATION
zapps.meta$ORIG_ID_GA<-paste(zapps.meta$ORIG_ID, zapps.meta$GA, sep="_")
baseline.samples<-vector()
for(i in unique(zapps.meta$ORIG_ID)){
  s<-zapps.meta[zapps.meta$ORIG_ID %in% i, c("GA", "ORIG_ID_GA")]
  baseline.samples<-append(baseline.samples, s[s$GA == min(s$GA), "ORIG_ID_GA"])
}
zapps.meta.baseline<-zapps.meta[zapps.meta$ORIG_ID_GA %in% baseline.samples, c("ORIG_ID", "PW_AGE", "GA", "GAGEBRTH", "SYPHILIS", "GRAVIDITY", "DRINKING_SOURCE", "ERLY_PTB", "PREG_OUTCOME", "HIV", "PTB")]

zapps.meta.baseline$PID<-zapps.meta.baseline$ORIG_ID
#zapps.meta.baseline$sd_age.cat<-factor(ifelse(zapps.meta.baseline$PW_AGE %in% 18:24, "18-24", ifelse(zapps.meta.baseline$PW_AGE %in% 25:30, "25-30", ifelse(zapps.meta.baseline$PW_AGE < 18, NA, "31-42"))), ordered = F)
zapps.meta.baseline$sd_age<-zapps.meta.baseline$PW_AGE

#GRAVIDITY: Including this pregnancy, how many times in your life have you been pregnant?
zapps.meta.baseline$gravidity<-factor(ifelse(zapps.meta.baseline$GRAVIDITY > 0, "At least one prior pregnancy", "No prior pregnancies"))

#DRINKING_SOURCE: 1=Piped/Bottle Water/Tanker Truck, 2=Tubewell(anytype), 3=Rain Water, 4=Surface Water(River,Pond,etc.)
zapps.meta.baseline$water<-factor(ifelse(zapps.meta.baseline$DRINKING_SOURCE %in% c(1, 2), "Yes", "No"))

#SYPHILIS: Syphilis testing at enrollment: 0 = non-reactive/negative; 1 = reactive/positive; 2 = indeterminate 
zapps.meta.baseline$bltest_sypresults<-factor(ifelse(zapps.meta.baseline$SYPHILIS %in% 0, "No", "Yes"))

zapps.cast<-merge(zapps.cast, zapps.meta.baseline, all=TRUE, by.x="participant_id", by.y="ORIG_ID")
```

#### Get HIV neg cases which match the above criteria as best we can.


```
### MLAWI
a2.mg.df.hivneg<-a2.mg.df[a2.mg.df$hiv %in% "HIV-Negative", c("PID", "1", "2", "sd_age", "ph_ega", "bltest_sypresults", "gravidity", "water", "date_diff")]
a2.mg.df.hivneg$study<-"Tonse Pamodzi 2\n(PrEP-exposed)"
a2.mg.df.hivneg$exposure<-"PrEP"

### ZAPPS
## GET HIV-NEG SUBJECTS WITH SIMILAR TIME BETWEEN VISITS
ev1.subj<-zapps.cast[zapps.cast$case_pattern %in% "000" & zapps.cast$ega_day_EV1_diff <= 40, "participant_id"] # n=106 Enroll->V1
v1v2.subj<-zapps.cast[zapps.cast$case_pattern %in% "000" & zapps.cast$ega_day_V1V2_diff <= 40, "participant_id"] # n=121 V1->V2
v1v2.subj<-v1v2.subj[!v1v2.subj %in% ev1.subj] ## remove any already chosen for enrollment->V1

zapps.hiv.neg<-zapps.cast[zapps.cast$participant_id %in% unique(c(ev1.subj, v1v2.subj)), ] #n=256
### 

### CAPTURE GESTATIONAL AGE OF SAMPLE DEPENDING ON IF ENROLL->V1 OR V1->V2
zapps.hiv.neg$ega_weeks<-as.numeric(ifelse(zapps.hiv.neg$participant_id %in% ev1.subj, 
                                           as.character(zapps.hiv.neg$ega_weeks_Enrollment), 
                                           ifelse(zapps.hiv.neg$participant_id %in% v1v2.subj, 
                                                  as.character(zapps.hiv.neg$ega_weeks_Visit_1), NA)))
  #   Min. 1st Qu.  Median    Mean 3rd Qu.    Max. 
  # 17.10   19.50   27.60   25.48   28.20   30.00 ## majority in the second trimester, some in 1st.
zapps.hiv.neg$ph_ega<-zapps.hiv.neg$ega_weeks

zapps.hiv.neg$date_diff<-as.numeric(ifelse(zapps.hiv.neg$participant_id %in% ev1.subj, as.character(zapps.hiv.neg$ega_day_EV1_diff), ifelse(zapps.hiv.neg$participant_id %in% v1v2.subj, as.character(zapps.hiv.neg$ega_day_V1V2_diff), NA)))

zapps.hiv.neg.samples.to.get<-c(paste(ev1.subj, "Enrollment", sep = "_"), paste(ev1.subj, "Visit_1", sep = "_"), paste(v1v2.subj, "Visit_1", sep = "_"), paste(v1v2.subj, "Visit_2", sep = "_"))
zapps$participant_id_Visit<-paste(zapps$participant_id, zapps$Visit, sep="_")

zapps.hiv.neg.cst<-zapps[zapps$participant_id_Visit %in% zapps.hiv.neg.samples.to.get, ]

### VISITS
zapps.hiv.neg.cst$visnum<-ifelse(zapps.hiv.neg.cst$participant_id %in% ev1.subj, ifelse(zapps.hiv.neg.cst$Visit %in% "Enrollment", 1, 2), NA)
zapps.hiv.neg.cst$visnum<-ifelse(zapps.hiv.neg.cst$participant_id %in% v1v2.subj, ifelse(zapps.hiv.neg.cst$Visit %in% "Visit_1", 1, 2), as.character(zapps.hiv.neg.cst$visnum))


zapps.hiv.neg.cast<-reshape2::dcast(zapps.hiv.neg.cst, participant_id~visnum, value.var = "subCST")
zapps.hiv.neg.cast$study<-"ZAPPS\n(Unexposed)"
zapps.hiv.neg.cast$exposure<-"No PrEP"
zapps.hiv.neg$preg_outcome<-ifelse(zapps.hiv.neg$PTB == 0, "Term Delivery", "Preterm Delivery")

zapps.hiv.neg.cast<-merge(zapps.hiv.neg.cast, zapps.hiv.neg, all.x=TRUE)
#+sd_age.cat+ph_ega.cat+bltest_sypresults + gravidity + electric + educat + water


### COMBINE ZAPPS AND TP2### 
cmp.transitions<-rbind(a2.mg.df.hivneg[, c("PID", "1", "2", "sd_age", "ph_ega", "bltest_sypresults", "gravidity", "water", "study", "exposure", "date_diff")], zapps.hiv.neg.cast[,c("PID", "1", "2", "sd_age", "ph_ega", "bltest_sypresults", "gravidity", "water", "study", "exposure", "date_diff")])
cmp.transitions<-cmp.transitions[complete.cases(cmp.transitions), ]
### TABLE 1### 
# FIXED EFFECTS - to reduce the chances that an HIV/microbiome association is confounded by something else.
label(cmp.transitions$sd_age)<-"Age at enrollment (years)"

## ph_ega: estimated gestational age at enrollment: Continuous 
label(cmp.transitions$ph_ega)<-"Gestational age at enrollment (weeks)"

# TP2: Gravidity: "0=No prior pregnancies, 1=At least one prior pregnancy"
# ZAPPS: Gravidity: "0=No prior pregnancies, 1=At least one prior pregnancy"
label(cmp.transitions$gravidity)<-"Gravidity"

## ph_syphilis_told: self-report syphilis past 3 months. CAT
# Results of syphilis test during index pregnancy (antenatal record): "0=Nonreactive, 1=Reactive, 999=No test results recorded"
label(cmp.transitions$bltest_sypresults)<-"Diagnosed with syphilis in past 3 months"

# Running water in home: "0=No, 1=Yes"
label(cmp.transitions$water)<-"Running water in home"

label(cmp.transitions$date_diff)<-"Days between M0 and M1"

table1(~ sd_age + ph_ega + gravidity + bltest_sypresults + water + date_diff | study, data=cmp.transitions, overall=F, extra.col=list(`P-value`=pvalue))
```


|  | Tonse Pamodzi 2 (PrEP-exposed) (N=130) | ZAPPS (Unexposed) (N=196) | P-value |
| --- | --- | --- | --- |
| Age at enrollment (years) |  |  |  |
| Mean (SD) | 25.5 (5.40) | 26.1 (5.21) | 0.27 |
| Median [Min, Max] | 25.0 [18.0, 40.0] | 26.0 [15.0, 40.0] |  |
| Gestational age at enrollment (weeks) |  |  |  |
| Mean (SD) | 22.8 (7.47) | 24.5 (4.66) | 0.03 |
| Median [Min, Max] | 23.0 [9.00, 41.0] | 27.5 [16.4, 30.0] |  |
| Gravidity |  |  |  |
| No prior pregnancies | 29 (22.3%) | 0 (0%) | <0.01 |
| At least one prior pregnancy | 101 (77.7%) | 196 (100%) |  |
| Diagnosed with syphilis in past 3 months |  |  |  |
| No | 73 (56.2%) | 127 (64.8%) | 0.15 |
| Yes | 57 (43.8%) | 69 (35.2%) |  |
| Running water in home |  |  |  |
| No | 89 (68.5%) | 2 (1.0%) | <0.01 |
| Yes | 41 (31.5%) | 194 (99.0%) |  |
| Days between M0 and M1 |  |  |  |
| Mean (SD) | 29.6 (5.40) | 30.2 (5.26) | 0.34 |
| Median [Min, Max] | 29.0 [18.0, 73.0] | 29.0 [19.0, 40.0] |  |


```
### ESTIMATE TRANSITION BY PREP EXPOSURE
cmp.transitions$exposure<-factor(cmp.transitions$exposure, levels=c("No PrEP", "PrEP"))

cmp.transitions$cst1<-cmp.transitions$`1`
cmp.transitions$cst1<-ifelse(cmp.transitions$`1` %in% c("I-A", "I-B"), "I", as.character(cmp.transitions$cst1)) 
cmp.transitions$cst1<-ifelse(cmp.transitions$`1` %in% c("III-A", "III-B"), "III", as.character(cmp.transitions$cst1)) 
cmp.transitions$cst1<-ifelse(cmp.transitions$`1` %in% c("IV-A", "IV-B", "IV-C", "IV-C0"), "IV", as.character(cmp.transitions$cst1)) 

cmp.transitions$cst2<-cmp.transitions$`2`
cmp.transitions$cst2<-ifelse(cmp.transitions$`2` %in% c("I-A", "I-B"), "I", as.character(cmp.transitions$cst2)) 
cmp.transitions$cst2<-ifelse(cmp.transitions$`2` %in% c("III-A", "III-B"), "III", as.character(cmp.transitions$cst2)) 
cmp.transitions$cst2<-ifelse(cmp.transitions$`2` %in% c("IV-A", "IV-B", "IV-C", "IV-C0"), "IV", as.character(cmp.transitions$cst2)) 

cmp.transitions$transition<-factor(ifelse(cmp.transitions$cst1 == cmp.transitions$cst2, "No Change", "Changed"), levels=c("No Change", "Changed"))
oddsratio(table(cmp.transitions$exposure, cmp.transitions$transition))
```


```
$data
         
          No Change Changed Total
  No PrEP       143      53   196
  PrEP           83      47   130
  Total         226     100   326

$measure
         odds ratio with 95% C.I.
          estimate     lower    upper
  No PrEP 1.000000        NA       NA
  PrEP    1.525842 0.9450522 2.463237

$p.value
         two-sided
          midp.exact fisher.exact chi.square
  No PrEP         NA           NA         NA
  PrEP    0.08372125    0.0870137 0.08062047

$correction
[1] FALSE

attr(,"method")
[1] "median-unbiased estimate & mid-p exact CI"
```


```
## Individuals exposed to prep were no more likely to transition CSTs than those unexposed. 
to.test<-cmp.transitions
to.test$outcomebin<-factor(ifelse(to.test$transition %in% "Changed", 1, 0))
the.model<-glm(outcomebin ~ exposure + sd_age + ph_ega + gravidity + bltest_sypresults + water + date_diff, data = to.test, family = "binomial") ## 21 samples removed due to missingness (mostly zapps)
as.data.frame(broom::tidy(the.model, conf.int = T, exponentiate = T))
```


```
label(cmp.transitions$transition)<-"CST transition between M0 and M1"
table1(~ sd_age + ph_ega + gravidity + bltest_sypresults + water + date_diff + transition| study, data=cmp.transitions, overall=F, extra.col=list(`P-value`=pvalue))
```


|  | Tonse Pamodzi 2 (PrEP-exposed) (N=130) | ZAPPS (Unexposed) (N=196) | P-value |
| --- | --- | --- | --- |
| Age at enrollment (years) |  |  |  |
| Mean (SD) | 25.5 (5.40) | 26.1 (5.21) | 0.27 |
| Median [Min, Max] | 25.0 [18.0, 40.0] | 26.0 [15.0, 40.0] |  |
| Gestational age at enrollment (weeks) |  |  |  |
| Mean (SD) | 22.8 (7.47) | 24.5 (4.66) | 0.03 |
| Median [Min, Max] | 23.0 [9.00, 41.0] | 27.5 [16.4, 30.0] |  |
| Gravidity |  |  |  |
| No prior pregnancies | 29 (22.3%) | 0 (0%) | <0.01 |
| At least one prior pregnancy | 101 (77.7%) | 196 (100%) |  |
| Diagnosed with syphilis in past 3 months |  |  |  |
| No | 73 (56.2%) | 127 (64.8%) | 0.15 |
| Yes | 57 (43.8%) | 69 (35.2%) |  |
| Running water in home |  |  |  |
| No | 89 (68.5%) | 2 (1.0%) | <0.01 |
| Yes | 41 (31.5%) | 194 (99.0%) |  |
| Days between M0 and M1 |  |  |  |
| Mean (SD) | 29.6 (5.40) | 30.2 (5.26) | 0.34 |
| Median [Min, Max] | 29.0 [18.0, 73.0] | 29.0 [19.0, 40.0] |  |
| CST transition between M0 and M1 |  |  |  |
| No Change | 83 (63.8%) | 143 (73.0%) | 0.1 |
| Changed | 47 (36.2%) | 53 (27.0%) |  |


```
cmp.transitions$transition_det<-factor(ifelse(cmp.transitions$transition %in% "No Change", "No Change", paste(cmp.transitions$cst1, cmp.transitions$cst2, sep="\n↓\n")), levels=c("No Change", "I\n↓\nII", "I\n↓\nIII", "I\n↓\nIV", "II\n↓\nI",  "II\n↓\nIII", "II\n↓\nIV", "III\n↓\nI","III\n↓\nII", "III\n↓\nIV", "III\n↓\nV", "IV\n↓\nI", "IV\n↓\nII","IV\n↓\nIII", "V\n↓\nI", "V\n↓\nIV"))
label(cmp.transitions$transition_det)<-"CSTs from M0 to M1"

## Specifically test the transition from CST III to IV between exposed and unexposed
to.test<-cmp.transitions[cmp.transitions$transition %in% "Changed", ]
to.test$outcomebin<-ifelse(to.test$cst2 %in% "IV", 1, 0)
the.model<-glm(outcomebin ~ exposure + sd_age + ph_ega + gravidity + bltest_sypresults + water + date_diff, data = to.test, family = "binomial") ## 21 samples removed due to missingness (mostly zapps)
as.data.frame(broom::tidy(the.model, conf.int = T, exponentiate = T))
```

# Aim 3, explore sPTB

Aim 3 (exploratory): To describe whether the vaginal microbiota
composition, structure, and changes differ between pregnant women who
have a spontaneous preterm birth (SPTB) compared to women who deliver at
term.

Analysis plan: Using the delivery outcomes, we will compare how the
vaginal microbiota composition and structure differ between the women
with SPTB and those that deliver at term. We defined preterm delivery as
delivery at less than 37 completed weeks of pregnancy. SPTB include
preterm labor, preterm spontaneous rupture of membranes, preterm
premature rupture of membranes (PPROM) and cervical weakness; we exclude
indicated preterm delivery for maternal or fetal conditions. We will use
clinical data from participant interview as well as the medical and
obstetric history, intrapartum management and birth outcomes that were
recorded for all participants. We will assess associations between each
of the measures noted in Aim 2 above and SPTB using logistic regression
in both univariable and multivariable models. We will account for
possible confounders: maternal age, parity, education and socioeconomic
status, nutrition, gestational age at the time of sample collection, HIV
viral load (For HIV+ women).

## Association between CST IV and PTB


```
meta<-read.csv("Feb_10_email/TP2_pregoutcome_needed.csv")
a3.mg<-a1.mg
a3.mg$preg_outcome<-meta$preg_outcome[match(a3.mg$maryland_ID, meta$maryland_ID)]
a3.mg$preg_outcome<-ifelse(is.na(a3.mg$preg_outcome), a2.mg.rich$preg_outcome[match(a3.mg$maryland_ID, a2.mg.rich$maryland_ID)], as.character(a3.mg$preg_outcome))
a3.mg$preg_outcome_clean<-factor(ifelse(grepl("Preterm Delivery", a3.mg$preg_outcome), "Preterm Delivery", ifelse(grepl("Term Delivery", a3.mg$preg_outcome), "Term Delivery", NA)), levels=c("Term Delivery", "Preterm Delivery"))
a3.mg$preg_outcome_clean<-relevel(a3.mg$preg_outcome_clean, ref="Term Delivery")
a3.mg<-a3.mg[!is.na(a3.mg$preg_outcome_clean), ]

### TEST HIV vs sPTB
a3.mg$hiv<-relevel(a3.mg$hiv, ref="HIV-Negative")
the.model<-glm(preg_outcome_clean ~ hiv + sd_age + ph_ega + gravidity + p1_status + bltest_sypresults + ph_discharge + ph_sores_ulcers + water, data = a3.mg, family = "binomial") ## 41 samples removed due to missingness (mostly zapps)
wlhiv.sptb<-as.data.frame(broom::tidy(the.model, conf.int = T, exponentiate = T))[2,]
table(a3.mg$hiv, a3.mg$preg_outcome_clean)
```


```
               Term Delivery Preterm Delivery
  HIV-Negative           159               10
  WLHIV                   48                7
```


```
### GET M0 CST
a3.mg$CST_M0<-a3.mg$CST
a3.mg$CST_M0<-gsub("IV-A", "IV", a3.mg$CST_M0)
a3.mg$CST_M0<-gsub("IV-B", "IV", a3.mg$CST_M0)
a3.mg$CST_M0<-gsub("IV-C", "IV", a3.mg$CST_M0)
a3.mg$CST_M0<-factor(a3.mg$CST_M0)
a3.mg$CSTIV_M0_exp<-factor(ifelse(grepl("IV", a3.mg$CST_M0), 1, 0))

### GET M1 CST
swab2<-a2.mg[a2.mg$swab %in% 2, ]
a3.mg$CST_M1<-swab2[match(a3.mg$pid, swab2$PID), "CST"]
a3.mg$CST_M1<-gsub("IV-A", "IV", a3.mg$CST_M1)
a3.mg$CST_M1<-gsub("IV-B", "IV", a3.mg$CST_M1)
a3.mg$CST_M1<-gsub("IV-C", "IV", a3.mg$CST_M1)
a3.mg$CST_M1<-factor(a3.mg$CST_M1)

### TEST CST IV AT M0 OR M1 AND sPTB
a3.mg$CSTIV_any<-factor(ifelse(a3.mg$CST_M0 %in% "IV" | a3.mg$CST_M1 %in% "IV", 1, 0))
the.model<-glm(preg_outcome_clean ~ CSTIV_any + sd_age + ph_ega + gravidity + p1_status + bltest_sypresults + ph_discharge + ph_sores_ulcers + water, data = a3.mg, family = "binomial") 
as.data.frame(broom::tidy(the.model, conf.int = T, exponentiate = T))
```


```
cstiv.m0.m1.sptb<-as.data.frame(broom::tidy(the.model, conf.int = T, exponentiate = T))[2,]
table(a3.mg[, "CSTIV_any"], a3.mg[, "preg_outcome_clean"])
```


```
    Term Delivery Preterm Delivery
  0            78                8
  1           129                9
```


```
### TEST ANY CHANGE AND sPTB
a3.mg<-a3.mg[!is.na(a3.mg$CST_M1), ]
a3.mg$transition<-factor(ifelse(!a3.mg$CST_M0 == a3.mg$CST_M1, 1, 0))
the.model<-glm(preg_outcome_clean ~ transition + sd_age + ph_ega + gravidity + p1_status + bltest_sypresults + ph_discharge + ph_sores_ulcers + water, data = a3.mg[!is.na(a3.mg$CST_M1), ], family = "binomial") 
change.cst.sptb<-as.data.frame(broom::tidy(the.model, conf.int = T, exponentiate = T))[2,]
table(a3.mg[!is.na(a3.mg$CST_M1), "transition"], a3.mg[!is.na(a3.mg$CST_M1), "preg_outcome_clean"])
```


```
    Term Delivery Preterm Delivery
  0           111                7
  1            54                4
```


```
### TEST TRANSITION TO CST IV AT M1 AND sPTB
a3.mg$CSTIV_trans_exp<-factor(ifelse(!grepl("IV", a3.mg$CST_M0) & grepl("IV", a3.mg$CST_M1), 1, 0))
the.model<-glm(preg_outcome_clean ~ CSTIV_trans_exp + sd_age + ph_ega + gravidity + p1_status + bltest_sypresults + ph_discharge + ph_sores_ulcers + water, data = a3.mg[!is.na(a3.mg$CST_M1), ], family = "binomial") 
change.to.cstiv.sptb<-as.data.frame(broom::tidy(the.model, conf.int = T, exponentiate = T))[2,]
table(a3.mg[!is.na(a3.mg$CST_M1), "CSTIV_trans_exp"], a3.mg[!is.na(a3.mg$CST_M1), "preg_outcome_clean"])
```


```
    Term Delivery Preterm Delivery
  0           137                8
  1            28                3
```


```
### TEST TRANSITION TO CST IV AT M1 AND sPTB -- among HIV-Neg
the.model<-glm(preg_outcome_clean ~ CSTIV_trans_exp + sd_age + ph_ega + gravidity + bltest_sypresults + ph_discharge + ph_sores_ulcers + water, data = a3.mg[a3.mg$hiv %in% "HIV-Negative" & !is.na(a3.mg$CST_M1)  & !is.na(a3.mg$preg_outcome_clean), ], family = "binomial") 
change.to.cstiv.sptb.hivneg<-as.data.frame(broom::tidy(the.model, conf.int = T, exponentiate = T))[2,]
table(a3.mg[a3.mg$hiv %in% "HIV-Negative" & !is.na(a3.mg$CST_M1), "CSTIV_trans_exp"], a3.mg[a3.mg$hiv %in% "HIV-Negative" & !is.na(a3.mg$CST_M1), "preg_outcome_clean"])
```


```
    Term Delivery Preterm Delivery
  0            93                4
  1            28                3
```


```
save.image("MLAWI_Holm_Saidi_2024.RData")
```

LS0tCnRpdGxlOiAiTUxBV0kgMjAyMyIKb3V0cHV0OiBodG1sX25vdGVib29rCmVkaXRvcl9vcHRpb25zOiAKICBjaHVua19vdXRwdXRfdHlwZTogaW5saW5lCi0tLQoKVGhpcyBub3RlYm9vayBjYXB0dXJlcyB0aGUgYW5hbHlzaXMgb2YgdGhlIE1MQVdJIHByb2plY3QgYXMgb3V0bGluZWQgaW4gRmViXzA4X2VtYWlsL1NhaWRpX01pY3JvYmlvbWUgRHJhZnQuZG9jeCBieSBGcmlkYXkgU2FpZGkuIAoKMjRNYXIyMDIzOiAKLSBTd2l0Y2ggYXJvdW5kIHRoZSBleHBvc3VyZSBhbmQgdGhlIG91dGNvbWUgKHRoZSBleHBvc3VyZSBpcyBISVYgc3RhdHVzIGFuZCB0aGUgb3V0Y29tZSBpcyBDU1QpLiAKLSBSZS1leHBsb3JlIHRoZSBjb3ZhcmlhdGVzIGluY2x1ZGVkIHRvIG9ubHkgbG9vayBhdCB0aG9zZSBhc3NvY2lhdGVkIHdpdGggQ1NUIChvdXRjb21lKSwgbm90IEhJVi4gCi0gVXBkYXRlIHRoZSBtb2RlbHMgdG8gcmVmbGVjdCB0aGUgY29ycmVjdCBhZGp1c3RtZW50cy4KYGBge3J9CnNldHdkKCJ+L0Ryb3Bib3ggKElHUykvTVNML01MQVdJLyIpCmBgYAoKIyBEYXRlIGFuZCBjb2xvcnMKIyMgQ29sb3JzCmBgYHtyIENvbG9ycyB9CkNTVC5jb2w8LWFzLmRhdGEuZnJhbWUocmJpbmQoYygiSSIsICIjRkUwMzA4IiksYygiSS1BIiwgIiNGRTAzMDgiKSwgYygiSS1CIiwgIiNGNkQzREEiKSwgYygiSUkiLCAiIzg2QzYxQSIpLGMoIklJSSIsICIjRkY3MjAwIiksYygiSUlJLUEiLCAiI0ZGNzIwMCIpLGMoIklJSS1CIiwgIiNGOEE0MEUiKSwgYygiSVYiLCAiIzIyMTg4NiIpLGMoIklWLUEiLCAiIzQ0OEE3MyIpLCBjKCJJVi1CIiwgIiMyMjE4ODYiKSwgYygiSVYtQyIsICIjQzBBQ0QzIiksYygiSVYtQzAiLCAiIzk4OTg5OCIpLGMoIklWLUMxIiwgIiNFRjUzQTciKSxjKCJJVi1DMiIsICIjQTdERERDIiksYygiSVYtQzMiLCAiIzk4Qzk5OSIpLGMoIklWLUM0IiwgIiM3RjBCN0MiKSwgYygiViIsICIjRkFFNTBEIiksIGMoIiIsICJ3aGl0ZSIpLCBjKCJOQSIsICJ3aGl0ZSIpKSkKbmFtZXMoQ1NULmNvbCk8LWMoIkNTVCIsICJjb2xvciIpCgptZ0NTVC5jb2w8LWFzLmRhdGEuZnJhbWUocmJpbmQoYygiMSIsICIjRkUwMzA4IiksYygiMiIsICIjRjU0QzVFIiksIGMoIjMiLCAiI0YwNzA4NCIpLCBjKCI0IiwgIiNFQzk0QTUiKSxjKCI1IiwgIiNGMEJDQ0MiKSxjKCI2IiwgIiNGNkQzREEiKSxjKCI3IiwgIiM4NkM2MUEiKSwgYygiOCIsICIjQjREQjI5IiksYygiOSIsICIjREJFQTc3IiksIGMoIjEwIiwgIiNGRjcyMDAiKSwgYygiMTEiLCAiI0Y2OEExMSIpLGMoIjEyIiwgIiNGOEE0MEUiKSxjKCIxMyIsICIjRjNCQzExIiksYygiMTQiLCAiI2Y3ZDE1YSIpLCBjKCIxNSIsICIjRkFFNTBEIiksYygiMTYiLCAiI0YzRjQ2RSIpLGMoIjE3IiwgIiM0NDhBNzMiKSxjKCIxOCIsICIjODlCRUFCIiksIGMoIjE5IiwgIiNCQ0Q2Q0QiKSxjKCIyMCIsICIjMjIxODg2IiksYygiMjEiLCAiIzNFMzc5MiIpLGMoIjIyIiwgIiM1RDU3OUUiKSxjKCIyMyIsICIjN0M3NkFDIiksYygiMjQiLCAiIzlBOThCRiIpLGMoIjI1IiwgIiNDOUM4RDgiKSxjKCIyNiIsICIjOThDOTk5IiksIGMoIjI3IiwgIiM5ODk4OTgiKSwgYygiIiwgIndoaXRlIiksIGMoIk5BIiwgIndoaXRlIikpKQpuYW1lcyhtZ0NTVC5jb2wpPC1jKCJtZ0NTVCIsICJjb2xvciIpCmBgYApgYGB7cn0KbWdzcy5jb2xvcnM8LWFzLmRhdGEuZnJhbWUocmJpbmQoYygiTGFjdG9iYWNpbGx1c19jcmlzcGF0dXNfMSIsICIjRkUwMzA4IiksYygiTGFjdG9iYWNpbGx1c19jcmlzcGF0dXNfMiIsICIjRjU0QzVFIiksIGMoIkxhY3RvYmFjaWxsdXNfY3Jpc3BhdHVzXzMiLCAiI0YwNzA4NCIpLCBjKCJMYWN0b2JhY2lsbHVzX2NyaXNwYXR1c180IiwgIiNFQzk0QTUiKSxjKCJMYWN0b2JhY2lsbHVzX2NyaXNwYXR1c181IiwgIiNGMEJDQ0MiKSxjKCJMYWN0b2JhY2lsbHVzX2NyaXNwYXR1c182IiwgIiNGNkQzREEiKSxjKCJMYWN0b2JhY2lsbHVzX2dhc3NlcmlfMSIsICIjODZDNjFBIiksIGMoIkxhY3RvYmFjaWxsdXNfZ2Fzc2VyaV8yIiwgIiNCNERCMjkiKSxjKCJMYWN0b2JhY2lsbHVzX2dhc3NlcmlfMyIsICIjREJFQTc3IiksIGMoIkxhY3RvYmFjaWxsdXNfaW5lcnNfMSIsICIjRkY3MjAwIiksIGMoIkxhY3RvYmFjaWxsdXNfaW5lcnNfMiIsICIjRjY4QTExIiksYygiTGFjdG9iYWNpbGx1c19pbmVyc18zIiwgIiNGOEE0MEUiKSxjKCJMYWN0b2JhY2lsbHVzX2luZXJzXzUiLCAiI0YzQkMxMSIpLGMoIkxhY3RvYmFjaWxsdXNfaW5lcnNfNiIsICIjZjdkMTVhIiksYygiTGFjdG9iYWNpbGx1c19pbmVyc180IiwgIiNmN2QxNmEiKSwgYygiTGFjdG9iYWNpbGx1c19qZW5zZW5paV8xIiwgIiNGQUU1MEQiKSxjKCJMYWN0b2JhY2lsbHVzX2plbnNlbmlpXzIiLCAiI0YzRjQ2RSIpLGMoIkNhLkxhY2hub2N1cnZhX3ZhZ2luYWVfMSIsICIjNDQ4QTczIiksYygiQ2EuTGFjaG5vY3VydmFfdmFnaW5hZV8yIiwgIiM4OUJFQUIiKSwgYygiQ2EuTGFjaG5vY3VydmFfdmFnaW5hZV8zIiwgIiNCQ0Q2Q0QiKSxjKCJDYS5MYWNobm9jdXJ2YV92YWdpbmFlXzQiLCAiI0JDRDZDRiIpLCBjKCJDYS5MYWNobm9jdXJ2YV92YWdpbmFlXzUiLCAiI0JDRDZEQyIpLCBjKCJHYXJkbmVyZWxsYV92YWdpbmFsaXNfMCIsICIjMjIxOTkwIiksIGMoIkdhcmRuZXJlbGxhX3ZhZ2luYWxpc18xIiwgIiMyMjE4ODYiKSxjKCJHYXJkbmVyZWxsYV92YWdpbmFsaXNfMiIsICIjM0UzNzkyIiksYygiR2FyZG5lcmVsbGFfdmFnaW5hbGlzXzMiLCAiIzVENTc5RSIpLGMoIkdhcmRuZXJlbGxhX3ZhZ2luYWxpc180IiwgIiM3Qzc2QUMiKSxjKCJHYXJkbmVyZWxsYV92YWdpbmFsaXNfNSIsICIjOUE5OEJGIiksYygiR2FyZG5lcmVsbGFfdmFnaW5hbGlzXzYiLCAiI0M5QzhEOCIpLGMoIkJpZmlkb2JhY3Rlcml1bSoiLCAiIzk4Qzk5OSIpLCBjKCJTdGFwaHlsb2NvY2N1cyoiLCAiIzk4OTg5OCIpLCBjKCIiLCAid2hpdGUiKSwgYygiTkEiLCAid2hpdGUiKSwgYygiRW50ZXJvY29jY3VzX2ZhZWNhbGlzIiwiI2JiZmZmZiIpLGMoIlJhb3VsdGVsbGFfcGxhbnRpY29sYSIsIiNmZmMyZmYiKSxjKCJnX1BlcHRvbmlwaGlsdXMiLCIjQ0NDQzAwIiksYygiU25lYXRoaWFfc2FuZ3VpbmVnZW5zIiwiI2M3YWE4ZiIpLGMoIkF0b3BvYml1bV92YWdpbmFlXzEiLCIjMDAwMGNkIiksYygiQXRvcG9iaXVtX3ZhZ2luYWVfMiIsIiMwMDAwY2QiKSxjKCJnX0F0b3BvYml1bSIsIiMwMDAwY2QiKSxjKCJMYWNvdGJhY2lsbHVzX2hlbHZldGljdXMiLCIjMDBjY2ZmIiksYygiTWFnZWVpYmFjaWxsdXNfaW5kb2xpY3VzIiwiIzNjYjM3MSIpLGMoImdfQW5hZXJvY29jY3VzIiwiIzg3Y2VmYSIpLGMoImdfR2FyZG5lcmVsbGEiLCIjMjBiMmFhIiksYygiTWVnYXNwaGFlcmFfc3BfdHlwZV8xIiwiIzAwMDBmZiIpLGMoIlN0cmVwdG9jb2NjdXNfYWdhbGFjdGlhZSIsIiNmZjczOGEiKSxjKCJnX01lZ2FzcGhhZXJhIiwiIzAwOEI0NSIpLGMoIk1lZ2FzcGhhZXJhX2dlbm9tb3NwLiIsIiMwMDhCNDUiKSxjKCJTdHJlcHRvY29jY3VzX29yYWxpcyIsIiNmZjY2ZmYiKSxjKCJQcmV2b3RlbGxhX2JpdmlhXzEiLCIjYmZiZmJmIiksYygiUHJldm90ZWxsYV9iaXZpYV8xIiwiI2JmYmZiZiIpLGMoIlByZXZvdGVsbGFfYml2aWFfMyIsIiNiZmJmYmYiKSwgYygiQWVyb2NvY2N1c19jaHJpc3RlbnNlbmlpIiwiI2JlYmViZSIpLGMoIkFuYWVyb2NvY2N1c190ZXRyYWRpdXMiLCIjODdjZWZhIiksYygiR2VtZWxsYSIsIiNkYWE1MjAiKSxjKCJQcmV2b3RlbGxhX2dlbm9ncm91cF8xIiwiI2IwYjBiMCIpLGMoIlByZXZvdGVsbGFfYnVjY2FsaXMiLCIjYjBiMGIwIiksYygiTGFjdG9iYWNpbGx1c192YWdpbmFsaXMiLCIjZmZmZmZmIiksYygiT3RoZXIiLCJibGFjayIpLGMoIkJpZmlkb2JhY3Rlcml1bV9sb25ndW0iLCIjYzFmZmMxIiksYygiQmlmaWRvYmFjdGVyaXVtX2JyZXZlIiwiI2MxZmZjMSIpLGMoIkVnZ2VydGhlbGxhIiwiI0RFNzcxMCIpLGMoIk15Y29wbGFzbWFfaG9taW5pcyIsIiMxMERFNEUiKSxjKCJQb3JwaHlyb21vbmFzX2Jlbm5vbmlzIiwiI0RFNDMxMCIpLGMoIkV1YmFjdGVyaXVtX3NhcGhlbnVtIiwiIzhGMTBERSIpLGMoIkZ1c29iYWN0ZXJpdW1fbnVjbGVhdHVtIiwiI0NEODUzRiIpLGMoIkZ1c29iYWN0ZXJpdW1fZ29uaWRpYWZvcm1hbnMiLCIjQ0Q4NTNGIiksYygiU3RyZXB0b2NvY2N1c19hbmdpbm9zdXMiLCIjZmZjMGNiIiksYygiUGVwdG9zdHJlcHRvY29jY3VzX2FuYWVyb2JpdXMiLCIjREVEQjEwIiksYygiQXJjYW5vYmFjdGVyaXVtX3Bob2NhZSIsIiM4YzEwZGUiKSxjKCJCYWN0ZXJvaWRlc191bmlmb3JtaXMiLCIjZGUxMDU4IiksYygiVXJlYXBsYXNtYV9wYXJ2dW0iLCIjOTk5OWZmIiksYygiUGVwdG9uaXBoaWx1c19oYXJlaSIsIiNDQ0NDMDAiKSxjKCJNb2JpbHVuY3VzX211bGllcmlzIiwiI2YwODA4MCIpLGMoIk1lZ2FzcGhhZXJhX3NwLl90eXBlXzIiLCIjMDA4QjQ1IiksYygiTWFnZWVpYmFjaWxsdXNfaW5kb2xpY3VzIiwiIzNiYjE2ZiIpLGMoImdfRXNjaGVyaWNoaWEuU2hpZ2VsbGEiLCIjMTI0NTZiIiksYygiUGVwdG9uaXBoaWx1c19sYWNyaW1hbGlzIiwiI2QyYjQ4YyIpLGMoIlZlaWxsb25lbGxhX21vbnRwZWxsaWVyZW5zaXMiLCIjZmY4YzY5IiksYygiUHJldm90ZWxsYV9nZW5vZ3JvdXBfMyIsIiNiMzYyMDAiKSxjKCJQcmV2b3RlbGxhX2Rpc2llbnMiLCIjYjM2MjAwIiksYygiUGFydmltb25hc19taWNyYSIsIiNjZGNkMDAiKSxjKCJDb3J5bmViYWN0ZXJpdW1fYWNjb2xlbnMiLCIjZmZmZjAwIiksYygiRmluZWdvbGRpYV9tYWduYSIsIiM4MDAwODAiKSxjKCJQcmV2b3RlbGxhX2dlbm9ncm91cF8yIiwiI2IwYjBiMCIpLGMoIlN0YXBoeWxvY29jY3VzX2VwaWRlcm1pZGlzIiwiI2ZmZmZmZiIpLGMoIlByZXZvdGVsbGFfdGltb25lbnNpcyIsIiNmOGZjYTkiKSxjKCJnX1N0cmVwdG9jb2NjdXMiLCIjZmZjMGNiIiksYygiZ19CaWZpZG9iYWN0ZXJpdW0iLCIjYzFmZmMxIiksYygiZ19FbnRlcm9jb2NjdXMiLCIjYmJmZmZmIiksYygiZ19TdGFwaHlsb2NvY2N1cyIsIiM4MDAwODAiKSxjKCJnX0ZpbmVnb2xkaWEiLCIjODAwMDgwIiksYygiZ19QcmV2b3RlbGxhIiwiI2JmYmZiZiIpLGMoImdfU25lYXRoaWEiLCIjZDFlOGViIiksYygiZ19BZXJvY29jY3VzIiwiI2U4ZTFiYSIpLGMoImdfTGVwdG90cmljaGlhIiwiIzdjYTM4NiIpLGMoImdfVmVpbGxvbmVsbGEiLCIjNDk5OTk2IiksYygiZ19EaWFsaXN0ZXIiLCIjOTk3ZGJkIiksYygiZ19Db3J5bmViYWN0ZXJpdW1fMSIsIiNmZmZmMDAiKSxjKCJnX1ZhcmliYWN1bHVtIiwiIzA5NDcxNyIpLGMoImdfRGVsZnRpYSIsIiMwOTBjNDciKSxjKCJnX0NvcnluZWJhY3Rlcml1bV8xIiwiI2ZmZmYwMCIpLGMoIlByZXZvdGVsbGFfYW1uaWlfMSIsIiNhYzhmYzciKSxjKCJQcmV2b3RlbGxhX2FtbmlpXzYiLCIjYWM4ZmM3IiksYygiU25lYXRoaWFfYW1uaWkiLCIjNjk2M2ZmIiksIGMoIlByb3Bpb25pYmFjdGVyaXVtX3NwLiIsIiNiZmJmYmQiKSkpCm5hbWVzKG1nc3MuY29sb3JzKTwtYygidGF4YSIsICJjb2xvciIpCgp0YXhhLmNvbG9yczwtYXMuZGF0YS5mcmFtZShyYmluZChjKCJMYWN0b2JhY2lsbHVzX2NyaXNwYXR1cyIsIiNmZjAwMDAiKSxjKCJHYXJkbmVyZWxsYV92YWdpbmFsaXMiLCIjMjBiMmFhIiksYygiZ19MYWN0b2JhY2lsbHVzIiwiI2VlZjA2YyIpLGMoIkxhY3RvYmFjaWxsdXNfaW5lcnMiLCIjZmY4YzAwIiksYygiTGFjdG9iYWNpbGx1c19nYXNzZXJpIiwiIzdmZmYwMCIpLGMoIkxhY3RvYmFjaWxsdXNfamVuc2VuaWkiLCIjMzMzMzMzIiksYygiRW50ZXJvY29jY3VzX2ZhZWNhbGlzIiwiI2JiZmZmZiIpLGMoIlJhb3VsdGVsbGFfcGxhbnRpY29sYSIsIiNmZmMyZmYiKSxjKCJnX1BlcHRvbmlwaGlsdXMiLCIjQ0NDQzAwIiksYygiU25lYXRoaWFfc2FuZ3VpbmVnZW5zIiwiI2M3YWE4ZiIpLGMoIkF0b3BvYml1bV92YWdpbmFlIiwiIzAwMDBjZCIpLGMoImdfQXRvcG9iaXVtIiwiIzAwMDBjZCIpLGMoIkxhY290YmFjaWxsdXNfaGVsdmV0aWN1cyIsIiMwMGNjZmYiKSxjKCJNYWdlZWliYWNpbGx1c19pbmRvbGljdXMiLCIjM2NiMzcxIiksYygiZ19BbmFlcm9jb2NjdXMiLCIjODdjZWZhIiksYygiZ19HYXJkbmVyZWxsYSIsIiMyMGIyYWEiKSxjKCJNZWdhc3BoYWVyYV9zcF90eXBlXzEiLCIjMDAwMGZmIiksYygiU3RyZXB0b2NvY2N1c19hZ2FsYWN0aWFlIiwiI2ZmNzM4YSIpLGMoImdfTWVnYXNwaGFlcmEiLCIjMDA4QjQ1IiksYygiTWVnYXNwaGFlcmFfZ2Vub21vc3AuIiwiIzAwOEI0NSIpLGMoIlN0cmVwdG9jb2NjdXNfb3JhbGlzIiwiI2ZmNjZmZiIpLGMoIlByZXZvdGVsbGFfYml2aWEiLCIjYmZiZmJmIiksYygiQWVyb2NvY2N1c19jaHJpc3RlbnNlbmlpIiwiI2JlYmViZSIpLGMoIkFuYWVyb2NvY2N1c190ZXRyYWRpdXMiLCIjODdjZWZhIiksYygiR2VtZWxsYSIsIiNkYWE1MjAiKSxjKCJQcmV2b3RlbGxhX2dlbm9ncm91cF8xIiwiI2IwYjBiMCIpLGMoIlByZXZvdGVsbGFfYnVjY2FsaXMiLCIjYjBiMGIwIiksYygiTGFjdG9iYWNpbGx1c192YWdpbmFsaXMiLCIjZmZmZmZmIiksYygib3RoZXIiLCIjODA4MDgwIiksYygiQmlmaWRvYmFjdGVyaXVtX2xvbmd1bSIsIiNjMWZmYzEiKSxjKCJCaWZpZG9iYWN0ZXJpdW1fYnJldmUiLCIjYzFmZmMxIiksYygiRWdnZXJ0aGVsbGEiLCIjREU3NzEwIiksYygiTXljb3BsYXNtYV9ob21pbmlzIiwiIzEwREU0RSIpLGMoIlBvcnBoeXJvbW9uYXNfYmVubm9uaXMiLCIjREU0MzEwIiksYygiRXViYWN0ZXJpdW1fc2FwaGVudW0iLCIjOEYxMERFIiksYygiRnVzb2JhY3Rlcml1bV9udWNsZWF0dW0iLCIjQ0Q4NTNGIiksYygiRnVzb2JhY3Rlcml1bV9nb25pZGlhZm9ybWFucyIsIiNDRDg1M0YiKSxjKCJTdHJlcHRvY29jY3VzX2FuZ2lub3N1cyIsIiNmZmMwY2IiKSxjKCJQZXB0b3N0cmVwdG9jb2NjdXNfYW5hZXJvYml1cyIsIiNERURCMTAiKSxjKCJBcmNhbm9iYWN0ZXJpdW1fcGhvY2FlIiwiIzhjMTBkZSIpLGMoIkJhY3Rlcm9pZGVzX3VuaWZvcm1pcyIsIiNkZTEwNTgiKSxjKCJVcmVhcGxhc21hX3BhcnZ1bSIsIiM5OTk5ZmYiKSxjKCJQZXB0b25pcGhpbHVzX2hhcmVpIiwiI0NDQ0MwMCIpLGMoIk1vYmlsdW5jdXNfbXVsaWVyaXMiLCIjZjA4MDgwIiksYygiTWVnYXNwaGFlcmFfc3AuX3R5cGVfMiIsIiMwMDhCNDUiKSxjKCJDYV9MYWNobmN1cnZhVmFnaW5hZSIsIiNiMzE5MDAiKSxjKCJnX0VzY2hlcmljaGlhLlNoaWdlbGxhIiwiIzEyNDU2YiIpLGMoIlBlcHRvbmlwaGlsdXNfbGFjcmltYWxpcyIsIiNkMmI0OGMiKSxjKCJWZWlsbG9uZWxsYV9tb250cGVsbGllcmVuc2lzIiwiI2ZmOGM2OSIpLGMoIlByZXZvdGVsbGFfZ2Vub2dyb3VwXzMiLCIjYjM2MjAwIiksYygiUHJldm90ZWxsYV9kaXNpZW5zIiwiI2IzNjIwMCIpLGMoIlBhcnZpbW9uYXNfbWljcmEiLCIjY2RjZDAwIiksYygiQ29yeW5lYmFjdGVyaXVtX2FjY29sZW5zIiwiI2ZmZmYwMCIpLGMoIkZpbmVnb2xkaWFfbWFnbmEiLCIjODAwMDgwIiksYygiUHJldm90ZWxsYV9nZW5vZ3JvdXBfMiIsIiNiMGIwYjAiKSxjKCJTdGFwaHlsb2NvY2N1c19lcGlkZXJtaWRpcyIsIiNmZmZmZmYiKSxjKCJQcmV2b3RlbGxhX3RpbW9uZW5zaXMiLCIjZjhmY2E5IiksYygiZ19TdHJlcHRvY29jY3VzIiwiI2ZmYzBjYiIpLGMoImdfQmlmaWRvYmFjdGVyaXVtIiwiI2MxZmZjMSIpLGMoImdfRW50ZXJvY29jY3VzIiwiI2JiZmZmZiIpLGMoImdfU3RhcGh5bG9jb2NjdXMiLCIjODAwMDgwIiksYygiZ19GaW5lZ29sZGlhIiwiIzgwMDA4MCIpLGMoImdfUHJldm90ZWxsYSIsIiNiZmJmYmYiKSxjKCJnX1NuZWF0aGlhIiwiI2QxZThlYiIpLGMoImdfQWVyb2NvY2N1cyIsIiNlOGUxYmEiKSxjKCJnX0xlcHRvdHJpY2hpYSIsIiM3Y2EzODYiKSxjKCJnX1ZlaWxsb25lbGxhIiwiIzQ5OTk5NiIpLGMoImdfRGlhbGlzdGVyIiwiIzk5N2RiZCIpLGMoImdfQ29yeW5lYmFjdGVyaXVtXzEiLCIjZmZmZjAwIiksYygiZ19WYXJpYmFjdWx1bSIsIiMwOTQ3MTciKSxjKCJnX0RlbGZ0aWEiLCIjMDkwYzQ3IiksYygiUHJldm90ZWxsYV9hbW5paSIsIiNhYzhmYzciKSxjKCJTbmVhdGhpYV9hbW5paSIsIiM2OTYzZmYiKSkpCm5hbWVzKHRheGEuY29sb3JzKTwtYygidGF4YSIsICJjb2xvciIpCgoKaGl2LmNvbHM8LWMoImdvbGQiLCAiZGFya29yY2hpZDQiKQp0cmVhdC5jb2xzPC1jKCIjQjhERTI5RkYiLCAiIzMzNjM4REZGIikKYGBgCgoKIyMgRGF0ZQpgYGB7cn0KdG9kYXkgPC0gc3Ryc3BsaXQoZGF0ZSgpLCAiICIpCm1vbnRoIDwtIHRvZGF5W1sxXV1bMl0KaWYodG9kYXlbWzFdXVszXSAlaW4lICIiKXsKICAgIGRheSA8LSB0b2RheVtbMV1dWzRdCiAgICB5ZWFyIDwtIHRvZGF5W1sxXV1bNl0KICAgIH1lbHNlewogICAgZGF5IDwtIHRvZGF5W1sxXV1bM10KICAgIHllYXIgPC0gdG9kYXlbWzFdXVs1XQogICAgfQp0b2RheTIgPC0gcGFzdGUoZGF5LG1vbnRoLHllYXIsIHNlcD0iIikKYGBgCgojIyBQYWNrYWdlcwpgYGB7ciBQYWNrYWdlc30KcmVxdWlyZShicm9vbSkKcmVxdWlyZShnZ3Bsb3QyKQpyZXF1aXJlKGRwbHlyKQpyZXF1aXJlKHJlc2hhcGUyKQpyZXF1aXJlKEVudlN0YXRzKQpyZXF1aXJlKHJlYWR4bCkKcmVxdWlyZSh2ZWdhbikKcmVxdWlyZShzdHJpbmdyKQpyZXF1aXJlKGdncHVicikKcmVxdWlyZShnZ3N0YXRzcGxvdCkKcmVxdWlyZShERVNlcTIpCnJlcXVpcmUoZ2dyZXBlbCkKcmVxdWlyZShsbWVyVGVzdCkKcmVxdWlyZShsbWU0KQpyZXF1aXJlKHRpZHl2ZXJzZSkKcmVxdWlyZShwaHlsb3NlcSkKbGlicmFyeShlZGdlUikKcmVxdWlyZSh0YWJsZTEpCnJlcXVpcmUobmxtZSkKcmVxdWlyZShlcGl0b29scykKcmVxdWlyZShnZ3BtaXNjKQpyZXF1aXJlKHBoZWF0bWFwKQoKcHZhbHVlIDwtIGZ1bmN0aW9uKHgsIC4uLikgewogICMgQ29uc3RydWN0IHZlY3RvcnMgb2YgZGF0YSB5LCBhbmQgZ3JvdXBzIChzdHJhdGEpIGcKICB5IDwtIHVubGlzdCh4KQogIGcgPC0gZmFjdG9yKHJlcCgxOmxlbmd0aCh4KSwgdGltZXM9c2FwcGx5KHgsIGxlbmd0aCkpKQogIGlmIChpcy5udW1lcmljKHkpKSB7CiAgICAjIEZvciBudW1lcmljIHZhcmlhYmxlcywgcGVyZm9ybSBhIHN0YW5kYXJkIDItc2FtcGxlIHQtdGVzdAogICAgcCA8LSByb3VuZCh0LnRlc3QoeSB+IGcpJHAudmFsdWUsIDIpCiAgfSBlbHNlIHsKICAgICMgRm9yIGNhdGVnb3JpY2FsIHZhcmlhYmxlcywgcGVyZm9ybSBhIGNoaS1zcXVhcmVkIHRlc3Qgb2YgaW5kZXBlbmRlbmNlCiAgICBwIDwtIHJvdW5kKGNoaXNxLnRlc3QodGFibGUoeSwgZykpJHAudmFsdWUsIDIpCiAgfQogICMgRm9ybWF0IHRoZSBwLXZhbHVlLCB1c2luZyBhbiBIVE1MIGVudGl0eSBmb3IgdGhlIGxlc3MtdGhhbiBzaWduLgogICMgVGhlIGluaXRpYWwgZW1wdHkgc3RyaW5nIHBsYWNlcyB0aGUgb3V0cHV0IG9uIHRoZSBsaW5lIGJlbG93IHRoZSB2YXJpYWJsZSBsYWJlbC4KICBjKCIiLCBzdWIoIjwiLCAiJmx0OyIsIGZvcm1hdC5wdmFsKHAsIGRpZ2l0cz0yLCBlcHM9MC4wMSkpKQp9CmBgYAojIExvYWQgRGF0YQpgYGB7cn0KI2xvYWQoIk1MQVdJLlJEYXRhIikKYGBgCgojIE1ldGFkYXRhCgojIyBBaW0gMQpBaW0gMTogIFRvIGNvbXBhcmUgdGhlIHZhZ2luYWwgbWljcm9iaW90YSBjb21wb3NpdGlvbiBhbmQgc3RydWN0dXJlLCBiZXR3ZWVuIEhJVisgYW5kIEhJVi0gcHJlZ25hbnQgd29tZW4sIHByaW9yIHRvIGluaXRpYXRpb24gb2YgZWl0aGVyIEFSVCAoZm9yIEhJVisgcHJlZ25hbnQgd29tZW4pIG9yIFByRVAgKGZvciBISVYtIHByZWduYW50IHdvbWVuKS4KIE9SIHByZS10cmVhdG1lbnQgKEFSVCBvciBQckVQKSBtaWNyb2Jpb21lIEhJViArLy0KCkFJTSAxIEFuYWx5c2lzIHBsYW46IFdlIHdpbGwgcXVhbnRpZnkga2V5IHN0cnVjdHVyZSBhbmQgZnVuY3Rpb25hbCBjaGFyYWN0ZXJpc3RpY3Mgb2YgdGhlIHZhZ2luYWwgbWljcm9iaW90YSBhdCBiYXNlbGluZSAocHJpb3IgdG8gZXhwb3N1cmUgdG8gQVJWcykgdXNpbmcgCjEuIG1lYW4gcmVsYXRpdmUgYWJ1bmRhbmNlIG9mIHByZXZhbGVudCBiYWN0ZXJpYWwgdGF4YSAoZS5nLiwgTC4gY3Jpc3BhdHVzLCBMLiBpbmVycywgRy4gdmFnaW5hbGlzKSwgYW5kIAoyLiBtZXRhZ2Vub21pYyBjbHVzdGVyczsgCjMuIHNwZWNpZXMgZGl2ZXJzaXR5IChTaGFubm9uIGRpdmVyc2l0eSBpbmRleCk7IGFuZCAKNC4gc3BlY2llcyByaWNobmVzcyAoQ2hhbyByaWNobmVzcyBlc3RpbWF0b3IpLiAKClRoZSB0YXhvbm9taWMgY29tcG9zaXRpb24gb2YgdGhlIHZhZ2luYWwgbWljcm9iaW90YSB3aWxsIGJlIGVzdGFibGlzaGVkIGJ5IG1hcHBpbmcgdGhlIG1ldGFnZW5vbWljIHJlYWRzIHRvIFZJUkdPLCBhIGNvbXByZWhlbnNpdmUgZ2VuZSBjYXRhbG9ndWUgb2YgdGhlIHZhZ2luYWwgbWljcm9iaW9tZS4gVGhlIHBhdHRlcm5zIG9mIGdlbmUgY29udGVudCBpbiBlYWNoIHNhbXBsZSB3aWxsIGJlIHN1YmplY3RlZCB0byBoaWVyYXJjaGljYWwgY2x1c3RlcmluZyB1c2luZyBCcmF5LUN1cnRpcyBkaXNzaW1pbGFyaXR5IGFuZCBXYXJkIGxpbmthZ2UgdG8gZXN0YWJsaXNoIG1ldGFnZW5vbWljIGNsdXN0ZXJzLiBCZWNhdXNlIG9mIFZJUkdPIGV4dGVuc2l2ZSBmdW5jdGlvbmFsIGFuZCB0YXhvbm9taWMgYW5ub3RhdGlvbnMsIHRoZXNlIGNsdXN0ZXJzIGFyZSBkZWZpbmVkIGJ5IGJvdGggY29tcG9zaXRpb24gYW5kIGZ1bmN0aW9uLiAKCkFzc29jaWF0aW9ucyBiZXR3ZWVuIHZhZ2luYWwgbWljcm9iaW90YSBjb21wb3NpdGlvbiAodGF4b25vbXkpIGFuZCBzdHJ1Y3R1cmUgKG1ldGFnZW5vbWljIGNsdXN0ZXJzKSBhbmQgSElWIHNlcm9zdGF0dXMgKEhJVisgdnMgSElWLSkgYW5kIG1pY3JvYmlvbWUgY2hhcmFjdGVyaXN0aWNzIHdpbGwgYmUgdGVzdGVkIHVzaW5nIGEgcmFuay1zdW0gdGVzdCBhbmQgbGluZWFyIHJlZ3Jlc3Npb24gd2lsbCBiZSB1c2VkIGluIGFkanVzdGVkIGFuYWx5c2VzLgoKRmlndXJlIDI6IENvbXBhcmlzb24gb2YgdGhlIG1lYW4gcmVsYXRpdmUgYWJ1bmRhbmNlIGJ5IEhJViBzdGF0dXMKRmlndXJlIDNhOiBTaGFubm9uIGRpdmVyc2l0eSBpbmRpY2VzIGJ5IGNvbW11bml0eSBzdGF0ZSB0eXBlIGFuZCBGaWd1cmUgM2IgU2hhbm5vbiBkaXZlcnNpdHkgaW5kaWNlcyBieSBISVYgc2Vyb3N0YXR1cwoKUVVFU1RJT046IFdIRVJFIElTIE1FVEFEQVRBIEZPUiBBREpVU1RNRU5UUywgU1VDSCBBUyBUSU1FIElOIFBSRUdOQU5DWSwgU1lQSElMSVMgSU5GRUNUSU9OLCBhbnRpYmlvdGljcz8KIC0tIFN5cGhpbGlzIGJjIGhpZ2ggcmlzayBncm91cCAtLSBpdCB3YXMgcGFydCBvZiBlbnJvbGxtZW50IGNyaXRlcmlhIGZvciBISVYgKC0pIHRvIGhhdmUgaGFkIFNUSSBvciB2YWdpbmFsIGluIHByaW9yIDMgbW9udGhzCiAKCm1hdGVybmFsIGFnZSwgcGFyaXR5LCBlZHVjYXRpb24gYW5kIHNvY2lvZWNvbm9taWMgc3RhdHVzLCBudXRyaXRpb24sIGdlc3RhdGlvbmFsIGFnZSBhdCB0aGUgdGltZSBvZiBzYW1wbGUgY29sbGVjdGlvbiwgSElWIHZpcmFsIGxvYWQgKEZvciBISVYrIHdvbWVuKT8KYGBge3J9CmExLnQxPC1yZWFkX2V4Y2VsKCJGZWJfMDhfRW1haWwvU2FpZGlfQWltMV9UcmlhbDFfQW5hbHl0aWNTYW1wbGUueGxzeCIpCmRpbShhMS50MSkgIyMgNjQgeCA1IC0tLSBUaGVzZSBhcmUgSElWKwphMS50MSRoaXY8LTEKYTEudDI8LXJlYWRfZXhjZWwoIkZlYl8wOF9FbWFpbC9TYWlkaV9BaW0xX1RyaWFsMl9BbmFseXRpY1NhbXBsZS54bHN4IikKZGltKGExLnQyKSAjIyAxOTEgeCAzIC0tLSBISVYgKC0pCmExLnQyJGhpdjwtMAoKYTE8LW1lcmdlKGExLnQxLCBhMS50MiwgYWxsPVRSVUUpCiMyNTUgeCA2CmExJGhpdjwtZmFjdG9yKGExJGhpdikKYTEkaGl2PC1yZWxldmVsKGExJGhpdiwgcmVmPSIxIikKYTEkYWltPC0iQWltIDEiCmBgYAoKIyMgQWltIDIKQWltIDI6IFRvIGNvbXBhcmUgbG9uZ2l0dWRpbmFsIGNoYW5nZXMgaW4gdmFnaW5hbCBtaWNyb2Jpb3RhIGNvbXBvc2l0aW9uIGFuZCBzdHJ1Y3R1cmUsIHdpdGhpbiBISVYrIHdvbWVuIG9uIEFSVCAoRFRHLzNUQy9UREYpIGFuZCB3aXRoaW4gSElWLSB3b21lbiBvbiBQckVQLCBiZWZvcmUgYW5kIGFmdGVyIGluaXRpYXRpb24gb2YgQVJWIGRydWdzLgoKQW5hbHlzaXMgcGxhbjogVG8gZXZhbHVhdGUgdGhlIGxvbmdpdHVkaW5hbCBjaGFuZ2VzIGluIHRoZSBtaWNyb2Jpb3RhIGNvbXBvc2l0aW9uIGFuZCBzdHJ1Y3R1cmUsIHdlIHdpbGwgYXNzZXNzIHRoZSByZWxhdGl2ZSBzdGFiaWxpdHkgb2YgdGhlIHZhZ2luYWwgbWljcm9iaW9tZSBiZWZvcmUgYW5kIGFmdGVyIGV4cG9zdXJlIHRvIEFSVnMgdXNpbmcgdmFyaW91cyBtZWFzdXJlczogMSkgY2hhbmdlIGluIFNoYW5ub24gRGl2ZXJzaXR5IEluZGV4IGFuZCBhbiBpbmRpY2F0b3IgdmFyaWFibGUgZm9yIGluY3JlYXNpbmcgZGl2ZXJzaXR5LCAyKSBjaGFuZ2UgaW4gc3BlY2llcyByaWNobmVzcyBhbmQgYW4gaW5kaWNhdG9yIGZvciBpbmNyZWFzaW5nIHJpY2huZXNzLCAzKSBKZW5zZW4tU2hhbm5vbiBkaXZlcmdlbmNlIChKU0QpLCAgYW5kIDQpIGFuIGluZGljYXRvciBvZiBjaGFuZ2UgZnJvbSBMYWN0b2JhY2lsbHVzLWRvbWluYW50IHRvIExhY3RvYmFjaWxsdXMtZGVmaWNpZW50IG1ldGFnZW5vbWljIGNsdXN0ZXJzLiBBbGwgdGhlc2UgbWVhc3VyZXMgaW4gdmFnaW5hbCBtaWNyb2Jpb3RhIGNvbXBvc2l0aW9uIGFuZCBzdHJ1Y3R1cmUgd2lsbCBiZSBjb21wYXJlZCB3aXRoaW4gSElWKyB3b21lbiBvbiBBUlQgKERURy8zVEMvVERGKSBhbmQgd2l0aGluIEhJVi0gd29tZW4gb24gUHJFUCBiZWZvcmUgYW5kIGFmdGVyIGV4cG9zdXJlIHRvIEFSVnMgdXNpbmcgbG9naXN0aWMgcmVncmVzc2lvbi4KCkZpZ3VyZSA0OiBDaGFuZ2VzIGluIFNoYW5ub24gZGl2ZXJzaXR5IGluZGljZXMsIHNwZWNpZXMgcmljaG5lc3MgYW5kIEplbnNlbi1TaGFubm9uIGRpdmVyZ2VuY2UgYnkgSElWIHNlcm9zdGF0dXMvQVJWIHR5cGUgKEFSVCBhbmQgUHJFUCkKClNlbnNpdGl2aXR5IGFuYWx5c2lzIHRvIGFjY291bnQgZm9yIGNhc2VzIHdoZXJlIHRoZXkgZGlkIG5vdCBhZGhlcmUgdG8gUFJFcCBvciBISVYKClFVRVNUSU9OOiBGT1IgSElWLSBXSEVSRSBJUyBQUkVQIElOSVRJQVRJT04gREFUQT8KYGBge3J9CmEyLnQxPC1yZWFkX2V4Y2VsKCJGZWJfMDhfRW1haWwvU2FpZGlfQWltMiYzX1RyaWFsMV9BbmFseXRpY1NhbXBsZS54bHN4IikKZGltKGEyLnQxKSAjIDQ5IFggOQphMi50MSRoaXY8LTEKYTIudDFhPC1hMi50MVssYygxOjMsNToxMCldCm5hbWVzKGEyLnQxYSlbM108LSJtYXJ5bGFuZF9JRCIKYTIudDFhJHN3YWI8LTEKYTIudDFiPC1hMi50MVssYygxOjIsNDoxMCldCm5hbWVzKGEyLnQxYilbM108LSJtYXJ5bGFuZF9JRCIKYTIudDFiJHN3YWI8LTIKYTIudDE8LXJiaW5kKGEyLnQxYSwgYTIudDFiKQoKYTIudDI8LXJlYWRfZXhjZWwoIkZlYl8wOF9FbWFpbC9TYWlkaV9BaW0yJjNfVHJpYWwyX0FuYWx5dGljU2FtcGxlLnhsc3giKQpkaW0oYTIudDIpICMgMTMyIHggNwphMi50MiRoaXY8LTAKYTIudDJhPC1hMi50MlssYygxOjMsNTo4KV0KbmFtZXMoYTIudDJhKVszXTwtIm1hcnlsYW5kX0lEIgphMi50MmEkc3dhYjwtMQphMi50MmI8LWEyLnQyWyxjKDE6Miw0OjgpXQpuYW1lcyhhMi50MmIpWzNdPC0ibWFyeWxhbmRfSUQiCmEyLnQyYiRzd2FiPC0yCmEyLnQyPC1yYmluZChhMi50MmEsIGEyLnQyYikKCmEyPC1tZXJnZShhMi50MSwgYTIudDIsIGFsbD1UKQoKYTIkaGl2PC1mYWN0b3IoYTIkaGl2KQphMiRoaXY8LXJlbGV2ZWwoYTIkaGl2LCByZWY9IjEiKQoKYTIkYWltPC0iQWltIDIiCgphbGwuc2FtcGxlczwtbWVyZ2UoYTEsIGEyLCBhbGw9VFJVRSkKc2FtcGxlLmxpc3Q8LXVuaXF1ZShhbGwuc2FtcGxlcyRtYXJ5bGFuZF9JRCkKYGBgCgojIyBDb3ZhcmlhdGVzCmBgYHtyfQpmaWxlLm5hbWVzPC1saXN0LmZpbGVzKCJGZWJfMTBfZW1haWwvIiwgcGF0dGVybj0iKi54bHN4IiwgZnVsbC5uYW1lcyA9IFRSVUUpCmxpc3RfYWxsIDwtIGxhcHBseShmaWxlLm5hbWVzLCBmdW5jdGlvbih4KSByZWFkX2V4Y2VsKHgpKQpkaWN0aW9uYXJ5PC1saXN0X2FsbFs1XQpsaXN0X2FsbFs1XTwtTlVMTApmaWxlLm5hbWVzPC1saXN0LmZpbGVzKCJGZWJfMTBfZW1haWwvIiwgcGF0dGVybj0iKi54bHN4IiwgZnVsbC5uYW1lcyA9IEZBTFNFKVsxOjRdCgphMS5jb3ZhcmlhdGU8LW1lcmdlKGxpc3RfYWxsWzFdLGxpc3RfYWxsWzJdLCBhbGw9VFJVRSkKYTE8LW1lcmdlKGExLCBhMS5jb3ZhcmlhdGUsIGFsbD1UUlVFKQphMi5jb3ZhcmlhdGU8LW1lcmdlKGxpc3RfYWxsWzNdLGxpc3RfYWxsWzRdLCBhbGw9VFJVRSkKYTI8LW1lcmdlKGEyLCBhMi5jb3ZhcmlhdGUsIGFsbD1UUlVFKQpgYGAKCgojIE1ldGFnZW5vbWUgRGF0YQojIyMgU2VxdWVuY2luZyBRQQpgYGB7cn0KIyMgQ2hlY2sgcmVhZHMgZGVsaXZlcmVkIHZzLiBxdWFsaXR5IGZpbHRlcmVkIHJlYWRzIHBlciBzYW1wbGUKcmVhZC5zdGF0czwtcmVhZC5jc3YoIk1HX3Byb2Nlc3Npbmdfc3RhdHMuY3N2IikKcmVhZC5zdGF0cyRzYW1wbGVbcmVhZC5zdGF0cyRzYW1wbGUgJWluJSAiNDAxODI1X05FRyJdPC0iNDAxODI1X1pZTU9fUE9TIgpzYW1wbGVfZXhjbHVkZTwtcmVhZF9leGNlbCgiUUNfUmVzdWx0cy54bHN4IikKcmVhZC5zdGF0cyRleGNsdWRlPC1pZmVsc2UoZ3JlcGwoIkNvbnRyb2wiLCByZWFkLnN0YXRzJHR5cGUpLCBhcy5jaGFyYWN0ZXIocmVhZC5zdGF0cyR0eXBlKSwgaWZlbHNlKHJlYWQuc3RhdHMkc2FtcGxlICVpbiUgc2FtcGxlX2V4Y2x1ZGUkbWFyeWxhbmRfSUQsIGFzLmNoYXJhY3RlcihzYW1wbGVfZXhjbHVkZSRyZWFzb25bbWF0Y2gocmVhZC5zdGF0cyRzYW1wbGUsIHNhbXBsZV9leGNsdWRlJG1hcnlsYW5kX0lEKV0pLCBOQSkpCiMjIDEzMiBzYW1wbGVzIChzd2Ficykgd2VyZSBleGNsdWRlZCBmb3IgcmVhc29ucyBpbmRpY2F0ZWQgaW4gImV4Y2x1ZGUiIGNvbHVtbgoKcmVhZC5zdGF0cy5tPC1yZXNoYXBlMjo6bWVsdChyZWFkLnN0YXRzW2lzLm5hKHJlYWQuc3RhdHMkZXhjbHVkZSksIGFzLnZlY3RvcihuYW1lcyhyZWFkLnN0YXRzKVsxOjEzXSldLCBpZC52YXJzPWMoInNhbXBsZSIsICJ0eXBlIiksIHZhcmlhYmxlLm5hbWUgPSAic3RhdF90eXBlIiwgdmFsdWUubmFtZSA9ICJyZWFkcyIpICMjIG1lbHQgYWxsIGNvbHVtbnMgZXhjZXB0ICJleGNsdWRlIiAoY29sdW1uIDE0KQpyZWFkLnN0YXRzLm0kZ3JvdXA8LWlmZWxzZShncmVwbCgiQ29udHJvbCIsIHJlYWQuc3RhdHMubSR0eXBlKSwgYXMuY2hhcmFjdGVyKHJlYWQuc3RhdHMubSR0eXBlKSwgaWZlbHNlKHJlYWQuc3RhdHMubSRzYW1wbGUgJWluJSBhbGwuc2FtcGxlc1thbGwuc2FtcGxlcyRoaXYgPT0gMSwgIm1hcnlsYW5kX0lEIl0sICJXTEhJViIsIGlmZWxzZShyZWFkLnN0YXRzLm0kc2FtcGxlICVpbiUgYWxsLnNhbXBsZXNbYWxsLnNhbXBsZXMkaGl2ID09IDAsICJtYXJ5bGFuZF9JRCJdLCAiSElWLU5lZ2F0aXZlIiwgTkEpKSkKcmVhZC5zdGF0cy5tJHByZV9wb3N0PC1pZmVsc2UoZ3JlcGwoIkNvbnRyb2wiLCByZWFkLnN0YXRzLm0kdHlwZSksIGFzLmNoYXJhY3RlcihyZWFkLnN0YXRzLm0kdHlwZSksIGlmZWxzZShyZWFkLnN0YXRzLm0kc2FtcGxlICVpbiUgYTIkbWFyeWxhbmRfSUQsIGFzLmNoYXJhY3RlcihhMiRzd2FiW21hdGNoKHJlYWQuc3RhdHMubSRzYW1wbGUsIGEyJG1hcnlsYW5kX0lEKV0pLCAiMSIpKQoKdG8ucGxvdDwtcmVhZC5zdGF0cy5tCnRvLnBsb3QkZ3JvdXA8LWZhY3Rvcih0by5wbG90JGdyb3VwLCBsZXZlbHM9YygiV0xISVYiLCAiSElWLU5lZ2F0aXZlIikpCmdncGxvdCh0by5wbG90W3RvLnBsb3Qkc3RhdF90eXBlICVpbiUgYygiUmVhZHNfZGVsaXZlcmVkIiwgImhvc3RfcmVtb3ZlZCIsICJyUk5BX3JlbW92ZWQiLCAicXVhbGl0eV9maWx0ZXJlZCIpLCBdLCBhZXMoeD1ncm91cCwgeT1hcy5udW1lcmljKHJlYWRzKSwgZmlsbD1ncm91cCkpK2dlb21fYm94cGxvdChsd2Q9MC4xLCBub3RjaCA9IFQsIG91dGxpZXIuc2hhcGUgPSBOQSkrZ2VvbV9qaXR0ZXIod2lkdGg9MC4xLCBzaXplPTAuMikrc2NhbGVfeV9sb2cxMCgpK3RoZW1lX2J3KCkrdGhlbWUodGV4dD1lbGVtZW50X3RleHQoc2l6ZT04KSwgbGVnZW5kLnBvc2l0aW9uID0gIm5vbmUiKSt5bGFiKCJUb3RhbCBSZWFkcyIpK3hsYWIoIkhJViBTdGF0dXMiKStmYWNldF93cmFwKH5zdGF0X3R5cGUpK3NjYWxlX2ZpbGxfbWFudWFsKHZhbHVlcz1oaXYuY29scykrc3RhdF9rcnVza2FsX3Rlc3Qoc2l6ZSA9IDIsIHZqdXN0ID0gMC44KQpnZ3NhdmUocGFzdGUoIkFuYWx5c2lzX0ZpZ3VyZXMvU2FtcGxlX2NvdmVyYWdlX2J5X0hJVl8iLCB0b2RheTIsICIudGlmZiIsIHNlcD0iIiksIGhlaWdodD01LCB3aWR0aD04KQoKZ2dwbG90KHRvLnBsb3RbdG8ucGxvdCRzdGF0X3R5cGUgJWluJSBjKCJSZWFkc19kZWxpdmVyZWQiLCAiaG9zdF9yZW1vdmVkIiwgInJSTkFfcmVtb3ZlZCIsICJxdWFsaXR5X2ZpbHRlcmVkIiksIF0sIGFlcyh4PXByZV9wb3N0LCB5PWFzLm51bWVyaWMocmVhZHMpLCBmaWxsPXByZV9wb3N0KSkrZ2VvbV9ib3hwbG90KGx3ZD0wLjEsIG5vdGNoID0gVCwgb3V0bGllci5zaGFwZSA9IE5BKStnZW9tX2ppdHRlcih3aWR0aD0wLjEsIHNpemU9MC4yKStzY2FsZV95X2xvZzEwKCkrdGhlbWVfYncoKSt0aGVtZSh0ZXh0PWVsZW1lbnRfdGV4dChzaXplPTgpLCBsZWdlbmQucG9zaXRpb24gPSAibm9uZSIpK3lsYWIoIlRvdGFsIFJlYWRzIikreGxhYigiU3dhYiIpK2ZhY2V0X3dyYXAofnN0YXRfdHlwZSkrc2NhbGVfZmlsbF9tYW51YWwodmFsdWVzPWMoImJsdWUiLCAiZGFya2JsdWUiKSkrc3RhdF9rcnVza2FsX3Rlc3Qoc2l6ZSA9IDIsIHZqdXN0ID0gMC44KQpnZ3NhdmUocGFzdGUoIkFuYWx5c2lzX0ZpZ3VyZXMvU2FtcGxlX2NvdmVyYWdlX2J5X3N3YWJfIiwgdG9kYXkyLCAiLnRpZmYiLCBzZXA9IiIpLCBoZWlnaHQ9NSwgd2lkdGg9OCkKCmdncGxvdCh0by5wbG90W3RvLnBsb3Qkc3RhdF90eXBlICVpbiUgYygiUmVhZHNfZGVsaXZlcmVkIiwgImhvc3RfcmVtb3ZlZCIsICJyUk5BX3JlbW92ZWQiLCAicXVhbGl0eV9maWx0ZXJlZCIpLCBdLCBhZXMoeD1zdGF0X3R5cGUsIHk9YXMubnVtZXJpYyhyZWFkcykpKStnZW9tX2JveHBsb3QobHdkPTAuMSwgbm90Y2ggPSBULCBvdXRsaWVyLnNoYXBlID0gTkEpK2dlb21faml0dGVyKHdpZHRoPTAuMSwgc2l6ZT0wLjIpK3RoZW1lX2J3KCkrdGhlbWUodGV4dD1lbGVtZW50X3RleHQoc2l6ZT04KSwgbGVnZW5kLnBvc2l0aW9uID0gIm5vbmUiKSt5bGFiKCJUb3RhbCBSZWFkcyIpK3hsYWIoIiIpK3N0YXRfbWVhbl9zZF90ZXh0KHNpemUgPSAyLCB2anVzdCA9IDAuOCkKZ2dzYXZlKHBhc3RlKCJBbmFseXNpc19GaWd1cmVzL1NhbXBsZV9jb3ZlcmFnZV8iLCB0b2RheTIsICIudGlmZiIsIHNlcD0iIiksIGhlaWdodD01LCB3aWR0aD04KQpgYGAKTm90ZTogVGhlcmUgYXJlIG1vcmUgaG9zdC1yZW1vdmVkIHJlYWRzIGluIHRoZSBISVYtTmVnIHNhbXBsZXMuIFRoaXMgaXMgbGlrZWx5IGJlY2F1c2UgbW9yZSBvZiB0aGVzZSBzYW1wbGVzIHdlcmUgbGFjdG9iYWNpbGx1cy1wcmVkb21pbmF0ZWQgY29tbXVuaXRpZXMgaW4gSElWLU5lZyBzYW1wbGVzIHdoaWNoIGhhdmUgYmVlbiBzaG93biB0byBjb250YWluIHJlbGF0aXZlbHkgbW9yZSBodW1hbiByZWFkcyB0aGFuIENTVCBJViBzYW1wbGVzIChDaXRlIE1hIGV0IGFsLiAoVklSR08pKSAKCiMjIyBDb250cm9scwpgYGB7cn0KbWc8LXJlYWQuY3N2KCIyME1hcjIwMjMvbm9ybV9jb3VudHNfbWdTc19tZ0NTVF8yME1hcjIwMjMuY3N2IiwgaGVhZGVyPVQsIHN0cmluZ3NBc0ZhY3RvcnMgPSBGKQpuYW1lcyhtZylbMV08LSJzYW1wbGVJRCIKbjwtd2hpY2gobmFtZXMobWcpICVpbiUgIlN0cmVwdG9jb2NjdXNfYWdhbGFjdGlhZV8xIikKY291bnRzPC1tZ1ssYygyOm4pXQpjb3VudHMkbWFyeWxhbmRfSUQ8LW1nJHNhbXBsZUlECiM0MDE4MjVfTkVHIHdhcyBkZXRlcm1pbmVkIHRvIGFjdHVhbGx5IGJlIGEgcG9zaXRpdmUgY29udHJvbC4KY291bnRzJG1hcnlsYW5kX0lEW2NvdW50cyRtYXJ5bGFuZF9JRCAlaW4lICI0MDE4MjVfTkVHIl08LSI0MDE4MjVfWllNT19QT1MiCnJvd25hbWVzKGNvdW50cyk8LWNvdW50cyRtYXJ5bGFuZF9JRAoKY291bnRzLm08LXJlc2hhcGUyOjptZWx0KGNvdW50cywgaWQudmFycz0ibWFyeWxhbmRfSUQiLCB2YXJpYWJsZS5uYW1lID0gInRheGEiLCB2YWx1ZS5uYW1lID0gImNvdW50cyIpCnRvdGFsLmNvdW50czwtYXMuZGF0YS5mcmFtZShjb3VudHMubSAlPiUgZ3JvdXBfYnkobWFyeWxhbmRfSUQpICU+JSBkcGx5cjo6c3VtbWFyaXNlKHRvdGFsX2NvdW50cz1zdW0oY291bnRzKSkpCnRvdGFsLnNwZWNpZXM8LWFzLmRhdGEuZnJhbWUoY2JpbmQodG90YWxfc3BlY2llcz1hcHBseShjb3VudHMsIDEsIGZ1bmN0aW9uKHgpIHN1bSh4ID49IDAuNSkpLCBtYXJ5bGFuZF9JRD1yb3duYW1lcyhjb3VudHMpKSkKY291bnRzJG1hcnlsYW5kX0lEPC1OVUxMCgojIyBQT1NJVElWRSBDT05UUk9MUyAodmFnaW5hICsgWllNTykKcG9zLmN0cmxzPC1jb3VudHMubVtncmVwbCgicG9zIiwgY291bnRzLm0kbWFyeWxhbmRfSUQsIGlnbm9yZS5jYXNlID0gVCksIF0KdG9wLnRheGE8LWFzLmRhdGEuZnJhbWUocG9zLmN0cmxzW3Bvcy5jdHJscyRjb3VudHMgPiAwLCBdICU+JSBncm91cF9ieSh0YXhhKSAlPiUgZHBseXI6OnN1bW1hcmlzZShtZWFuPW1lYW4oY291bnRzKSkpCgp0b3AudGF4YTwtYXMuZGF0YS5mcmFtZShwb3MuY3RybHNbcG9zLmN0cmxzJGNvdW50cyA+IDAsIF0gJT4lIGRwbHlyOjpncm91cF9ieSh0YXhhKSAlPiUgZHBseXI6OnN1bW1hcmlzZShtZWFuPW1lYW4oY291bnRzKSkpCmN0cmwudGF4YS5jb2xzPC1hcy5kYXRhLmZyYW1lKGNiaW5kKHRheGE9YXMudmVjdG9yKHRvcC50YXhhW3RvcC50YXhhJG1lYW4gPiAxZTQsICJ0YXhhIl0pLCBjb2xvcj1jKFJDb2xvckJyZXdlcjo6YnJld2VyLnBhbCgxMiwiU2V0MyIpLCBSQ29sb3JCcmV3ZXI6OmJyZXdlci5wYWwoMTIsIlBhaXJlZCIpLCBSQ29sb3JCcmV3ZXI6OmJyZXdlci5wYWwoMywiU2V0MSIpKSkpCnRvcC50YXhhPC1hcy52ZWN0b3IodG9wLnRheGFbdG9wLnRheGEkbWVhbiA+IDFlNCwgInRheGEiXSkKcG9zLmN0cmxzPC1wb3MuY3RybHNbcG9zLmN0cmxzJHRheGEgJWluJSB0b3AudGF4YSwgXQpjb2xzPC1jdHJsLnRheGEuY29sc1ttYXRjaCh0b3AudGF4YSwgY3RybC50YXhhLmNvbHMkdGF4YSksICJjb2xvciJdCmdncGxvdChwb3MuY3RybHMsIGFlcyh4PW1hcnlsYW5kX0lELCB5PWNvdW50cywgZmlsbD10YXhhKSkrZ2VvbV9iYXIoc3RhdD0iaWRlbnRpdHkiKSt0aGVtZV9idygpK3RoZW1lKGxlZ2VuZC5wb3NpdGlvbiA9ICJib3R0b20iLCBsZWdlbmQudGV4dCA9IGVsZW1lbnRfdGV4dChzaXplPTYpLCBsZWdlbmQudGl0bGUgPSBlbGVtZW50X2JsYW5rKCksIGF4aXMudGV4dC54ID0gZWxlbWVudF90ZXh0KHNpemU9NSkpK3hsYWIoIiIpK3lsYWIoIlRvdGFsIENvcnJlY3RlZCBSZWFkcyIpK3NjYWxlX2ZpbGxfbWFudWFsKHZhbHVlcz1jb2xzKStnZ3RpdGxlKCJUYXhhIG9ic2VydmVkIGluIHBvc2l0aXZlIGNvbnRyb2xzIHdpdGggPj0gMWU0IHJlYWRzIikKZ2dzYXZlKHBhc3RlKCJBbmFseXNpc19GaWd1cmVzL1Bvc19jdHJsc18iLCB0b2RheTIsICIudGlmZiIsIHNlcD0iIiksIGhlaWdodCA9IDUsIHdpZHRoPTcpCiMjIGNvbXBvc2l0aW9uYWxseSBzaW1pbGFyLCBidXQgdGhlIHRvdGFsIG51bWJlciBvZiByZWFkcyBwZXIgcGxhdGUgZGlmZmVyIChjb3ZlcmFnZSBieSBwbGF0ZSkKYXMuZGF0YS5mcmFtZShwb3MuY3RybHMgJT4lIGdyb3VwX2J5KG1hcnlsYW5kX0lEKSAlPiUgZHBseXI6OnN1bW1hcmlzZShzdW0oY291bnRzKSkpCgojIyBORUdBVElWRSBDT05UUk9MUwpuZWcuY3RybHM8LWNvdW50cy5tW2dyZXBsKCJuZWciLCBjb3VudHMubSRtYXJ5bGFuZF9JRCwgaWdub3JlLmNhc2UgPSBUKSwgXQp0b3AudGF4YTwtYXMuZGF0YS5mcmFtZShuZWcuY3RybHNbbmVnLmN0cmxzJGNvdW50cyA+IDAsIF0gJT4lIGdyb3VwX2J5KHRheGEpICU+JSBkcGx5cjo6c3VtbWFyaXNlKG1lYW49bWVhbihjb3VudHMpLCBuPWxlbmd0aCh1bmlxdWUobWFyeWxhbmRfSUQpKSkpCnRvLnJtPC1hcy52ZWN0b3IodG9wLnRheGFbdG9wLnRheGEkbiA9PSAzICYgdG9wLnRheGEkbWVhbiA+IDMwMCwgInRheGEiXSkKCmdncGxvdChuZWcuY3RybHMsIGFlcyh4PW1hcnlsYW5kX0lELCB5PWNvdW50cywgZmlsbD10YXhhKSkrZ2VvbV9iYXIoc3RhdD0iaWRlbnRpdHkiKSt0aGVtZV9idygpK3RoZW1lKGxlZ2VuZC5wb3NpdGlvbiA9ICJub25lIiwgbGVnZW5kLnRleHQgPSBlbGVtZW50X3RleHQoc2l6ZT02KSwgbGVnZW5kLmtleS5zaXplID0gdW5pdCgwLjUsImxpbmUiKSwgbGVnZW5kLnRpdGxlID0gZWxlbWVudF9ibGFuaygpLCBheGlzLnRleHQueCA9IGVsZW1lbnRfdGV4dChzaXplPTUpKSt4bGFiKCIiKSt5bGFiKCJUb3RhbCBDb3JyZWN0ZWQgUmVhZHMiKStnZ3RpdGxlKCJUYXhhIG9ic2VydmVkIGluIG5lZ2F0aXZlIGNvbnRyb2xzIHdpdGggPj0gMTAwIHJlYWRzIikjK2d1aWRlcyhmaWxsPWd1aWRlX2xlZ2VuZChucm93PTMsYnlyb3c9VFJVRSkpCmdnc2F2ZShwYXN0ZSgiQW5hbHlzaXNfRmlndXJlcy9OZWdfY3RybHNfIiwgdG9kYXkyLCAiLnRpZmYiLCBzZXA9IiIpLCBoZWlnaHQgPSA1LCB3aWR0aD03KQoKYXMuZGF0YS5mcmFtZShuZWcuY3RybHMgJT4lIGdyb3VwX2J5KG1hcnlsYW5kX0lEKSAlPiUgZHBseXI6OnN1bW1hcmlzZShzdW0oY291bnRzKSkpCgojIyBnZXQgdmVjdG9yIG9mIGFsbCBjb250cm9sIG5hbWVzIHRvIHJlbW92ZSBsYXRlci4KY3RybHM8LWFzLnZlY3Rvcih1bmlxdWUoYyhwb3MuY3RybHMkbWFyeWxhbmRfSUQsIG5lZy5jdHJscyRtYXJ5bGFuZF9JRCkpKQpgYGAKTmVnYXRpdmUgY29udHJvbHMgaGF2ZSAxMi01MmsgcmVhZHMgYW5kIGNvbnRhbWluYXRpbmcgRE5BIGZyb206CiJFc2NoZXJpY2hpYV9jb2xpIiAgICAgICAgICAgCiJQcmV2b3RlbGxhX3RpbW9uZW5zaXNfMCIgICAgCiJQcm9waW9uaWJhY3Rlcml1bV9zcC4iICAgICAgCiJTYWxtb25lbGxhX2VudGVyaWNhIiAgICAgICAgCiJTbmVhdGhpYV9hbW5paV8wIiAgICAgICAgICAKIlN0YXBoeWxvY29jY3VzX2VwaWRlcm1pZGlzIgoKCiMjIENvdW50IFRhYmxlcwpgYGB7cn0KIyMgbm9ybSBzcHAuIGNvdW50cyB3L21nU3MKY291bnRzPC1jb3VudHNbLCAhbmFtZXMoY291bnRzKSAlaW4lIHRvLnJtXSAjIyByZW1vdmUgY29udGFtaW5hbnRzCgojIyByZWxhYnVuZApyZWxhYnVuZDwtY291bnRzL3Jvd1N1bXMoY291bnRzKQpyZWxhYnVuZCRtYXJ5bGFuZF9JRDwtcm93bmFtZXMoY291bnRzKQpyZWxhYnVuZC5tPC1yZXNoYXBlMjo6bWVsdChyZWxhYnVuZCwgaWQudmFycz0ibWFyeWxhbmRfSUQiLCB2YXJpYWJsZS5uYW1lID0gInRheGEiLCB2YWx1ZS5uYW1lID0gInJlbGFidW5kIikKCmNvdW50cyRtYXJ5bGFuZF9JRDwtcm93bmFtZXMoY291bnRzKQpjb3VudHMubTwtcmVzaGFwZTI6Om1lbHQoY291bnRzLCBpZC52YXJzPSJtYXJ5bGFuZF9JRCIsIHZhcmlhYmxlLm5hbWUgPSAidGF4YSIsIHZhbHVlLm5hbWUgPSAiY291bnRzIikKCiMjIG1lcmdlCmNvdW50cy5tPC1tZXJnZShjb3VudHMubSwgcmVsYWJ1bmQubSwgYWxsPVRSVUUpCmNvdW50cy5tJHRheGE8LWdzdWIoIlxcLl8iLCAiXFwuIiwgY291bnRzLm0kdGF4YSkKY291bnRzLm08LWNvdW50cy5tW2NvdW50cy5tJG1hcnlsYW5kX0lEICVpbiUgc2FtcGxlLmxpc3QsIF0KY291bnRzLm08LWNvdW50cy5tW2NvbXBsZXRlLmNhc2VzKGNvdW50cy5tKSwgXQpjb3VudHMubSRjb3ZlcmFnZTwtcmVhZC5zdGF0c1ttYXRjaChjb3VudHMubSRtYXJ5bGFuZF9JRCwgcmVhZC5zdGF0cyRzYW1wbGUpLCAiUmVhZHNfZGVsaXZlcmVkIl0KCiMjIHdyaXRlIG91dCBmb3IgVkFMRU5DSUEKdG8ud3JpdGU8LXJlYWQuZGVsaW0oIk1HIHByb2Nlc3Npbmcvc3VtbWFyeS5BYnVuZGFuY2UudHh0IiwgaGVhZGVyPVQsIHN0cmluZ3NBc0ZhY3RvcnMgPSBGLCBjaGVjay5uYW1lcyA9IEYpCm5hbWVzKHRvLndyaXRlKVsxXTwtInNhbXBsZUlEIgp0by53cml0ZSRzYW1wbGVJRFt0by53cml0ZSRzYW1wbGVJRCAlaW4lICI0MDE4MjVfTkVHIl08LSI0MDE4MjVfWllNT19QT1MiCnRvLndyaXRlPC10by53cml0ZVshdG8ud3JpdGUkc2FtcGxlSUQgJWluJSB1bmlxdWUoY3RybHMpLCFuYW1lcyh0by53cml0ZSkgJWluJSB0by5ybV0gIyMgcm0gdGF4YSBmcm9tIG5lZ2F0aXZlIGNvbnRyb2xzCnRvLndyaXRlWywyOm5jb2wodG8ud3JpdGUpXTwtcm91bmQodG8ud3JpdGVbLDI6bmNvbCh0by53cml0ZSldKQpsPC1hcy5kYXRhLmZyYW1lKGNiaW5kKHNhbXBsZUlEPXRvLndyaXRlJHNhbXBsZUlELCByZWFkX2NvdW50PXJvdW5kKHJvd1N1bXModG8ud3JpdGVbLDI6bmNvbCh0by53cml0ZSldKSkpKQp0by53cml0ZTwtbWVyZ2UobCwgdG8ud3JpdGUsIGFsbCA9IFQpCndyaXRlLmNzdih0by53cml0ZSwgIjIwTWFyMjAyMy9jb3VudHNfZm9yX1ZBTEVOQ0lBLmNzdiIsIHJvdy5uYW1lcyA9IEYsIHF1b3RlPUYpCmBgYAoKCiMjIyBSZWFkIGluIEdlbmUgY291bnRzIChub3JtYWxpemVkIGJ5IGdlbmUgbGVuZ3RoKQpgYGB7cn0KIyMgbm9ybSBnZW5lIGNvdW50cwpnZW5lczwtcmVhZC5kZWxpbSgiMjBNYXIyMDIzL3N1bW1hcnkuTlIuYWJ1bmRhbmNlLnR4dCIsIGNoZWNrLm5hbWVzID0gRikKbmFtZXMoZ2VuZXMpW25hbWVzKGdlbmVzKSAlaW4lICI0MDE4MjVfTkVHIl08LSI0MDE4MjVfWllNT19QT1MiCnJvd25hbWVzKGdlbmVzKTwtZ2VuZXMkc2FtcGxlSUQKZ2VuZXMkc2FtcGxlSUQ8LU5VTEwKYGBgCgojIyMgU3VtbWFyeSBTdGF0cwpgYGB7cn0KdG90YWwuc2FtcC5uR2VuZXM8LWFzLmRhdGEuZnJhbWUoY2JpbmQodG90YWxfZ2VuZXM9YXBwbHkoZ2VuZXMsIE1BUkdJTiA9IDIsIGZ1bmN0aW9uKHgpIHN1bSh4Pj0wLjUpKSwgbWFyeWxhbmRfSUQ9bmFtZXMoZ2VuZXMpKSkKdG90YWwuc2FtcC5jb3VudHM8LWFzLmRhdGEuZnJhbWUoY291bnRzLm0gJT4lIGdyb3VwX2J5KG1hcnlsYW5kX0lEKSAlPiUgZHBseXI6OnN1bW1hcmlzZSh0b3RhbF9jb3VudHM9c3VtKGNvdW50cykpKQp0b3RhbC5zYW1wLnNwZWNpZXM8LWFzLmRhdGEuZnJhbWUoY2JpbmQodG90YWxfc3BlY2llcz1hcHBseShjb3VudHMsIDEsIGZ1bmN0aW9uKHgpIHN1bSh4ID49IDAuNSkpLCBtYXJ5bGFuZF9JRD1yb3duYW1lcyhjb3VudHMpKSkKCnNhbXBsZS5zdW1tYXJ5PC1tZXJnZSh0b3RhbC5zYW1wLm5HZW5lcywgdG90YWwuc2FtcC5jb3VudHMsIGFsbD1UUlVFKQpzYW1wbGUuc3VtbWFyeTwtbWVyZ2Uoc2FtcGxlLnN1bW1hcnksIHRvdGFsLnNhbXAuc3BlY2llcywgYWxsPVRSVUUpCnNhbXBsZS5zdW1tYXJ5PC1tZXJnZShzYW1wbGUuc3VtbWFyeSwgcmVhZC5zdGF0cywgYWxsLng9VFJVRSwgYnkueT0ic2FtcGxlIiwgYnkueD0ibWFyeWxhbmRfSUQiKQpgYGAKCiMjIyMjIyAgUGxvdHMKYGBge3J9CgojRG9lcyB0b3RhbCBzcGVjaWVzIGNvcnJlc3BvbmQgdG8gc2VxdWVuY2luZyBkZXB0aD8gCmdncGxvdChzYW1wbGUuc3VtbWFyeSwgYWVzKHg9bG9nMTAoYXMubnVtZXJpYyh0b3RhbF9zcGVjaWVzKSksIHk9bG9nMTAoUmVhZHNfZGVsaXZlcmVkKSkpK2dlb21fcG9pbnQoKStnZW9tX3Ntb290aChtZXRob2Q9ImxtIikrc3RhdF9wb2x5X2VxKCkgIyBObwoKI0RvZXMgdG90YWwgZ2VuZXMgY29ycmVzcG9uZCB0byBzZXF1ZW5jaW5nIGRlcHRoPwpnZ3Bsb3Qoc2FtcGxlLnN1bW1hcnksIGFlcyh4PWxvZzEwKGFzLm51bWVyaWModG90YWxfZ2VuZXMpKSwgeT1sb2cxMChSZWFkc19kZWxpdmVyZWQpKSkrZ2VvbV9wb2ludCgpK2dlb21fc21vb3RoKG1ldGhvZD0ibG0iKStzdGF0X3BvbHlfZXEoKSAjIFNsaWdodCBwb3NpdGl2ZSwgYnV0IFIyIDwgMC4xCmBgYAoKCgpjZCAyME1hcjIwMjMKCnB5dGhvbjMgfi9iaW4vVkFMRU5DSUEvVmFsZW5jaWEucHkgLXJlZiB+L2Jpbi9zcGVjaWF0ZUlUL1ZBTEVOQ0lBMl9DU1RfY2VudHJvaWRzXzIwTWFyMjAyNC5jc3YgLWkgY291bnRzX2Zvcl9WQUxFTkNJQS5jc3YgLW8gTUxBV0lfQ1NUCgpSc2NyaXB0IH4vYmluL21nQ1NULWNsYXNzaWZpZXIvY2xhc3NpZnlfbWdDU1RfY2VudHJvaWQuUiAvVXNlcnMvam9oYW5uYWhvbG0vSUdTXCBEcm9wYm94L0pvaGFubmFcIEhvbG0vTVNML01MQVdJLzIwTWFyMjAyMy9zdW1tYXJ5LkFidW5kYW5jZS50eHQgL1VzZXJzL2pvaGFubmFob2xtL0lHU1wgRHJvcGJveC9Kb2hhbm5hXCBIb2xtL01TTC9NTEFXSS8yME1hcjIwMjMvc3VtbWFyeS5OUi5hYnVuZGFuY2UudHh0IH4vYmluL1ZJUkdPLW1hc3Rlci8gfi9iaW4vbWdDU1QtY2xhc3NpZmllci8KCgojIyBMb2FkIGFuZCBwcmVwYXJlIHNlcXVlbmNpbmcsIG1nQ1NULCBhbmQgQ1NUIGRhdGEgZm9yIGFuYWx5c2VzCmBgYHtyfQojIyBtZ0NTVHMKbWdDU1RzPC1yZWFkLmNzdigiMjBNYXIyMDIzL21nQ1NUc18yNkp1bjIwMjQuY3N2IikKbmFtZXMobWdDU1RzKVsxXTwtIm1hcnlsYW5kX0lEIgojbWdDU1RzPC1tZ0NTVHNbbWdDU1RzJG1hcnlsYW5kX0lEICVpbiUgdW5pcXVlKGFsbC5zYW1wbGVzJG1hcnlsYW5kX0lEKSwgXQptZ0NTVHMkbWdDU1Q8LWdzdWIoIm1nQ1NUICIsICIiLCBtZ0NTVHMkbWdDU1QpCm1nQ1NUcyRtZ0NTVDwtZmFjdG9yKG1nQ1NUcyRtZ0NTVCwgbGV2ZWxzPXVuaXF1ZShzb3J0KGFzLm51bWVyaWMobWdDU1RzJG1nQ1NUKSkpLCBvcmRlcmVkID0gRikKCiMjIFZBTEVOQ0lBIGNzdHMKQ1NUczwtcmVhZC5jc3YoIjIwTWFyMjAyMy9NTEFXSV9DU1QuY3N2IikKbmFtZXMoQ1NUcylbMV08LSJtYXJ5bGFuZF9JRCIKQ1NUczwtQ1NUc1ssYygibWFyeWxhbmRfSUQiLCAiQ1NUIiwgInNjb3JlIildCkNTVHM8LUNTVHNbQ1NUcyRtYXJ5bGFuZF9JRCAlaW4lIHVuaXF1ZShhbGwuc2FtcGxlcyRtYXJ5bGFuZF9JRCksIF0KQ1NUcyRDU1Q8LWZhY3RvcihDU1RzJENTVCwgbGV2ZWxzPXNvcnQodW5pcXVlKENTVHMkQ1NUKSksIG9yZGVyZWQgPSBGKQpDU1RzJGZpbmFsUmVhZHNfcGVfc2U8LXNhbXBsZS5zdW1tYXJ5W21hdGNoKENTVHMkbWFyeWxhbmRfSUQsIHNhbXBsZS5zdW1tYXJ5JG1hcnlsYW5kX0lEKSwgImZpbmFsUmVhZHNfcGVfc2UiXQpgYGAKCiMjIyMjIyBVcGRhdGUgbWdTcwpgYGB7cn0KIyMgR2V0IHJpZCBvZiBtZ1NzIGZvciBiZWxvdzogCiMjIGZldyBzYW1wbGVzOiAKY291bnRzLm0kdGF4YTI8LWlmZWxzZShncmVwbCgiQ2xvc3RyaWRpYWxlc19GYW1pbHkiLCBjb3VudHMubSR0YXhhKSwgIkNsb3N0cmlkaWFsZXNfRmFtaWx5IiwgYXMuY2hhcmFjdGVyKGNvdW50cy5tJHRheGEpKQpjb3VudHMubSR0YXhhMjwtaWZlbHNlKGdyZXBsKCJFbnRlcm9jb2NjdXNfZmFlY2FsaXMiLCBjb3VudHMubSR0YXhhKSwgIkVudGVyb2NvY2N1c19mYWVjYWxpcyIsIGFzLmNoYXJhY3Rlcihjb3VudHMubSR0YXhhMikpCmNvdW50cy5tJHRheGEyPC1pZmVsc2UoZ3JlcGwoIkxhY3RvYmFjaWxsdXNfZ2Fzc2VyaSIsIGNvdW50cy5tJHRheGEpLCAiTGFjdG9iYWNpbGx1c19nYXNzZXJpIiwgYXMuY2hhcmFjdGVyKGNvdW50cy5tJHRheGEyKSkKY291bnRzLm0kdGF4YTI8LWlmZWxzZShncmVwbCgiTGFjdG9iYWNpbGx1c19qZW5zZW5paSIsIGNvdW50cy5tJHRheGEpLCAiTGFjdG9iYWNpbGx1c19qZW5zZW5paSIsIGFzLmNoYXJhY3Rlcihjb3VudHMubSR0YXhhMikpCmNvdW50cy5tJHRheGEyPC1pZmVsc2UoZ3JlcGwoIk1vYmlsdW5jdXNfbXVsaWVyaXMiLCBjb3VudHMubSR0YXhhKSwgIk1vYmlsdW5jdXNfbXVsaWVyaXMiLCBhcy5jaGFyYWN0ZXIoY291bnRzLm0kdGF4YTIpKQpjb3VudHMubSR0YXhhMjwtaWZlbHNlKGdyZXBsKCJNb2JpbHVuY3VzX2N1cnRpc2lpIiwgY291bnRzLm0kdGF4YSksICJNb2JpbHVuY3VzX2N1cnRpc2lpIiwgYXMuY2hhcmFjdGVyKGNvdW50cy5tJHRheGEyKSkKY291bnRzLm0kdGF4YTI8LWlmZWxzZShncmVwbCgiTXljb3BsYXNtYV9ob21pbmlzIiwgY291bnRzLm0kdGF4YSksICJNeWNvcGxhc21hX2hvbWluaXMiLCBhcy5jaGFyYWN0ZXIoY291bnRzLm0kdGF4YTIpKQpjb3VudHMubSR0YXhhMjwtaWZlbHNlKGdyZXBsKCJQZXB0b3N0cmVwdG9jb2NjdXNfYW5hZXJvYml1cyIsIGNvdW50cy5tJHRheGEpLCAiUGVwdG9zdHJlcHRvY29jY3VzX2FuYWVyb2JpdXMiLCBhcy5jaGFyYWN0ZXIoY291bnRzLm0kdGF4YTIpKQpjb3VudHMubSR0YXhhMjwtaWZlbHNlKGdyZXBsKCJQb3JwaHlyb21vbmFzX3Vlbm9uaXMiLCBjb3VudHMubSR0YXhhKSwgIlBvcnBoeXJvbW9uYXNfdWVub25pcyIsIGFzLmNoYXJhY3Rlcihjb3VudHMubSR0YXhhMikpCmNvdW50cy5tJHRheGEyPC1pZmVsc2UoZ3JlcGwoIlN0cmVwdG9jb2NjdXNfYWdhbGFjdGlhZSIsIGNvdW50cy5tJHRheGEpLCAiU3RyZXB0b2NvY2N1c19hZ2FsYWN0aWFlIiwgYXMuY2hhcmFjdGVyKGNvdW50cy5tJHRheGEyKSkKY291bnRzLm08LWNvdW50cy5tWyFjb3VudHMubSR0YXhhMiAlaW4lIHRvLnJtLCBdCgpjb3VudHMubSRzcHA8LXBhc3RlKHN0cl9zcGxpdF9maXhlZChjb3VudHMubSR0YXhhMiwgcGF0dGVybiA9ICJfIiwgbiA9IDMpWywxXSwgc3RyX3NwbGl0X2ZpeGVkKGNvdW50cy5tJHRheGEyLCBwYXR0ZXJuID0gIl8iLCBuID0gMylbLDJdLCBzZXA9Il8iKQpjb3VudHMubSRtZ3NzPC1zdHJfc3BsaXRfZml4ZWQoY291bnRzLm0kdGF4YTIsIHBhdHRlcm4gPSAiXyIsIG4gPSAzKVssM10KY291bnRzLm0kbWdzczwtZmFjdG9yKGNvdW50cy5tJG1nc3MsIGxldmVscz1jKDA6MTApLCBvcmRlcmVkPUYpCgptZ3NzPC11bmlxdWUoY291bnRzLm0kdGF4YTJbZ3JlcGwoIl9bMC05XSQiLCBjb3VudHMubSR0YXhhKV0pCnNwcDwtdW5pcXVlKHBhc3RlKHN0cl9zcGxpdF9maXhlZChtZ3NzLCBwYXR0ZXJuID0gIl8iLCBuID0gMylbLDFdLCBzdHJfc3BsaXRfZml4ZWQobWdzcywgcGF0dGVybiA9ICJfIiwgbiA9IDMpWywyXSwgc2VwPSJfIikpCmBgYAoKIyMjIyMjIFVwZGF0ZSAmIE1lcmdlIG1nQ1NUcwpgYGB7ciwgd2FybmluZz1GQUxTRX0KbWdDU1QuY29sPC1hcy5kYXRhLmZyYW1lKHJiaW5kKGMoIjEiLCAiI0ZFMDMwOCIpLGMoIjIiLCAiI0Y1NEM1RSIpLCBjKCIzIiwgIiNGMDcwODQiKSwgYygiNCIsICIjRUM5NEE1IiksYygiNSIsICIjRjBCQ0NDIiksYygiNiIsICIjRjZEM0RBIiksYygiNyIsICIjODZDNjFBIiksIGMoIjgiLCAiI0I0REIyOSIpLGMoIjkiLCAiI0RCRUE3NyIpLCBjKCIxMCIsICIjRkY3MjAwIiksIGMoIjExIiwgIiNGNjhBMTEiKSxjKCIxMiIsICIjRjhBNDBFIiksYygiMTMiLCAiI0YzQkMxMSIpLGMoIjE0IiwgIiNmN2QxNWEiKSwgYygiMTUiLCAiI0ZBRTUwRCIpLGMoIjE2IiwgIiNGM0Y0NkUiKSxjKCIxNyIsICIjNDQ4QTczIiksYygiMTgiLCAiIzg5QkVBQiIpLCBjKCIxOSIsICIjQkNENkNEIiksYygiMjAiLCAiIzIyMTg4NiIpLGMoIjIxIiwgIiMzRTM3OTIiKSxjKCIyMiIsICIjNUQ1NzlFIiksYygiMjMiLCAiIzdDNzZBQyIpLGMoIjI0IiwgIiM5QTk4QkYiKSxjKCIyNSIsICIjQzlDOEQ4IiksYygiMjYiLCAiIzk4Qzk5OSIpLCBjKCIyNyIsICIjOTg5ODk4IiksIGMoIiIsICJ3aGl0ZSIpLCBjKCJOQSIsICJ3aGl0ZSIpKSkKbmFtZXMobWdDU1QuY29sKTwtYygibWdDU1QiLCAiY29sb3IiKQoKdGFibGUobWdDU1RzJG1nQ1NUKQptZ0NTVHMkbWdDU1QyPC1pZmVsc2UobWdDU1RzJG1nQ1NUICVpbiUgMTo2LCA0LCBhcy5jaGFyYWN0ZXIobWdDU1RzJG1nQ1NUKSkKbWdDU1RzJG1nQ1NUMjwtaWZlbHNlKG1nQ1NUcyRtZ0NTVDIgJWluJSAxMywgMjcsIGFzLmNoYXJhY3RlcihtZ0NTVHMkbWdDU1QyKSkKbWdDU1RzJG1nQ1NUMjwtaWZlbHNlKG1nQ1NUcyRtZ0NTVDIgJWluJSAyMSwgMjAsIGFzLmNoYXJhY3RlcihtZ0NTVHMkbWdDU1QyKSkKbWdDU1RzJG1nQ1NUMjwtaWZlbHNlKG1nQ1NUcyRtZ0NTVDIgJWluJSAxNzoxOSwgMTcsIGFzLmNoYXJhY3RlcihtZ0NTVHMkbWdDU1QyKSkKbDwtYXMuZGF0YS5mcmFtZShjYmluZCh0b3BfdGF4YT1jb2xuYW1lcyhjb3VudHMpW2FwcGx5KGNvdW50cywxLHdoaWNoLm1heCldLCBtYXJ5bGFuZF9JRD1yb3duYW1lcyhjb3VudHMpKSkKbCR0b3BfdGF4YTwtZ3N1YigiLy8uXyIsICIvLy4iLCBsJHRvcF90YXhhKQptZ0NTVHM8LW1lcmdlKG1nQ1NUcywgbCwgYWxsLng9VFJVRSkKbWdDU1RzJG1nQ1NUMjwtaWZlbHNlKG1nQ1NUcyRtZ0NTVDIgJWluJSAyNyAmIG1nQ1NUcyR0b3BfdGF4YSAlaW4lICJMYWN0b2JhY2lsbHVzX2luZXJzXzQiLCAxMSwgYXMuY2hhcmFjdGVyKG1nQ1NUcyRtZ0NTVDIpKQp0YWJsZShtZ0NTVHMkbWdDU1QyKQoKIyMgYWRkIG1nQ1NUMiB0byBjb3VudHMubQpjb3VudHMubSRtZ0NTVDwtbWdDU1RzW21hdGNoKGNvdW50cy5tJG1hcnlsYW5kX0lELCBtZ0NTVHMkbWFyeWxhbmRfSUQpLCAibWdDU1QyIl0KY291bnRzLm0kbWdDU1Q8LWZhY3Rvcihjb3VudHMubSRtZ0NTVCwgbGV2ZWxzPXVuaXF1ZShzb3J0KGFzLm51bWVyaWMoY291bnRzLm0kbWdDU1QpKSksIG9yZGVyZWQgPSBGKQpgYGAKCiMjIyMjIFNwZWNpZXMKYGBge3J9CmNvdW50cy5tJHNwcDwtcGFzdGUoc3RyX3NwbGl0X2ZpeGVkKGNvdW50cy5tJHRheGEsIHBhdHRlcm4gPSAiXyIsIG4gPSAzKVssMV0sIHN0cl9zcGxpdF9maXhlZChjb3VudHMubSR0YXhhLCBwYXR0ZXJuID0gIl8iLCBuID0gMylbLDJdLCBzZXA9Il8iKQpjb3VudHMubSRtZ3NzPC1zdHJfc3BsaXRfZml4ZWQoY291bnRzLm0kdGF4YSwgcGF0dGVybiA9ICJfIiwgbiA9IDMpWywzXQptZ3NzPC11bmlxdWUoY291bnRzLm0kdGF4YVtncmVwbCgiX1swLTldJCIsIGNvdW50cy5tJHRheGEpXSkjIyB3aGljaCBzcGVjaWVzIGhhdmUgbWdTcwpzcHA8LXVuaXF1ZShwYXN0ZShzdHJfc3BsaXRfZml4ZWQobWdzcywgcGF0dGVybiA9ICJfIiwgbiA9IDMpWywxXSwgc3RyX3NwbGl0X2ZpeGVkKG1nc3MsIHBhdHRlcm4gPSAiXyIsIG4gPSAzKVssMl0sIHNlcD0iXyIpKQoKc3BwLnN1bW1hcnk8LWFzLmRhdGEuZnJhbWUoY291bnRzLm0gJT4lIGdyb3VwX2J5KHNwcCkgJT4lIGRwbHlyOjpzdW1tYXJpc2UobWVhblJlbGFidW5kPW1lYW4ocmVsYWJ1bmQpLCBtZWFuQ291bnRzPW1lYW4oY291bnRzKSwgbk1nc3M9bGVuZ3RoKHVuaXF1ZShtZ3NzKSkpKQptZ3NzLnN1bW1hcnk8LWFzLmRhdGEuZnJhbWUoY291bnRzLm0gJT4lIGdyb3VwX2J5KHNwcCwgdGF4YSkgJT4lIGRwbHlyOjpzdW1tYXJpc2UobWVhblJlbGFidW5kPW1lYW4ocmVsYWJ1bmQpLCBtZWFuQ291bnRzPW1lYW4oY291bnRzKSkpCmBgYAoKIyMjIyMjICBGSUcgUzE6IENvdmVyYWdlIGJ5IENTVCBBbmQgbWdTUwpgYGB7cn0KI0RvZXMgdGhlIG5HZW5lcyBwZXIgc3BlY2llcyBwZXIgc2FtcGxlIGRpZmZlciBieSBzZXF1ZW5jaW5nIGRlcHRoIG9yIHJlbGF0aXZlIGFidW5kYW5jZT8KI0lmIHRoZSBuR2VuZXMgcGVyIHNwZWNpZXMgZGlmZmVycyBieSBzZXF1ZW5jaW5nIGRlcHRoLCB0aGVuIG1nU3MgbGlrZWx5IHdvbid0IHdvcmssIGJjIG1nU3Mgd2lsbCBiZSBkZXBlbmRlbnQgb24gY292ZXJhZ2UuIElmIGl0IGRpZmZlcnMgYnkgcmVsYWJ1bmQsIHRoZSBtZ1NzIHdpbGwgd29yawojIyBEZXRlcm1pbmUgc3BlY2llcyBmb3Igd2hpY2ggbWdTcyB3aWxsIHdvcmsuCnAxPC1nZ3Bsb3QoQ1NUcywgYWVzKHg9ZmluYWxSZWFkc19wZV9zZSwgeT1zY29yZSkpKwogIGdlb21fcG9pbnQoc2l6ZT0wLjEpKwogIGZhY2V0X3dyYXAofkNTVCwgc2NhbGVzPSJmcmVlIikrCiAgc2NhbGVfeF9sb2cxMCgpKwogIHRoZW1lX2J3KCkrCiAgdGhlbWUobGVnZW5kLnBvc2l0aW9uID0gIm5vbmUiLCAKICAgICAgdGV4dD1lbGVtZW50X3RleHQoc2l6ZT0xLCBjb2xvcj0iYmxhY2siKSwgCiAgICAgIGxlZ2VuZC5rZXkuc2l6ZSA9IHVuaXQoMC4zLCAiY20iKSwgCiAgICAgIGxpbmUgPSBlbGVtZW50X2xpbmUobGluZXdpZHRoID0gMC4xKSwgCiAgICAgIGF4aXMudGl0bGUgPSBlbGVtZW50X3RleHQoc2l6ZT00KSwKICAgICAgc3RyaXAudGV4dCA9IGVsZW1lbnRfdGV4dChzaXplPTMpLAogICAgICBheGlzLnRleHQgPSBlbGVtZW50X3RleHQoc2l6ZT00LCBjb2xvcj0iYmxhY2siKSkrCiAgeWxhYigiWXVlLUNsYXl0b24gz7QiKSsKICB4bGFiKCJTZXF1ZW5jaW5nIERlcHRoIChsb2cxMCkiKQoKCnAyPC1nZ3Bsb3QoY291bnRzLm1bY291bnRzLm0kc3BwICVpbiUgc3BwWzE6MjVdICYgY291bnRzLm0kbWdzcyAlaW4lIGMoMToxMCkgJiBjb3VudHMubSRjb3VudHMgPiAwLCBdLCBhZXMoeD1yZW9yZGVyKHggPSBmYWN0b3IobWdzcyksIGFzLm51bWVyaWMobWdzcykpLCB5PWNvdmVyYWdlKSkrCiAgZ2VvbV9ib3hwbG90KGx3ZD0wLjEsIGZpbGw9ImdyYXkiLCBzaXplPTAuMSwgb3V0bGllci5zaGFwZSA9IE5BKSsKICBnZW9tX2ppdHRlcihzaXplPTAuMSwgd2lkdGg9MC4yKSsKICBmYWNldF93cmFwKH5zcHAsIHNjYWxlcz0iZnJlZV94IikrCiAgdGhlbWVfYncoKSsKICB0aGVtZShsZWdlbmQucG9zaXRpb24gPSAibm9uZSIsIAogICAgICB0ZXh0PWVsZW1lbnRfdGV4dChzaXplPTEsIGNvbG9yPSJibGFjayIpLCAKICAgICAgbGVnZW5kLmtleS5zaXplID0gdW5pdCgwLjMsICJjbSIpLCAKICAgICAgbGluZSA9IGVsZW1lbnRfbGluZShsaW5ld2lkdGggPSAwLjEpLCAKICAgICAgYXhpcy50aXRsZSA9IGVsZW1lbnRfdGV4dChzaXplPTQpLCAKICAgICAgc3RyaXAudGV4dCA9IGVsZW1lbnRfdGV4dChzaXplPTMpLAogICAgICBheGlzLnRleHQgPSBlbGVtZW50X3RleHQoc2l6ZT00LCBjb2xvcj0iYmxhY2siKSkrCiAgeGxhYigibWdTcyIpKwogIHlsYWIoIlJlYWRzIERlbGl2ZXJlZCBwZXIgU2FtcGxlIikrCiAgdGhlbWUodGV4dD1lbGVtZW50X3RleHQoc2l6ZT02KSwgbGVnZW5kLnBvc2l0aW9uID0gIm5vbmUiKSsKICBzY2FsZV95X2xvZzEwKCkKCnRvcF9yb3cgPSBnZ2FycmFuZ2UocDEsIHAyLCBuY29sID0gMiwgbGFiZWxzID0gYygiQS4iLCAiQi4iKSwgZm9udC5sYWJlbCA9IGxpc3QoZmFjZT0icGxhaW4iLCBzaXplPTgpLCB3aWR0aHMgPSBjKDAuNzUsIDEpKQp0aWZmKHBhc3RlKCJNYW51c2NyaXB0X0ZJR1VSRVMvRmlndXJlX1MxXyIsIHRvZGF5MiwgIi50aWZmIiwgc2VwPSIiKSwgaGVpZ2h0ID0gMjQwMCwgd2lkdGg9NTYwMCwgcmVzPTYwMCkKdG9wX3JvdwpkZXYub2ZmKCkKYGBgCgojIyMjIENvdmVyYWdlICYgbWdDU1RzCmBgYHtyfQptZ2NzdC5jb2xzPC1tZ0NTVC5jb2xbbWF0Y2goc29ydCh1bmlxdWUoY291bnRzLm0kbWdDU1QpKSwgbWdDU1QuY29sJG1nQ1NUKSwgImNvbG9yIl0KCmdncGxvdCh1bmlxdWUoY291bnRzLm1bY291bnRzLm0kY291bnRzID4gMCwgYygibWFyeWxhbmRfSUQiLCAiY292ZXJhZ2UiLCAibWdDU1QiKV0pLCBhZXMoeD1tZ0NTVCwgeT1jb3ZlcmFnZSwgZmlsbD1tZ0NTVCkpK2dlb21fdmlvbGluKGx3ZD0wLjEpK2dlb21faml0dGVyKHNpemU9MC4xLCB3aWR0aD0wLjIpK3RoZW1lX2J3KCkreGxhYigibWdDU1QiKSt5bGFiKCJSZWFkcyBEZWxpdmVyZWQgcGVyIFNhbXBsZSIpK3RoZW1lKHRleHQ9ZWxlbWVudF90ZXh0KHNpemU9OCksIGxlZ2VuZC5wb3NpdGlvbiA9ICJub25lIikrc2NhbGVfeV9sb2cxMCgpK3NjYWxlX2ZpbGxfbWFudWFsKHZhbHVlcz1tZ2NzdC5jb2xzKQojI2dnc2F2ZShwYXN0ZSgiQW5hbHlzaXNfRmlndXJlcy9TYW1wbGVfY292ZXJhZ2VfYnlfbWdDU1RfIiwgdG9kYXkyLCAiLnRpZmYiLCBzZXA9IiIpLCBoZWlnaHQ9NSwgd2lkdGg9OCkKYGBgCgojIE1lcmdlIE1HIGFuZCBNRVRBREFUQQpgYGB7cn0KYTEubWc8LW1lcmdlKGExLCBtZ0NTVHMsIGFsbC54PVRSVUUpCmExLm1nPC1tZXJnZShhMS5tZywgQ1NUcywgYWxsLng9VFJVRSkKIyBPVVRDT01FCmExLm1nJGhpdjwtZmFjdG9yKGExLm1nJGhpdiwgbGV2ZWxzPWMoMSwgMCksIGxhYmVscz1jKCJXTEhJViIsICJISVYtTmVnYXRpdmUiKSkKCmEyLnQxPC1yZWFkX2V4Y2VsKCJGZWJfMTBfRW1haWwvU2FpZGlfQWltMiYzX1RyaWFsMV9BbmFseXRpY1NhbXBsZS54bHN4IikgIyMgQVJUCmRpbShhMi50MSkgIyA0OSBYIDM4CmEyLnQxJGhpdjwtMQphMi50MWE8LWEyLnQxWyxjKDE6Myw1OjYsIDg6MzkpXQpuYW1lcyhhMi50MWEpWzM6NF08LWMoIm1hcnlsYW5kX0lEIiwgIlNwZWNfRGF0ZSIpCmEyLnQxYSRzd2FiPC0xCmEyLnQxYjwtYTIudDFbLGMoMToyLDQsNjo3LDg6MzkpXQpuYW1lcyhhMi50MWIpW2MoMyw1KV08LWMoIm1hcnlsYW5kX0lEIiwgIlNwZWNfRGF0ZSIpCmEyLnQxYiRzd2FiPC0yCmEyLnQxPC1yYmluZChhMi50MWEsIGEyLnQxYikKYTIudDEkQVJUX1BSRVBfZGF0ZTwtYTIudDEkQVJUX2RhdGUKCmEyLnQyPC1yZWFkX2V4Y2VsKCJGZWJfMTBfRW1haWwvU2FpZGlfQWltMiYzX1RyaWFsMl9BbmFseXRpY1NhbXBsZS54bHN4IikgIyMgUFJFUApkaW0oYTIudDIpICMgMTMyIHggMzcKYTIudDIkaGl2PC0wCmEyLnQyYTwtYTIudDJbLGMoMTozLDU6Niw4OjM4KV0KbmFtZXMoYTIudDJhKVszOjRdPC1jKCJtYXJ5bGFuZF9JRCIsICJTcGVjX0RhdGUiKQphMi50MmEkc3dhYjwtMQphMi50MmI8LWEyLnQyWyxjKDE6Miw0LDY6MzgpXQpuYW1lcyhhMi50MmIpW2MoMywgNSldPC1jKCJtYXJ5bGFuZF9JRCIsICJTcGVjX0RhdGUiKQphMi50MmIkc3dhYjwtMgphMi50MjwtcmJpbmQoYTIudDJhLCBhMi50MmIpCmEyLnQyJEFSVF9QUkVQX2RhdGU8LWEyLnQyJFByRVBfRGF0ZQoKYTI8LW1lcmdlKGEyLnQxLCBhMi50MiwgYWxsPVQpCmEyJEFSVF9kYXRlPC1OVUxMCmEyJFByRVBfRGF0ZTwtTlVMTAoKYTIubWc8LW1lcmdlKGEyLCBtZ0NTVHNbLGMoIm1hcnlsYW5kX0lEIiwgIm1nQ1NUMiIpXSwgYWxsLng9VFJVRSkKYTIubWc8LW1lcmdlKGEyLm1nLCBDU1RzWyxjKCJtYXJ5bGFuZF9JRCIsICJDU1QiKV0sIGFsbC54PVRSVUUpCm9yZGVyaW5nPC11bmlxdWUoYTIubWdbb3JkZXIoYTIubWckQ1NUKSwgIm1hcnlsYW5kX0lEIl0pCmEyLm1nJG1hcnlsYW5kX0lEPC1mYWN0b3IoYTIubWckbWFyeWxhbmRfSUQsIGxldmVscz1vcmRlcmluZykKYTIubWckUElEPC1mYWN0b3IoYTIubWckUElEKQphMi5tZyRzd2FiPC1mYWN0b3IoYTIubWckc3dhYikKCnRyZWF0LmNvbHM8LWMoIiNCOERFMjlGRiIsICIjMzM2MzhERkYiKQphMi5tZyR0cmVhdG1lbnQ8LWZhY3RvcihpZmVsc2UoYTIubWckaGl2ID09IDEsICJBUlQiLCAiUHJFUCIpKQphMi5tZyR0cmVhdG1lbnQ8LWZhY3RvcihhMi5tZyR0cmVhdG1lbnQsIGxldmVscz1jKCJBUlQiLCAiUHJFUCIpKQoKIyBPVVRDT01FCmEyLm1nJGhpdjwtZmFjdG9yKGEyLm1nJGhpdiwgbGV2ZWxzPWMoMSwgMCksIGxhYmVscz1jKCJXTEhJViIsICJISVYtTmVnYXRpdmUiKSkKYGBgCgoKIyMgSEVBVE1BUCBQbG90IENTVCArIFRheGEgCmBgYHtyLCB3YXJuaW5nPUZ9CmNvbGZ1bmMgPC0gY29sb3JSYW1wUGFsZXR0ZShjKCJraGFraSIsICJsaW1lZ3JlZW4iLCAiZGFya3NsYXRlZ3JheTEiLCAibWVkaXVtYmx1ZSIsICJtYWdlbnRhIiwgInJlZCIpKQoKI3JlbGFidW5kLm1nQ1NUPC1kY2FzdChyZWxhYnVuZC5tW3JlbGFidW5kJG1hcnlsYW5kX0lEICVpbiUgc2FtcGxlLmxpc3QsIF0sIGZvcm11bGEgPSBtYXJ5bGFuZF9JRH5zcGVjaWVzLCB2YWx1ZS52YXIgPSAicmVsYWJ1bmQiLCBmdW4uYWdncmVnYXRlID0gc3VtKQpyZWxhYnVuZC5tZ0NTVDwtZGNhc3QoY291bnRzLm1bY291bnRzLm0kbWFyeWxhbmRfSUQgJWluJSBzYW1wbGUubGlzdCwgXSwgZm9ybXVsYSA9IG1hcnlsYW5kX0lEfnRheGEsIHZhbHVlLnZhciA9ICJyZWxhYnVuZCIsIGZ1bi5hZ2dyZWdhdGUgPSBzdW0pCnJvd25hbWVzKHJlbGFidW5kLm1nQ1NUKTwtcmVsYWJ1bmQubWdDU1QkbWFyeWxhbmRfSUQKcmVsYWJ1bmQubWdDU1QkbWFyeWxhbmRfSUQ8LU5VTEwKcmVsYWJ1bmQubWdDU1Q8LXJlbGFidW5kLm1nQ1NUWyxvcmRlcihjb2xTdW1zKHJlbGFidW5kLm1nQ1NUKSwgZGVjcmVhc2luZyA9IFRSVUUpXQpyZWxhYnVuZC5tZ0NTVCRDU1Q8LUNTVHNbbWF0Y2gocm93bmFtZXMocmVsYWJ1bmQubWdDU1QpLCBDU1RzJG1hcnlsYW5kX0lEKSwgIkNTVCJdCgp0b3AudGF4YS5uYW1lczwtbmFtZXMocmVsYWJ1bmQubWdDU1QpWzE6NTBdCnRheGEub3JkZXI8LWMoc29ydCh0b3AudGF4YS5uYW1lc1tncmVwKCJMYWN0b2JhY2lsbHVzX2NyaXNwYXR1cyIsIHRvcC50YXhhLm5hbWVzKV0pLApzb3J0KHRvcC50YXhhLm5hbWVzW2dyZXAoIkxhY3RvYmFjaWxsdXNfaW5lcnMiLCB0b3AudGF4YS5uYW1lcyldKSwKc29ydCh0b3AudGF4YS5uYW1lc1tncmVwKCJHYXJkbmVyZWxsYV92YWdpbmFsaXMiLCB0b3AudGF4YS5uYW1lcyldKSwKc29ydCh0b3AudGF4YS5uYW1lc1tncmVwKCJDYS5MYWNobm9jdXJ2YV92YWdpbmFlIiwgdG9wLnRheGEubmFtZXMpXSkpCnRheGEub3JkZXI8LWFwcGVuZCh0YXhhLm9yZGVyLCBjKHRvcC50YXhhLm5hbWVzWyF0b3AudGF4YS5uYW1lcyAlaW4lIHRheGEub3JkZXJdLCAiUHJldm90ZWxsYV9vcmlzIikpCiAgICAgICAgICAgICAgIAp0aGUuZGlzdDwtdmVnZGlzdChhcy5tYXRyaXgocmVsYWJ1bmQubWdDU1RbLDE6MTAwXSksIG5hLnJtID0gVCkKdGhlLmNsc3Rpbmc8LWhjbHVzdCh0aGUuZGlzdCwgbWV0aG9kPSJ3YXJkLkQiKQp0b3A8LXJlbGFidW5kLm1nQ1NUWywxOjEwMF0KCnRvLndyaXRlPC1hcy5kYXRhLmZyYW1lKGNiaW5kKHNhbXBsZUlEPXRoZS5jbHN0aW5nJGxhYmVscywgb3JkZXI9dGhlLmNsc3Rpbmckb3JkZXIsIGRvbVRheGE9Y29sbmFtZXModG9wKVttYXguY29sKHRvcCwgdGllcy5tZXRob2Q9ImZpcnN0IildLCBhYnVuZD1hcHBseSh0b3AsIDEsIG1heCkpKQp0by53cml0ZSRDU1Q8LXJlbGFidW5kLm1nQ1NUW21hdGNoKHRvLndyaXRlJHNhbXBsZUlELCByb3duYW1lcyhyZWxhYnVuZC5tZ0NTVCkpLCAiQ1NUIl0KdG8ud3JpdGUkY3V0cmVlPC1jdXRyZWUodGhlLmNsc3RpbmcsIGs9OCkKCnRvLndyaXRlPC10by53cml0ZVtvcmRlcih0by53cml0ZSRDU1QsIHRvLndyaXRlJGRvbVRheGEsIHRvLndyaXRlJGFidW5kKSwgXQpzYW1wbGUub3JkZXI8LXRvLndyaXRlJHNhbXBsZUlECgphbGwuc2FtcGxlcy5oaXY8LXVuaXF1ZShyYmluZChhMS5tZ1ssYygibWFyeWxhbmRfSUQiLCAiaGl2IildLCBhMi5tZ1ssYygibWFyeWxhbmRfSUQiLCAiaGl2IildKSkKYWxsLnNhbXBsZXMuaGl2JHN3YWI8LWlmZWxzZSh0by53cml0ZSRzYW1wbGVJRCAlaW4lIGEyLm1nJG1hcnlsYW5kX0lELCBhcy5jaGFyYWN0ZXIoYTIubWdbbWF0Y2godG8ud3JpdGUkc2FtcGxlSUQsIGEyLm1nJG1hcnlsYW5kX0lEKSwgInN3YWIiXSksICIxIikKYWxsLnNhbXBsZXMuaGl2JHN3YWI8LWdzdWIoIjEiLCAiTTAiLCBhbGwuc2FtcGxlcy5oaXYkc3dhYikKYWxsLnNhbXBsZXMuaGl2JHN3YWI8LWdzdWIoIjIiLCAiTTEiLCBhbGwuc2FtcGxlcy5oaXYkc3dhYikKYWxsLnNhbXBsZXMuaGl2JHN3YWI8LWZhY3RvcihhbGwuc2FtcGxlcy5oaXYkc3dhYikKICAKICBhMi5tZ1ttYXRjaChhbGwuc2FtcGxlcy5oaXYkbWFyeWxhbmRfSUQsIGEyLm1nJG1hcnlsYW5kX0lEKSwgInN3YWIiXQphbGwuc2FtcGxlcy5oaXYkc3dhYltpcy5uYShhbGwuc2FtcGxlcy5oaXYkc3dhYildPC0iTTAiCgp0aGUuY2x1c3RzPC1hcy5kYXRhLmZyYW1lKGNiaW5kKGBWQUxFTkNJQSBDU1RgPWFzLmNoYXJhY3Rlcih0by53cml0ZSRDU1QpLCAKICAgICAgICAgICAgICAgICAgICAgICAgICAgICAgICBgSElWIFNlcm9zdGF0dXNgPWFzLmNoYXJhY3RlcihhbGwuc2FtcGxlcy5oaXZbbWF0Y2godG8ud3JpdGUkc2FtcGxlSUQsIGFsbC5zYW1wbGVzLmhpdiRtYXJ5bGFuZF9JRCksICJoaXYiXSksIAogICAgICAgICAgICAgICAgICAgICAgICAgICAgICAgIHN3YWI9YXMuY2hhcmFjdGVyKGFsbC5zYW1wbGVzLmhpdlttYXRjaCh0by53cml0ZSRzYW1wbGVJRCwgYWxsLnNhbXBsZXMuaGl2JG1hcnlsYW5kX0lEKSwgInN3YWIiXSkpKQoKcm93Lm5hbWVzKHRoZS5jbHVzdHMpPC10by53cml0ZSRzYW1wbGVJRAp0aGUuY2x1c3RzJGBWQUxFTkNJQSBDU1RgPC1hcy5mYWN0b3IodGhlLmNsdXN0cyRgVkFMRU5DSUEgQ1NUYCkKQ1NUPC1hcy5kYXRhLmZyYW1lKHJiaW5kKGMoIkkiLCAiI0ZFMDMwOCIpLGMoIkktQSIsICIjRkUwMzA4IiksIGMoIkktQiIsICIjRjZEM0RBIiksIGMoIklJIiwgIiM4NkM2MUEiKSxjKCJJSUkiLCAiI0ZGNzIwMCIpLGMoIklJSS1BIiwgIiNGRjcyMDAiKSxjKCJJSUktQiIsICIjRjhBNDBFIiksIGMoIklWIiwgIiMyMjE4ODYiKSxjKCJJVi1BIiwgIiM0NDhBNzMiKSwgYygiSVYtQiIsICIjMjIxODg2IiksIGMoIklWLUMiLCAiI0MwQUNEMyIpLGMoIklWLUMwIiwgIiM5ODk4OTgiKSxjKCJJVi1DMSIsICIjRUY1M0E3IiksYygiSVYtQzIiLCAiI0E3REREQyIpLGMoIklWLUMzIiwgIiM5OEM5OTkiKSxjKCJJVi1DNCIsICIjN0YwQjdDIiksIGMoIlYiLCAiI0ZBRTUwRCIpLCBjKCIiLCAid2hpdGUiKSwgYygiTkEiLCAid2hpdGUiKSkpCmNvbHM8LUNTVFttYXRjaChzb3J0KHVuaXF1ZSh0aGUuY2x1c3RzJGBWQUxFTkNJQSBDU1RgKSksIENTVCRWMSksICJWMiJdCm5hbWVzKGNvbHMpPC11bmlxdWUodGhlLmNsdXN0cyRgVkFMRU5DSUEgQ1NUYCkKCiN0by5wbG90PC1yZWxhYnVuZC5tZ0NTVFtzYW1wbGUub3JkZXIsIDE6NTBdCnRvLnBsb3Q8LXJlbGFidW5kLm1nQ1NUW3NhbXBsZS5vcmRlciwgdGF4YS5vcmRlcl0KbmFtZXModG8ucGxvdCk8LWdzdWIoIl8iLCAiICIsIG5hbWVzKHRvLnBsb3QpKQpuYW1lcyh0by5wbG90KTwtZ3N1YigiXyIsICIgIiwgbmFtZXModG8ucGxvdCkpCml0YWxpY19yb3dfbGFiZWxzIDwtIGxhcHBseShuYW1lcyh0by5wbG90KSwgZnVuY3Rpb24obGFiZWwpIHsKICByZXR1cm4oYnF1b3RlKGl0YWxpYyguKGxhYmVsKSkpKQp9KQoKbmFtZXMoaGl2LmNvbHMpPC1jKCJXTEhJViIsICJISVYtTmVnYXRpdmUiKQpzd2FiLmNvbHM8LWMoImJsYWNrIiwgImdyYXkiKQpuYW1lcyhzd2FiLmNvbHMpPC1jKCJNMCIsICJNMSIpCmNvbGZ1bmMgPC0gY29sb3JSYW1wUGFsZXR0ZShjKCJraGFraSIsICJsaW1lZ3JlZW4iLCAiZGFya3NsYXRlZ3JheTEiLCAibWVkaXVtYmx1ZSIsICJtYWdlbnRhIiwgInJlZCIpKQoKcG5nKHBhc3RlKCJBbmFseXNpc19GaWd1cmVzL2hlYXRtYXBfZGVuZF8iLCB0b2RheTIsICIucG5nIiwgc2VwPSIiKSwgd2lkdGg9NywgaGVpZ2h0PTUuNSwgdW5pdHM9ImluIiwgcmVzPTYwMCkKcGhlYXRtYXAodChhcy5tYXRyaXgodG8ucGxvdCkpLCAKICAgICAgICAgY29sb3IgPSBhbHBoYShjb2xmdW5jKDEwMCksIDEpLCAKICAgICAgICAgY2x1c3Rlcl9yb3dzID0gRkFMU0UsIAogICAgICAgICBjbHVzdGVyX2NvbHMgPSBGQUxTRSwKICAgICAgICAgYW5ub3RhdGlvbl9yb3cgPSBOVUxMLAogICAgICAgICBsZWdlbmQgPSBUUlVFLCAKICAgICAgICAgZm9udHNpemUgPSA3LCAKICAgICAgICAgbGFiZWxzX2NvbCA9ICIiLCAKICAgICAgICAgYW5ub3RhdGlvbl9jb2wgPSB0aGUuY2x1c3RzLCAKICAgICAgICAgYW5ub3RhdGlvbl9jb2xvcnMgPSBsaXN0KGBWQUxFTkNJQSBDU1RgPWNvbHMsIGBISVYgU2Vyb3N0YXR1c2A9aGl2LmNvbHMsIHN3YWI9c3dhYi5jb2xzKSwKICAgICAgICAgbGFiZWxzX3JvdyA9IGFzLmV4cHJlc3Npb24oaXRhbGljX3Jvd19sYWJlbHMpCiAgICAgICAgICkKZGV2Lm9mZigpCgp0aWZmKHBhc3RlKCJBbmFseXNpc19GaWd1cmVzL2hlYXRtYXBfZGVuZF8iLCB0b2RheTIsICIudGlmZiIsIHNlcD0iIiksIHdpZHRoPTcsIGhlaWdodD01LjUpCnBoZWF0bWFwKHQoYXMubWF0cml4KHRvLnBsb3QpKSwgCiAgICAgICAgIGNvbG9yID0gYWxwaGEoY29sZnVuYygxMDApLCAxKSwgCiAgICAgICAgIGNsdXN0ZXJfcm93cyA9IEZBTFNFLCAKICAgICAgICAgY2x1c3Rlcl9jb2xzID0gRkFMU0UsCiAgICAgICAgIGFubm90YXRpb25fcm93ID0gTlVMTCwKICAgICAgICAgbGVnZW5kID0gVFJVRSwgCiAgICAgICAgIGZvbnRzaXplID0gNywgCiAgICAgICAgIGxhYmVsc19jb2wgPSAiIiwgCiAgICAgICAgIGFubm90YXRpb25fY29sID0gdGhlLmNsdXN0cywgCiAgICAgICAgIGFubm90YXRpb25fY29sb3JzID0gbGlzdChgVkFMRU5DSUEgQ1NUYD1jb2xzLCBgSElWIFNlcm9zdGF0dXNgPWhpdi5jb2xzLCBzd2FiPXN3YWIuY29scyksCiAgICAgICAgIGxhYmVsc19yb3cgPSBhcy5leHByZXNzaW9uKGl0YWxpY19yb3dfbGFiZWxzKQogICAgICAgICApCmRldi5vZmYoKQpgYGAKCiMjIEdhcmRuZXJlbGxhIHNwZWNpYXRpb24KIyMjIG1nQ1NUcyAmIEdhcmRuZXJlbGxhCmBgYHtyfQojIyBub3JtIHNwcC4gY291bnRzCnRlbXA8LXJlc2hhcGUyOjptZWx0KGNvdW50cywgaWQudmFycz0ibWFyeWxhbmRfSUQiLCB2YXJpYWJsZS5uYW1lID0gInRheGEiLCB2YWx1ZS5uYW1lID0gImNvdW50cyIpCnRlbXA8LW1lcmdlKHRlbXAsIHJlbGFidW5kLm0sIGFsbD1UUlVFKQp0ZW1wJHRheGE8LWdzdWIoIlxcLl8iLCAiXFwuIiwgdGVtcCR0YXhhKQp0ZW1wJGNvdmVyYWdlPC1yZWFkLnN0YXRzW21hdGNoKHRlbXAkbWFyeWxhbmRfSUQsIHJlYWQuc3RhdHMkc2FtcGxlKSwgIlJlYWRzX2RlbGl2ZXJlZCJdCnRlbXAkc3BwPC1wYXN0ZShzdHJfc3BsaXRfZml4ZWQodGVtcCR0YXhhLCBwYXR0ZXJuID0gIl8iLCBuID0gMylbLDFdLCBzdHJfc3BsaXRfZml4ZWQodGVtcCR0YXhhLCBwYXR0ZXJuID0gIl8iLCBuID0gMylbLDJdLCBzZXA9Il8iKQp0ZW1wPC10ZW1wWyF0ZW1wJG1hcnlsYW5kX0lEICVpbiUgY3RybHMsIF0KCmc8LXJlYWQuZGVsaW0oIn4vRHJvcGJveCAoSUdTKS9tZ3NzX21jc3RfZGV2ZWwvZ2FyZGVuZXJlbGxhX2dlbm9tb3NwLnR4dCIsIGhlYWRlcj1UKQpnLmNvdW50czwtZ2VuZXNbcm93bmFtZXMoZ2VuZXMpICVpbiUgZyRWSVJHT19JRCwgXQpnLmNvdW50czwtZy5jb3VudHNbLCFuYW1lcyhnLmNvdW50cykgJWluJSBjdHJsc10gCmcuY291bnRzJEdnZW5vbW9zcGVjaWVzPC1nW21hdGNoKHJvd25hbWVzKGcuY291bnRzKSwgZyRWSVJHT19JRCksICJTcGVjaWVzIl0gIyMgZ2V0IGdlbm9tb3NwCmcuY291bnRzJFZJUkdPX0lEPC1yb3duYW1lcyhnLmNvdW50cykKZy5jb3VudHMubTwtcmVzaGFwZTI6Om1lbHQoZy5jb3VudHMsIGlkLnZhcnM9YygiVklSR09fSUQiLCAiR2dlbm9tb3NwZWNpZXMiKSwgdmFyaWFibGUubmFtZSA9ICJtYXJ5bGFuZF9JRCIsIHZhbHVlLm5hbWUgPSAibm9ybWNvdW50cyIpICMjIHRvdGFsIGNvdW50cwpnLmNvdW50cy5tJG1nQ1NUPC1tZ0NTVHNbbWF0Y2goZy5jb3VudHMubSRtYXJ5bGFuZF9JRCwgbWdDU1RzJG1hcnlsYW5kX0lEKSwgIm1nQ1NUMiJdICMjIGFkZCBtZ0NTVAoKIyMgZ2V0IHJlbGFidW5kYW5jZSBvZiBHIGluIGEgc2FtcGxlCnRvdGFsLkdjb3VudHM8LWFzLmRhdGEuZnJhbWUodGVtcFt0ZW1wJHNwcCAlaW4lICJHYXJkbmVyZWxsYV92YWdpbmFsaXMiLCBdICU+JSBkcGx5cjo6Z3JvdXBfYnkobWFyeWxhbmRfSUQpICU+JSBkcGx5cjo6c3VtbWFyaXNlKHRvdGFsQ291bnRzPXN1bShjb3VudHMpLCByZWxBYnVuZD1zdW0ocmVsYWJ1bmQpKSkKCmcuY291bnRzLm0uc3VtPC1nLmNvdW50cy5tW2cuY291bnRzLm0kbm9ybWNvdW50cyA+IDAsIF0gJT4lIGRwbHlyOjpncm91cF9ieShtYXJ5bGFuZF9JRCwgR2dlbm9tb3NwZWNpZXMsIG1nQ1NUKSAlPiUgZHBseXI6OnN1bW1hcmlzZShuTlJnZW5lcz1sZW5ndGgodW5pcXVlKFZJUkdPX0lEKSksIGdlbmVfYWJ1bmRhbmNlPXN1bShub3JtY291bnRzKSkKZy5jb3VudHMubS5zdW0kY292ZXJhZ2U8LXNhbXBsZS5zdW1tYXJ5W21hdGNoKGcuY291bnRzLm0uc3VtJG1hcnlsYW5kX0lELCBzYW1wbGUuc3VtbWFyeSRtYXJ5bGFuZF9JRCksICJmaW5hbFJlYWRzX3BlX3NlIl0KZy5jb3VudHMubS5zdW08LW1lcmdlKGcuY291bnRzLm0uc3VtLCB0b3RhbC5HY291bnRzLCBhbGwueD1UUlVFKQpnLmNvdW50cy5tLnN1bSRwcm9wR2dzPC1nLmNvdW50cy5tLnN1bSRnZW5lX2FidW5kYW5jZS9nLmNvdW50cy5tLnN1bSR0b3RhbENvdW50cwpnLmNvdW50cy5tLnN1bSRwcm9wX29mX0dfZ3M8LWcuY291bnRzLm0uc3VtJHJlbEFidW5kKmcuY291bnRzLm0uc3VtJHByb3BHZ3MKbmFtZXMoZy5jb3VudHMubS5zdW0pW2MoMiwgNSwgMTApXTwtYygidGF4YSIsICJjb3VudHMiLCAicmVsYWJ1bmQiKQogIApjb3VudHMubS50ZW1wPC1jb3VudHMubVshY291bnRzLm0kc3BwICVpbiUgIkdhcmRuZXJlbGxhX3ZhZ2luYWxpcyIsIF0KY291bnRzLm0udGVtcDwtcmJpbmQoY291bnRzLm0udGVtcFssYygibWFyeWxhbmRfSUQiLCAidGF4YSIsICJjb3VudHMiLCAicmVsYWJ1bmQiKV0sIGcuY291bnRzLm0uc3VtWyxjKCJtYXJ5bGFuZF9JRCIsICJ0YXhhIiwgImNvdW50cyIsICJyZWxhYnVuZCIpXSkKY291bnRzLm0udGVtcCR0YXhhPC1nc3ViKCJub3QgYXNzaWduZWQiLCAiR2FyZG5lcmVsbGFfdmFnaW5hbGlzIiwgY291bnRzLm0udGVtcCR0YXhhKQpgYGAKCiMjIyBGSUcgMjogSEVBVE1BUCBQbG90IENTVCArIFRheGEgdy9HYXJkICAKYGBge3IsIHdhcm5pbmc9Rn0KY29sZnVuYyA8LSBjb2xvclJhbXBQYWxldHRlKGMoImtoYWtpIiwgImxpbWVncmVlbiIsICJkYXJrc2xhdGVncmF5MSIsICJtZWRpdW1ibHVlIiwgIm1hZ2VudGEiLCAicmVkIikpCgojcmVsYWJ1bmQubWdDU1Q8LWRjYXN0KHJlbGFidW5kLm1bcmVsYWJ1bmQkbWFyeWxhbmRfSUQgJWluJSBzYW1wbGUubGlzdCwgXSwgZm9ybXVsYSA9IG1hcnlsYW5kX0lEfnNwZWNpZXMsIHZhbHVlLnZhciA9ICJyZWxhYnVuZCIsIGZ1bi5hZ2dyZWdhdGUgPSBzdW0pCnJlbGFidW5kLmdhcmQ8LWRjYXN0KGNvdW50cy5tLnRlbXBbY291bnRzLm0udGVtcCRtYXJ5bGFuZF9JRCAlaW4lIHNhbXBsZS5saXN0LCBdLCBmb3JtdWxhID0gbWFyeWxhbmRfSUR+dGF4YSwgdmFsdWUudmFyID0gImNvdW50cyIsIGZ1bi5hZ2dyZWdhdGUgPSBzdW0pCnJvd25hbWVzKHJlbGFidW5kLmdhcmQpPC1yZWxhYnVuZC5nYXJkJG1hcnlsYW5kX0lECnJlbGFidW5kLmdhcmQkbWFyeWxhbmRfSUQ8LU5VTEwKcmVsYWJ1bmQuZ2FyZDwtcmVsYWJ1bmQuZ2FyZC9yb3dTdW1zKHJlbGFidW5kLmdhcmQpCnJlbGFidW5kLmdhcmQ8LXJlbGFidW5kLmdhcmRbLG9yZGVyKGNvbFN1bXMocmVsYWJ1bmQuZ2FyZCksIGRlY3JlYXNpbmcgPSBUUlVFKV0KCnRvcC50YXhhLm5hbWVzPC1uYW1lcyhyZWxhYnVuZC5nYXJkKVsxOjQ1XQp0YXhhLm9yZGVyPC1jKHNvcnQodG9wLnRheGEubmFtZXNbZ3JlcCgiTGFjdG9iYWNpbGx1c19jcmlzcGF0dXMiLCB0b3AudGF4YS5uYW1lcyldKSwKc29ydCh0b3AudGF4YS5uYW1lc1tncmVwKCJMYWN0b2JhY2lsbHVzX2luZXJzIiwgdG9wLnRheGEubmFtZXMpXSksCnNvcnQodG9wLnRheGEubmFtZXNbZ3JlcCgiR2FyZG5lcmVsbGEiLCB0b3AudGF4YS5uYW1lcyldKSwKc29ydCh0b3AudGF4YS5uYW1lc1tncmVwKCJDYS5MYWNobm9jdXJ2YV92YWdpbmFlIiwgdG9wLnRheGEubmFtZXMpXSkpCnRheGEub3JkZXI8LWFwcGVuZCh0YXhhLm9yZGVyLCBjKHRvcC50YXhhLm5hbWVzWyF0b3AudGF4YS5uYW1lcyAlaW4lIHRheGEub3JkZXJdKSkKCmFsbC5zYW1wbGVzLmhpdjwtdW5pcXVlKHJiaW5kKGExLm1nWyxjKCJtYXJ5bGFuZF9JRCIsICJoaXYiKV0sIGEyLm1nWyxjKCJtYXJ5bGFuZF9JRCIsICJoaXYiKV0pKQphbGwuc2FtcGxlcy5oaXYkc3dhYjwtaWZlbHNlKHRvLndyaXRlJHNhbXBsZUlEICVpbiUgYTIubWckbWFyeWxhbmRfSUQsIGFzLmNoYXJhY3RlcihhMi5tZ1ttYXRjaCh0by53cml0ZSRzYW1wbGVJRCwgYTIubWckbWFyeWxhbmRfSUQpLCAic3dhYiJdKSwgIjEiKQphbGwuc2FtcGxlcy5oaXYkc3dhYjwtZ3N1YigiMSIsICJNMCIsIGFsbC5zYW1wbGVzLmhpdiRzd2FiKQphbGwuc2FtcGxlcy5oaXYkc3dhYjwtZ3N1YigiMiIsICJNMSIsIGFsbC5zYW1wbGVzLmhpdiRzd2FiKQphbGwuc2FtcGxlcy5oaXYkc3dhYjwtZmFjdG9yKGFsbC5zYW1wbGVzLmhpdiRzd2FiKQogIAogIGEyLm1nW21hdGNoKGFsbC5zYW1wbGVzLmhpdiRtYXJ5bGFuZF9JRCwgYTIubWckbWFyeWxhbmRfSUQpLCAic3dhYiJdCmFsbC5zYW1wbGVzLmhpdiRzd2FiW2lzLm5hKGFsbC5zYW1wbGVzLmhpdiRzd2FiKV08LSJNMCIKCnRoZS5jbHVzdHM8LWFzLmRhdGEuZnJhbWUoY2JpbmQoIHN3YWI9YXMuY2hhcmFjdGVyKGFsbC5zYW1wbGVzLmhpdlttYXRjaCh0by53cml0ZSRzYW1wbGVJRCwgYWxsLnNhbXBsZXMuaGl2JG1hcnlsYW5kX0lEKSwgInN3YWIiXSksCiAgICAgICAgICAgICAgICAgICAgICAgICAgICAgICAgYEhJViBTZXJvc3RhdHVzYD1hcy5jaGFyYWN0ZXIoYWxsLnNhbXBsZXMuaGl2W21hdGNoKHRvLndyaXRlJHNhbXBsZUlELCBhbGwuc2FtcGxlcy5oaXYkbWFyeWxhbmRfSUQpLCAiaGl2Il0pLCAKICAgICAgICAgICAgICAgICAgICAgICAgICAgICAgICAgYFZBTEVOQ0lBIENTVGA9YXMuY2hhcmFjdGVyKHRvLndyaXRlJENTVCkpKQoKcm93Lm5hbWVzKHRoZS5jbHVzdHMpPC10by53cml0ZSRzYW1wbGVJRAp0aGUuY2x1c3RzJGBWQUxFTkNJQSBDU1RgPC1hcy5mYWN0b3IodGhlLmNsdXN0cyRgVkFMRU5DSUEgQ1NUYCkKdGhlLmNsdXN0cyRgSElWIFNlcm9zdGF0dXNgPC1mYWN0b3IodGhlLmNsdXN0cyRgSElWIFNlcm9zdGF0dXNgLCBsZXZlbHM9YygiV0xISVYiLCAiSElWLU5lZ2F0aXZlIikpCiNzYW1wbGUub3JkZXI8LXJvd25hbWVzKHRoZS5jbHVzdHMpW29yZGVyKHRoZS5jbHVzdHMkc3dhYiwgdGhlLmNsdXN0cyRgSElWIFNlcm9zdGF0dXNgLCB0aGUuY2x1c3RzJGBWQUxFTkNJQSBDU1RgKV0Kc2FtcGxlLm9yZGVyPC1yb3duYW1lcyh0aGUuY2x1c3RzKVtvcmRlcih0aGUuY2x1c3RzJGBWQUxFTkNJQSBDU1RgLCB0aGUuY2x1c3RzJGBISVYgU2Vyb3N0YXR1c2ApXQoKCkNTVDwtYXMuZGF0YS5mcmFtZShyYmluZChjKCJJIiwgIiNGRTAzMDgiKSxjKCJJLUEiLCAiI0ZFMDMwOCIpLCBjKCJJLUIiLCAiI0Y2RDNEQSIpLCBjKCJJSSIsICIjODZDNjFBIiksYygiSUlJIiwgIiNGRjcyMDAiKSxjKCJJSUktQSIsICIjRkY3MjAwIiksYygiSUlJLUIiLCAiI0Y4QTQwRSIpLCBjKCJJViIsICIjMjIxODg2IiksYygiSVYtQSIsICIjNDQ4QTczIiksIGMoIklWLUIiLCAiIzIyMTg4NiIpLCBjKCJJVi1DIiwgIiNDMEFDRDMiKSxjKCJJVi1DMCIsICIjOTg5ODk4IiksYygiSVYtQzEiLCAiI0VGNTNBNyIpLGMoIklWLUMyIiwgIiNBN0REREMiKSxjKCJJVi1DMyIsICIjOThDOTk5IiksYygiSVYtQzQiLCAiIzdGMEI3QyIpLCBjKCJWIiwgIiNGQUU1MEQiKSwgYygiIiwgIndoaXRlIiksIGMoIk5BIiwgIndoaXRlIikpKQpjb2xzPC1DU1RbbWF0Y2goc29ydCh1bmlxdWUodGhlLmNsdXN0cyRgVkFMRU5DSUEgQ1NUYCkpLCBDU1QkVjEpLCAiVjIiXQpuYW1lcyhjb2xzKTwtdW5pcXVlKHRoZS5jbHVzdHMkYFZBTEVOQ0lBIENTVGApCgojdG8ucGxvdDwtcmVsYWJ1bmQubWdDU1Rbc2FtcGxlLm9yZGVyLCAxOjUwXQp0by5wbG90PC1yZWxhYnVuZC5nYXJkW3NhbXBsZS5vcmRlciwgdGF4YS5vcmRlclsxOjQ1XV0KdGhlLmRpc3Q8LXZlZ2Rpc3QoYXMubWF0cml4KHRvLnBsb3QpLCBuYS5ybSA9IFQpCnRoZS5jbHN0aW5nPC1oY2x1c3QodGhlLmRpc3QsIG1ldGhvZD0id2FyZC5EIikKCm5hbWVzKHRvLnBsb3QpPC1nc3ViKCJfIiwgIiAiLCBuYW1lcyh0by5wbG90KSkKbmFtZXModG8ucGxvdCk8LWdzdWIoIl8iLCAiICIsIG5hbWVzKHRvLnBsb3QpKQppdGFsaWNfcm93X2xhYmVscyA8LSBsYXBwbHkobmFtZXModG8ucGxvdCksIGZ1bmN0aW9uKGxhYmVsKSB7CiAgcmV0dXJuKGJxdW90ZShpdGFsaWMoLihsYWJlbCkpKSkKfSkKCm5hbWVzKGhpdi5jb2xzKTwtYygiV0xISVYiLCAiSElWLU5lZ2F0aXZlIikKc3dhYi5jb2xzPC1jKCJibGFjayIsICJncmF5IikKbmFtZXMoc3dhYi5jb2xzKTwtYygiTTAiLCAiTTEiKQpjb2xmdW5jIDwtIGNvbG9yUmFtcFBhbGV0dGUoYygia2hha2kiLCAibGltZWdyZWVuIiwgImRhcmtzbGF0ZWdyYXkxIiwgIm1lZGl1bWJsdWUiLCAibWFnZW50YSIsICJyZWQiKSkKI3RpZmYocGFzdGUoIk1hbnVzY3JpcHRfRklHVVJFUy9GaWd1cmVfMl8iLCB0b2RheTIsICIudGlmZiIsIHNlcD0iIiksIHdpZHRoPTQsIGhlaWdodD0zKQojd2lkdGhfaW5fcGl4ZWxzIDwtIHdpZHRoX2luX2luY2hlcyAqIGhpZ2hfcmVzCiNoZWlnaHRfaW5fcGl4ZWxzIDwtIGhlaWdodF9pbl9pbmNoZXMgKiBoaWdoX3JlcwoKdGlmZihmaWxlbmFtZSA9IHBhc3RlKCJNYW51c2NyaXB0X0ZJR1VSRVMvRmlndXJlXzJfIiwgdG9kYXkyLCAiLnRpZmYiLCBzZXA9IiIpLCB3aWR0aCA9IDI0MDAsIGhlaWdodCA9IDE4MDAsIHJlcyA9IDYwMCkKcGhlYXRtYXAodChhcy5tYXRyaXgodG8ucGxvdCkpLCAKICAgICAgICAgY29sb3IgPSBhbHBoYShjb2xmdW5jKDEwMCksIDEpLCAKICAgICAgICAgY2x1c3Rlcl9yb3dzID0gRkFMU0UsIAogICAgICAgICBjbHVzdGVyX2NvbHMgPSBGQUxTRSwKICAgICAgICAgYW5ub3RhdGlvbl9yb3cgPSBOVUxMLAogICAgICAgICBhbm5vdGF0aW9uX2xlZ2VuZCA9IFRSVUUsCiAgICAgICAgIGxlZ2VuZCA9IFRSVUUsIAogICAgICAgICBmb250c2l6ZSA9IDMsIAogICAgICAgICBzaG93X2NvbG5hbWVzID0gRiwKICAgICAgICAgYW5ub3RhdGlvbl9jb2wgPSB0aGUuY2x1c3RzLCAKICAgICAgICAgYW5ub3RhdGlvbl9jb2xvcnMgPSBsaXN0KGBWQUxFTkNJQSBDU1RgPWNvbHMsIGBISVYgU2Vyb3N0YXR1c2A9aGl2LmNvbHMsIHN3YWI9c3dhYi5jb2xzKSwKICAgICAgICAgbGFiZWxzX3JvdyA9IGFzLmV4cHJlc3Npb24oaXRhbGljX3Jvd19sYWJlbHMpLAogICAgICAgICB3aWR0aCA9IDMsCiAgICAgICAgIGhlaWdodCA9IDQKICAgICAgICAgKQpkZXYub2ZmKCkKYGBgCiAKCiMgQWltIDEsIEhJViAoKykgdG8gKC0pIHgucy4KIyMgQ292YXJpYXRlcyAKYGBge3J9CiMgRklYRUQgRUZGRUNUUyAtIHRvIHJlZHVjZSB0aGUgY2hhbmNlcyB0aGF0IGFuIEhJVi9taWNyb2Jpb21lIGFzc29jaWF0aW9uIGlzIGNvbmZvdW5kZWQgYnkgc29tZXRoaW5nIGVsc2UuCmxhYmVsKGExLm1nJHNkX2FnZSk8LSJBZ2UgYXQgZW5yb2xsbWVudCAoeWVhcnMpIgpjb3ZhcjwtYygic2RfYWdlIikKCiMjIHBoX2VnYTogZXN0aW1hdGVkIGdlc3RhdGlvbmFsIGFnZSBhdCBlbnJvbGxtZW50OiBDb250aW51b3VzIApsYWJlbChhMS5tZyRwaF9lZ2EpPC0iR2VzdGF0aW9uYWwgYWdlIGF0IGVucm9sbG1lbnQgKHdlZWtzKSIKY292YXI8LWFwcGVuZChjb3ZhciwgInBoX2VnYSIpCgojIEdyYXZpZGl0eTogIjA9Tm8gcHJpb3IgcHJlZ25hbmNpZXMsIDE9QXQgbGVhc3Qgb25lIHByaW9yIHByZWduYW5jeSIKYTEubWckZ3JhdmlkaXR5PC1mYWN0b3IoYTEubWckZ3JhdmlkaXR5LCBsZXZlbHM9YygwLCAxKSwgbGFiZWxzPWMoIk5vIHByaW9yIHByZWduYW5jaWVzIiwgIkF0IGxlYXN0IG9uZSBwcmlvciBwcmVnbmFuY3kiKSkKbGFiZWwoYTEubWckZ3JhdmlkaXR5KTwtIkdyYXZpZGl0eSIKY292YXI8LWFwcGVuZChjb3ZhciwgImdyYXZpZGl0eSIpCgojIFByaW1hcnkgcGFydG5lciBISVYgc3RhdHVzOiAwPUhJVi1uZWdhdGl2ZSwgMT1ISVYtcG9zaXRpdmUsIDI9SW5kZXRlcm1pbmF0ZSwgMz1QYXJ0bmVyIG5ldmVyIHRlc3RlZCBmb3IgSElWLCA5OTg9SSBkb24ndCBrbm93LCA5OTk9Tm8gcmVzcG9uc2UKYTEubWckcDFfc3RhdHVzW2ExLm1nJHAxX3N0YXR1cyAlaW4lIGMoMywgOTk4KV08LTMKYTEubWckcDFfc3RhdHVzPC1mYWN0b3IoYTEubWckcDFfc3RhdHVzLCBsZXZlbHM9YygwLCAxLCAzKSwgbGFiZWxzPWMoIkhJVi1uZWdhdGl2ZSIsICJISVYtcG9zaXRpdmUiLCAiRG9uJ3Qga25vdyIpKQpsYWJlbChhMS5tZyRwMV9zdGF0dXMpPC0iUHJpbWFyeSBwYXJ0bmVyIEhJViBzZXJvc3RhdHVzICoiCmNvdmFyPC1hcHBlbmQoY292YXIsICJwMV9zdGF0dXMiKQoKIyAjIE51bWJlciBvZiBzZXh1YWwgaW50ZXJjb3Vyc2UgYWN0cyBpbiB0aGUgcGFzdCAzMCBkYXlzOiAiMD1ObyBwYXJ0bmVycywgMT1PbmUgcGFydG5lciwgMj1NdWx0aXBsZSBwYXJ0bmVycyIKIyBhMS5tZyRzZXhfZnJlcV9jYXQ8LWlmZWxzZShhMS5tZyRzZXhfZnJlcSAlaW4lIDAsIDAsIGlmZWxzZShhMS5tZyRzZXhfZnJlcSAlaW4lIDEtNSwgMSwgaWZlbHNlKGlzLm5hKGExLm1nJHNleF9mcmVxKSwgMywgMikpKQojIGExLm1nJHNleF9mcmVxX2NhdDwtZmFjdG9yKGExLm1nJHNleF9mcmVxLCBsZXZlbHM9YygwLCAxLCAyLCAzKSwgbGFiZWxzPWMoIjAiLCAiMS01IiwgIj41IiwgIkFic3RhaW5lZCIpKQojIGxhYmVsKGExLm1nJHNleF9mcmVxX2NhdCk8LSJOdW1iZXIgb2Ygc2V4dWFsIGludGVyY291cnNlIGFjdHMgaW4gdGhlIHBhc3QgMzAgZGF5cyIKIyBjb3ZhcjwtYXBwZW5kKGNvdmFyLCAic2V4X2ZyZXFfY2F0IikKCiMgTnVtYmVyIG9mIHBhcnRuZXJzIGluIHBhc3QgdGhyZWUgbW9udGhzOiAiMD1ObyBwYXJ0bmVycywgMT1PbmUgcGFydG5lciwgMj1NdWx0aXBsZSBwYXJ0bmVycyIKYTEubWckY3Vycl9wdG5yczwtZmFjdG9yKGExLm1nJGN1cnJfcHRucnMsIGxldmVscz1jKDAsIDEsIDIpLCBsYWJlbHM9YygiMCIsICIxIiwgIk11bHRpcGxlIikpIApsYWJlbChhMS5tZyRjdXJyX3B0bnJzKTwtIk51bWJlciBvZiBwYXJ0bmVycyBpbiBwYXN0IHRocmVlIG1vbnRocyIKY292YXI8LWFwcGVuZChjb3ZhciwgImN1cnJfcHRucnMiKQoKI0ZJWCBhMS5tZyRjb25kb21fZnJlcV9jYXQgLi4uIHRyeSBncm91cGluZyBzb21ldGltZXMgd2l0aCBuZXZlciAtIGhvdyBkb2VzIHRoaXMgY2hhbmdlPwojQ29uc2lzdGVudCBjb25kb20gdXNlIHdpdGggcHJpbWFyeSBwYXJ0bmVyIGluIHBhc3QgMzAgZGF5czogIjA9TmV2ZXIsIDE9U29tZXRpbWVzLCAyPUNvbnNpc3RlbnQiCmExLm1nJGNvbmRvbV9mcmVxX2NhdF9mPC1hMS5tZyRjb25kb21fZnJlcV9jYXQKYTEubWckY29uZG9tX2ZyZXFfY2F0X2ZbYTEubWckY29uZG9tX2ZyZXFfY2F0X2YgJWluJSBjKDAsIDEpXTwtMAphMS5tZyRjb25kb21fZnJlcV9jYXRfZlthMS5tZyRjb25kb21fZnJlcV9jYXRfZiAlaW4lIDJdPC0yCmExLm1nJGNvbmRvbV9mcmVxX2NhdF9mW2lzLm5hKGExLm1nJGNvbmRvbV9mcmVxX2NhdCldPC0zCmExLm1nJGNvbmRvbV9mcmVxX2NhdF9mPC1mYWN0b3IoYTEubWckY29uZG9tX2ZyZXFfY2F0X2YsIGxldmVscz1jKDAsIDIsIDMpLCBsYWJlbHM9YygiTmV2ZXIgb3IgU29tZXRpbWVzIiwgIkNvbnNpc3RlbnQiLCAiQWJzdGFpbmVkIikpCmExLm1nJGNvbmRvbV9mcmVxX2NhdF9mPC1yZWxldmVsKGExLm1nJGNvbmRvbV9mcmVxX2NhdF9mLCByZWY9IkFic3RhaW5lZCIpCmxhYmVsKGExLm1nJGNvbmRvbV9mcmVxX2NhdF9mKTwtIkNvbnNpc3RlbnQgY29uZG9tIHVzZSB3aXRoIHByaW1hcnkgcGFydG5lciBpbiBwYXN0IDMwIGRheXMgXiIKIyBjb3ZhcjwtYXBwZW5kKGNvdmFyLCAiY29uZG9tX2ZyZXFfY2F0X2YiKQoKIyMgcGhfc3lwaGlsaXNfdG9sZDogc2VsZi1yZXBvcnQgc3lwaGlsaXMgcGFzdCAzIG1vbnRocy4gQ0FUCiMgUmVzdWx0cyBvZiBzeXBoaWxpcyB0ZXN0IGR1cmluZyBpbmRleCBwcmVnbmFuY3kgKGFudGVuYXRhbCByZWNvcmQpOiAiMD1Ob25yZWFjdGl2ZSwgMT1SZWFjdGl2ZSwgOTk5PU5vIHRlc3QgcmVzdWx0cyByZWNvcmRlZCIKYTEubWckYmx0ZXN0X3N5cHJlc3VsdHM8LWZhY3RvcihhMS5tZyRibHRlc3Rfc3lwcmVzdWx0cywgbGV2ZWxzPWMoMCwgMSksIGxhYmVscyA9IGMoIk5vIiwgIlllcyIpKQpsYWJlbChhMS5tZyRibHRlc3Rfc3lwcmVzdWx0cyk8LSJEaWFnbm9zZWQgd2l0aCBzeXBoaWxpcyBpbiBwYXN0IDMgbW9udGhzIgpjb3ZhcjwtYXBwZW5kKGNvdmFyLCAiYmx0ZXN0X3N5cHJlc3VsdHMiKQoKIyBBYm5vcm1hbCB2YWdpbmFsIGRpc2NoYXJnZSBvYnNlcnZlZCBpbiBwYXN0IDMgbW9udGhzIChzZWxmLXJlcG9ydCk6ICIwPU5vLCAxPVllcywgOTk4PUkgZG9uJ3Qga25vdywgOTk5PU5vIFJlc3BvbnNlIgphMS5tZyRwaF9kaXNjaGFyZ2U8LWZhY3RvcihhMS5tZyRwaF9kaXNjaGFyZ2UsIGxldmVscz1jKDAsIDEpLCBsYWJlbHM9YygiTm8iLCAiWWVzIikpCmxhYmVsKGExLm1nJHBoX2Rpc2NoYXJnZSk8LSJBYm5vcm1hbCB2YWdpbmFsIGRpc2NoYXJnZSBvYnNlcnZlZCBpbiBwYXN0IDMgbW9udGhzIChzZWxmLXJlcG9ydCkiCmNvdmFyPC1hcHBlbmQoY292YXIsICJwaF9kaXNjaGFyZ2UiKQoKIyBHZW5pdGFsIHVsY2VycyBvYnNlcnZlZCBpbiBwYXN0IDMgbW9udGhzIChzZWxmLXJlcG9ydCk6ICIwPU5vLCAxPVllcywgOTk4PUkgZG9uJ3Qga25vdywgOTk5PU5vIFJlc3BvbnNlIgphMS5tZyRwaF9zb3Jlc191bGNlcnM8LWZhY3RvcihhMS5tZyRwaF9zb3Jlc191bGNlcnMsIGxldmVscz1jKDAsIDEpLCBsYWJlbHM9YygiTm8iLCAiWWVzIikpCmxhYmVsKGExLm1nJHBoX3NvcmVzX3VsY2Vycyk8LSJHZW5pdGFsIHVsY2VycyBvYnNlcnZlZCBpbiBwYXN0IDMgbW9udGhzIChzZWxmLXJlcG9ydCkiCmNvdmFyPC1hcHBlbmQoY292YXIsICJwaF9zb3Jlc191bGNlcnMiKQoKIyBSdW5uaW5nIHdhdGVyIGluIGhvbWU6ICIwPU5vLCAxPVllcyIKYTEubWckd2F0ZXI8LWZhY3RvcihhMS5tZyR3YXRlciwgbGV2ZWxzPWMoMCwgMSksIGxhYmVscz1jKCJObyIsICJZZXMiKSkKbGFiZWwoYTEubWckd2F0ZXIpPC0iUnVubmluZyB3YXRlciBpbiBob21lIgpjb3ZhcjwtYXBwZW5kKGNvdmFyLCAid2F0ZXIiKQoKIyBQcmVnbmFuY3kgT3V0Y29tZQphMS5tZyRwcmVnX291dGNvbWU8LWEyJHByZWdfb3V0Y29tZVttYXRjaChhMS5tZyRtYXJ5bGFuZF9JRCwgYTIkbWFyeWxhbmRfSUQpXQptZXRhPC1yZWFkLmNzdigiRmViXzEwX2VtYWlsL1RQMl9wcmVnb3V0Y29tZV9uZWVkZWQuY3N2IikKYTEubWckcHJlZ19vdXRjb21lPC1pZmVsc2UoaXMubmEoYTEubWckcHJlZ19vdXRjb21lKSwgbWV0YSRwcmVnX291dGNvbWVbbWF0Y2goYTEubWckbWFyeWxhbmRfSUQsIG1ldGEkbWFyeWxhbmRfSUQpXSwgYXMuY2hhcmFjdGVyKGExLm1nJHByZWdfb3V0Y29tZSkpCmExLm1nJHByZWdfb3V0Y29tZV9jbGVhbjwtZmFjdG9yKGlmZWxzZShncmVwbCgiUHJldGVybSBEZWxpdmVyeSIsIGExLm1nJHByZWdfb3V0Y29tZSksICJQcmV0ZXJtIERlbGl2ZXJ5IiwgaWZlbHNlKGdyZXBsKCJUZXJtIERlbGl2ZXJ5IiwgYTEubWckcHJlZ19vdXRjb21lKSwgIlRlcm0gRGVsaXZlcnkiLCBOQSkpLCBsZXZlbHM9YygiVGVybSBEZWxpdmVyeSIsICJQcmV0ZXJtIERlbGl2ZXJ5IikpCmExLm1nJHByZWdfb3V0Y29tZV9jbGVhbjwtcmVsZXZlbChhMS5tZyRwcmVnX291dGNvbWVfY2xlYW4sIHJlZj0iVGVybSBEZWxpdmVyeSIpCmxhYmVsKGExLm1nJHByZWdfb3V0Y29tZV9jbGVhbik8LSJQcmVnbmFuY3kgT3V0Y29tZSIKCmNvdmFyczwtcGFzdGUoY292YXIsIGNvbGxhcHNlPSIgKyAiKQp0YWJsZTEofiBzZF9hZ2UgKyBwaF9lZ2EgKyBncmF2aWRpdHkgKyBwMV9zdGF0dXMgKyBjdXJyX3B0bnJzICsgY29uZG9tX2ZyZXFfY2F0X2YgKyBibHRlc3Rfc3lwcmVzdWx0cyArIHBoX2Rpc2NoYXJnZSArIHBoX3NvcmVzX3VsY2VycyArIHdhdGVyICsgcHJlZ19vdXRjb21lX2NsZWFuICB8IGhpdiwgZGF0YT1hMS5tZywgb3ZlcmFsbD1GLCBleHRyYS5jb2w9bGlzdChgUC12YWx1ZWA9cHZhbHVlKSkKYGBgCgojIyMgU3BlY2llcyByaWNobmVzcyAmIGRpdmVyc2l0eSAoQ2hhbyByaWNobmVzcyAmIFNoYW5ub24pCjI0RmViIC0gdGhlIHJlc3VsdHMgbG9vayBhcy1leHBlY3RlZCBmb3IgcHJpb3IgSElWIHN0dWRpZXMgYWNjb3JkaW5nIHRvIEpvbmkuIAotLSBVcGRhdGUgYW5hbHlzZXMgdG8gYWNjb3VudCBmb3IgY292YXJpYXRlIGRhdGEuCiMjIyMjLSBEaXZlcnNpdHkgYnkgcmF3IGFuZCByYXJlZmllZCBkYXRhCmBgYHtyfQpjb3VudHM8LWFzLmRhdGEuZnJhbWUocmVzaGFwZTI6OmRjYXN0KGNvdW50cy5tLCBtYXJ5bGFuZF9JRH50YXhhMiwgdmFsdWUudmFyPSJjb3VudHMiLCBmdW4uYWdncmVnYXRlID0gc3VtKSkKcm93bmFtZXMoY291bnRzKTwtY291bnRzJG1hcnlsYW5kX0lECmNvdW50cyRtYXJ5bGFuZF9JRDwtTlVMTApleHA8LXBoeWxvc2VxKG90dV90YWJsZShyb3VuZChjb3VudHNbcm93bmFtZXMoY291bnRzKSAlaW4lIGExLm1nJG1hcnlsYW5kX0lELCBdKSwgdGF4YV9hcmVfcm93cyA9IEYpKQpjb3VudHMkbWFyeWxhbmRfSUQ8LXJvd25hbWVzKGNvdW50cykKcm93bmFtZXMoYTEubWcpPC1hMS5tZyRtYXJ5bGFuZF9JRApleHA8LW1lcmdlX3BoeWxvc2VxKGV4cCwgc2FtcGxlX2RhdGEoYTEubWcpKQpyaWNoPC1lc3RpbWF0ZV9yaWNobmVzcyggZXhwLCBtZWFzdXJlcyA9IGMoIkNoYW8xIiwgIlNoYW5ub24iKSkKcmljaCRtYXJ5bGFuZF9JRDwtcm93bmFtZXMocmljaCkKYTEubWc8LW1lcmdlKGExLm1nLCByaWNoLCBhbGwueD1UUlVFKQpgYGAgCgoKCiMjIyMjLSBBbHBoYSBEaXZlcnNpdHkgJiBISVYKYGBge3J9CmExLm1nICU+JQogIHN1bW1hcmlzZSgKICAgIGNvdW50ID0gbigpLAogICAgbWVkaWFuID0gbWVkaWFuKFNoYW5ub24sIG5hLnJtID0gVFJVRSksCiAgICBJUVIgPSBJUVIoU2hhbm5vbiwgbmEucm0gPSBUUlVFKQogICkKCmdyb3VwX2J5KGExLm1nLCBoaXYpICU+JQogIHN1bW1hcmlzZSgKICAgIGNvdW50ID0gbigpLAogICAgbWVkaWFuID0gbWVkaWFuKFNoYW5ub24sIG5hLnJtID0gVFJVRSksCiAgICBJUVIgPSBJUVIoU2hhbm5vbiwgbmEucm0gPSBUUlVFKQogICkKCndpbGNveC50ZXN0KFNoYW5ub24gfiBoaXYsIGRhdGEgPSBhMS5tZywKICAgICAgICAgICAgICAgICAgIGV4YWN0ID0gRkFMU0UpCgp3aWxjb3hfdGVzdDwtd2lsY294LnRlc3QoU2hhbm5vbiB+IGhpdiwgZGF0YSA9IGExLm1nLAogICAgICAgICAgICAgICAgICAgZXhhY3QgPSBGQUxTRSkKcF92YWx1ZSA8LSB3aWxjb3hfdGVzdCRwLnZhbHVlCnNpZ25pZmljYW5jZSA8LSBpZmVsc2UocF92YWx1ZSA8IDAuMDAxLCAiKioqIiwgaWZlbHNlKHBfdmFsdWUgPCAwLjAxLCAiKioiLCBpZmVsc2UocF92YWx1ZSA8IDAuMDUsICIqIiwgIm5zIikpKQojQXQgZW5yb2xsbWVudCAocHJpb3IgdG8gQVJWIGluaXRhdGlvbiksIG1lZGlhbiBTaGFubm9uIGRpdmVyc2l0eSBvZiBhbGwgdmFnaW5hbCBtaWNyb2Jpb21lcyB3YXMgMC44LCB3aXRoIGEgc2lnbmlmaWNhbnRseSBoaWdoZXIgdmFsdWUgb2JzZXJ2ZWQgYW1vbmcgV0xISVYgY29tcGFyZWQgdG8gSElWLW5lZ2F0aXZlIHdvbWVuICgxLjQ1IHZzLiAwLjcsIHA8MC4wMDEsIEZpZ3VyZSAzQSkuIApgYGAKCiMjIyMjI0ZJRyAzQTogU2hhbm5vbiAmIEhJVgpgYGB7cn0KY21wPC1saXN0KGMoIldMSElWIiwgIkhJVi1OZWdhdGl2ZSIpKQpwMTwtZ2dwbG90KGExLm1nLCBhZXMoeD1oaXYsIHk9U2hhbm5vbiwgZmlsbD1oaXYpKSsKICBnZW9tX2JveHBsb3QoY29sb3I9ImJsYWNrIiwgbHdkPTAuMSwgb3V0bGllci5zaGFwZSA9IE5BLCBub3RjaCA9IFQpKwogIHNjYWxlX2ZpbGxfbWFudWFsKHZhbHVlcz1oaXYuY29scywgbGFiZWw9YygiSElWKC0pIiwgIldMSElWIiksIG5hbWU9IiIpKwogIGdlb21fcG9pbnQocG9zaXRpb249cG9zaXRpb25faml0dGVyZG9kZ2UoKSwgc2l6ZT0wLjAxKSsKICB0aGVtZV9idygpKwogIHRoZW1lKGxlZ2VuZC5wb3NpdGlvbiA9ICJub25lIiwKICAgICAgICB0ZXh0PWVsZW1lbnRfdGV4dChzaXplPTYsIGNvbG9yPSJibGFjayIpLAogICAgICAgIGxpbmUgPSBlbGVtZW50X2xpbmUobGluZXdpZHRoID0gMC4xKSwgCiAgICAgICAgYXhpcy50aXRsZSA9IGVsZW1lbnRfdGV4dChzaXplPTUpLCAKICAgICAgICBheGlzLnRleHQgPSBlbGVtZW50X3RleHQoY29sb3I9ImJsYWNrIikpKwogIHlsYWIoIlNoYW5ub24gRGl2ZXJzaXR5IikrCiAgeGxhYigiIikrCiAgZ2VvbV9zaWduaWYoY29tcGFyaXNvbnMgPSBjbXAsIAogICAgICAgICAgICAgIGFubm90YXRpb25zID0gc2lnbmlmaWNhbmNlLCAKICAgICAgICAgICAgICB5X3Bvc2l0aW9uID0gMi44LCAKICAgICAgICAgICAgICB0aXBfbGVuZ3RoID0gMCwgCiAgICAgICAgICAgICAgbWFwX3NpZ25pZl9sZXZlbCA9IGMoIioqKiI9MC4wMDEsICIqKiI9MC4wMSwgIioiPTAuMDUpLCAKICAgICAgICAgICAgICB2anVzdCA9IDAuOCwgCiAgICAgICAgICAgICAgc2l6ZSA9IDAuMywgCiAgICAgICAgICAgICAgdGV4dHNpemUgPSAzKQojZ2dzYXZlKHBhc3RlKCJNYW51c2NyaXB0X0ZJR1VSRVMvRmlndXJlXzNhIiwgdG9kYXkyLCAiLnRpZmYiLCBzZXA9IiIpLCBoZWlnaHQgPSAzLCB3aWR0aD0yLCBkcGkgPSA2MDApCmBgYAoKIyMjIyMtIEFscGhhIERpdmVyc2l0eSAmIEhJVgpgYGB7cn0KIyMjIENoaVNxCmNoaXNxX3Rlc3Q8LWNoaXNxLnRlc3QoeCA9IGExLm1nJENTVCwgeT1hMS5tZyRoaXYpCnBfdmFsdWUgPC0gY2hpc3FfdGVzdCRwLnZhbHVlCnNpZ25pZmljYW5jZSA8LSBpZmVsc2UocF92YWx1ZSA8IDAuMDAxLCAiKioqIiwgaWZlbHNlKHBfdmFsdWUgPCAwLjAxLCAiKioiLCBpZmVsc2UocF92YWx1ZSA8IDAuMDUsICIqIiwgIm5zIikpKQoKIyMgWC1zcXVhcmVkID0gMjUuMjE5LCBkZiA9IDMsIHAtdmFsdWUgPSAxLjM5ZS0wNQojQmFzZWxpbmUgSElWIHNlcm9zdGF0dXMgd2FzIGFzc29jaWF0ZWQgd2l0aCBDU1QgKHA8MC4wMDEsIEZpZ3VyZSAzQikuCmBgYAoKIyMjIyMjRklHIDNCOiBTaGFubm9uICYgSElWIFRlc3QgcHJvcG9ydGlvbiBvZiBDU1QgYnkgSElWCmBgYHtyLCB3YXJuaW5nPUZ9CmNoaS50ZXN0IDwtIGZ1bmN0aW9uKGEsIGIpIHsKICByZXR1cm4oY2hpc3EudGVzdChjYmluZChhLCBiKSkpCn0KIyMgbWFrZSBkZjogQ1NUIGhpdiBuCnRvLnBsb3Q8LWFzLmRhdGEuZnJhbWUoYTEubWdbLGMoImhpdiIsICJDU1QiLCAibWFyeWxhbmRfSUQiKV0gJT4lIAogICAgICAgICAgICAgICAgICAgICAgICAgZ3JvdXBfYnkoaGl2LCBDU1QpICU+JSAKICAgICAgICAgICAgICAgICAgICAgICAgIHN1bW1hcmlzZShuPWxlbmd0aCh1bmlxdWUobWFyeWxhbmRfSUQpKSkpICU+JSAKICAgICAgICAgICAgICAgICAgICAgICAgIGdyb3VwX2J5KGhpdikgJT4lCiAgICAgICAgICAgICAgICAgICAgICAgICBtdXRhdGUoZnJlcSA9IG4gLyBzdW0obikpCgpjc3QuY29sczwtQ1NULmNvbFttYXRjaChzb3J0KHVuaXF1ZSh0by5wbG90JENTVCkpLCBDU1QuY29sJENTVCksICJjb2xvciJdCgp0by5wbG90IDwtIHRvLnBsb3QgJT4lCiAgZ3JvdXBfYnkoaGl2KSAlPiUKICBtdXRhdGUoeV9wb3MgPSAxLShjdW1zdW0oZnJlcSkgLSAwLjUgKiBmcmVxKSkKCnAyPC1nZ3Bsb3QodG8ucGxvdCwgYWVzKHg9aGl2LCB5PWZyZXEsIGZpbGw9Q1NULCBsYWJlbD1DU1QpKSsKICBnZW9tX2JhcihzdGF0PSJpZGVudGl0eSIsIHBvc2l0aW9uID0gImZpbGwiLCBjb2xvcj0iYmxhY2siLCBsd2Q9MC4yKSsKICBnZW9tX3RleHQoYWVzKHk9eV9wb3MpLCBzaXplPTEuNSwgY29sb3I9IndoaXRlIikrCiAgc2NhbGVfZmlsbF9tYW51YWwodmFsdWVzPWNzdC5jb2xzKSsKICB0aGVtZV9idygpKwogIHRoZW1lKGxlZ2VuZC5wb3NpdGlvbj0ibm9uZSIsIAogICAgICAgIHRleHQ9ZWxlbWVudF90ZXh0KHNpemU9NiwgY29sb3I9ImJsYWNrIiksIAogICAgICAgIGxpbmUgPSBlbGVtZW50X2xpbmUobGluZXdpZHRoID0gMC4xKSwgCiAgICAgICAgYXhpcy50aXRsZSA9IGVsZW1lbnRfdGV4dChzaXplPTUpLCAKICAgICAgICBheGlzLnRleHQgPSBlbGVtZW50X3RleHQoY29sb3I9ImJsYWNrIikpKwogIHhsYWIoIiIpKwogIHlsYWIoIlByb3BvcnRpb24gb2YgU2FtcGxlcyBpbiBDU1QiKSsKICB5bGltKGMoMCwxLjEpKSsKICBnZW9tX3NpZ25pZihjb21wYXJpc29ucyA9IGxpc3QoYygiV0xISVYiLCJISVYtTmVnYXRpdmUiKSksIAogICAgICAgICAgICAgIGFubm90YXRpb25zPXNpZ25pZmljYW5jZSwKICAgICAgICAgICAgICB5X3Bvc2l0aW9uID0gMS4wMSwgCiAgICAgICAgICAgICAgdGlwX2xlbmd0aCA9IDAsIAogICAgICAgICAgICAgIG1hcF9zaWduaWZfbGV2ZWwgPSBjKCIqKioiPTAuMDAxLCAiKioiPTAuMDEsICIqIj0wLjA1KSwgCiAgICAgICAgICAgICAgdmp1c3QgPSAwLjQsIAogICAgICAgICAgICAgIHNpemUgPSAwLjMsIAogICAgICAgICAgICAgIHRleHRzaXplID0gMykKI2dnc2F2ZShwYXN0ZSgiTWFudXNjcmlwdF9GSUdVUkVTL0ZpZ3VyZV8zYiIsIHRvZGF5MiwgIi50aWZmIiwgc2VwPSIiKSwgaGVpZ2h0ID0gMywgd2lkdGg9MiwgZHBpID0gNjAwKQpgYGAKCiMjIyMjLSBPRERTIG9mIENTVCBHSVZFTiBISVYgU1RBVFVTCmBgYHtyLCB3YXJuaW5nPUZ9CiNzdHJhdGlmeWluZyB0aGUgZGF0YSBieSBDU1QgKyBjb3ZhcmlhdGVzIGlzIHZlcnkgdG91Z2guIFdoaWNoIGNvdmFyaWF0ZXMgYXJlIGFic29sdXRlbHkgbmVlZGVkPyAKIyMgTk9URTogQ1NUIFYgPT0gMiBzYW1wbGVzIC0tPiBjb21iaW5lIHdpdGggQ1NUIEkgb3IgcmVtb3ZlPyAKIyMgTk9URTogQ1NUIElWLUMgPT0gMiBzYW1wbGVzIC0tPiByZW1vdmU/CiMjIFJlbW92ZSBjb3ZhcmlhdGVzIGR1ZSB0byBzcGFyc2VuZXNzOiBjb25kb21fZnJlcV9jYXRfZiwgY3Vycl9wdG5ycwojIyMgVVNFIFRISVMgIyMjIAojIyBEb2VzIG5vdCBjb252ZXJnZSB3aGVuIHVzaW5nIGNvbmRvbV9mcmVxX2NhdF9mIGZvciBDU1QgSVYtQgpyZXF1aXJlKG5uZXQpCnY8LWExLm1nCnYkaGl2PC1mYWN0b3IodiRoaXYsIGxldmVscz1jKCJXTEhJViIsICJISVYtTmVnYXRpdmUiKSkKdiRoaXY8LXJlbGV2ZWwodiRoaXYsIHJlZj0iSElWLU5lZ2F0aXZlIikKdiRDU1Q8LWlmZWxzZSh2JENTVCAlaW4lIGMoIkkiLCAiSUlJIiksIDAsIDEpCnYkQ1NUPC1mYWN0b3IodiRDU1QsIGxldmVscz1jKDAsMSksIGxhYmVscz1jKCJJIG9yIElJSSIsICJJViIpKQp0aGUubW9kZWw8LWdsbShDU1R+aGl2ICsgc2RfYWdlICsgcGhfZWdhICsgZ3JhdmlkaXR5ICsgcDFfc3RhdHVzICsgY3Vycl9wdG5ycyArIGJsdGVzdF9zeXByZXN1bHRzICsgcGhfZGlzY2hhcmdlICsgcGhfc29yZXNfdWxjZXJzICsgd2F0ZXIsIGRhdGEgPSB2LCBmYW1pbHkgPSAiYmlub21pYWwiKQpjPC1hcy5kYXRhLmZyYW1lKGJyb29tOjp0aWR5KHRoZS5tb2RlbCwgY29uZi5pbnQgPSBULCBleHBvbmVudGlhdGUgPSBUKSkKY1tncmVwbCgiV0xISVYiLCBjJHRlcm0pLCBdCiMgQ29tcGFyZWQgdG8gV0xISVYsIEhJVi1uZWdhdGl2ZSB3b21lbiBoYWQgdGhyZWUgdGltZXMgdGhlIG9kZHMgb2YgYmVpbmcgYXNzaWduZWQgdG8gQ1NUIEkgb3IgSUlJIGF0IGVucm9sbG1lbnQgKENTVCBJOiBhT1I6IDUuNSwgOTUlIENJOiAxLjEsIDU1LCBwYWRqPTAuMDc7IENTVCBJSUk6IGFPUjogMi45LCA5NSUgQ0k6IDEuMiwgNy44LCBwYWRqPTAuMDksIEZpZ3VyZSAzQykuIApgYGAKCiMjIyMjI0ZJRyAzQzogUHJvcG9ydGlvbiBvZiBISVYgaW4gQ1NUCmBgYHtyfQp0by5wbG90PC1hMS5tZ1thMS5tZyRDU1QgJWluJSBjKCJJIiwgIklJSSIsICJJVi1CIiksIF0KdG8ucGxvdCRDU1Q8LWlmZWxzZSh0by5wbG90JENTVCAlaW4lIGMoIkkiLCAiSUlJIiksICJJIG9yIElJSSIsIGFzLmNoYXJhY3Rlcih0by5wbG90JENTVCkpCnRvLnBsb3QkQ1NUPC1nc3ViKCJJVi1CIiwgIklWLUIgKioiLCB0by5wbG90JENTVCkKcDM8LWdncGxvdCh0by5wbG90LCBhZXMoeD1DU1QsIHk9Li5wcm9wLi4sIGZpbGw9aGl2LCBncm91cD1oaXYpKSsKICBnZW9tX2JhcihzdGF0PSJjb3VudCIsIHBvc2l0aW9uPSJkb2RnZSIsIHdpZHRoPTAuNywgY29sb3I9ImJsYWNrIiwgbHdkPTAuMSkrCiAgc2NhbGVfZmlsbF9tYW51YWwodmFsdWVzPWhpdi5jb2xzLCBsYWJlbD1jKCJXTEhJViwgbj02NCIsICJISVYoLSksIG49MTkxIiksIG5hbWU9IiIpKwogIHRoZW1lX2J3KCkrCiAgdGhlbWUobGVnZW5kLnBvc2l0aW9uID0gYygwLjIsMC45KSwgCiAgICAgICAgdGV4dD1lbGVtZW50X3RleHQoc2l6ZT02LCBjb2xvcj0iYmxhY2siKSwgCiAgICAgICAgYXhpcy50aXRsZSA9IGVsZW1lbnRfdGV4dChzaXplPTUpLCAKICAgICAgICBheGlzLnRleHQgPSBlbGVtZW50X3RleHQoY29sb3I9ImJsYWNrIiksCiAgICAgICAgbGVnZW5kLmtleS5zaXplID0gdW5pdCgwLjMsICJjbSIpLCAKICAgICAgICBsZWdlbmQuYmFja2dyb3VuZCA9IGVsZW1lbnRfYmxhbmsoKSwKICAgICAgICBsaW5lID0gZWxlbWVudF9saW5lKGxpbmV3aWR0aCA9IDAuMSkpKwogIHlsYWIoIlByb3BvcnRpb24gb2YgU2FtcGxlcyBpbiBISVYgR3JvdXAiKSsKICB4bGFiKCJDb21tdW5pdHkgU3RhdGUgVHlwZSAoQ1NUKSIpIysKICAjYW5ub3RhdGUoZ2VvbSA9ICJ0ZXh0IiwgeCA9IDMsIHkgPSAwLjc1LCBsYWJlbCA9ICIqKiIsIHNpemU9MykKICAjZ2VvbV9zaWduaWYoY29tcGFyaXNvbnMgPSBsaXN0KGMoIldMSElWIiwiSElWLU5lZ2F0aXZlIikpLCB0ZXN0ID0gImNoaS50ZXN0IiwgeV9wb3NpdGlvbiA9IC0zLjE1LCB0aXBfbGVuZ3RoID0gMCwgbWFwX3NpZ25pZl9sZXZlbCA9IGMoIioqKioiPTAuMDAxLCAiKioqIj0wLjAxLCAiKiI9MC4wNSksIHZqdXN0ID0gMC44LCBzaXplID0gMC4zLCB0ZXh0c2l6ZSA9IDMpCgojZ2dzYXZlKHBhc3RlKCJNYW51c2NyaXB0X0ZJR1VSRVMvRmlndXJlXzNjIiwgdG9kYXkyLCAiLnRpZmYiLCBzZXA9IiIpLCBoZWlnaHQgPSAzLCB3aWR0aD0zLCBkcGkgPSA2MDApCmBgYAoKIyMjIyMgRklHIDM6IFBSSU5UCmBgYHtyfQp0b3Bfcm93ID0gZ2dhcnJhbmdlKHAxLCBwMiwgcDMsIG5jb2wgPSAzLCBsYWJlbHMgPSBjKCJBLiIsICJCLiIsICJDLiIpLCBmb250LmxhYmVsID0gbGlzdChmYWNlPSJwbGFpbiIsIHNpemU9OCkpCnRvcF9yb3cKZ2dzYXZlKHBhc3RlKCJNYW51c2NyaXB0X0ZJR1VSRVMvRmlndXJlXzNfIiwgdG9kYXkyLCAiLnRpZmYiLCBzZXA9IiIpLCBoZWlnaHQgPSAzLCB3aWR0aD03LCBkcGkgPSA2MDApCmBgYAoKCiMjIyBUYXhhIFNwZWNpZmljIEFuYWx5c2lzCiAtLS0gVGhpcyByZXByZXNlbnRhdGlvbiBuZWVkcyB0byBiZSBpbXByb3ZlZCAtLSBjb2xvcnMgYW5kIGFsc28gc29ydGluZy4gCiAtLS0gdXNlIGRlc2VxIGJhc2VkIGNvZGUgaGVyZSAtLSBzZWUgemFwcGdhcHBzIHNjcmlwdHMgZm9yIGxvb3BpbmcgdGhyb3VnaCBDU1RzLgpgYGB7cn0KI2NvdW50czwtcmVhZC5jc3YoIjIwTWFyMjAyMy9ub3JtX2NvdW50c19tZ1NzX21nQ1NUXzIwTWFyMjAyMy5jc3YiLCBoZWFkZXI9VCwgc3RyaW5nc0FzRmFjdG9ycyA9IEYsIGNoZWNrLm5hbWVzID0gRikKI25hbWVzKGNvdW50cylbMV08LSJtYXJ5bGFuZF9JRCIKI2NvdW50czwtZGNhc3QoY291bnRzLm1bY291bnRzLm0kbWFyeWxhbmRfSUQgJWluJSBzYW1wbGUubGlzdCwgXSwgZm9ybXVsYSA9IG1hcnlsYW5kX0lEfnRheGEsIHZhbHVlLnZhciA9ICJjb3VudHMiLCBmdW4uYWdncmVnYXRlID0gc3VtKQpjb3VudHM8LWRjYXN0KGNvdW50cy5tLnRlbXBbY291bnRzLm0udGVtcCRtYXJ5bGFuZF9JRCAlaW4lIHNhbXBsZS5saXN0LCBdLCBmb3JtdWxhID0gbWFyeWxhbmRfSUR+dGF4YSwgdmFsdWUudmFyID0gImNvdW50cyIsIGZ1bi5hZ2dyZWdhdGUgPSBzdW0pCgojIyBSZW1vdmUgYWxsIHRheGEgZm91bmQgaW4genltbyBwb3NpdGl2ZSBjb250cm9scwptZ3NzMDwtbmFtZXMoY291bnRzKVtncmVwbCgiMCQiLCBuYW1lcyhjb3VudHMpKV0KY291bnRzPC1jb3VudHNbLCFuYW1lcyhjb3VudHMpICVpbiUgYygiRW50ZXJvY29jY3VzX2ZhZWNhbGlzIiwgIkVzY2hlcmljaGlhX2NvbGkiLCAiTGFjdG9iYWNpbGx1c19mZXJtZW50dW0iLCAiU2FsbW9uZWxsYV9lbnRlcmljYSIsICJTdGFwaHlsb2NvY2N1c19hdXJldXMiLCAiQ2FuZGlkYXR1c19QZWxhZ2liYWN0ZXIiLCBtZ3NzMCwgdG8ucm0pXQoKcm93bmFtZXMoY291bnRzKTwtY291bnRzJG1hcnlsYW5kX0lECmNvdW50cyRtYXJ5bGFuZF9JRDwtTlVMTApjb3VudHMuYWxsPC1jb3VudHMKCnNhbXBsZXM8LWExLm1nJG1hcnlsYW5kX0lECmNvdW50czwtY291bnRzLmFsbFtyb3duYW1lcyhjb3VudHMuYWxsKSAlaW4lIHNhbXBsZXMsIF0KbmFtZXMoY291bnRzKTwtZ3N1YigiX1xcLiIsICIgIiwgbmFtZXMoY291bnRzKSkKbmFtZXMoY291bnRzKTwtZ3N1YigiXyIsICIgIiwgbmFtZXMoY291bnRzKSkKbmFtZXMoY291bnRzKTwtZ3N1YihwYXR0ZXJuID0gIlxcLlxcLiIsIHJlcGxhY2VtZW50ID0gIiAiLCBuYW1lcyhjb3VudHMpKQpuYW1lcyhjb3VudHMpPC1nc3ViKHBhdHRlcm4gPSAiXFwuIiwgcmVwbGFjZW1lbnQgPSAiICIsIG5hbWVzKGNvdW50cykpCmNvdW50czwtY291bnRzWywhaXMubmEobmFtZXMoY291bnRzKSldCmNvdW50cyRtYXJ5bGFuZF9JRDwtcm93bmFtZXMoY291bnRzKQoKZC5tPC1yZXNoYXBlMjo6bWVsdChjb3VudHMsIGlkLnZhcnMgPSAibWFyeWxhbmRfSUQiLCB2YXJpYWJsZS5uYW1lID0gIlRheG9uIiwgdmFsdWUubmFtZSA9ICJjb3VudCIsIG5hLnJtID0gVCkKZC5tPC1tZXJnZShkLm0sIGExLm1nWyxjKCJtYXJ5bGFuZF9JRCIsICJoaXYiKV0sIGFsbC54PVRSVUUpCgpzYW1wbGVzLngudGF4b248LWQubVtkLm0kY291bnQgPiAwLCBdICU+JSBkcGx5cjo6Z3JvdXBfYnkoVGF4b24sIGhpdikgJT4lIGRwbHlyOjpzdW1tYXJpc2UoblNhbXBsZXM9bGVuZ3RoKHVuaXF1ZShtYXJ5bGFuZF9JRCkpKQpwcm9wLnRhYmxlKHRhYmxlKGExLm1nJGhpdikpIAoKc2FtcGxlcy54LnRheG9uLmRmPC1yZXNoYXBlMjo6ZGNhc3Qoc2FtcGxlcy54LnRheG9uLCBUYXhvbn5oaXYsIHZhbHVlLnZhciA9ICJuU2FtcGxlcyIpCnNhbXBsZXMueC50YXhvbi5kZiRgTkFgPC1OVUxMCnNhbXBsZXMueC50YXhvbi5kZltpcy5uYShzYW1wbGVzLngudGF4b24uZGYpXTwtMApzYW1wbGVzLngudGF4b24uZGYkVG90YWw8LXNhbXBsZXMueC50YXhvbi5kZiRgV0xISVZgK3NhbXBsZXMueC50YXhvbi5kZiRgSElWLU5lZ2F0aXZlYAoKIyMgVGVzdCBvbmx5IHRob3NlIHRheGEgd2hpY2ggYXJlIHByZXNlbnQgaW4gYXQgbGVhc3QgNSUgb2Ygc2FtcGxlcyA9ICBtaW4uIDggc2FtcGxlcyA9IDEwNyB0YXhhCmtlZXA8LWFzLnZlY3RvcihzYW1wbGVzLngudGF4b24uZGZbc2FtcGxlcy54LnRheG9uLmRmJFRvdGFsID49IDAuMSpucm93KGNvdW50cyksICJUYXhvbiJdKQpjPC1yZXNoYXBlMjo6ZGNhc3QoZC5tW2QubSRUYXhvbiAlaW4lIGtlZXAsIF0sIFRheG9ufm1hcnlsYW5kX0lELCB2YWx1ZS52YXIgPSAiY291bnQiLCBmdW4uYWdncmVnYXRlID0gc3VtKQpyb3duYW1lcyhjKTwtYyRUYXhvbgpjPC1jWy0xXQpjPC1jWywgY29sU3VtcyhjKSA+IDBdCmM8LWNbcm93U3VtcyhjKSA+IDAsICBdCgptZXRhLnN5bTwtYTEubWdbLGMoIm1hcnlsYW5kX0lEIiwgY292YXIsICJoaXYiKV0KbWV0YS5zeW08LW1ldGEuc3ltW21ldGEuc3ltJG1hcnlsYW5kX0lEICVpbiUgbmFtZXMoYyksIF0KbWV0YS5zeW08LW1ldGEuc3ltW2NvbXBsZXRlLmNhc2VzKG1ldGEuc3ltKSwgXQpjPC1jWyxjb2xuYW1lcyhjKSAlaW4lIG1ldGEuc3ltJG1hcnlsYW5kX0lEXQoKb2JqLnN5bTwtREVTZXFEYXRhU2V0RnJvbU1hdHJpeChjb3VudERhdGEgPSByb3VuZChjKSwgY29sRGF0YSA9IG1ldGEuc3ltLCBkZXNpZ249fiBzZF9hZ2UgKyBwaF9lZ2EgKyBncmF2aWRpdHkgKyBwMV9zdGF0dXMgKyBjdXJyX3B0bnJzICsgYmx0ZXN0X3N5cHJlc3VsdHMgKyBwaF9kaXNjaGFyZ2UgKyBwaF9zb3Jlc191bGNlcnMgKyB3YXRlciArIGhpdikKI29iai5zeW0kaGl2PC1yZWxldmVsKG9iai5zeW0kaGl2LCByZWY9IjAiKQpvYmouc3ltID0gZXN0aW1hdGVTaXplRmFjdG9ycyggb2JqLnN5bSwgdHlwZT0icG9zY291bnRzIiApCm9iai5zeW0gPSBlc3RpbWF0ZURpc3BlcnNpb25zKCBvYmouc3ltLCBmaXRUeXBlPSJsb2NhbCIgKQoKIyMgbGltbWEgKyB2b29tCmhpdjwtbWFrZS5uYW1lcyhtZXRhLnN5bSRoaXYpCnNkX2FnZTwtbWV0YS5zeW0kc2RfYWdlCnBoX2VnYTwtbWV0YS5zeW0kcGhfZWdhCmdyYXZpZGl0eTwtbWFrZS5uYW1lcyhtZXRhLnN5bSRncmF2aWRpdHkpCnAxX3N0YXR1czwtbWFrZS5uYW1lcyhtZXRhLnN5bSRwMV9zdGF0dXMpCmN1cnJfcHRucnM8LW1ha2UubmFtZXMobWV0YS5zeW0kY3Vycl9wdG5ycykKYmx0ZXN0X3N5cHJlc3VsdHM8LW1ha2UubmFtZXMobWV0YS5zeW0kYmx0ZXN0X3N5cHJlc3VsdHMpCnBoX2Rpc2NoYXJnZTwtbWFrZS5uYW1lcyhtZXRhLnN5bSRwaF9kaXNjaGFyZ2UpCnBoX3NvcmVzX3VsY2VyczwtbWFrZS5uYW1lcyhtZXRhLnN5bSRwaF9zb3Jlc191bGNlcnMpCndhdGVyPC1tYWtlLm5hbWVzKG1ldGEuc3ltJHdhdGVyKQoKI2Rlc2lnbjwtbW9kZWwubWF0cml4KH4gc2RfYWdlICsgcGhfZWdhICsgZ3JhdmlkaXR5ICsgcDFfc3RhdHVzICsgY3Vycl9wdG5ycyArIGJsdGVzdF9zeXByZXN1bHRzICsgcGhfZGlzY2hhcmdlICsgcGhfc29yZXNfdWxjZXJzICsgd2F0ZXIgKyBoaXYpCiMjIE1vZGVsIGRvZXMgbm90IGNvbnZlcmdlIHdoZW4gaW5jbHVkaW5nIGN1cnJfcHRucnMKZGVzaWduPC1tb2RlbC5tYXRyaXgofnNkX2FnZSArIHBoX2VnYSArIGdyYXZpZGl0eSArIHAxX3N0YXR1cyArIGJsdGVzdF9zeXByZXN1bHRzICsgcGhfZGlzY2hhcmdlICsgcGhfc29yZXNfdWxjZXJzICsgd2F0ZXIgKyBoaXYpCgpuYyA8LSBjb3VudHMob2JqLnN5bSwgbm9ybWFsaXplZD1UUlVFKQpkMCA8LSBER0VMaXN0KG5jKQpkMCA8LSBjYWxjTm9ybUZhY3RvcnMoZDApCnY8LXZvb20oZDAsIGRlc2lnbiwgcGxvdD1UKQpmaXQ8LWxtRml0KHYsIGRlc2lnbikKdG1wIDwtIGVCYXllcyhmaXQpCgp0b3AudGFibGUgPC0gdG9wVGFibGUodG1wLCBhZGp1c3QubWV0aG9kID0gIkJIIiwgc29ydC5ieSA9ICJGIiwgbiA9IEluZikKdG9wLnRhYmxlW3RvcC50YWJsZSRhZGouUC5WYWwgPCAwLjAxLCBdCnRvcC50YWJsZSR0YXhhPC1yb3duYW1lcyh0b3AudGFibGUpCnRheGEuYWxsLnJlc3VsdHMucHJlPC10b3AudGFibGUKdGF4YS5hbGwucmVzdWx0cy5wcmU8LXVuaXF1ZSh0YXhhLmFsbC5yZXN1bHRzLnByZSkKdGF4YS5hbGwucmVzdWx0cy5wcmUkYXNzb2NpYXRpb248LWlmZWxzZSh0YXhhLmFsbC5yZXN1bHRzLnByZSRhZGouUC5WYWwgPCAwLjAxMSwgaWZlbHNlKHRheGEuYWxsLnJlc3VsdHMucHJlJGhpdldMSElWIDwgMCwgIlBvc2l0aW9uIEFzc29jaWF0aW9uIHdpdGggSElWKC0pIHN0YXR1cyIsICJQb3NpdGlvbiBBc3NvY2lhdGlvbiB3aXRoIFdMSElWIiksICJOb3QgU2lnbmlmaWNhbnQiKQp0YXhhLmFsbC5yZXN1bHRzLnByZSR0YXhhPC1mYWN0b3IodGF4YS5hbGwucmVzdWx0cy5wcmUkdGF4YSkKYGBgCgojIyMjIEZJRyA0OiBUYXhhIGJ5IEhJViBzZXJvc3RhdHVzCmBgYHtyfQojIyBUaGUgcmVmZXJlbmNlIGhlcmUgaXMgSElWLSAiMCIuCmdncGxvdCh0YXhhLmFsbC5yZXN1bHRzLnByZVt0YXhhLmFsbC5yZXN1bHRzLnByZSRhZGouUC5WYWwgPCAwLjAxMSAmICFpcy5uYSh0YXhhLmFsbC5yZXN1bHRzLnByZSRhZGouUC5WYWwpICYgIXRheGEuYWxsLnJlc3VsdHMucHJlJHRheGEgJWluJSAiTkEiLCBdLCBhZXMoeD1oaXZXTEhJViwgeT1yZW9yZGVyKHRheGEsaGl2V0xISVYpLCBmaWxsPWFzc29jaWF0aW9uKSkrCiAgZ2VvbV9iYXIoc3RhdD0iaWRlbnRpdHkiLCBjb2xvcj0iYmxhY2siLCBsd2Q9MC4xKSsKICB4bGFiKCJMb2ctRm9sZCBEaWZmZXJlbmNlIGluIG1nU3MgQWJ1bmRhbmNlXG4oV0xISVYgLyBISVYtTmVnYXRpdmUpIikrCiAgc2NhbGVfZmlsbF9tYW51YWwodmFsdWVzPXJldihhcy52ZWN0b3IoaGl2LmNvbHMpKSwgbmFtZT0iQXNzb2NpYXRlZCB3aXRoOiIsIGxhYmVscz1jKCJISVYoLSkiLCAiV0xISVYiKSkrCiAgdGhlbWVfYncoKSsKICB0aGVtZSh0ZXh0PWVsZW1lbnRfdGV4dChzaXplPTUsIGNvbG9yPSJibGFjayIpLCAKICAgICAgICBheGlzLnRleHQgPSBlbGVtZW50X3RleHQoY29sb3I9ImJsYWNrIiksCiAgICAgICAgYXhpcy50ZXh0LnkgPSBlbGVtZW50X3RleHQoZmFjZT0iaXRhbGljIiwgImJsYWNrIiwgZmFtaWx5PSJBcmlhbCIpLCAKICAgICAgICBsZWdlbmQucG9zaXRpb24gPSAibm9uZSIsIAogICAgICAgIGxlZ2VuZC5iYWNrZ3JvdW5kID0gZWxlbWVudF9yZWN0KGZpbGw9InRyYW5zcGFyZW50IiksCiAgICAgICAgYXhpcy50aWNrcyA9IGVsZW1lbnRfbGluZShsaW5ld2lkdGggPSAwLjEpLCAKICAgICAgICBsZWdlbmQua2V5LnNpemUgPSB1bml0KDAuNCwgImNtIiksIAogICAgICAgIGxpbmUgPSBlbGVtZW50X2xpbmUobGluZXdpZHRoID0gMC41KSkrCiAgeWxhYigiIikKZ2dzYXZlKHBhc3RlKCJNYW51c2NyaXB0X0ZJR1VSRVMvRmlndXJlXzRfIix0b2RheTIsICIudGlmZiIsIHNlcD0iIiksIGhlaWdodD00LCB3aWR0aD0zLCBkcGkgPSA2MDApCndyaXRlLmNzdih0YXhhLmFsbC5yZXN1bHRzLnByZSwgcGFzdGUoIkFuYWx5c2lzX0ZpZ3VyZXMvVGFibGVfbWdTc19BaW0xXyIsdG9kYXkyLCIuY3N2Iiwgc2VwPSIiKSwgcXVvdGU9RikKYGBgCiAKIyBBaW0gMiwgUHJFUCBBUlQgTG9uZwojIyBEYXRhICYgQ292YXJpYXRlcyAoYXQgZW5yb2xsbWVudCkKYGBge3J9CiMjIENPVkFSSUFURVMKIyBGSVhFRCBFRkZFQ1RTIC0gdG8gcmVkdWNlIHRoZSBjaGFuY2VzIHRoYXQgYW4gSElWL21pY3JvYmlvbWUgYXNzb2NpYXRpb24gaXMgY29uZm91bmRlZCBieSBzb21ldGhpbmcgZWxzZS4KbGFiZWwoYTIubWckc2RfYWdlKTwtIkFnZSBhdCBlbnJvbGxtZW50ICh5ZWFycykiCmNvdmFyPC1jKCJzZF9hZ2UiKQoKIyMgcGhfZWdhOiBlc3RpbWF0ZWQgZ2VzdGF0aW9uYWwgYWdlIGF0IGVucm9sbG1lbnQ6IENvbnRpbnVvdXMgCmxhYmVsKGEyLm1nJHBoX2VnYSk8LSJHZXN0YXRpb25hbCBhZ2UgYXQgZW5yb2xsbWVudCAod2Vla3MpIgpjb3ZhcjwtYXBwZW5kKGNvdmFyLCAicGhfZWdhIikKCiMgR3JhdmlkaXR5OiAiMD1ObyBwcmlvciBwcmVnbmFuY2llcywgMT1BdCBsZWFzdCBvbmUgcHJpb3IgcHJlZ25hbmN5IgphMi5tZyRncmF2aWRpdHk8LWZhY3RvcihhMi5tZyRncmF2aWRpdHksIGxldmVscz1jKDAsIDEpLCBsYWJlbHM9YygiTm8gcHJpb3IgcHJlZ25hbmNpZXMiLCAiQXQgbGVhc3Qgb25lIHByaW9yIHByZWduYW5jeSIpKQpsYWJlbChhMi5tZyRncmF2aWRpdHkpPC0iR3JhdmlkaXR5Igpjb3ZhcjwtYXBwZW5kKGNvdmFyLCAiZ3JhdmlkaXR5IikKCiMgUHJpbWFyeSBwYXJ0bmVyIEhJViBzdGF0dXM6IDA9SElWLW5lZ2F0aXZlLCAxPUhJVi1wb3NpdGl2ZSwgMj1JbmRldGVybWluYXRlLCAzPVBhcnRuZXIgbmV2ZXIgdGVzdGVkIGZvciBISVYsIDk5OD1JIGRvbid0IGtub3csIDk5OT1ObyByZXNwb25zZQphMi5tZyRwMV9zdGF0dXNbYTIubWckcDFfc3RhdHVzICVpbiUgYygzLCA5OTgpXTwtMwphMi5tZyRwMV9zdGF0dXM8LWZhY3RvcihhMi5tZyRwMV9zdGF0dXMsIGxldmVscz1jKDAsIDEsIDMpLCBsYWJlbHM9YygiSElWLW5lZ2F0aXZlIiwgIkhJVi1wb3NpdGl2ZSIsICJEb24ndCBrbm93IikpCmxhYmVsKGEyLm1nJHAxX3N0YXR1cyk8LSJQcmltYXJ5IHBhcnRuZXIgSElWIHNlcm9zdGF0dXMgKiIKY292YXI8LWFwcGVuZChjb3ZhciwgInAxX3N0YXR1cyIpCgojIE51bWJlciBvZiBwYXJ0bmVycyBpbiBwYXN0IHRocmVlIG1vbnRoczogIjA9Tm8gcGFydG5lcnMsIDE9T25lIHBhcnRuZXIsIDI9TXVsdGlwbGUgcGFydG5lcnMiCmEyLm1nJGN1cnJfcHRucnM8LWZhY3RvcihhMi5tZyRjdXJyX3B0bnJzLCBsZXZlbHM9YygwLCAxLCAyKSwgbGFiZWxzPWMoIjAiLCAiMSIsICJNdWx0aXBsZSIpKSAKbGFiZWwoYTIubWckY3Vycl9wdG5ycyk8LSJOdW1iZXIgb2YgcGFydG5lcnMgaW4gcGFzdCB0aHJlZSBtb250aHMiCmNvdmFyPC1hcHBlbmQoY292YXIsICJjdXJyX3B0bnJzIikKCiNGSVggYTIubWckY29uZG9tX2ZyZXFfY2F0IC4uLiB0cnkgZ3JvdXBpbmcgc29tZXRpbWVzIHdpdGggbmV2ZXIgLSBob3cgZG9lcyB0aGlzIGNoYW5nZT8KI0NvbnNpc3RlbnQgY29uZG9tIHVzZSB3aXRoIHByaW1hcnkgcGFydG5lciBpbiBwYXN0IDMwIGRheXM6ICIwPU5ldmVyLCAxPVNvbWV0aW1lcywgMj1Db25zaXN0ZW50IgphMi5tZyRjb25kb21fZnJlcV9jYXRfZjwtYTIubWckY29uZG9tX2ZyZXFfY2F0CmEyLm1nJGNvbmRvbV9mcmVxX2NhdF9mW2EyLm1nJGNvbmRvbV9mcmVxX2NhdF9mICVpbiUgYygwLCAxKV08LTAKYTIubWckY29uZG9tX2ZyZXFfY2F0X2ZbYTIubWckY29uZG9tX2ZyZXFfY2F0X2YgJWluJSAyXTwtMgphMi5tZyRjb25kb21fZnJlcV9jYXRfZltpcy5uYShhMi5tZyRjb25kb21fZnJlcV9jYXQpXTwtMwphMi5tZyRjb25kb21fZnJlcV9jYXRfZjwtZmFjdG9yKGEyLm1nJGNvbmRvbV9mcmVxX2NhdF9mLCBsZXZlbHM9YygwLCAyLCAzKSwgbGFiZWxzPWMoIk5ldmVyIG9yIFNvbWV0aW1lcyIsICJDb25zaXN0ZW50IiwgIkFic3RhaW5lZCIpKQphMi5tZyRjb25kb21fZnJlcV9jYXRfZjwtcmVsZXZlbChhMi5tZyRjb25kb21fZnJlcV9jYXRfZiwgcmVmPSJBYnN0YWluZWQiKQpsYWJlbChhMi5tZyRjb25kb21fZnJlcV9jYXRfZik8LSJDb25zaXN0ZW50IGNvbmRvbSB1c2Ugd2l0aCBwcmltYXJ5IHBhcnRuZXIgaW4gcGFzdCAzMCBkYXlzIF4iCiMgY292YXI8LWFwcGVuZChjb3ZhciwgImNvbmRvbV9mcmVxX2NhdF9mIikKCiMjIHBoX3N5cGhpbGlzX3RvbGQ6IHNlbGYtcmVwb3J0IHN5cGhpbGlzIHBhc3QgMyBtb250aHMuIENBVAojIFJlc3VsdHMgb2Ygc3lwaGlsaXMgdGVzdCBkdXJpbmcgaW5kZXggcHJlZ25hbmN5IChhbnRlbmF0YWwgcmVjb3JkKTogIjA9Tm9ucmVhY3RpdmUsIDE9UmVhY3RpdmUsIDk5OT1ObyB0ZXN0IHJlc3VsdHMgcmVjb3JkZWQiCmEyLm1nJGJsdGVzdF9zeXByZXN1bHRzPC1mYWN0b3IoYTIubWckYmx0ZXN0X3N5cHJlc3VsdHMsIGxldmVscz1jKDAsIDEpLCBsYWJlbHMgPSBjKCJObyIsICJZZXMiKSkKbGFiZWwoYTIubWckYmx0ZXN0X3N5cHJlc3VsdHMpPC0iRGlhZ25vc2VkIHdpdGggc3lwaGlsaXMgaW4gcGFzdCAzIG1vbnRocyIKY292YXI8LWFwcGVuZChjb3ZhciwgImJsdGVzdF9zeXByZXN1bHRzIikKCiMgQWJub3JtYWwgdmFnaW5hbCBkaXNjaGFyZ2Ugb2JzZXJ2ZWQgaW4gcGFzdCAzIG1vbnRocyAoc2VsZi1yZXBvcnQpOiAiMD1ObywgMT1ZZXMsIDk5OD1JIGRvbid0IGtub3csIDk5OT1ObyBSZXNwb25zZSIKYTIubWckcGhfZGlzY2hhcmdlPC1mYWN0b3IoYTIubWckcGhfZGlzY2hhcmdlLCBsZXZlbHM9YygwLCAxKSwgbGFiZWxzPWMoIk5vIiwgIlllcyIpKQpsYWJlbChhMi5tZyRwaF9kaXNjaGFyZ2UpPC0iQWJub3JtYWwgdmFnaW5hbCBkaXNjaGFyZ2Ugb2JzZXJ2ZWQgaW4gcGFzdCAzIG1vbnRocyAoc2VsZi1yZXBvcnQpIgpjb3ZhcjwtYXBwZW5kKGNvdmFyLCAicGhfZGlzY2hhcmdlIikKCiMgR2VuaXRhbCB1bGNlcnMgb2JzZXJ2ZWQgaW4gcGFzdCAzIG1vbnRocyAoc2VsZi1yZXBvcnQpOiAiMD1ObywgMT1ZZXMsIDk5OD1JIGRvbid0IGtub3csIDk5OT1ObyBSZXNwb25zZSIKYTIubWckcGhfc29yZXNfdWxjZXJzPC1mYWN0b3IoYTIubWckcGhfc29yZXNfdWxjZXJzLCBsZXZlbHM9YygwLCAxKSwgbGFiZWxzPWMoIk5vIiwgIlllcyIpKQpsYWJlbChhMi5tZyRwaF9zb3Jlc191bGNlcnMpPC0iR2VuaXRhbCB1bGNlcnMgb2JzZXJ2ZWQgaW4gcGFzdCAzIG1vbnRocyAoc2VsZi1yZXBvcnQpIgpjb3ZhcjwtYXBwZW5kKGNvdmFyLCAicGhfc29yZXNfdWxjZXJzIikKCiMgUnVubmluZyB3YXRlciBpbiBob21lOiAiMD1ObywgMT1ZZXMiCmEyLm1nJHdhdGVyPC1mYWN0b3IoYTIubWckd2F0ZXIsIGxldmVscz1jKDAsIDEpLCBsYWJlbHM9YygiTm8iLCAiWWVzIikpCmxhYmVsKGEyLm1nJHdhdGVyKTwtIlJ1bm5pbmcgd2F0ZXIgaW4gaG9tZSIKY292YXI8LWFwcGVuZChjb3ZhciwgIndhdGVyIikKCiMgUHJlZ25hbmN5IE91dGNvbWUKYTIubWckcHJlZ19vdXRjb21lX2NsZWFuPC1mYWN0b3IoaWZlbHNlKGdyZXBsKCJQcmV0ZXJtIERlbGl2ZXJ5IiwgYTIubWckcHJlZ19vdXRjb21lKSwgIlByZXRlcm0gRGVsaXZlcnkiLCBpZmVsc2UoZ3JlcGwoIlRlcm0gRGVsaXZlcnkiLCBhMi5tZyRwcmVnX291dGNvbWUpLCAiVGVybSBEZWxpdmVyeSIsIE5BKSksIGxldmVscz1jKCJUZXJtIERlbGl2ZXJ5IiwgIlByZXRlcm0gRGVsaXZlcnkiKSkKYTIubWckcHJlZ19vdXRjb21lX2NsZWFuPC1yZWxldmVsKGEyLm1nJHByZWdfb3V0Y29tZV9jbGVhbiwgcmVmPSJUZXJtIERlbGl2ZXJ5IikKbGFiZWwoYTIubWckcHJlZ19vdXRjb21lX2NsZWFuKTwtIlByZWduYW5jeSBPdXRjb21lIgoKbGFiZWwoYTIubWckZGF0ZV9kaWZmKTwtIkRheXMgYmV0d2VlbiBNMCBhbmQgTTEiCgpjb3ZhcnM8LXBhc3RlKGNvdmFyLCBjb2xsYXBzZT0iICsgIikKYGBgCgojIyBUYWJsZTEgKGF0IGVucm9sbG1lbnQpCmBgYHtyfQphMi5lbnJvbGw8LXVuaXF1ZShhMi5tZ1thMi5tZyRzd2FiICVpbiUgIjEiLCAhbmFtZXMoYTIubWcpICVpbiUgYygiU3BlY19EYXRlIiwgIm1hcnlsYW5kX0lEIiwgIm1nQ1NUMiIsICJBUlRfUFJFUF9kYXRlIiwgIkNTVCIsICJzd2FiIildKQoKIyBGSVhFRCBFRkZFQ1RTIC0gdG8gcmVkdWNlIHRoZSBjaGFuY2VzIHRoYXQgYW4gSElWL21pY3JvYmlvbWUgYXNzb2NpYXRpb24gaXMgY29uZm91bmRlZCBieSBzb21ldGhpbmcgZWxzZS4KbGFiZWwoYTIuZW5yb2xsJHNkX2FnZSk8LSJBZ2UgYXQgZW5yb2xsbWVudCAoeWVhcnMpIgoKIyMgcGhfZWdhOiBlc3RpbWF0ZWQgZ2VzdGF0aW9uYWwgYWdlIGF0IGVucm9sbG1lbnQ6IENvbnRpbnVvdXMgCmxhYmVsKGEyLmVucm9sbCRwaF9lZ2EpPC0iR2VzdGF0aW9uYWwgYWdlIGF0IGVucm9sbG1lbnQgKHdlZWtzKSIKCiMgR3JhdmlkaXR5OiAiMD1ObyBwcmlvciBwcmVnbmFuY2llcywgMT1BdCBsZWFzdCBvbmUgcHJpb3IgcHJlZ25hbmN5IgpsYWJlbChhMi5lbnJvbGwkZ3JhdmlkaXR5KTwtIkdyYXZpZGl0eSIKCiMgUHJpbWFyeSBwYXJ0bmVyIEhJViBzdGF0dXM6IDA9SElWLW5lZ2F0aXZlLCAxPUhJVi1wb3NpdGl2ZSwgMj1JbmRldGVybWluYXRlLCAzPVBhcnRuZXIgbmV2ZXIgdGVzdGVkIGZvciBISVYsIDk5OD1JIGRvbid0IGtub3csIDk5OT1ObyByZXNwb25zZQpsYWJlbChhMi5lbnJvbGwkcDFfc3RhdHVzKTwtIlByaW1hcnkgcGFydG5lciBISVYgc2Vyb3N0YXR1cyAqIgoKCiMgTnVtYmVyIG9mIHBhcnRuZXJzIGluIHBhc3QgdGhyZWUgbW9udGhzOiAiMD1ObyBwYXJ0bmVycywgMT1PbmUgcGFydG5lciwgMj1NdWx0aXBsZSBwYXJ0bmVycyIKbGFiZWwoYTIuZW5yb2xsJGN1cnJfcHRucnMpPC0iTnVtYmVyIG9mIHBhcnRuZXJzIGluIHBhc3QgdGhyZWUgbW9udGhzIgoKI0ZJWCBhMi5lbnJvbGwkY29uZG9tX2ZyZXFfY2F0IC4uLiB0cnkgZ3JvdXBpbmcgc29tZXRpbWVzIHdpdGggbmV2ZXIgLSBob3cgZG9lcyB0aGlzIGNoYW5nZT8KI0NvbnNpc3RlbnQgY29uZG9tIHVzZSB3aXRoIHByaW1hcnkgcGFydG5lciBpbiBwYXN0IDMwIGRheXM6ICIwPU5ldmVyLCAxPVNvbWV0aW1lcywgMj1Db25zaXN0ZW50IgpsYWJlbChhMi5lbnJvbGwkY29uZG9tX2ZyZXFfY2F0X2YpPC0iQ29uc2lzdGVudCBjb25kb20gdXNlIHdpdGggcHJpbWFyeSBwYXJ0bmVyIGluIHBhc3QgMzAgZGF5cyBeIgoKIyMgcGhfc3lwaGlsaXNfdG9sZDogc2VsZi1yZXBvcnQgc3lwaGlsaXMgcGFzdCAzIG1vbnRocy4gQ0FUCiMgUmVzdWx0cyBvZiBzeXBoaWxpcyB0ZXN0IGR1cmluZyBpbmRleCBwcmVnbmFuY3kgKGFudGVuYXRhbCByZWNvcmQpOiAiMD1Ob25yZWFjdGl2ZSwgMT1SZWFjdGl2ZSwgOTk5PU5vIHRlc3QgcmVzdWx0cyByZWNvcmRlZCIKbGFiZWwoYTIuZW5yb2xsJGJsdGVzdF9zeXByZXN1bHRzKTwtIkRpYWdub3NlZCB3aXRoIHN5cGhpbGlzIGluIHBhc3QgMyBtb250aHMiCgojIEFibm9ybWFsIHZhZ2luYWwgZGlzY2hhcmdlIG9ic2VydmVkIGluIHBhc3QgMyBtb250aHMgKHNlbGYtcmVwb3J0KTogIjA9Tm8sIDE9WWVzLCA5OTg9SSBkb24ndCBrbm93LCA5OTk9Tm8gUmVzcG9uc2UiCmxhYmVsKGEyLmVucm9sbCRwaF9kaXNjaGFyZ2UpPC0iQWJub3JtYWwgdmFnaW5hbCBkaXNjaGFyZ2Ugb2JzZXJ2ZWQgaW4gcGFzdCAzIG1vbnRocyAoc2VsZi1yZXBvcnQpIgoKIyBHZW5pdGFsIHVsY2VycyBvYnNlcnZlZCBpbiBwYXN0IDMgbW9udGhzIChzZWxmLXJlcG9ydCk6ICIwPU5vLCAxPVllcywgOTk4PUkgZG9uJ3Qga25vdywgOTk5PU5vIFJlc3BvbnNlIgpsYWJlbChhMi5lbnJvbGwkcGhfc29yZXNfdWxjZXJzKTwtIkdlbml0YWwgdWxjZXJzIG9ic2VydmVkIGluIHBhc3QgMyBtb250aHMgKHNlbGYtcmVwb3J0KSIKCiMgUnVubmluZyB3YXRlciBpbiBob21lOiAiMD1ObywgMT1ZZXMiCmxhYmVsKGEyLmVucm9sbCR3YXRlcik8LSJSdW5uaW5nIHdhdGVyIGluIGhvbWUiCgpsYWJlbChhMi5lbnJvbGwkcHJlZ19vdXRjb21lX2NsZWFuKTwtIlByZWduYW5jeSBPdXRjb21lIgoKbGFiZWwoYTIuZW5yb2xsJGRhdGVfZGlmZik8LSJEYXlzIGJldHdlZW4gTTAgYW5kIE0xIgoKdGFibGUxKH4gc2RfYWdlICsgcGhfZWdhICsgZ3JhdmlkaXR5ICsgcDFfc3RhdHVzICsgY3Vycl9wdG5ycyArIGNvbmRvbV9mcmVxX2NhdF9mICsgYmx0ZXN0X3N5cHJlc3VsdHMgKyBwaF9kaXNjaGFyZ2UgKyBwaF9zb3Jlc191bGNlcnMgKyB3YXRlciArIHByZWdfb3V0Y29tZV9jbGVhbiArIGRhdGVfZGlmZiB8IGhpdiwgZGF0YT1hMi5lbnJvbGwsIG92ZXJhbGw9RiwgZXh0cmEuY29sPWxpc3QoYFAtdmFsdWVgPXB2YWx1ZSkpCmBgYAoKCiMjIEFscGhhIERpdmVyc2l0eSAmIEFSVCB2cyBQckVQCmBgYHtyfQpjb3VudHM8LWFzLmRhdGEuZnJhbWUocmVzaGFwZTI6OmRjYXN0KGNvdW50cy5tLCBtYXJ5bGFuZF9JRH50YXhhMiwgdmFsdWUudmFyPSJjb3VudHMiLCBmdW4uYWdncmVnYXRlID0gc3VtKSkKcm93bmFtZXMoY291bnRzKTwtY291bnRzJG1hcnlsYW5kX0lECmNvdW50cyRtYXJ5bGFuZF9JRDwtTlVMTApleHA8LXBoeWxvc2VxKG90dV90YWJsZShyb3VuZChjb3VudHNbcm93bmFtZXMoY291bnRzKSAlaW4lIGEyLm1nJG1hcnlsYW5kX0lELCBdKSwgdGF4YV9hcmVfcm93cyA9IEYpKQpjb3VudHMkbWFyeWxhbmRfSUQ8LXJvd25hbWVzKGNvdW50cykKcm93bmFtZXMoYTIubWcpPC1hMi5tZyRtYXJ5bGFuZF9JRApleHA8LW1lcmdlX3BoeWxvc2VxKGV4cCwgc2FtcGxlX2RhdGEoYTIubWcpKQpyaWNoPC1lc3RpbWF0ZV9yaWNobmVzcyggZXhwLCBtZWFzdXJlcyA9IGMoIkNoYW8xIiwgIlNoYW5ub24iKSkKcmljaCRtYXJ5bGFuZF9JRDwtcm93bmFtZXMocmljaCkKCmEyLm1nLnJpY2g8LW1lcmdlKGEyLm1nLCByaWNoLCBhbGw9VFJVRSkKCmEyLm1nPC1hMi5tZy5yaWNoWyxjKCJQSUQiLCAibWFyeWxhbmRfSUQiLCBjb3ZhciwgIlNoYW5ub24iLCAic3dhYiIsICJoaXYiLCAidHJlYXRtZW50IiwgIkNTVCIsICJkYXRlX2RpZmYiKV0KI2EyLm1nPC1hMi5tZ1tjb21wbGV0ZS5jYXNlcyhhMi5tZyksIF0gIyMgbG9zdCAxNCBzYW1wbGVzIGRvIHRvIGluY29tcGxldGUgY2FzZXMuCmBgYAoKIyMjIC0gRGl2ZXJzaXR5IGJ5IHN3YWIgYW5kIHRyZWF0bWVudApgYGB7cn0KIyBJbiBBUlQ6IG9ubHkgY3Vycl9wdG5ycz0xCnRoZS5tb2RlbDE8LWxtZXIoZm9ybXVsYSA9IFNoYW5ub24gfiBzd2FiICsgc2RfYWdlICsgcGhfZWdhICsgZ3JhdmlkaXR5ICsgcDFfc3RhdHVzICsgYmx0ZXN0X3N5cHJlc3VsdHMgKyBwaF9kaXNjaGFyZ2UgKyBwaF9zb3Jlc191bGNlcnMgKyB3YXRlciArIGRhdGVfZGlmZiArICgxfFBJRCksIGRhdGEgPSBhMi5tZ1thMi5tZyR0cmVhdG1lbnQgJWluJSAiQVJUIiwgXSkKYW5vdmEodGhlLm1vZGVsMSkKcHZhbHVlMTwtc3VtbWFyeSh0aGUubW9kZWwxKSRjb2VmZmljaWVudHNbLCAiUHIoPnx0fCkiXVsic3dhYjIiXQoKdGhlLm1vZGVsMjwtbG1lcihmb3JtdWxhID0gU2hhbm5vbiB+IHN3YWIgKyBzZF9hZ2UgKyBwaF9lZ2EgKyBncmF2aWRpdHkgKyBwMV9zdGF0dXMgKyBibHRlc3Rfc3lwcmVzdWx0cyArIHBoX2Rpc2NoYXJnZSArIHBoX3NvcmVzX3VsY2VycyArIHdhdGVyICsgZGF0ZV9kaWZmICsgKDF8UElEKSwgZGF0YSA9IGEyLm1nW2EyLm1nJHRyZWF0bWVudCAlaW4lICJQckVQIiwgXSkKYW5vdmEodGhlLm1vZGVsMikKcHZhbHVlMjwtc3VtbWFyeSh0aGUubW9kZWwyKSRjb2VmZmljaWVudHNbLCAiUHIoPnx0fCkiXVsic3dhYjIiXQoKcHZhbHNfZGYgPC0gZGF0YS5mcmFtZSgKICB0cmVhdG1lbnQgPSBjKCJBUlQiLCAiUHJFUCIpLAogIHBfdmFsdWUgPSBjKCIqKiIsICIqKioiKQopCmBgYAoKIyMjIEZJRyA1OiBTaGFubm9uIGJ5IHN3YWIKYGBge3J9CiMjIEluZGl2aWR1YWxzIHdoaWNoIGluaXRpYXRlIEFSVCBhcmUgbGVzcyBsaWtlbHkgdG8gZXhwZXJpZW5jZSBhbiBpbmNyZWFzZSBpbiBhbHBoYS1kaXZlcnNpdHkgcmVsYXRpdmUgdG8gdGhvc2UgdGhhdCBpbml0aWFsIFByRVAuCmNtcDwtbGlzdChjKCJNMCIsICJNMSIpKQp0by5wbG90PC1hMi5tZwp0by5wbG90JHN3YWI8LWZhY3RvcihpZmVsc2UodG8ucGxvdCRzd2FiICVpbiUgIjEiLCAiTTAiLCAiTTEiKSkKCnNlZ21lbnRzX2RmIDwtIGRhdGEuZnJhbWUoCiAgdHJlYXRtZW50X2dyb3VwID0gYygiQVJUIiwgIlByRVAiKSwKICB4ID0gYygxLCAxKSwKICB4ZW5kID0gYygyLCAyKSwKICB5ID0gMywKICB5ZW5kID0gMwopCgpwMTwtZ2dwbG90KHRvLnBsb3RbdG8ucGxvdCR0cmVhdG1lbnQgJWluJSAiQVJUIiwgXSwgYWVzKHg9c3dhYiwgeT1TaGFubm9uKSkrCiAgZ2VvbV9ib3hwbG90KGNvbG9yPSJibGFjayIsIGx3ZD0wLjEsIG91dGxpZXIuc2hhcGUgPSBOQSwgbm90Y2ggPSBULCBhZXMoZmlsbD10cmVhdG1lbnQpKSsKICBnZW9tX3BvaW50KHBvc2l0aW9uPXBvc2l0aW9uX2ppdHRlcmRvZGdlKCksIHNpemU9MC4wNSwgYWVzKGZpbGw9dHJlYXRtZW50KSkrCiAgc2NhbGVfZmlsbF9tYW51YWwodmFsdWVzPWModHJlYXQuY29sc1sxXSksIGxhYmVsPWMoIkFSVCIsICJQckVQIiksIG5hbWU9IiIpKwogIHRoZW1lX2J3KCkrCiAgdGhlbWUobGVnZW5kLnBvc2l0aW9uID0gIm5vbmUiLCAKICAgICAgICB0ZXh0PWVsZW1lbnRfdGV4dChzaXplPTUsIGNvbG9yPSJibGFjayIpLCAKICAgICAgICBsZWdlbmQua2V5LnNpemUgPSB1bml0KDAuMywgImNtIiksIAogICAgICAgIGxpbmUgPSBlbGVtZW50X2xpbmUobGluZXdpZHRoID0gMC4xKSwgCiAgICAgICAgYXhpcy50aXRsZSA9IGVsZW1lbnRfdGV4dChzaXplPTUpLCAKICAgICAgICBheGlzLnRleHQgPSBlbGVtZW50X3RleHQoc2l6ZT02LCBjb2xvcj0iYmxhY2siKSkrCiAgeWxhYigiU2hhbm5vbiBEaXZlcnNpdHkiKSsKICB4bGFiKCIiKSsKICBnZW9tX3RleHQoZGF0YSA9IHB2YWxzX2RmWzEsXSwgYWVzKHggPSAxLjUsIHkgPSAzLjEsIGxhYmVsID0gcF92YWx1ZSwgaGp1c3QgPSAwLjUpKSsKICBnZW9tX3NlZ21lbnQoZGF0YSA9IHNlZ21lbnRzX2RmWzEsXSwgYWVzKHggPSB4LCB4ZW5kID0geGVuZCwgeSA9IHksIHllbmQgPSB5ZW5kKSwgbGluZXdpZHRoID0gMC4yNSkKCnAyPC1nZ3Bsb3QodG8ucGxvdFt0by5wbG90JHRyZWF0bWVudCAlaW4lICJQckVQIiwgXSwgYWVzKHg9c3dhYiwgeT1TaGFubm9uKSkrCiAgZ2VvbV9ib3hwbG90KGNvbG9yPSJibGFjayIsIGx3ZD0wLjEsIG91dGxpZXIuc2hhcGUgPSBOQSwgbm90Y2ggPSBULCBhZXMoZmlsbD10cmVhdG1lbnQpKSsKICBnZW9tX3BvaW50KHBvc2l0aW9uPXBvc2l0aW9uX2ppdHRlcmRvZGdlKCksIHNpemU9MC4wNSwgYWVzKGZpbGw9dHJlYXRtZW50KSkrCiAgc2NhbGVfZmlsbF9tYW51YWwodmFsdWVzPWModHJlYXQuY29sc1syXSksIGxhYmVsPWMoIkFSVCIsICJQckVQIiksIG5hbWU9IiIpKwogIHRoZW1lX2J3KCkrCiAgdGhlbWUobGVnZW5kLnBvc2l0aW9uID0gIm5vbmUiLCAKICAgICAgICB0ZXh0PWVsZW1lbnRfdGV4dChzaXplPTUsIGNvbG9yPSJibGFjayIpLCAKICAgICAgICBsZWdlbmQua2V5LnNpemUgPSB1bml0KDAuMywgImNtIiksIAogICAgICAgIGxpbmUgPSBlbGVtZW50X2xpbmUobGluZXdpZHRoID0gMC4xKSwgCiAgICAgICAgYXhpcy50aXRsZSA9IGVsZW1lbnRfdGV4dChzaXplPTUpLCAKICAgICAgICBheGlzLnRleHQgPSBlbGVtZW50X3RleHQoc2l6ZT02LCBjb2xvcj0iYmxhY2siKSkrCiAgeWxhYigiU2hhbm5vbiBEaXZlcnNpdHkiKSsKICB4bGFiKCIiKSsKICBnZW9tX3RleHQoZGF0YSA9IHB2YWxzX2RmWzIsXSwgYWVzKHggPSAxLjUsIHkgPSAzLjEsIGxhYmVsID0gcF92YWx1ZSwgaGp1c3QgPSAwLjUpKSsKICBnZW9tX3NlZ21lbnQoZGF0YSA9IHNlZ21lbnRzX2RmWzIsXSwgYWVzKHggPSB4LCB4ZW5kID0geGVuZCwgeSA9IHksIHllbmQgPSB5ZW5kKSwgbGluZXdpZHRoID0gMC4yNSkKCnRvcF9yb3cgPSBnZ2FycmFuZ2UocDEsIHAyLCBuY29sID0gMiwgbGFiZWxzID0gYygiQS4iLCAiQi4iKSwgZm9udC5sYWJlbCA9IGxpc3QoZmFjZT0icGxhaW4iLCBzaXplPTgpLCB3aWR0aHMgPSBjKDAuNSwgMC41KSkKdGlmZihwYXN0ZSgiTWFudXNjcmlwdF9GSUdVUkVTL0ZpZ3VyZV81XyIsIHRvZGF5MiwgIi50aWZmIiwgc2VwPSIiKSwgaGVpZ2h0ID0gMTgwMCwgd2lkdGg9MjQwMCwgcmVzPTYwMCkKdG9wX3JvdwpkZXYub2ZmKCkKYGBgCgojIyMgLSBDU1QgVHJhbnNpdGlvbiBhbmQgQVJWCmBgYHtyfQphMi5tZy5kZjwtcmVzaGFwZTI6OmRjYXN0KGEyLm1nLCBQSUQrdHJlYXRtZW50K2RhdGVfZGlmZn5zd2FiLCB2YWx1ZS52YXIgPSAiQ1NUIikKYTIubWcuZGYkYDFgPC1nc3ViKCJJVi1BIiwgIklWIiwgYTIubWcuZGYkYDFgKQphMi5tZy5kZiRgMWA8LWdzdWIoIklWLUIiLCAiSVYiLCBhMi5tZy5kZiRgMWApCmEyLm1nLmRmJGAxYDwtZ3N1YigiSVYtQyIsICJJViIsIGEyLm1nLmRmJGAxYCkKYTIubWcuZGYkYDJgPC1nc3ViKCJJVi1BIiwgIklWIiwgYTIubWcuZGYkYDJgKQphMi5tZy5kZiRgMmA8LWdzdWIoIklWLUIiLCAiSVYiLCBhMi5tZy5kZiRgMmApCmEyLm1nLmRmJGAyYDwtZ3N1YigiSVYtQyIsICJJViIsIGEyLm1nLmRmJGAyYCkKYTIubWcuZGYkdHJhbnNpdGlvbjwtZmFjdG9yKGlmZWxzZShhMi5tZy5kZiRgMWAgPT0gYTIubWcuZGYkYDJgLCAwLCAxKSwgbGV2ZWxzPWMoMCwgMSkpCmEyLm1nLmRmJHRyYW5zaXRpb25fZGV0PC1mYWN0b3IoaWZlbHNlKGEyLm1nLmRmJHRyYW5zaXRpb24gPT0gMCwgIk5vIENoYW5nZSIsIHBhc3RlKGEyLm1nLmRmJGAxYCwgIuKGkyIsIGEyLm1nLmRmJGAyYCwgc2VwPSJcbiIpKSwgbGV2ZWxzPWMoIk5vIENoYW5nZSIsICJJXG7ihpNcbklJSSIsICJJXG7ihpNcbklWIiwgIklJSVxu4oaTXG5JIiwgIklJSVxu4oaTXG5JViIsICJJSUlcbuKGk1xuViIsICJJVlxu4oaTXG5JIiwgIklWXG7ihpNcbklJSSIsICJWXG7ihpNcbklWIikpCgojIyBNZWFuIGRpZmYgaW4gc2hhbm5vbiBhZnRlciBBUlYKc2hhbm5vbjwtcmVzaGFwZTI6OmRjYXN0KGEyLm1nLCBQSUR+c3dhYiwgdmFsdWUudmFyID0gIlNoYW5ub24iKQpzaGFubm9uJHNoYW5ub25fZGlmZjwtc2hhbm5vbiRgMmAtc2hhbm5vbiRgMWAKbmFtZXMoc2hhbm5vbilbMjozXTwtYygiQmFzZWxpbmVfc2hhbm5vbiIsICJBZnRlckFSVl9zaGFubm9uIikKYTIubWcuZGYkU2hhbm5vbjwtTlVMTAphMi5tZy5kZjwtbWVyZ2UoYTIubWcuZGYsIHNoYW5ub24sIGFsbD1UUlVFKQphMi5tZy5kZiAlPiUgZ3JvdXBfYnkodHJlYXRtZW50KSAlPiUgc3VtbWFyaXNlKG1lYW5TaGFubm9uZGlmZj1tZWFuKHNoYW5ub25fZGlmZikpCgojIyBBZGQgaW4gY292YXJpYXRlcyBhdCBlbnJvbGxtZW50CmEyLm1nLmRmPC1tZXJnZShhMi5tZy5kZiwgYTIubWdbYTIubWckc3dhYiAlaW4lICIxIiwgXSwgYWxsLng9VFJVRSkKCnRoZS5tb2RlbDwtZ2xtKHRyYW5zaXRpb25+dHJlYXRtZW50ICsgc2RfYWdlICsgcGhfZWdhICsgZ3JhdmlkaXR5ICsgcDFfc3RhdHVzICsgYmx0ZXN0X3N5cHJlc3VsdHMgKyBwaF9kaXNjaGFyZ2UgKyBwaF9zb3Jlc191bGNlcnMgKyB3YXRlciArIGRhdGVfZGlmZiwgZGF0YSA9IGEyLm1nLmRmLCBmYW1pbHkgPSAiYmlub21pYWwiKQpjPC1hcy5kYXRhLmZyYW1lKGJyb29tOjp0aWR5KHRoZS5tb2RlbCwgY29uZi5pbnQgPSBULCBleHBvbmVudGlhdGUgPSBUKSkKIyBwZW9wbGUgdGhhdCBjaGFuZ2UgY3N0cyBhcmUgbGVzcyBsaWtlbHkgdG8gaGF2ZSBzeXBoIGF0IGVucm9sbG1lbnQgYW5kIGxlc3MgbGlrZWx5IHRvIGhhdmUgd2F0ZXIKYGBgCgojIyMgRklHIDY6IFBSSU5UIApgYGB7cn0KdG8ucGxvdDwtYTIubWcuZGYgJT4lIGdyb3VwX2J5KHRyZWF0bWVudCwgdHJhbnNpdGlvbl9kZXQpICU+JSBzdW1tYXJpc2Uobj1sZW5ndGgodW5pcXVlKFBJRCkpKQp0by5wbG90W3RvLnBsb3QkdHJlYXRtZW50ICVpbiUgIkFSVCIsICJwcm9wIl08LXRvLnBsb3RbdG8ucGxvdCR0cmVhdG1lbnQgJWluJSAiQVJUIiwgIm4iXS80OQp0by5wbG90W3RvLnBsb3QkdHJlYXRtZW50ICVpbiUgIlByRVAiLCAicHJvcCJdPC10by5wbG90W3RvLnBsb3QkdHJlYXRtZW50ICVpbiUgIlByRVAiLCAibiJdLzEzMgoKbGV2ZWxzKHRvLnBsb3QkdHJlYXRtZW50KSA8LSBjKCJBUlQsIG4gPSAzOSIsICJQckVQLCBuID0gODQiKQpwMTwtZ2dwbG90KHRvLnBsb3RbdG8ucGxvdCR0cmFuc2l0aW9uX2RldCAlaW4lICJObyBDaGFuZ2UiLCBdLCBhZXMoeD10cmVhdG1lbnQsIHk9cHJvcCwgZmlsbD10cmVhdG1lbnQpKSsKICBnZW9tX2JhcihzdGF0PSJpZGVudGl0eSIsIHBvc2l0aW9uPSJkb2RnZSIsIHdpZHRoPTAuNywgY29sb3I9ImJsYWNrIiwgbHdkPTAuMSkrCiAgc2NhbGVfZmlsbF9tYW51YWwodmFsdWVzPXRyZWF0LmNvbHMsIG5hbWU9IiIpKwogIHRoZW1lX2J3KCkrCiAgdGhlbWUobGVnZW5kLnBvc2l0aW9uID0gIm5vbmUiLCAKICAgICAgICB0ZXh0PWVsZW1lbnRfdGV4dChzaXplPTUsIGNvbG9yPSJibGFjayIpLCAKICAgICAgICBsZWdlbmQua2V5LnNpemUgPSB1bml0KDAuMywgImNtIiksIAogICAgICAgIGxpbmUgPSBlbGVtZW50X2xpbmUobGluZXdpZHRoID0gMC4xKSwgCiAgICAgICAgYXhpcy50aXRsZSA9IGVsZW1lbnRfdGV4dChzaXplPTUpLCAKICAgICAgICBheGlzLnRleHQgPSBlbGVtZW50X3RleHQoY29sb3I9ImJsYWNrIikpKwogIHlsYWIoIlByb3BvcnRpb24gb2YgU2FtcGxlcyBpbiBBUlYgR3JvdXAiKSsKICB4bGFiKCIiKQoKbGV2ZWxzKHRvLnBsb3QkdHJlYXRtZW50KSA8LSBjKCJBUlQsIG4gPSAxMCIsICJQckVQLCBuID0gNDgiKQpwMjwtZ2dwbG90KHRvLnBsb3RbIXRvLnBsb3QkdHJhbnNpdGlvbl9kZXQgJWluJSAiTm8gQ2hhbmdlIiwgXSwgYWVzKHg9dHJhbnNpdGlvbl9kZXQsIHk9cHJvcCwgZmlsbD10cmVhdG1lbnQsIGdyb3VwPXRyZWF0bWVudCkpKwogIGdlb21fYmFyKHN0YXQ9ImlkZW50aXR5IiwgcG9zaXRpb249ImRvZGdlIiwgd2lkdGg9MC43LCBjb2xvcj0iYmxhY2siLCBsd2Q9MC4xKSsKICBzY2FsZV9maWxsX21hbnVhbCh2YWx1ZXM9dHJlYXQuY29scywgbmFtZT0iIikrCiAgdGhlbWVfYncoKSsKICB0aGVtZShsZWdlbmQucG9zaXRpb24gPSAibm9uZSIsIAogICAgICAgIHRleHQ9ZWxlbWVudF90ZXh0KHNpemU9NSwgY29sb3I9ImJsYWNrIiksIAogICAgICAgIGxlZ2VuZC5rZXkuc2l6ZSA9IHVuaXQoMC4zLCAiY20iKSwgCiAgICAgICAgbGluZSA9IGVsZW1lbnRfbGluZShsaW5ld2lkdGggPSAwLjEpLCAKICAgICAgICBheGlzLnRpdGxlID0gZWxlbWVudF90ZXh0KHNpemU9NSksIAogICAgICAgIGF4aXMudGV4dCA9IGVsZW1lbnRfdGV4dChjb2xvcj0iYmxhY2siKSkrCiAgeWxhYigiUHJvcG9ydGlvbiBvZiBTYW1wbGVzIGluIEFSViBHcm91cCIpKwogIHhsYWIoIiIpKwogIGZhY2V0X3dyYXAofnRyZWF0bWVudCwgbnJvdyA9IDIpCiNnZ3NhdmUocGFzdGUoIkFuYWx5c2lzX0ZpZ3VyZXMvQWltMl90cmVhdG1lbnRfcHJldmFsZW5jZV9ieV9jc3RfdHJhbnNpdGlvbl90eXBlXyIsIHRvZGF5MiwgIi5wbmciLCBzZXA9IiIpLCBoZWlnaHQgPSAzLCB3aWR0aD0zLCBkcGkgPSAxMjAwKQoKIyMjIyMgRklHIDY6IFBSSU5UCnRvcF9yb3cgPSBnZ2FycmFuZ2UocDEsIHAyLCBuY29sID0gMiwgbGFiZWxzID0gYygiQS4iLCAiQi4iKSwgZm9udC5sYWJlbCA9IGxpc3QoZmFjZT0icGxhaW4iLCBzaXplPTgpKQp0aWZmKHBhc3RlKCJNYW51c2NyaXB0X0ZJR1VSRVMvRmlndXJlXzZfIiwgdG9kYXkyLCAiLnRpZmYiLCBzZXA9IiIpLCBoZWlnaHQgPSAxODAwLCB3aWR0aD0yNDAwLCByZXM9NjAwKQp0b3Bfcm93CmRldi5vZmYoKQpgYGAKCiMjIFRheGEgc3BlY2lmaWMgYW5hbHlzaXMsIGJlZm9yZSB2cy4gYWZ0ZXIgYnkgdHJlYXRtZW50CmBgYHtyLCB3YXJuaW5nPUZBTFNFfQpjb3VudHM8LWRjYXN0KGNvdW50cy5tLnRlbXBbY291bnRzLm0udGVtcCRtYXJ5bGFuZF9JRCAlaW4lIHNhbXBsZS5saXN0LCBdLCBmb3JtdWxhID0gbWFyeWxhbmRfSUR+dGF4YSwgdmFsdWUudmFyID0gImNvdW50cyIsIGZ1bi5hZ2dyZWdhdGUgPSBzdW0pCgojIyBSZW1vdmUgYWxsIHRheGEgZm91bmQgaW4genltbyBwb3NpdGl2ZSBjb250cm9scwptZ3NzMDwtbmFtZXMoY291bnRzKVtncmVwbCgiMCQiLCBuYW1lcyhjb3VudHMpKV0KY291bnRzPC1jb3VudHNbLCFuYW1lcyhjb3VudHMpICVpbiUgYygiRW50ZXJvY29jY3VzX2ZhZWNhbGlzIiwgIkVzY2hlcmljaGlhX2NvbGkiLCAiTGFjdG9iYWNpbGx1c19mZXJtZW50dW0iLCAiU2FsbW9uZWxsYV9lbnRlcmljYSIsICJTdGFwaHlsb2NvY2N1c19hdXJldXMiLCAiQ2FuZGlkYXR1c19QZWxhZ2liYWN0ZXIiLCBtZ3NzMCwgdG8ucm0pXQoKcm93bmFtZXMoY291bnRzKTwtY291bnRzJG1hcnlsYW5kX0lECmNvdW50cyRtYXJ5bGFuZF9JRDwtTlVMTApjb3VudHMuYWxsPC1jb3VudHMKCnRheGEuYWxsLnJlc3VsdHMubG9uZzwtZGF0YS5mcmFtZSgpCmZvcihpIGluIGMoIkFSVCIsICJQckVQIikpewogIHBpZDwtYTIubWcuZGZbYTIubWcuZGYkdHJlYXRtZW50ICVpbiUgaSAsICJQSUQiXQogIHNhbXBsZXM8LWEyLm1nW2EyLm1nJFBJRCAlaW4lIHBpZCwgIm1hcnlsYW5kX0lEIl0KICBjb3VudHM8LWNvdW50cy5hbGxbcm93bmFtZXMoY291bnRzLmFsbCkgJWluJSBzYW1wbGVzLCBdCiAgbmFtZXMoY291bnRzKTwtZ3N1YigiX1xcLiIsICIgIiwgbmFtZXMoY291bnRzKSkKICBuYW1lcyhjb3VudHMpPC1nc3ViKCJfIiwgIiAiLCBuYW1lcyhjb3VudHMpKQogIG5hbWVzKGNvdW50cyk8LWdzdWIocGF0dGVybiA9ICJcXC5cXC4iLCByZXBsYWNlbWVudCA9ICIgIiwgbmFtZXMoY291bnRzKSkKICBuYW1lcyhjb3VudHMpPC1nc3ViKHBhdHRlcm4gPSAiXFwuIiwgcmVwbGFjZW1lbnQgPSAiICIsIG5hbWVzKGNvdW50cykpCiAgY291bnRzJG1hcnlsYW5kX0lEPC1yb3duYW1lcyhjb3VudHMpCgogIGQubTwtcmVzaGFwZTI6Om1lbHQoY291bnRzLCBpZC52YXJzID0gIm1hcnlsYW5kX0lEIiwgdmFyaWFibGUubmFtZSA9ICJUYXhvbiIsIHZhbHVlLm5hbWUgPSAiY291bnQiLCBuYS5ybSA9IFQpCiAgZC5tPC1tZXJnZShkLm0sIGEyLm1nWyxjKCJtYXJ5bGFuZF9JRCIsICJzd2FiIildLCBhbGwueD1UUlVFKQogIGQubSRzd2FiPC1mYWN0b3IoaWZlbHNlKGQubSRzd2FiICVpbiUgIjEiLCAiTTAiLCAiTTEiKSkKICBzYW1wbGVzLngudGF4b248LWQubVtkLm0kY291bnQgPiAwLCBdICU+JSBkcGx5cjo6Z3JvdXBfYnkoVGF4b24sIHN3YWIpICU+JSBkcGx5cjo6c3VtbWFyaXNlKG5TYW1wbGVzPWxlbmd0aCh1bmlxdWUobWFyeWxhbmRfSUQpKSkKCiAgc2FtcGxlcy54LnRheG9uLmRmPC1yZXNoYXBlMjo6ZGNhc3Qoc2FtcGxlcy54LnRheG9uLCBUYXhvbn5zd2FiLCB2YWx1ZS52YXIgPSAiblNhbXBsZXMiKQogIHNhbXBsZXMueC50YXhvbi5kZiRgTkFgPC1OVUxMCiAgc2FtcGxlcy54LnRheG9uLmRmW2lzLm5hKHNhbXBsZXMueC50YXhvbi5kZildPC0wCiAgc2FtcGxlcy54LnRheG9uLmRmJFRvdGFsPC1zYW1wbGVzLngudGF4b24uZGYkYE0wYCtzYW1wbGVzLngudGF4b24uZGYkYE0xYAoKICAjIyBUZXN0IG9ubHkgdGhvc2UgdGF4YSB3aGljaCBhcmUgcHJlc2VudCBpbiBhdCBsZWFzdCA1JSBvZiBzYW1wbGVzID0gIG1pbi4gOCBzYW1wbGVzID0gMTA3IHRheGEKICBrZWVwPC1hcy52ZWN0b3Ioc2FtcGxlcy54LnRheG9uLmRmW3NhbXBsZXMueC50YXhvbi5kZiRUb3RhbCA+PSAwLjA1Km5yb3coY291bnRzKSwgIlRheG9uIl0pCiAgYzwtcmVzaGFwZTI6OmRjYXN0KGQubVtkLm0kVGF4b24gJWluJSBrZWVwLCBdLCBUYXhvbn5tYXJ5bGFuZF9JRCwgdmFsdWUudmFyID0gImNvdW50IiwgZnVuLmFnZ3JlZ2F0ZSA9IHN1bSkKICByb3duYW1lcyhjKTwtYyRUYXhvbgogIGM8LWNbLTFdCiAgYzwtY1ssIGNvbFN1bXMoYykgPiAwXQogIGM8LWNbcm93U3VtcyhjKSA+IDAsICBdCgogIG1ldGEuc3ltPC1hMi5tZ1ssYygibWFyeWxhbmRfSUQiLCAic3dhYiIsIGNvdmFyLCAiZGF0ZV9kaWZmIildCiAgbWV0YS5zeW0kc3dhYjwtZmFjdG9yKGlmZWxzZShtZXRhLnN5bSRzd2FiICVpbiUgIjEiLCAiTTAiLCAiTTEiKSkKICBtZXRhLnN5bSRjdXJyX3B0bnJzPC1OVUxMCiAgbWV0YS5zeW0kc3dhYjwtZmFjdG9yKG1ldGEuc3ltJHN3YWIpCiAgbWV0YS5zeW0kc3dhYjwtcmVsZXZlbChtZXRhLnN5bSRzd2FiLCByZWY9Ik0wIikKICBtZXRhLnN5bTwtbWV0YS5zeW1bbWV0YS5zeW0kbWFyeWxhbmRfSUQgJWluJSBuYW1lcyhjKSwgXQogIG1ldGEuc3ltPC1tZXRhLnN5bVtjb21wbGV0ZS5jYXNlcyhtZXRhLnN5bSksIF0KICBjPC1jWyxjb2xuYW1lcyhjKSAlaW4lIG1ldGEuc3ltJG1hcnlsYW5kX0lEXQogIAogIG9iai5zeW08LURFU2VxRGF0YVNldEZyb21NYXRyaXgoY291bnREYXRhID0gcm91bmQoYyksIGNvbERhdGEgPSBtZXRhLnN5bSwgZGVzaWduPX5zZF9hZ2UgKyBwaF9lZ2EgKyBncmF2aWRpdHkgKyBwMV9zdGF0dXMgKyBibHRlc3Rfc3lwcmVzdWx0cyArIHBoX2Rpc2NoYXJnZSArIHBoX3NvcmVzX3VsY2VycyArIHdhdGVyICsgZGF0ZV9kaWZmICsgc3dhYikKCiAgb2JqLnN5bSA9IGVzdGltYXRlU2l6ZUZhY3RvcnMoIG9iai5zeW0sIHR5cGU9InBvc2NvdW50cyIgKQogIG9iai5zeW0gPSBlc3RpbWF0ZURpc3BlcnNpb25zKCBvYmouc3ltLCBmaXRUeXBlPSJsb2NhbCIgKQoKICAjIyBUcnkgbGltbWEgKyB2b29tCiAgc3dhYjwtbWFrZS5uYW1lcyhtZXRhLnN5bSRzd2FiKQogIHNkX2FnZTwtbWV0YS5zeW0kc2RfYWdlCiAgcGhfZWdhPC1tZXRhLnN5bSRwaF9lZ2EKICBkYXRlX2RpZmY8LW1ldGEuc3ltJGRhdGVfZGlmZgogIGdyYXZpZGl0eTwtbWFrZS5uYW1lcyhtZXRhLnN5bSRncmF2aWRpdHkpCiAgcDFfc3RhdHVzPC1tYWtlLm5hbWVzKG1ldGEuc3ltJHAxX3N0YXR1cykKICBibHRlc3Rfc3lwcmVzdWx0czwtbWFrZS5uYW1lcyhtZXRhLnN5bSRibHRlc3Rfc3lwcmVzdWx0cykKICBwaF9kaXNjaGFyZ2U8LW1ha2UubmFtZXMobWV0YS5zeW0kcGhfZGlzY2hhcmdlKQogIHBoX3NvcmVzX3VsY2VyczwtbWFrZS5uYW1lcyhtZXRhLnN5bSRwaF9zb3Jlc191bGNlcnMpCiAgd2F0ZXI8LW1ha2UubmFtZXMobWV0YS5zeW0kd2F0ZXIpCiAgCiAgZGVzaWduPC1tb2RlbC5tYXRyaXgofnNkX2FnZSArIHBoX2VnYSArIGdyYXZpZGl0eSArIHAxX3N0YXR1cyArIGJsdGVzdF9zeXByZXN1bHRzICsgcGhfZGlzY2hhcmdlICsgcGhfc29yZXNfdWxjZXJzICsgd2F0ZXIgKyBkYXRlX2RpZmYgKyBzd2FiKQogIAogIG5jIDwtIGNvdW50cyhvYmouc3ltLCBub3JtYWxpemVkPVRSVUUpCiAgUElEPC1hMi5tZ1thMi5tZyRtYXJ5bGFuZF9JRCAlaW4lIGNvbG5hbWVzKG5jKSwgYygiUElEIildCiAgZDAgPC0gREdFTGlzdChuYykKICBkMCA8LSBjYWxjTm9ybUZhY3RvcnMoZDApCiAgdjwtdm9vbShkMCwgZGVzaWduLCBibG9jaz1QSUQsIHBsb3Q9VCkKICBjb3JmaXQ8LWR1cGxpY2F0ZUNvcnJlbGF0aW9uKHYsIGRlc2lnbiwgYmxvY2s9UElEKQogIGZpdDwtbG1GaXQodiwgZGVzaWduLCBibG9jaz1QSUQsIGNvcnJlbGF0aW9uID0gY29yZml0JGNvbnNlbnN1cy5jb3JyZWxhdGlvbikKICB0bXAgPC0gZUJheWVzKGZpdCkKICB0b3AudGFibGUgPC0gdG9wVGFibGUodG1wLCBzb3J0LmJ5ID0gIkYiLCBuID0gSW5mKQogIHRvcC50YWJsZSR0YXhhPC1yb3duYW1lcyh0b3AudGFibGUpCiAgdG9wLnRhYmxlJHRyZWF0bWVudDwtaQogIHRheGEuYWxsLnJlc3VsdHMubG9uZzwtcmJpbmQodGF4YS5hbGwucmVzdWx0cy5sb25nLCB0b3AudGFibGUpCn0KCnRheGEuYWxsLnJlc3VsdHMubG9uZzwtdW5pcXVlKHRheGEuYWxsLnJlc3VsdHMubG9uZykKdGF4YS5hbGwucmVzdWx0cy5sb25nJGFzc29jaWF0aW9uPC1pZmVsc2UodGF4YS5hbGwucmVzdWx0cy5sb25nJHN3YWJNMSA8IDAsICJOZWdhdGl2ZSIsICJQb3NpdGl2ZSIpCm9yZGVyLnRheDwtdGF4YS5hbGwucmVzdWx0cy5sb25nW29yZGVyKHRheGEuYWxsLnJlc3VsdHMubG9uZyRzd2FiTTEsIGRlY3JlYXNpbmcgPSBUKSwgInRheGEiXQp0YXhhLmFsbC5yZXN1bHRzLmxvbmckdGF4YTwtZmFjdG9yKHRheGEuYWxsLnJlc3VsdHMubG9uZyR0YXhhKQoKdGF4YS5hbGwucmVzdWx0cy5sb25nJHRyZWF0bWVudDwtZmFjdG9yKHRheGEuYWxsLnJlc3VsdHMubG9uZyR0cmVhdG1lbnQsIGxldmVscz1jKCJBUlQiLCAiUHJFUCIpKQpgYGAKIyMjIEZJRyA3OiBQckVQIC8gQVJUIFRheGEKYGBge3J9CiMjIFRoZSByZWZlcmVuY2Ugd2FzIGJlZm9yZSwgc28gd2hpdGUgaXMgaW5jcmVhc2VkIGFmdGVyIGFuZCBibGFjayBpcyBkZWNyZWFzZSBhZnRlciBBUlYgaW5pdGlhdGlvbi4gCnAyPC1nZ3Bsb3QodGF4YS5hbGwucmVzdWx0cy5sb25nW3RheGEuYWxsLnJlc3VsdHMubG9uZyRhZGouUC5WYWwgPCAwLjA1ICYgIWlzLm5hKHRheGEuYWxsLnJlc3VsdHMubG9uZyRhZGouUC5WYWwpICYgdGF4YS5hbGwucmVzdWx0cy5sb25nJHRyZWF0bWVudCAlaW4lICJQckVQIiwgXSwgYWVzKHg9c3dhYk0xLCB5PXJlb3JkZXIodGF4YSwgc3dhYk0xKSwgZmlsbD1hc3NvY2lhdGlvbikpKwogIGdlb21fYmFyKHN0YXQ9ImlkZW50aXR5IiwgY29sb3I9ImJsYWNrIiwgbHdkPTAuMSkrCiAgeGxhYigiTG9nLUZvbGQgRGlmZmVyZW5jZSBpbiBtZ1NzIEFidW5kYW5jZVxuKE0xIC8gTTApIikrCiAgc2NhbGVfZmlsbF9tYW51YWwodmFsdWVzPWMoImJsYWNrIiwgIndoaXRlIiksIG5hbWU9IlRheGEgQXNzb2NpYXRlZCB3aXRoIFRyZWF0bWVudCBJbml0aWF0aW9uIikrCiAgdGhlbWVfYncoKSsKICB0aGVtZShheGlzLnRpdGxlLnggPSBlbGVtZW50X3RleHQoc2l6ZT01LCBjb2xvcj0iYmxhY2siKSwgCiAgICAgICAgYXhpcy50ZXh0LnkgPSBlbGVtZW50X3RleHQoc2l6ZT0yLjUsIGZhY2U9Iml0YWxpYyIsIGNvbG9yPSJibGFjayIpLCAKICAgICAgICBheGlzLnRleHQueD1lbGVtZW50X3RleHQoc2l6ZT01LCBjb2xvcj0iYmxhY2siKSwgCiAgICAgICAgYXhpcy50aWNrcyA9IGVsZW1lbnRfbGluZShsaW5ld2lkdGggPSAwLjIpLAogICAgICAgIHN0cmlwLmJhY2tncm91bmQgPSBlbGVtZW50X3JlY3QoZmlsbD10cmVhdC5jb2xzWzJdKSwgCiAgICAgICAgc3RyaXAudGV4dCA9IGVsZW1lbnRfdGV4dChjb2xvcj0id2hpdGUiLCBmYWNlPSJib2xkIiwgc2l6ZT01KSwgCiAgICAgICAgbGVnZW5kLnBvc2l0aW9uID0gIm5vbmUiKSsKICB5bGFiKCIiKSsKICBmYWNldF93cmFwKH50cmVhdG1lbnQpIAojZ2dzYXZlKHBhc3RlKCJBbmFseXNpc19GaWd1cmVzL01MQVdJX21nU3NfdHJlYXRtZW50X2NoYW5nZV9QckVQXyIsdG9kYXkyLCAiLnRpZmYiLCBzZXA9IiIpLCBoZWlnaHQ9NCwgd2lkdGg9NCwgZHBpID0gNjAwKQoKcDE8LWdncGxvdCh0YXhhLmFsbC5yZXN1bHRzLmxvbmdbdGF4YS5hbGwucmVzdWx0cy5sb25nJGFkai5QLlZhbCA8IDAuMDUgJiAhaXMubmEodGF4YS5hbGwucmVzdWx0cy5sb25nJGFkai5QLlZhbCkgJiB0YXhhLmFsbC5yZXN1bHRzLmxvbmckdHJlYXRtZW50ICVpbiUgIkFSVCIsIF0sIGFlcyh4PXN3YWJNMSwgeT1yZW9yZGVyKHRheGEsIHN3YWJNMSksIGZpbGw9YXNzb2NpYXRpb24pKSsKICBnZW9tX2JhcihzdGF0PSJpZGVudGl0eSIsIGNvbG9yPSJibGFjayIsIGx3ZD0wLjEpKwogIHhsYWIoIkxvZy1Gb2xkIERpZmZlcmVuY2UgaW4gbWdTcyBBYnVuZGFuY2VcbihNMSAvIE0wKSIpKwogIHNjYWxlX2ZpbGxfbWFudWFsKHZhbHVlcz1jKCJibGFjayIsICJ3aGl0ZSIpLCBuYW1lPSJUYXhhIEFzc29jaWF0ZWQgd2l0aCBUcmVhdG1lbnQgSW5pdGlhdGlvbiIpKwogIHRoZW1lX2J3KCkrCiAgdGhlbWUoYXhpcy50aXRsZS54ID0gZWxlbWVudF90ZXh0KHNpemU9NSwgY29sb3I9ImJsYWNrIiksIAogICAgICAgIGF4aXMudGV4dC55ID0gZWxlbWVudF90ZXh0KHNpemU9MywgZmFjZT0iaXRhbGljIiwgY29sb3I9ImJsYWNrIiksIAogICAgICAgIGF4aXMudGV4dC54PWVsZW1lbnRfdGV4dChzaXplPTUsIGNvbG9yPSJibGFjayIpLAogICAgICAgIGF4aXMudGlja3MgPSBlbGVtZW50X2xpbmUobGluZXdpZHRoID0gMC4yKSwKICAgICAgICBzdHJpcC5iYWNrZ3JvdW5kID0gZWxlbWVudF9yZWN0KGZpbGw9dHJlYXQuY29sc1sxXSksIAogICAgICAgIHN0cmlwLnRleHQgPSBlbGVtZW50X3RleHQoY29sb3I9ImJsYWNrIiwgZmFjZT0iYm9sZCIsIHNpemU9NSksIAogICAgICAgIGxlZ2VuZC5wb3NpdGlvbiA9ICJub25lIikrCiAgeWxhYigiIikrCiAgZmFjZXRfd3JhcCh+dHJlYXRtZW50KQojZ2dzYXZlKHBhc3RlKCJBbmFseXNpc19GaWd1cmVzL01MQVdJX21nU3NfdHJlYXRtZW50X2NoYW5nZV9BUlRfIix0b2RheTIsICIudGlmZiIsIHNlcD0iIiksIGhlaWdodD00LCB3aWR0aD00LCBkcGkgPSA2MDApCnRvcF9yb3cgPSBnZ2FycmFuZ2UocDEsIHAyLCBuY29sID0gMiwgbGFiZWxzID0gYygiQS4iLCAiQi4iKSwgZm9udC5sYWJlbCA9IGxpc3QoZmFjZT0icGxhaW4iLCBzaXplPTgpLCB3aWR0aHMgPSBjKDAuNSwwLjUpKQp0aWZmKHBhc3RlKCJNYW51c2NyaXB0X0ZJR1VSRVMvRmlndXJlXzdfIiwgdG9kYXkyLCAiLnRpZmYiLCBzZXA9IiIpLCBoZWlnaHQgPSAxODAwLCB3aWR0aD0zNjAwLCByZXM9NjAwKQp0b3Bfcm93CmRldi5vZmYoKQpgYGAKIyMgQ29tcGFyaXNvbiBjb2hvcnQgKFpBUFBTKQpTdWJzZXQgdGhlIFpBUFBzIGRhdGEgdG8gaW5jbHVkZSB0aGUgYmVsb3cgaW5mb3JtYXRpb24gKHNlcGFyYXRlbHkgZm9yIEhJVi0gYW5kIFdMSElWKQpHZXN0YXRpb25hbCBBZ2UgYXQgRW5yb2xsbWVudCAod2Vla3MpCiAgICAgICAgV0xISVYgICAgICAgSElWKC0pCjYtMjAgICAgMTkgKDM4LjglKQk1MyAoNDAuMiUpCjIxLTMwICAgMjcgKDU1LjElKQk2MiAoNDcuMCUpCjMxLTQxICAgMyAoNi4xJSkJMTcgKDEyLjklKQoKR2VzdGF0aW9uYWwgQWdlIGF0IEVucm9sbG1lbnQgKHdlZWtzKQogICAgICAgICAgICAgICAgICAgICAgV0xISVYgICAgICAgICAgICAgSElWKC0pCk1lYW4gKFNEKQkgICAgICAgIDIyLjMgKDUuNTkpCSAgICAgIDIyLjggKDcuNDgpCTAuNjA2Ck1lZGlhbiBbTWluLCBNYXhdCTIyLjAgWzEwLjAsIDM2LjBdCTIzLjAgWzkuMDAsIDQxLjBdCQoKRGF5cyBiZXR3ZWVuIEFSViBpbml0aWF0aW9uIGFuZCAybmQgc2FtcGxlCiAgICAgICAgICAgICAgICAgICAgICAgIFdMSElWICAgICAgICAgICBISVYoLSkKTWVhbiAoU0QpICAgICAgICAgICAyOC4yICgyLjg1KQkgICAgICAgMjkuNiAoNS4zNikKTWVkaWFuIFtNaW4sIE1heF0gICAyOC4wIFsxOS4wLCA0MC4wXQkgMjkuMCBbMTguMCwgNzMuMF0KCkFnZSBhdCBFbnJvbGxtZW50ICh5ZWFycykKICAgICAgICBXTEhJViAgICAgICBISVYoLSkKMTgtMjQgICAxNyAoMzQuNyUpCTY1ICg0OS4yJSkJCjI1LTMwICAgMjEgKDQyLjklKQkzOSAoMjkuNSUpCQozMS00MiAgIDExICgyMi40JSkJMjggKDIxLjIlKQkKCiMjIyMjIFpBUFBTOiBQaWNrIHNhbXBsZXMKYGBge3J9CnphcHBzPC1yZWFkX2V4Y2VsKCJaQVBQUy9Db21iaW5lZF9aQVBQU19QcmVTU01hdF9NZXRhZGF0YV9GSU5BTF8xMTE2MjAyMV9jbGVhbi54bHN4Iiwgc2hlZXQgPSAiWkFQUFNfUHJlU1NNYXQiKQp6YXBwczwtdW5pcXVlKHphcHBzKQpjc3RzPC1yZWFkLmNzdigiWkFQUFMvMjAyMl9HQVRFU19hbGxfcnVuc19kYWRhMl9hYnVuZGFuY2VfdGFibGVfU3RSX0NTVC5jc3YiKQpjc3RzJHNhbXBsZUlEMjwtZ3N1YigiXFwuIiwgIi0iLCBjc3RzJHNhbXBsZUlEKQp6YXBwcyRzdWJDU1Q8LWNzdHNbbWF0Y2goemFwcHMkc3BlY2ltZW5pZCwgY3N0cyRzYW1wbGVJRDIpLCAic2ltX3N1YkNTVCJdCnphcHBzLmNhc3QuMTwtcmVzaGFwZTI6OmRjYXN0KHVuaXF1ZSh6YXBwc1shaXMubmEoemFwcHMkc3ViQ1NUKSwgYygicGFydGljaXBhbnRfaWQiLCAiVmlzaXQiLCAic3ViQ1NUIildKSwgcGFydGljaXBhbnRfaWR+VmlzaXQsIHZhbHVlLnZhciA9ICJzdWJDU1QiKQpuYW1lcyh6YXBwcy5jYXN0LjEpWzI6NF08LXBhc3RlKCJDU1RfIixuYW1lcyh6YXBwcy5jYXN0LjEpWzI6NF0sIHNlcD0iIikKemFwcHMuY2FzdC4yPC1yZXNoYXBlMjo6ZGNhc3QodW5pcXVlKHphcHBzWyFpcy5uYSh6YXBwcyRzdWJDU1QpLCBjKCJwYXJ0aWNpcGFudF9pZCIsICJWaXNpdCIsICJzdWJDU1QiLCAiZWdhX2RheXMiKV0pLCBwYXJ0aWNpcGFudF9pZH5WaXNpdCwgdmFsdWUudmFyID0gImVnYV9kYXlzIikKbmFtZXMoemFwcHMuY2FzdC4yKVsyOjRdPC1wYXN0ZSgiZWdhX2RheXNfIixuYW1lcyh6YXBwcy5jYXN0LjIpWzI6NF0sIHNlcD0iIikKemFwcHMuY2FzdC4zPC1yZXNoYXBlMjo6ZGNhc3QodW5pcXVlKHphcHBzWyFpcy5uYSh6YXBwcyRzdWJDU1QpLCBjKCJwYXJ0aWNpcGFudF9pZCIsICJWaXNpdCIsICJzdWJDU1QiLCAiZWdhX3dlZWtzIildKSwgcGFydGljaXBhbnRfaWR+VmlzaXQsIHZhbHVlLnZhciA9ICJlZ2Ffd2Vla3MiKQpuYW1lcyh6YXBwcy5jYXN0LjMpWzI6NF08LXBhc3RlKCJlZ2Ffd2Vla3NfIixuYW1lcyh6YXBwcy5jYXN0LjMpWzI6NF0sIHNlcD0iIikKemFwcHMuY2FzdC40PC1yZXNoYXBlMjo6ZGNhc3QodW5pcXVlKHphcHBzWyFpcy5uYSh6YXBwcyRzdWJDU1QpLCBjKCJwYXJ0aWNpcGFudF9pZCIsICJWaXNpdCIsICJzdWJDU1QiLCAiZWdhX3dlZWtzIiwgImNhc2UiKV0pLCBwYXJ0aWNpcGFudF9pZH5WaXNpdCwgdmFsdWUudmFyID0gImNhc2UiKQpuYW1lcyh6YXBwcy5jYXN0LjQpWzI6NF08LXBhc3RlKCJjYXNlXyIsbmFtZXMoemFwcHMuY2FzdC40KVsyOjRdLCBzZXA9IiIpCnphcHBzLmNhc3Q8LW1lcmdlKHphcHBzLmNhc3QuMSwgemFwcHMuY2FzdC4yLCBhbGw9VFJVRSkKemFwcHMuY2FzdDwtbWVyZ2UoemFwcHMuY2FzdCwgemFwcHMuY2FzdC4zLCBhbGw9VFJVRSkKemFwcHMuY2FzdDwtbWVyZ2UoemFwcHMuY2FzdCwgemFwcHMuY2FzdC40LCBhbGw9VFJVRSkKemFwcHMuY2FzdCRlZ2FfZGF5X0VWMV9kaWZmPC16YXBwcy5jYXN0JGVnYV9kYXlzX1Zpc2l0XzEtemFwcHMuY2FzdCRlZ2FfZGF5c19FbnJvbGxtZW50CnphcHBzLmNhc3QkZWdhX2RheV9WMVYyX2RpZmY8LXphcHBzLmNhc3QkZWdhX2RheXNfVmlzaXRfMi16YXBwcy5jYXN0JGVnYV9kYXlzX1Zpc2l0XzEKemFwcHMuY2FzdCRjYXNlX3BhdHRlcm48LXBhc3RlKHphcHBzLmNhc3QkY2FzZV9FbnJvbGxtZW50LCB6YXBwcy5jYXN0JGNhc2VfVmlzaXRfMSwgemFwcHMuY2FzdCRjYXNlX1Zpc2l0XzIsIHNlcD0iIikKCnphcHBzLm1ldGE8LXVuaXF1ZShyZWFkLmNzdigiWkFQUFMvcHJvY2Vzc2VkX2NsaW5pY2FsX2RhdGFfR0FQUFNfc3Vic2V0XzEyZGVjMjAyMy5jc3YiKSkgIyMgZXh0cmEgbWV0YWRhdGEgZm9yIHByb3BlciBtb2RlbGluZyB3aXRoIHNpbWlsYXIgY292YXJpYXRlcyB0byBNTEFXSToKemFwcHMubWV0YSR2aXNudW08LXN0cl9zcGxpdF9maXhlZChtYWtlLnVuaXF1ZSh6YXBwcy5tZXRhJE9SSUdfSUQsIHNlcCA9ICJfIiksICJfIiwgMilbLDJdCnphcHBzLm1ldGEkdmlzbnVtW3phcHBzLm1ldGEkdmlzbnVtICVpbiUgIiJdPC0iRW5yb2xsbWVudCIKIyBzZF9hZ2UuY2F0IC0tIFBXX0FHRQojICsgcHJlZ19vdXRjb21lIC0tIEdBR0VCUlRIICh3ZWVrcykgb3IgRVJMWV9QVEIKIyArIHBoX2VnYS5jYXQgLS0gRUdBXwojICsgYmx0ZXN0X3N5cHJlc3VsdHMgUlBSCiMgKyBzZXhfZnJlcS5jYXQgCiMgKyBjb25kb21fZnJlcV9jYXRfZiAKIyArIHAxX3N0YXR1c19mIAojICsgY3Vycl9wdG5ycyAKIyArIGdyYXZpZGl0eSAtLSBHUkFWSURJVFkKIyArIHBoX2Rpc2NoYXJnZSAKIyArIHdhdGVyIC0tIERSSU5LSU5HIFNPVVJDRSA9PSAxIG9yIDIKIyArIHBoX3NvcmVzX3VsY2VycyAtLSAKIyArIGVsZWN0cmljIC0tIEhIX0VMRUNUUklDSVRZCiMgKyBlZHVjYXQgLS0gUFdfRURVQ0FUSU9OCnphcHBzLm1ldGEkT1JJR19JRF9HQTwtcGFzdGUoemFwcHMubWV0YSRPUklHX0lELCB6YXBwcy5tZXRhJEdBLCBzZXA9Il8iKQpiYXNlbGluZS5zYW1wbGVzPC12ZWN0b3IoKQpmb3IoaSBpbiB1bmlxdWUoemFwcHMubWV0YSRPUklHX0lEKSl7CiAgczwtemFwcHMubWV0YVt6YXBwcy5tZXRhJE9SSUdfSUQgJWluJSBpLCBjKCJHQSIsICJPUklHX0lEX0dBIildCiAgYmFzZWxpbmUuc2FtcGxlczwtYXBwZW5kKGJhc2VsaW5lLnNhbXBsZXMsIHNbcyRHQSA9PSBtaW4ocyRHQSksICJPUklHX0lEX0dBIl0pCn0KemFwcHMubWV0YS5iYXNlbGluZTwtemFwcHMubWV0YVt6YXBwcy5tZXRhJE9SSUdfSURfR0EgJWluJSBiYXNlbGluZS5zYW1wbGVzLCBjKCJPUklHX0lEIiwgIlBXX0FHRSIsICJHQSIsICJHQUdFQlJUSCIsICJTWVBISUxJUyIsICJHUkFWSURJVFkiLCAiRFJJTktJTkdfU09VUkNFIiwgIkVSTFlfUFRCIiwgIlBSRUdfT1VUQ09NRSIsICJISVYiLCAiUFRCIildCgp6YXBwcy5tZXRhLmJhc2VsaW5lJFBJRDwtemFwcHMubWV0YS5iYXNlbGluZSRPUklHX0lECiN6YXBwcy5tZXRhLmJhc2VsaW5lJHNkX2FnZS5jYXQ8LWZhY3RvcihpZmVsc2UoemFwcHMubWV0YS5iYXNlbGluZSRQV19BR0UgJWluJSAxODoyNCwgIjE4LTI0IiwgaWZlbHNlKHphcHBzLm1ldGEuYmFzZWxpbmUkUFdfQUdFICVpbiUgMjU6MzAsICIyNS0zMCIsIGlmZWxzZSh6YXBwcy5tZXRhLmJhc2VsaW5lJFBXX0FHRSA8IDE4LCBOQSwgIjMxLTQyIikpKSwgb3JkZXJlZCA9IEYpCnphcHBzLm1ldGEuYmFzZWxpbmUkc2RfYWdlPC16YXBwcy5tZXRhLmJhc2VsaW5lJFBXX0FHRQoKI0dSQVZJRElUWTogSW5jbHVkaW5nIHRoaXMgcHJlZ25hbmN5LCBob3cgbWFueSB0aW1lcyBpbiB5b3VyIGxpZmUgaGF2ZSB5b3UgYmVlbiBwcmVnbmFudD8KemFwcHMubWV0YS5iYXNlbGluZSRncmF2aWRpdHk8LWZhY3RvcihpZmVsc2UoemFwcHMubWV0YS5iYXNlbGluZSRHUkFWSURJVFkgPiAwLCAiQXQgbGVhc3Qgb25lIHByaW9yIHByZWduYW5jeSIsICJObyBwcmlvciBwcmVnbmFuY2llcyIpKQoKI0RSSU5LSU5HX1NPVVJDRTogMT1QaXBlZC9Cb3R0bGUgV2F0ZXIvVGFua2VyIFRydWNrLCAyPVR1YmV3ZWxsKGFueXR5cGUpLCAzPVJhaW4gV2F0ZXIsIDQ9U3VyZmFjZSBXYXRlcihSaXZlcixQb25kLGV0Yy4pCnphcHBzLm1ldGEuYmFzZWxpbmUkd2F0ZXI8LWZhY3RvcihpZmVsc2UoemFwcHMubWV0YS5iYXNlbGluZSREUklOS0lOR19TT1VSQ0UgJWluJSBjKDEsIDIpLCAiWWVzIiwgIk5vIikpCgojU1lQSElMSVM6IFN5cGhpbGlzIHRlc3RpbmcgYXQgZW5yb2xsbWVudDogMCA9IG5vbi1yZWFjdGl2ZS9uZWdhdGl2ZTsgMSA9IHJlYWN0aXZlL3Bvc2l0aXZlOyAyID0gaW5kZXRlcm1pbmF0ZSAKemFwcHMubWV0YS5iYXNlbGluZSRibHRlc3Rfc3lwcmVzdWx0czwtZmFjdG9yKGlmZWxzZSh6YXBwcy5tZXRhLmJhc2VsaW5lJFNZUEhJTElTICVpbiUgMCwgIk5vIiwgIlllcyIpKQoKemFwcHMuY2FzdDwtbWVyZ2UoemFwcHMuY2FzdCwgemFwcHMubWV0YS5iYXNlbGluZSwgYWxsPVRSVUUsIGJ5Lng9InBhcnRpY2lwYW50X2lkIiwgYnkueT0iT1JJR19JRCIpCmBgYAoKIyMjIyBHZXQgSElWIG5lZyBjYXNlcyB3aGljaCBtYXRjaCB0aGUgYWJvdmUgY3JpdGVyaWEgYXMgYmVzdCB3ZSBjYW4uCmBgYHtyfQojIyMgTUxBV0kKYTIubWcuZGYuaGl2bmVnPC1hMi5tZy5kZlthMi5tZy5kZiRoaXYgJWluJSAiSElWLU5lZ2F0aXZlIiwgYygiUElEIiwgIjEiLCAiMiIsICJzZF9hZ2UiLCAicGhfZWdhIiwgImJsdGVzdF9zeXByZXN1bHRzIiwgImdyYXZpZGl0eSIsICJ3YXRlciIsICJkYXRlX2RpZmYiKV0KYTIubWcuZGYuaGl2bmVnJHN0dWR5PC0iVG9uc2UgUGFtb2R6aSAyXG4oUHJFUC1leHBvc2VkKSIKYTIubWcuZGYuaGl2bmVnJGV4cG9zdXJlPC0iUHJFUCIKCiMjIyBaQVBQUwojIyBHRVQgSElWLU5FRyBTVUJKRUNUUyBXSVRIIFNJTUlMQVIgVElNRSBCRVRXRUVOIFZJU0lUUwpldjEuc3ViajwtemFwcHMuY2FzdFt6YXBwcy5jYXN0JGNhc2VfcGF0dGVybiAlaW4lICIwMDAiICYgemFwcHMuY2FzdCRlZ2FfZGF5X0VWMV9kaWZmIDw9IDQwLCAicGFydGljaXBhbnRfaWQiXSAjIG49MTA2IEVucm9sbC0+VjEKdjF2Mi5zdWJqPC16YXBwcy5jYXN0W3phcHBzLmNhc3QkY2FzZV9wYXR0ZXJuICVpbiUgIjAwMCIgJiB6YXBwcy5jYXN0JGVnYV9kYXlfVjFWMl9kaWZmIDw9IDQwLCAicGFydGljaXBhbnRfaWQiXSAjIG49MTIxIFYxLT5WMgp2MXYyLnN1Ymo8LXYxdjIuc3VialshdjF2Mi5zdWJqICVpbiUgZXYxLnN1YmpdICMjIHJlbW92ZSBhbnkgYWxyZWFkeSBjaG9zZW4gZm9yIGVucm9sbG1lbnQtPlYxCgp6YXBwcy5oaXYubmVnPC16YXBwcy5jYXN0W3phcHBzLmNhc3QkcGFydGljaXBhbnRfaWQgJWluJSB1bmlxdWUoYyhldjEuc3ViaiwgdjF2Mi5zdWJqKSksIF0gI249MjU2CiMjIyAKCiMjIyBDQVBUVVJFIEdFU1RBVElPTkFMIEFHRSBPRiBTQU1QTEUgREVQRU5ESU5HIE9OIElGIEVOUk9MTC0+VjEgT1IgVjEtPlYyCnphcHBzLmhpdi5uZWckZWdhX3dlZWtzPC1hcy5udW1lcmljKGlmZWxzZSh6YXBwcy5oaXYubmVnJHBhcnRpY2lwYW50X2lkICVpbiUgZXYxLnN1YmosIAogICAgICAgICAgICAgICAgICAgICAgICAgICAgICAgICAgICAgICAgICAgYXMuY2hhcmFjdGVyKHphcHBzLmhpdi5uZWckZWdhX3dlZWtzX0Vucm9sbG1lbnQpLCAKICAgICAgICAgICAgICAgICAgICAgICAgICAgICAgICAgICAgICAgICAgIGlmZWxzZSh6YXBwcy5oaXYubmVnJHBhcnRpY2lwYW50X2lkICVpbiUgdjF2Mi5zdWJqLCAKICAgICAgICAgICAgICAgICAgICAgICAgICAgICAgICAgICAgICAgICAgICAgICAgICBhcy5jaGFyYWN0ZXIoemFwcHMuaGl2Lm5lZyRlZ2Ffd2Vla3NfVmlzaXRfMSksIE5BKSkpCiAgIyAgIE1pbi4gMXN0IFF1LiAgTWVkaWFuICAgIE1lYW4gM3JkIFF1LiAgICBNYXguIAogICMgMTcuMTAgICAxOS41MCAgIDI3LjYwICAgMjUuNDggICAyOC4yMCAgIDMwLjAwICMjIG1ham9yaXR5IGluIHRoZSBzZWNvbmQgdHJpbWVzdGVyLCBzb21lIGluIDFzdC4KemFwcHMuaGl2Lm5lZyRwaF9lZ2E8LXphcHBzLmhpdi5uZWckZWdhX3dlZWtzCgp6YXBwcy5oaXYubmVnJGRhdGVfZGlmZjwtYXMubnVtZXJpYyhpZmVsc2UoemFwcHMuaGl2Lm5lZyRwYXJ0aWNpcGFudF9pZCAlaW4lIGV2MS5zdWJqLCBhcy5jaGFyYWN0ZXIoemFwcHMuaGl2Lm5lZyRlZ2FfZGF5X0VWMV9kaWZmKSwgaWZlbHNlKHphcHBzLmhpdi5uZWckcGFydGljaXBhbnRfaWQgJWluJSB2MXYyLnN1YmosIGFzLmNoYXJhY3Rlcih6YXBwcy5oaXYubmVnJGVnYV9kYXlfVjFWMl9kaWZmKSwgTkEpKSkKCnphcHBzLmhpdi5uZWcuc2FtcGxlcy50by5nZXQ8LWMocGFzdGUoZXYxLnN1YmosICJFbnJvbGxtZW50Iiwgc2VwID0gIl8iKSwgcGFzdGUoZXYxLnN1YmosICJWaXNpdF8xIiwgc2VwID0gIl8iKSwgcGFzdGUodjF2Mi5zdWJqLCAiVmlzaXRfMSIsIHNlcCA9ICJfIiksIHBhc3RlKHYxdjIuc3ViaiwgIlZpc2l0XzIiLCBzZXAgPSAiXyIpKQp6YXBwcyRwYXJ0aWNpcGFudF9pZF9WaXNpdDwtcGFzdGUoemFwcHMkcGFydGljaXBhbnRfaWQsIHphcHBzJFZpc2l0LCBzZXA9Il8iKQoKemFwcHMuaGl2Lm5lZy5jc3Q8LXphcHBzW3phcHBzJHBhcnRpY2lwYW50X2lkX1Zpc2l0ICVpbiUgemFwcHMuaGl2Lm5lZy5zYW1wbGVzLnRvLmdldCwgXQoKIyMjIFZJU0lUUwp6YXBwcy5oaXYubmVnLmNzdCR2aXNudW08LWlmZWxzZSh6YXBwcy5oaXYubmVnLmNzdCRwYXJ0aWNpcGFudF9pZCAlaW4lIGV2MS5zdWJqLCBpZmVsc2UoemFwcHMuaGl2Lm5lZy5jc3QkVmlzaXQgJWluJSAiRW5yb2xsbWVudCIsIDEsIDIpLCBOQSkKemFwcHMuaGl2Lm5lZy5jc3QkdmlzbnVtPC1pZmVsc2UoemFwcHMuaGl2Lm5lZy5jc3QkcGFydGljaXBhbnRfaWQgJWluJSB2MXYyLnN1YmosIGlmZWxzZSh6YXBwcy5oaXYubmVnLmNzdCRWaXNpdCAlaW4lICJWaXNpdF8xIiwgMSwgMiksIGFzLmNoYXJhY3Rlcih6YXBwcy5oaXYubmVnLmNzdCR2aXNudW0pKQoKCnphcHBzLmhpdi5uZWcuY2FzdDwtcmVzaGFwZTI6OmRjYXN0KHphcHBzLmhpdi5uZWcuY3N0LCBwYXJ0aWNpcGFudF9pZH52aXNudW0sIHZhbHVlLnZhciA9ICJzdWJDU1QiKQp6YXBwcy5oaXYubmVnLmNhc3Qkc3R1ZHk8LSJaQVBQU1xuKFVuZXhwb3NlZCkiCnphcHBzLmhpdi5uZWcuY2FzdCRleHBvc3VyZTwtIk5vIFByRVAiCnphcHBzLmhpdi5uZWckcHJlZ19vdXRjb21lPC1pZmVsc2UoemFwcHMuaGl2Lm5lZyRQVEIgPT0gMCwgIlRlcm0gRGVsaXZlcnkiLCAiUHJldGVybSBEZWxpdmVyeSIpCgp6YXBwcy5oaXYubmVnLmNhc3Q8LW1lcmdlKHphcHBzLmhpdi5uZWcuY2FzdCwgemFwcHMuaGl2Lm5lZywgYWxsLng9VFJVRSkKIytzZF9hZ2UuY2F0K3BoX2VnYS5jYXQrYmx0ZXN0X3N5cHJlc3VsdHMgKyBncmF2aWRpdHkgKyBlbGVjdHJpYyArIGVkdWNhdCArIHdhdGVyCgoKIyMjIENPTUJJTkUgWkFQUFMgQU5EIFRQMiMjIyAKY21wLnRyYW5zaXRpb25zPC1yYmluZChhMi5tZy5kZi5oaXZuZWdbLCBjKCJQSUQiLCAiMSIsICIyIiwgInNkX2FnZSIsICJwaF9lZ2EiLCAiYmx0ZXN0X3N5cHJlc3VsdHMiLCAiZ3JhdmlkaXR5IiwgIndhdGVyIiwgInN0dWR5IiwgImV4cG9zdXJlIiwgImRhdGVfZGlmZiIpXSwgemFwcHMuaGl2Lm5lZy5jYXN0WyxjKCJQSUQiLCAiMSIsICIyIiwgInNkX2FnZSIsICJwaF9lZ2EiLCAiYmx0ZXN0X3N5cHJlc3VsdHMiLCAiZ3JhdmlkaXR5IiwgIndhdGVyIiwgInN0dWR5IiwgImV4cG9zdXJlIiwgImRhdGVfZGlmZiIpXSkKY21wLnRyYW5zaXRpb25zPC1jbXAudHJhbnNpdGlvbnNbY29tcGxldGUuY2FzZXMoY21wLnRyYW5zaXRpb25zKSwgXQojIyMgVEFCTEUgMSMjIyAKIyBGSVhFRCBFRkZFQ1RTIC0gdG8gcmVkdWNlIHRoZSBjaGFuY2VzIHRoYXQgYW4gSElWL21pY3JvYmlvbWUgYXNzb2NpYXRpb24gaXMgY29uZm91bmRlZCBieSBzb21ldGhpbmcgZWxzZS4KbGFiZWwoY21wLnRyYW5zaXRpb25zJHNkX2FnZSk8LSJBZ2UgYXQgZW5yb2xsbWVudCAoeWVhcnMpIgoKIyMgcGhfZWdhOiBlc3RpbWF0ZWQgZ2VzdGF0aW9uYWwgYWdlIGF0IGVucm9sbG1lbnQ6IENvbnRpbnVvdXMgCmxhYmVsKGNtcC50cmFuc2l0aW9ucyRwaF9lZ2EpPC0iR2VzdGF0aW9uYWwgYWdlIGF0IGVucm9sbG1lbnQgKHdlZWtzKSIKCiMgVFAyOiBHcmF2aWRpdHk6ICIwPU5vIHByaW9yIHByZWduYW5jaWVzLCAxPUF0IGxlYXN0IG9uZSBwcmlvciBwcmVnbmFuY3kiCiMgWkFQUFM6IEdyYXZpZGl0eTogIjA9Tm8gcHJpb3IgcHJlZ25hbmNpZXMsIDE9QXQgbGVhc3Qgb25lIHByaW9yIHByZWduYW5jeSIKbGFiZWwoY21wLnRyYW5zaXRpb25zJGdyYXZpZGl0eSk8LSJHcmF2aWRpdHkiCgojIyBwaF9zeXBoaWxpc190b2xkOiBzZWxmLXJlcG9ydCBzeXBoaWxpcyBwYXN0IDMgbW9udGhzLiBDQVQKIyBSZXN1bHRzIG9mIHN5cGhpbGlzIHRlc3QgZHVyaW5nIGluZGV4IHByZWduYW5jeSAoYW50ZW5hdGFsIHJlY29yZCk6ICIwPU5vbnJlYWN0aXZlLCAxPVJlYWN0aXZlLCA5OTk9Tm8gdGVzdCByZXN1bHRzIHJlY29yZGVkIgpsYWJlbChjbXAudHJhbnNpdGlvbnMkYmx0ZXN0X3N5cHJlc3VsdHMpPC0iRGlhZ25vc2VkIHdpdGggc3lwaGlsaXMgaW4gcGFzdCAzIG1vbnRocyIKCiMgUnVubmluZyB3YXRlciBpbiBob21lOiAiMD1ObywgMT1ZZXMiCmxhYmVsKGNtcC50cmFuc2l0aW9ucyR3YXRlcik8LSJSdW5uaW5nIHdhdGVyIGluIGhvbWUiCgpsYWJlbChjbXAudHJhbnNpdGlvbnMkZGF0ZV9kaWZmKTwtIkRheXMgYmV0d2VlbiBNMCBhbmQgTTEiCgp0YWJsZTEofiBzZF9hZ2UgKyBwaF9lZ2EgKyBncmF2aWRpdHkgKyBibHRlc3Rfc3lwcmVzdWx0cyArIHdhdGVyICsgZGF0ZV9kaWZmIHwgc3R1ZHksIGRhdGE9Y21wLnRyYW5zaXRpb25zLCBvdmVyYWxsPUYsIGV4dHJhLmNvbD1saXN0KGBQLXZhbHVlYD1wdmFsdWUpKQoKIyMjIEVTVElNQVRFIFRSQU5TSVRJT04gQlkgUFJFUCBFWFBPU1VSRQpjbXAudHJhbnNpdGlvbnMkZXhwb3N1cmU8LWZhY3RvcihjbXAudHJhbnNpdGlvbnMkZXhwb3N1cmUsIGxldmVscz1jKCJObyBQckVQIiwgIlByRVAiKSkKCmNtcC50cmFuc2l0aW9ucyRjc3QxPC1jbXAudHJhbnNpdGlvbnMkYDFgCmNtcC50cmFuc2l0aW9ucyRjc3QxPC1pZmVsc2UoY21wLnRyYW5zaXRpb25zJGAxYCAlaW4lIGMoIkktQSIsICJJLUIiKSwgIkkiLCBhcy5jaGFyYWN0ZXIoY21wLnRyYW5zaXRpb25zJGNzdDEpKSAKY21wLnRyYW5zaXRpb25zJGNzdDE8LWlmZWxzZShjbXAudHJhbnNpdGlvbnMkYDFgICVpbiUgYygiSUlJLUEiLCAiSUlJLUIiKSwgIklJSSIsIGFzLmNoYXJhY3RlcihjbXAudHJhbnNpdGlvbnMkY3N0MSkpIApjbXAudHJhbnNpdGlvbnMkY3N0MTwtaWZlbHNlKGNtcC50cmFuc2l0aW9ucyRgMWAgJWluJSBjKCJJVi1BIiwgIklWLUIiLCAiSVYtQyIsICJJVi1DMCIpLCAiSVYiLCBhcy5jaGFyYWN0ZXIoY21wLnRyYW5zaXRpb25zJGNzdDEpKSAKCmNtcC50cmFuc2l0aW9ucyRjc3QyPC1jbXAudHJhbnNpdGlvbnMkYDJgCmNtcC50cmFuc2l0aW9ucyRjc3QyPC1pZmVsc2UoY21wLnRyYW5zaXRpb25zJGAyYCAlaW4lIGMoIkktQSIsICJJLUIiKSwgIkkiLCBhcy5jaGFyYWN0ZXIoY21wLnRyYW5zaXRpb25zJGNzdDIpKSAKY21wLnRyYW5zaXRpb25zJGNzdDI8LWlmZWxzZShjbXAudHJhbnNpdGlvbnMkYDJgICVpbiUgYygiSUlJLUEiLCAiSUlJLUIiKSwgIklJSSIsIGFzLmNoYXJhY3RlcihjbXAudHJhbnNpdGlvbnMkY3N0MikpIApjbXAudHJhbnNpdGlvbnMkY3N0MjwtaWZlbHNlKGNtcC50cmFuc2l0aW9ucyRgMmAgJWluJSBjKCJJVi1BIiwgIklWLUIiLCAiSVYtQyIsICJJVi1DMCIpLCAiSVYiLCBhcy5jaGFyYWN0ZXIoY21wLnRyYW5zaXRpb25zJGNzdDIpKSAKCmNtcC50cmFuc2l0aW9ucyR0cmFuc2l0aW9uPC1mYWN0b3IoaWZlbHNlKGNtcC50cmFuc2l0aW9ucyRjc3QxID09IGNtcC50cmFuc2l0aW9ucyRjc3QyLCAiTm8gQ2hhbmdlIiwgIkNoYW5nZWQiKSwgbGV2ZWxzPWMoIk5vIENoYW5nZSIsICJDaGFuZ2VkIikpCm9kZHNyYXRpbyh0YWJsZShjbXAudHJhbnNpdGlvbnMkZXhwb3N1cmUsIGNtcC50cmFuc2l0aW9ucyR0cmFuc2l0aW9uKSkKIyMgSW5kaXZpZHVhbHMgZXhwb3NlZCB0byBwcmVwIHdlcmUgbm8gbW9yZSBsaWtlbHkgdG8gdHJhbnNpdGlvbiBDU1RzIHRoYW4gdGhvc2UgdW5leHBvc2VkLiAKdG8udGVzdDwtY21wLnRyYW5zaXRpb25zCnRvLnRlc3Qkb3V0Y29tZWJpbjwtZmFjdG9yKGlmZWxzZSh0by50ZXN0JHRyYW5zaXRpb24gJWluJSAiQ2hhbmdlZCIsIDEsIDApKQp0aGUubW9kZWw8LWdsbShvdXRjb21lYmluIH4gZXhwb3N1cmUgKyBzZF9hZ2UgKyBwaF9lZ2EgKyBncmF2aWRpdHkgKyBibHRlc3Rfc3lwcmVzdWx0cyArIHdhdGVyICsgZGF0ZV9kaWZmLCBkYXRhID0gdG8udGVzdCwgZmFtaWx5ID0gImJpbm9taWFsIikgIyMgMjEgc2FtcGxlcyByZW1vdmVkIGR1ZSB0byBtaXNzaW5nbmVzcyAobW9zdGx5IHphcHBzKQphcy5kYXRhLmZyYW1lKGJyb29tOjp0aWR5KHRoZS5tb2RlbCwgY29uZi5pbnQgPSBULCBleHBvbmVudGlhdGUgPSBUKSkKCmxhYmVsKGNtcC50cmFuc2l0aW9ucyR0cmFuc2l0aW9uKTwtIkNTVCB0cmFuc2l0aW9uIGJldHdlZW4gTTAgYW5kIE0xIgp0YWJsZTEofiBzZF9hZ2UgKyBwaF9lZ2EgKyBncmF2aWRpdHkgKyBibHRlc3Rfc3lwcmVzdWx0cyArIHdhdGVyICsgZGF0ZV9kaWZmICsgdHJhbnNpdGlvbnwgc3R1ZHksIGRhdGE9Y21wLnRyYW5zaXRpb25zLCBvdmVyYWxsPUYsIGV4dHJhLmNvbD1saXN0KGBQLXZhbHVlYD1wdmFsdWUpKQoKCmNtcC50cmFuc2l0aW9ucyR0cmFuc2l0aW9uX2RldDwtZmFjdG9yKGlmZWxzZShjbXAudHJhbnNpdGlvbnMkdHJhbnNpdGlvbiAlaW4lICJObyBDaGFuZ2UiLCAiTm8gQ2hhbmdlIiwgcGFzdGUoY21wLnRyYW5zaXRpb25zJGNzdDEsIGNtcC50cmFuc2l0aW9ucyRjc3QyLCBzZXA9Ilxu4oaTXG4iKSksIGxldmVscz1jKCJObyBDaGFuZ2UiLCAiSVxu4oaTXG5JSSIsICJJXG7ihpNcbklJSSIsICJJXG7ihpNcbklWIiwgIklJXG7ihpNcbkkiLCAgIklJXG7ihpNcbklJSSIsICJJSVxu4oaTXG5JViIsICJJSUlcbuKGk1xuSSIsIklJSVxu4oaTXG5JSSIsICJJSUlcbuKGk1xuSVYiLCAiSUlJXG7ihpNcblYiLCAiSVZcbuKGk1xuSSIsICJJVlxu4oaTXG5JSSIsIklWXG7ihpNcbklJSSIsICJWXG7ihpNcbkkiLCAiVlxu4oaTXG5JViIpKQpsYWJlbChjbXAudHJhbnNpdGlvbnMkdHJhbnNpdGlvbl9kZXQpPC0iQ1NUcyBmcm9tIE0wIHRvIE0xIgoKIyMgU3BlY2lmaWNhbGx5IHRlc3QgdGhlIHRyYW5zaXRpb24gZnJvbSBDU1QgSUlJIHRvIElWIGJldHdlZW4gZXhwb3NlZCBhbmQgdW5leHBvc2VkCnRvLnRlc3Q8LWNtcC50cmFuc2l0aW9uc1tjbXAudHJhbnNpdGlvbnMkdHJhbnNpdGlvbiAlaW4lICJDaGFuZ2VkIiwgXQp0by50ZXN0JG91dGNvbWViaW48LWlmZWxzZSh0by50ZXN0JGNzdDIgJWluJSAiSVYiLCAxLCAwKQp0aGUubW9kZWw8LWdsbShvdXRjb21lYmluIH4gZXhwb3N1cmUgKyBzZF9hZ2UgKyBwaF9lZ2EgKyBncmF2aWRpdHkgKyBibHRlc3Rfc3lwcmVzdWx0cyArIHdhdGVyICsgZGF0ZV9kaWZmLCBkYXRhID0gdG8udGVzdCwgZmFtaWx5ID0gImJpbm9taWFsIikgIyMgMjEgc2FtcGxlcyByZW1vdmVkIGR1ZSB0byBtaXNzaW5nbmVzcyAobW9zdGx5IHphcHBzKQphcy5kYXRhLmZyYW1lKGJyb29tOjp0aWR5KHRoZS5tb2RlbCwgY29uZi5pbnQgPSBULCBleHBvbmVudGlhdGUgPSBUKSkKYGBgCgoKCgoKCgojIEFpbSAzLCBleHBsb3JlIHNQVEIKQWltIDMgKGV4cGxvcmF0b3J5KTogVG8gZGVzY3JpYmUgd2hldGhlciB0aGUgdmFnaW5hbCBtaWNyb2Jpb3RhIGNvbXBvc2l0aW9uLCBzdHJ1Y3R1cmUsIGFuZCBjaGFuZ2VzIGRpZmZlciBiZXR3ZWVuIHByZWduYW50IHdvbWVuIHdobyBoYXZlIGEgc3BvbnRhbmVvdXMgcHJldGVybSBiaXJ0aCAoU1BUQikgY29tcGFyZWQgdG8gd29tZW4gd2hvIGRlbGl2ZXIgYXQgdGVybS4KCkFuYWx5c2lzIHBsYW46IFVzaW5nIHRoZSBkZWxpdmVyeSBvdXRjb21lcywgd2Ugd2lsbCBjb21wYXJlIGhvdyB0aGUgdmFnaW5hbCBtaWNyb2Jpb3RhIGNvbXBvc2l0aW9uIGFuZCBzdHJ1Y3R1cmUgZGlmZmVyIGJldHdlZW4gdGhlIHdvbWVuIHdpdGggU1BUQiBhbmQgdGhvc2UgdGhhdCBkZWxpdmVyIGF0IHRlcm0uIFdlIGRlZmluZWQgcHJldGVybSBkZWxpdmVyeSBhcyBkZWxpdmVyeSBhdCBsZXNzIHRoYW4gMzcgY29tcGxldGVkIHdlZWtzIG9mIHByZWduYW5jeS4gU1BUQiBpbmNsdWRlIHByZXRlcm0gbGFib3IsIHByZXRlcm0gc3BvbnRhbmVvdXMgcnVwdHVyZSBvZiBtZW1icmFuZXMsIHByZXRlcm0gcHJlbWF0dXJlIHJ1cHR1cmUgb2YgbWVtYnJhbmVzIChQUFJPTSkgYW5kIGNlcnZpY2FsIHdlYWtuZXNzOyB3ZSBleGNsdWRlIGluZGljYXRlZCBwcmV0ZXJtIGRlbGl2ZXJ5IGZvciBtYXRlcm5hbCBvciBmZXRhbCBjb25kaXRpb25zLiBXZSB3aWxsIHVzZSBjbGluaWNhbCBkYXRhIGZyb20gcGFydGljaXBhbnQgaW50ZXJ2aWV3IGFzIHdlbGwgYXMgdGhlIG1lZGljYWwgYW5kIG9ic3RldHJpYyBoaXN0b3J5LCBpbnRyYXBhcnR1bSBtYW5hZ2VtZW50IGFuZCBiaXJ0aCBvdXRjb21lcyB0aGF0IHdlcmUgcmVjb3JkZWQgZm9yIGFsbCBwYXJ0aWNpcGFudHMuIFdlIHdpbGwgYXNzZXNzIGFzc29jaWF0aW9ucyBiZXR3ZWVuIGVhY2ggb2YgdGhlIG1lYXN1cmVzIG5vdGVkIGluIEFpbSAyIGFib3ZlIGFuZCBTUFRCIHVzaW5nIGxvZ2lzdGljIHJlZ3Jlc3Npb24gaW4gYm90aCB1bml2YXJpYWJsZSBhbmQgbXVsdGl2YXJpYWJsZSBtb2RlbHMuIFdlIHdpbGwgYWNjb3VudCBmb3IgcG9zc2libGUgY29uZm91bmRlcnM6IG1hdGVybmFsIGFnZSwgcGFyaXR5LCBlZHVjYXRpb24gYW5kIHNvY2lvZWNvbm9taWMgc3RhdHVzLCBudXRyaXRpb24sIGdlc3RhdGlvbmFsIGFnZSBhdCB0aGUgdGltZSBvZiBzYW1wbGUgY29sbGVjdGlvbiwgSElWIHZpcmFsIGxvYWQgKEZvciBISVYrIHdvbWVuKS4KCiMjIEFzc29jaWF0aW9uIGJldHdlZW4gQ1NUIElWIGFuZCBQVEIKYGBge3J9Cm1ldGE8LXJlYWQuY3N2KCJGZWJfMTBfZW1haWwvVFAyX3ByZWdvdXRjb21lX25lZWRlZC5jc3YiKQphMy5tZzwtYTEubWcKYTMubWckcHJlZ19vdXRjb21lPC1tZXRhJHByZWdfb3V0Y29tZVttYXRjaChhMy5tZyRtYXJ5bGFuZF9JRCwgbWV0YSRtYXJ5bGFuZF9JRCldCmEzLm1nJHByZWdfb3V0Y29tZTwtaWZlbHNlKGlzLm5hKGEzLm1nJHByZWdfb3V0Y29tZSksIGEyLm1nLnJpY2gkcHJlZ19vdXRjb21lW21hdGNoKGEzLm1nJG1hcnlsYW5kX0lELCBhMi5tZy5yaWNoJG1hcnlsYW5kX0lEKV0sIGFzLmNoYXJhY3RlcihhMy5tZyRwcmVnX291dGNvbWUpKQphMy5tZyRwcmVnX291dGNvbWVfY2xlYW48LWZhY3RvcihpZmVsc2UoZ3JlcGwoIlByZXRlcm0gRGVsaXZlcnkiLCBhMy5tZyRwcmVnX291dGNvbWUpLCAiUHJldGVybSBEZWxpdmVyeSIsIGlmZWxzZShncmVwbCgiVGVybSBEZWxpdmVyeSIsIGEzLm1nJHByZWdfb3V0Y29tZSksICJUZXJtIERlbGl2ZXJ5IiwgTkEpKSwgbGV2ZWxzPWMoIlRlcm0gRGVsaXZlcnkiLCAiUHJldGVybSBEZWxpdmVyeSIpKQphMy5tZyRwcmVnX291dGNvbWVfY2xlYW48LXJlbGV2ZWwoYTMubWckcHJlZ19vdXRjb21lX2NsZWFuLCByZWY9IlRlcm0gRGVsaXZlcnkiKQphMy5tZzwtYTMubWdbIWlzLm5hKGEzLm1nJHByZWdfb3V0Y29tZV9jbGVhbiksIF0KCiMjIyBURVNUIEhJViB2cyBzUFRCCmEzLm1nJGhpdjwtcmVsZXZlbChhMy5tZyRoaXYsIHJlZj0iSElWLU5lZ2F0aXZlIikKdGhlLm1vZGVsPC1nbG0ocHJlZ19vdXRjb21lX2NsZWFuIH4gaGl2ICsgc2RfYWdlICsgcGhfZWdhICsgZ3JhdmlkaXR5ICsgcDFfc3RhdHVzICsgYmx0ZXN0X3N5cHJlc3VsdHMgKyBwaF9kaXNjaGFyZ2UgKyBwaF9zb3Jlc191bGNlcnMgKyB3YXRlciwgZGF0YSA9IGEzLm1nLCBmYW1pbHkgPSAiYmlub21pYWwiKSAjIyA0MSBzYW1wbGVzIHJlbW92ZWQgZHVlIHRvIG1pc3NpbmduZXNzIChtb3N0bHkgemFwcHMpCndsaGl2LnNwdGI8LWFzLmRhdGEuZnJhbWUoYnJvb206OnRpZHkodGhlLm1vZGVsLCBjb25mLmludCA9IFQsIGV4cG9uZW50aWF0ZSA9IFQpKVsyLF0KdGFibGUoYTMubWckaGl2LCBhMy5tZyRwcmVnX291dGNvbWVfY2xlYW4pCgojIyMgR0VUIE0wIENTVAphMy5tZyRDU1RfTTA8LWEzLm1nJENTVAphMy5tZyRDU1RfTTA8LWdzdWIoIklWLUEiLCAiSVYiLCBhMy5tZyRDU1RfTTApCmEzLm1nJENTVF9NMDwtZ3N1YigiSVYtQiIsICJJViIsIGEzLm1nJENTVF9NMCkKYTMubWckQ1NUX00wPC1nc3ViKCJJVi1DIiwgIklWIiwgYTMubWckQ1NUX00wKQphMy5tZyRDU1RfTTA8LWZhY3RvcihhMy5tZyRDU1RfTTApCmEzLm1nJENTVElWX00wX2V4cDwtZmFjdG9yKGlmZWxzZShncmVwbCgiSVYiLCBhMy5tZyRDU1RfTTApLCAxLCAwKSkKCiMjIyBHRVQgTTEgQ1NUCnN3YWIyPC1hMi5tZ1thMi5tZyRzd2FiICVpbiUgMiwgXQphMy5tZyRDU1RfTTE8LXN3YWIyW21hdGNoKGEzLm1nJHBpZCwgc3dhYjIkUElEKSwgIkNTVCJdCmEzLm1nJENTVF9NMTwtZ3N1YigiSVYtQSIsICJJViIsIGEzLm1nJENTVF9NMSkKYTMubWckQ1NUX00xPC1nc3ViKCJJVi1CIiwgIklWIiwgYTMubWckQ1NUX00xKQphMy5tZyRDU1RfTTE8LWdzdWIoIklWLUMiLCAiSVYiLCBhMy5tZyRDU1RfTTEpCmEzLm1nJENTVF9NMTwtZmFjdG9yKGEzLm1nJENTVF9NMSkKCiMjIyBURVNUIENTVCBJViBBVCBNMCBPUiBNMSBBTkQgc1BUQgphMy5tZyRDU1RJVl9hbnk8LWZhY3RvcihpZmVsc2UoYTMubWckQ1NUX00wICVpbiUgIklWIiB8IGEzLm1nJENTVF9NMSAlaW4lICJJViIsIDEsIDApKQp0aGUubW9kZWw8LWdsbShwcmVnX291dGNvbWVfY2xlYW4gfiBDU1RJVl9hbnkgKyBzZF9hZ2UgKyBwaF9lZ2EgKyBncmF2aWRpdHkgKyBwMV9zdGF0dXMgKyBibHRlc3Rfc3lwcmVzdWx0cyArIHBoX2Rpc2NoYXJnZSArIHBoX3NvcmVzX3VsY2VycyArIHdhdGVyLCBkYXRhID0gYTMubWcsIGZhbWlseSA9ICJiaW5vbWlhbCIpIAphcy5kYXRhLmZyYW1lKGJyb29tOjp0aWR5KHRoZS5tb2RlbCwgY29uZi5pbnQgPSBULCBleHBvbmVudGlhdGUgPSBUKSkKY3N0aXYubTAubTEuc3B0YjwtYXMuZGF0YS5mcmFtZShicm9vbTo6dGlkeSh0aGUubW9kZWwsIGNvbmYuaW50ID0gVCwgZXhwb25lbnRpYXRlID0gVCkpWzIsXQp0YWJsZShhMy5tZ1ssICJDU1RJVl9hbnkiXSwgYTMubWdbLCAicHJlZ19vdXRjb21lX2NsZWFuIl0pCgojIyMgVEVTVCBBTlkgQ0hBTkdFIEFORCBzUFRCCmEzLm1nPC1hMy5tZ1shaXMubmEoYTMubWckQ1NUX00xKSwgXQphMy5tZyR0cmFuc2l0aW9uPC1mYWN0b3IoaWZlbHNlKCFhMy5tZyRDU1RfTTAgPT0gYTMubWckQ1NUX00xLCAxLCAwKSkKdGhlLm1vZGVsPC1nbG0ocHJlZ19vdXRjb21lX2NsZWFuIH4gdHJhbnNpdGlvbiArIHNkX2FnZSArIHBoX2VnYSArIGdyYXZpZGl0eSArIHAxX3N0YXR1cyArIGJsdGVzdF9zeXByZXN1bHRzICsgcGhfZGlzY2hhcmdlICsgcGhfc29yZXNfdWxjZXJzICsgd2F0ZXIsIGRhdGEgPSBhMy5tZ1shaXMubmEoYTMubWckQ1NUX00xKSwgXSwgZmFtaWx5ID0gImJpbm9taWFsIikgCmNoYW5nZS5jc3Quc3B0YjwtYXMuZGF0YS5mcmFtZShicm9vbTo6dGlkeSh0aGUubW9kZWwsIGNvbmYuaW50ID0gVCwgZXhwb25lbnRpYXRlID0gVCkpWzIsXQp0YWJsZShhMy5tZ1shaXMubmEoYTMubWckQ1NUX00xKSwgInRyYW5zaXRpb24iXSwgYTMubWdbIWlzLm5hKGEzLm1nJENTVF9NMSksICJwcmVnX291dGNvbWVfY2xlYW4iXSkKCiMjIyBURVNUIFRSQU5TSVRJT04gVE8gQ1NUIElWIEFUIE0xIEFORCBzUFRCCmEzLm1nJENTVElWX3RyYW5zX2V4cDwtZmFjdG9yKGlmZWxzZSghZ3JlcGwoIklWIiwgYTMubWckQ1NUX00wKSAmIGdyZXBsKCJJViIsIGEzLm1nJENTVF9NMSksIDEsIDApKQp0aGUubW9kZWw8LWdsbShwcmVnX291dGNvbWVfY2xlYW4gfiBDU1RJVl90cmFuc19leHAgKyBzZF9hZ2UgKyBwaF9lZ2EgKyBncmF2aWRpdHkgKyBwMV9zdGF0dXMgKyBibHRlc3Rfc3lwcmVzdWx0cyArIHBoX2Rpc2NoYXJnZSArIHBoX3NvcmVzX3VsY2VycyArIHdhdGVyLCBkYXRhID0gYTMubWdbIWlzLm5hKGEzLm1nJENTVF9NMSksIF0sIGZhbWlseSA9ICJiaW5vbWlhbCIpIApjaGFuZ2UudG8uY3N0aXYuc3B0YjwtYXMuZGF0YS5mcmFtZShicm9vbTo6dGlkeSh0aGUubW9kZWwsIGNvbmYuaW50ID0gVCwgZXhwb25lbnRpYXRlID0gVCkpWzIsXQp0YWJsZShhMy5tZ1shaXMubmEoYTMubWckQ1NUX00xKSwgIkNTVElWX3RyYW5zX2V4cCJdLCBhMy5tZ1shaXMubmEoYTMubWckQ1NUX00xKSwgInByZWdfb3V0Y29tZV9jbGVhbiJdKQoKIyMjIFRFU1QgVFJBTlNJVElPTiBUTyBDU1QgSVYgQVQgTTEgQU5EIHNQVEIgLS0gYW1vbmcgSElWLU5lZwp0aGUubW9kZWw8LWdsbShwcmVnX291dGNvbWVfY2xlYW4gfiBDU1RJVl90cmFuc19leHAgKyBzZF9hZ2UgKyBwaF9lZ2EgKyBncmF2aWRpdHkgKyBibHRlc3Rfc3lwcmVzdWx0cyArIHBoX2Rpc2NoYXJnZSArIHBoX3NvcmVzX3VsY2VycyArIHdhdGVyLCBkYXRhID0gYTMubWdbYTMubWckaGl2ICVpbiUgIkhJVi1OZWdhdGl2ZSIgJiAhaXMubmEoYTMubWckQ1NUX00xKSAgJiAhaXMubmEoYTMubWckcHJlZ19vdXRjb21lX2NsZWFuKSwgXSwgZmFtaWx5ID0gImJpbm9taWFsIikgCmNoYW5nZS50by5jc3Rpdi5zcHRiLmhpdm5lZzwtYXMuZGF0YS5mcmFtZShicm9vbTo6dGlkeSh0aGUubW9kZWwsIGNvbmYuaW50ID0gVCwgZXhwb25lbnRpYXRlID0gVCkpWzIsXQp0YWJsZShhMy5tZ1thMy5tZyRoaXYgJWluJSAiSElWLU5lZ2F0aXZlIiAmICFpcy5uYShhMy5tZyRDU1RfTTEpLCAiQ1NUSVZfdHJhbnNfZXhwIl0sIGEzLm1nW2EzLm1nJGhpdiAlaW4lICJISVYtTmVnYXRpdmUiICYgIWlzLm5hKGEzLm1nJENTVF9NMSksICJwcmVnX291dGNvbWVfY2xlYW4iXSkKYGBgCmBgYHtyfQpzYXZlLmltYWdlKCJNTEFXSV9Ib2xtX1NhaWRpXzIwMjQuUkRhdGEiKQpgYGAKCg==
